# Supplementary material for: Collective total synthesis of C4-oxygenated securinine-type alkaloids via stereocontrolled diversifications on the piperidine core
Source: Nat Commun. 2022 Sep 2;13:5149. doi: 10.1038/s41467-022-32902-z (PMC9440219; doi:10.1038/s41467-022-32902-z)
Supplement: Supplementary file 1 — Supplementary Information [file 41467_2022_32902_MOESM1_ESM.pdf]

Supplementary Information

Collective Total Synthesis of C4-Oxygenated Securinine-Type Alkaloids via Stereocontrolled Diversifications on the Piperidine Core

Sangbin Park,[1] Gyumin Kang,[1,2] Chansu Kim,[1] Dongwook Kim,[2] and Sunkyu Han[1,2,]*

[1]Department of Chemistry, Korea Advanced Institute of Science & Technology (KAIST), Daejeon 34141, Republic of Korea.
[2]Centerfor Catalytic Hyrdrocarbon Functionalizations, Institute for Basic Science (IBS), Daejeon 34141, Republic of Korea.
email: sunkyu.han@kaist.ac.kr

Table of Contents

Supplementary Methods S03
1. General Information S03
2. Experimental Procedures and Physical Data for Newly Synthesized Compounds S04
    2.1. (+)-Cyclohexenol **18** S04
    2.2. (–)-Nitrobenzoic ester **S1** S05
    2.3. (+)-Epoxide **19** S06
    2.4. (+)-γ-Hydroxyenone **20** S07
    2.5. (+)-γ-TBDPS-oxy enone **S2** S08
    2.6. (+)-α-Hydroxy ketone **21** S09
    2.7. (–)-α-Phosphonoacetatoxy enone **22** S10
    2.8. (–)-*O*-TBDPS-menisdaurilide **23** S11
    2.9. (–)-β-Hydroxy lactam **26** S12
    2.10. (–)-β-Methoxy lactam **27** S13
    2.11. (–)-ε-Hydroxy butenolide **29** S14
    2.12. (–)-Azabicyclo[2.2.2]octane **30** S16
    2.13. (+)-Securingine A (**7b**) S17
    2.14. (–)-Securitinine (**3**) S18
    2.15. (–)-Secu'amamine D (**6**) S19
    2.16. (–)-α,β-Epoxy lactam **36** S20
    2.17. (–)-ε-Hydroxy butenolide **38** S21
    2.18. (–)-Securingine C (**8b**) S23
    2.19. (–)-Securingine D (**9b**) S24
    2.20. (–)-Azabicyclo[2.2.2]octane **42** S25
    2.21. (–)-4-*epi*-Phyllanthine (**4**) S26
    2.22. (–)-*ent*-Virosine B (**44**) S27

2.23. (–)-Securinine (**1**) S28
2.24. (–)-Allosecurinine (**2**) S29
2.25. (+)-γ-Piperidone **46** S30
2.26. (+)-γ-Hydroxy piperidine **47** S31
2.27. (+)-γ-Hydroxy piperidine **48** S32
2.28. (+)-γ-Methoxy piperidine **S6** S34
2.29. (+)-ε-Hydroxy butenolide **49** S35
2.30. (–)-Azabicyclo[2.2.2]octane **50** S36
2.31. (–)-Phyllanthine (**5**) S37
2.32. (–)-Azabicyclo[2.2.2]octane **51** S38
2.33. (–)-4-*epi*-Securitinine (**52**) S39
Supplementary Discussion S40
3. Determination of Enantiomeric Excess (% ee) S40
3.1. Chiral HPLC spectra of (–)-Nitrobenzoic ester **S1** S40
3.2. Chiral HPLC spectra of (–)-*O*-TBDPS-menisdaurilide **23** S41
3.3. Chiral ^{1}H NMR with the Co complex of (–)-β-Methoxy lactam **27** S42
3.4. Chiral ^{1}H NMR with the Co complex of (–)-α,β-Epoxy lactam **36** S43
4. Comparison of Spectral Data of Synthetic Natural Products with Other Reports S44
4.1. NMR data of natural and synthetic (+)-Securingine A (**7b**) S44
4.2. NMR data of natural and synthetic (–)-Securitinine (**3**) S46
4.3. NMR data of natural and synthetic (–)-Secu'amamine D (**6**) S48
4.4. NMR data of natural and synthetic (–)-Securingine C (**8b**) S50
4.5. NMR data of natural and synthetic (–)-Securingine D (**9b**) S52
4.6. NMR data of natural and synthetic (–)-4-*epi*-Phyllanthine (**4**) S54
4.7. NMR data of natural and synthetic (–)-Phyllanthine (**5**) S56
5. Comparison of NMR spectra of authentic and synthetic natural products S58
6. Computational Studies Regarding the Thermodynamics of C2-Epimerization S64
6.1. Computational Details S64
6.2. Ground-state conformation and thermodynamics of C2-epimerization S65
6.3. DFT-optimized structure's energy components S67
7. Single Crystal X-Ray Diffraction (SCXD) Analysis Data of Securingine D (**9b**) S68
8. Copies of NMR spectra of newly synthesized compounds S77
Supplementary References S150

Supplementary Methods

1. General Information

All reactions were performed in oven-dried or flame-dried round-bottomed flasks and vials. Unless otherwise noted, the flasks were fitted with rubber septa and reactions were conducted under a positive pressure of argon, and vials were tightly sealed with plastic septa and parafilm. Stainless steel syringes or cannula were used to transfer air- and moisture-sensitive liquids. Flash column chromatography was performed as described by Still et al. using silica gel (60-Å pore size, 40–63 µm, 4-6% H_2O content, Merck).[1] Analytical thin–layer chromatography (TLC) was performed using glass plates pre-coated with 0.25 mm silica gel impregnated with a fluorescent indicator (254 nm). Thin layer chromatography plates were visualized by exposure to ultraviolet light, an aqueous ceriic ammonium molybdate (CAM) solution, and/or a aqueous potassium permanganate ($KMnO_4$) solution.

Unless otherwise stated, all commercial reagents and solvents were used without additional purification with the following exceptions as indicated below. Dichloromethane and tetrahydrofuran were purchased from Merck and Daejung Inc., respectively and were purified by the method of Grubbs et al. under positive argon pressure.[2]

^{1}H and ^{13}C nuclear magnetic resonance spectra were recorded with Bruker AVANCE NEO (500 MHz), Bruker AVANCE III HD Nanobay (400 MHz), Bruker AVANCE III HD (400 MHz), or Bruker AVANE NEO Nanobay (400 MHz) and calibrated by using the residual undeuterated chloroform (δ_H= 7.24 ppm) and $CDCl_3$ (δ_C= 77.23 ppm) or monodeuterated dichloromethane (δ_H= 5.32 ppm) as internal references. Data are reported in the following manners: chemical shift in ppm [multiplicity (s = singlet, d = doublet, t = triplet, q = quartet, p = quintet, m = multiplet, app = apparent, br = broad), coupling constant(s) in Hertz, integration]. The NMR solvent $CDCl_3$ was taken from a stock containing anhydrous K_2CO_3 to remove residual DCl. High resolution mass spectra were obtained from KAIST Analysis Center for Research Advancement (Daejeon) by using ESI ionization method. Specific rotation $[\alpha]_D^T$was obtained by JASCO P-2000 polarimeter.

2. Experimental Procedures and Physical Data for Newly Synthesized Compounds

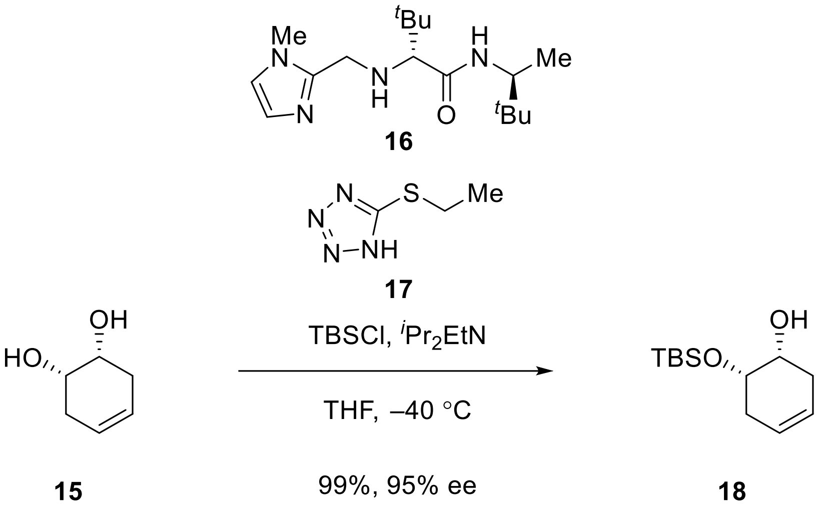

(+)-Cyclohexenol 18:

N,N-diisopropylethylamine (19.6 mL, 111.5 mmol, 1.2 equiv.) was added to a solution of an enantiomer of Hoveyda-Snapper catalyst **16** (5.74 g, 18.6 mmol, 0.2 equiv.), co-catalyst 5-ethylthiotetrazole **17** (1.24 g, 9.3 mmol, 0.1 equiv.), and diol **15** (10.61 g, 93.0 mmol, 1.0 equiv.) in tetrahydrofuran (74 mL) at –40 °C under argon atmosphere. Then, *tert*-butyldimethylsilyl chloride (28.89 g, 185.9 mmol, 2.0 equiv.) solution in tetrahydrofuran (65 mL) was added via cannula at –40 °C. After 1 h, the reaction was quenched with *N,N*-diisopropylethylamine (16.4 mL, 93.0 mmol, 1 equiv.) and methanol (8.2 mL), and the mixture was warmed to 23 °C. 10% aqueous citric acid solution (600 mL) was added and the layers were separated. The aqueous layer was extracted with dichloromethane (3 × 200 mL) and the combined organic layer was dried over anhydrous sodium sulfate. The resulting filtrate was concentrated under reduced pressure. The resulting crude residue was purified by flash column chromatography (silica gel: diam. 7 cm, ht. 10 cm; eluent: hexanes to ethyl acetate : hexanes = 1 : 19) to afford **18** (21.09 g, 99%) as a colorless oil. The chiral catalyst was recovered in the following manner: the aqueous layer was treated with a 3.0 N aqueous sodium hydroxide solution until pH = 12. The aqueous layer was extracted with dichloromethane (3 × 200 mL) and the combined organic layer was dried over anhydrous sodium sulfate. The resulting filtrate was concentrated under reduced pressure.

^{1}H NMR (500 MHz, $CDCl_3$): δ 5.56 – 5.49 (m, 2H), 3.90 – 3.83 (m, 2H), 2.32 – 2.12 (m, 5H), 0.88 (s, 9H), 0.07 (s, 3H), 0.06 (s, 3H).

^{13}C NMR (126 MHz, $CDCl_3$): δ 124.0, 123.7, 70.1, 69.4, 31.5, 30.8, 26.0, 18.3, -4.3, -4.6.

HRMS (ESI): Calculated for $C_{12}H_{24}O_2Si$ $[M+Na]^+$: 251.1438, found: 251.1450

TLC (ethyl acetate : hexanes= 1 : 9) R*f*: 0.40 ($KMnO_4$).

$[\boldsymbol{\alpha}]_{\boldsymbol{D}}^{\mathbf{25}}$: 29.4 (c 0.5, $CHCl_3$)

*Note: The synthesis of ent-**16** was reported in <Nature **443**, 67–70 (2006)>. Catalyst **16** was prepared using this protocol using enantiomeric starting materials.*

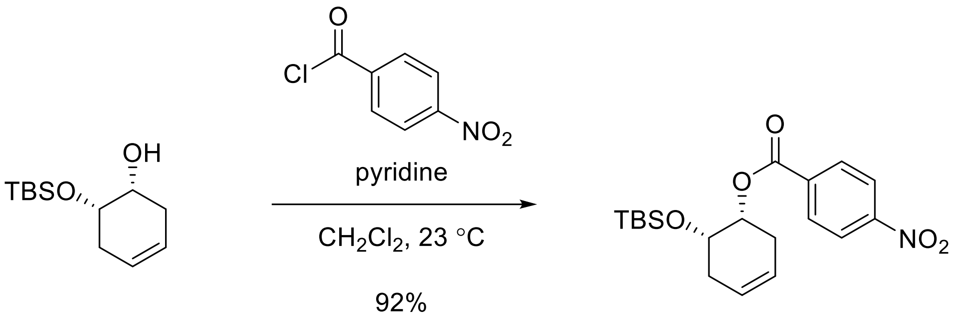

(–)-Nitrobenzoic ester S1:

Anhydrous pyridine (0.14 mL, 1.7 mmol, 10.0 equiv.) and 4-nitrobenzoyl chloride (48.2 mg, 0.255 mmol, 1.2 equiv.) were added to a solution of **18** (38.8 mg, 0.170 mmol, 1.0 equiv.) in dichloromethane (0.85 mL) under argon atmosphere. After 1 h, the reaction was quenched with saturated aqueous sodium bicarbonate solution (10 mL). The aqueous layer was extracted with dichloromethane (3 × 10 mL) and the combined organic layer was dried over anhydrous sodium sulfate. The resulting filtrate was concentrated under reduced pressure. The resulting crude residue was purified by flash column chromatography (silica gel: diam. 2 cm, ht. 11 cm; eluent: hexanes to ethyl acetate : hexanes = 1 : 25) to afford **S1** (58.9 mg, 92%) as a crystalline solid.

^{1}H NMR (500 MHz, $CDCl_3$): δ 8.29 – 8.16 (m, 4H), 5.65 – 5.56 (m, 2H), 5.28 (ddd, J = 6.8, 4.9, 2.1 Hz, 1H), 4.15 (ddd, J = 6.7, 4.9, 2.1 Hz, 1H), 2.55 – 2.47 (m, 1H), 2.44 – 2.33 (m, 2H), 2.32 – 2.24 (m, 1H), 0.83 (s, 9H), 0.02 (s, 3H), -0.01 (s, 3H).

^{13}C NMR (126 MHz, $CDCl_3$): δ 164.6, 150.7, 136.4, 130.9, 124.3, 123.7, 123.5, 74.1, 67.7, 32.8, 28.6, 25.9, 18.2, –4.4, –4.6.

HRMS (ESI): Calculated for $C_{19}H_{27}NO_5Si$ $[M+Na]^+$: 400.1550, found: 400.1550

TLC (ethyl acetate : hexanes= 1 : 19) R*f*: 0.35 (UV, $KMnO_4$).

$[\alpha]_D^{25}$: –20.3 (c 0.5, $CHCl_3$)

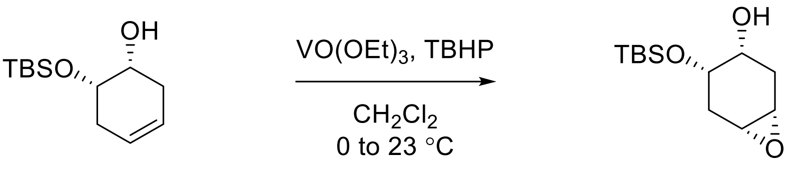

18 98% 19

(+)-Epoxide 19:

Vanadium oxytriethoxide (0.52 mL, 2.8 mmol, 0.05 equiv.) and *tert*-butyl hydroperoxide (5.5 M in decane, 10.1 mL, 55.8 mmol, 1.0 equiv.) were added to a solution of **18** (12.74 g, 55.8 mmol, 1.0 equiv.) in dichloromethane (56 mL) at 0 °C under argon atmosphere. The reaction mixture was warmed to 23 °C and allowed to stir for 7 h, during which the *tert*-butyl hydroperoxide solution (5.5 M in decane, 30.4 mL, 167.3 mmol, 3.0 equiv.) was added in six portions in every hour. The resulting mixture was quenched with saturated aqueous sodium thiosulfate solution (300 mL) and the layers were separated. The aqueous layer was extracted with diethyl ether (3 × 300 mL) and the combined organic layer was dried over anhydrous sodium sulfate. The resulting filtrate was concentrated under reduced pressure. The resulting crude residue was purified by flash column chromatography (silica gel: diam. 7 cm, ht. 16 cm; eluent: ethyl acetate : hexanes = 1 : 5) to afford **19** (13.41 g, 98%) as a colorless oil.

^{1}H NMR (500 MHz, $CDCl_3$): δ 3.72 – 3.63 (m, 2H), 3.22 – 3.11 (m, 2H), 2.63 (d, J = 8.8 Hz, 1H), 2.41 (ddt, J = 15.6, 2.9, 1.3 Hz, 1H), 2.16 – 2.11 (m, 2H), 1.98 (ddd, J = 15.8, 4.0, 2.1 Hz, 1H), 0.87 (s, 9H), 0.05 (s, 6H).

^{13}C NMR (126 MHz, $CDCl_3$): δ 70.0, 69.2, 52.4, 51.8, 30.5, 29.3, 26.1, 18.4, -4.4, -4.5.

HRMS (ESI): Calculated for $C_{12}H_{24}O_3Si$ $[M+Na]^+$: 267.1387, found: 267.1405

TLC (ethyl acetate : hexanes = 1 : 4) R*f*: 0.40 ($KMnO_4$).

$[\alpha]_D^{25}$: 0.8 (c 0.5, $CHCl_3$)

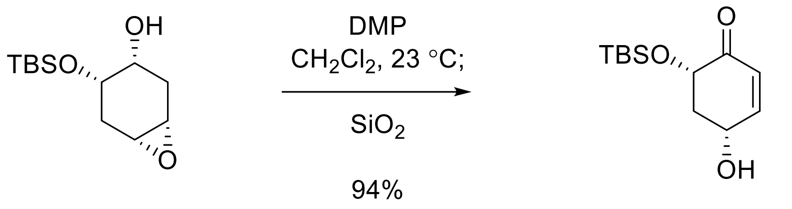

19 **20**

(+)-γ-Hydroxyenone 20:

Dess-Martin periodinane (45.58 g, 104.2 mmol, 1.25 equiv.) was added to a solution of **19** (20.38 g, 83.4 mmol, 1.0 equiv.) in dichloromethane (1190 mL). After 4 h, the reaction was quenched with saturated aqueous sodium thiosulfate solution (300 mL) and the mixture was stirred for 30 min. Then, saturated aqueous sodium bicarbonate solution (300 mL) was added and the layers were separated. The aqueous layer was extracted with dichloromethane (3 × 400 mL) and the combined organic layer was dried over anhydrous sodium sulfate. Excess SiO_2 (1000 g) was added to the resulting filtrate and concentrated under reduced pressure. After 36 hours, the mixture was washed with ethyl acetate and the resulting filtrate was concentrated under reduced pressure. The resulting crude residue was purified by flash column chromatography (silica gel: diam. 7 cm, ht. 12 cm; eluent: ethyl acetate : hexanes = 1 : 1) to afford **20** (19.01 g, 94%) as a white solid.

^{1}H NMR (500 MHz, $CDCl_3$): δ 6.89 (ddd, *J* = 10.2, 2.9, 1.4 Hz, 1H), 5.97 (dd, *J* = 10.2, 1.9 Hz, 1H), 4.53 (dtt, *J* = 8.0, 5.6, 2.3 Hz, 1H), 4.14 (dd, *J* = 10.6, 4.3 Hz, 1H), 2.50 – 2.44 (m, 2H), 2.16 – 2.08 (m, 1H), 0.88 (s, 9H), 0.13 (s, 3H), 0.08 (s, 3H).

^{13}C NMR (126 MHz, $CDCl_3$): δ 197.7, 151.3, 127.6, 72.7, 66.1, 41.5, 25.9, 18.5, -4.4, -5.2.

HRMS (ESI): Calculated for $C_{12}H_{22}O_3Si$ $[M+Na]^+$: 265.1230, found: 265.1230

TLC (ethyl acetate : hexanes = 1 : 1) R*f*: 0.30 (UV, $KMnO_4$).

$[\alpha]_D^{25}$: 31.1 (c 0.5, $CHCl_3$)

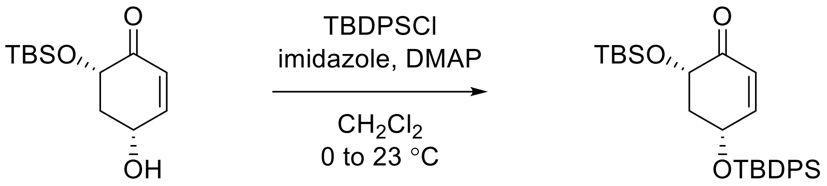

20 95% **S2**

(+)-γ-TBDPS-oxy enone S2:

tert-Butyl(chloro)diphenylsilane (16.1 mL, 60.0 mmol, 1.2 equiv.) was added dropwise to a solution of **20** (12.13 g, 50.0 mmol, 1.0 equiv.), imidazole (4.13 g, 60.0 mmol, 1.2 equiv.), and 4-dimethylaminopyridine (0.618 g, 5.0 mmol, 0.1 equiv.) in dichloromethane (500 mL) at 0 °C under argon atmosphere. The resulting mixture was heated to 23 °C. After 2 h, the reaction was quenched with saturated aqueous ammonium chloride solution (300 mL) and the layers were separated. The aqueous layer was extracted with dichloromethane (3 × 200 mL) and the combined organic layer was dried over anhydrous sodium sulfate. The resulting filtrate was concentrated under reduced pressure. The resulting crude residue was purified by flash column chromatography (silica gel: diam. 7 cm, ht. 12 cm; eluent: hexanes to ethyl acetate : hexanes = 1 : 30) to afford **S2** (22.92 g, 95%) as a white solid.

^{1}H NMR (500 MHz, $CDCl_3$): δ 7.69 – 7.65 (m, 4H), 7.48 – 7.35 (m, 6H), 6.78 (dt, *J* = 10.4, 2.0 Hz, 1H), 5.86 (dd, *J* = 10.2, 2.4 Hz, 1H), 4.55 (ddt, *J* = 10.0, 4.8, 2.1 Hz, 1H), 3.85 (dd, *J* = 13.3, 5.0 Hz, 1H), 2.25 – 2.17 (m, 1H), 2.09 (td, *J* = 12.7, 10.4 Hz, 1H), 1.07 (s, 9H), 0.82 (s, 9H), 0.05 (s, 3H), -0.07 (s, 3H).

^{13}C NMR (126 MHz, $CDCl_3$): δ 198.3, 153.4, 136.0, 136.0, 133.5, 133.4, 130.3, 130.3, 128.1, 128.1, 127.2, 72.6, 68.7, 43.5, 27.1, 26.0, 19.3, 18.7, -4.4, -5.3.

HRMS (ESI): Calculated for $C_{28}H_{40}O_3Si_2$ $[M+Na]^+$: 503.2408, found: 503.2453

TLC (ethyl acetate : hexanes = 1 : 19) R*f*: 0.15 (UV, $KMnO_4$).

$[\alpha]_D^{25}$: 2.1 (c 0.5, $CHCl_3$)

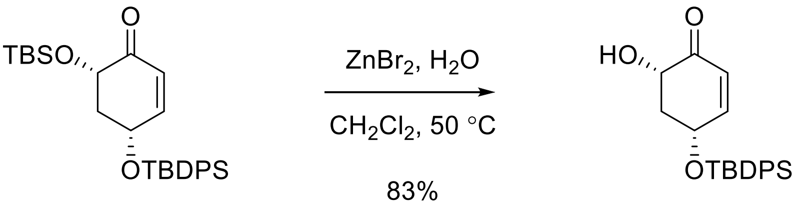

S2 **21**

(+)-α-Hydroxy ketone 21:

Zinc bromide (78.76 g, 349.4 mmol, 5.0 equiv.) and water (6.3 mL, 349.4 mmol, 5.0 equiv.) were added to a solution of **S2** (33.58 g, 69.9 mmol, 1.0 equiv.) in dichloromethane (140 mL), then the mixture was heated to 50 °C. After 2 h, the reaction mixture was diluted with dichloromethane (2000 mL). The organic layer was washed with water (2 × 100 mL) and saturated aqueous sodium bicarbonate solution (2 × 100 mL) and the resulting mixture was dried over anhydrous sodium sulfate. The resulting filtrate was concentrated under reduced pressure. The resulting crude residue was purified by flash column chromatography (silica gel: diam. 7 cm, ht. 14 cm; eluent: ethyl acetate : hexanes = 1 : 9 to 2 : 8) to afford **21** (21.29 g, 83%) as a colorless oil.

^{1}H NMR (500 MHz, $CDCl_3$): δ 7.70 – 7.64 (m, 4H), 7.48 – 7.36 (m, 6H), 6.86 (dt, *J* = 10.3, 2.0 Hz, 1H), 5.97 (dd, *J* = 10.2, 2.4 Hz, 1H), 4.59 (ddt, *J* = 10.4, 5.1, 2.2 Hz, 1H), 3.89 (ddd, *J* = 13.6, 5.3, 2.1 Hz, 1H), 3.48 (d, *J* = 2.0 Hz, 1H), 2.57 (dtd, *J* = 12.2, 5.2, 2.1 Hz, 1H), 2.00 (ddd, *J* = 13.6, 11.9, 10.3 Hz, 1H), 1.07 (s, 9H).

^{13}C NMR (126 MHz, $CDCl_3$): δ 199.7, 156.1, 136.0 (2), 133.3, 133.1, 130.3 (2), f128.2, 128.1, 125.5, z71.1, 68.2, 42.4, 27.0, 19.3.

HRMS (ESI): Calculated for $C_{22}H_{26}O_3Si$ $[M+Na]^+$: 389.1543, found: 389.1550

TLC (ethyl acetate : hexanes = 1 : 4) R*f*: 0.40 (UV, $KMnO_4$).

$[\alpha]_D^{25}$: 32.4 (c 0.5, $CHCl_3$)

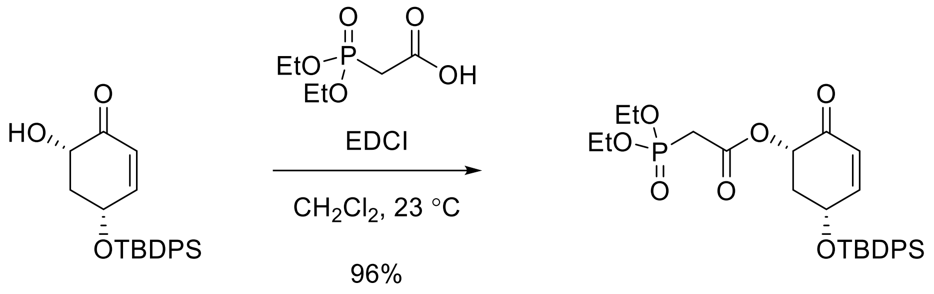

21 **22**

(–)-α-Phosphonoacetatoxy enone 22:

Diethylphosphonoacetic acid (21.0 mL, 127.7 mmol, 2.0 equiv.) and 1-ethyl-3-(3-dimethylaminopropyl)carbodiimide hydrochloride (24.99 g, 127.7 mmol, 2.0 equiv.) were added to a solution of **21** (23.41 g, 63.9 mmol, 1.0 equiv.) in dichloromethane (128 mL). After 30 min, the reaction mixture was quenched with saturated aqueous ammonium chloride solution (250 mL) and the layers were separated. The aqueous layer was extracted with dichloromethane (3 × 200 mL) and the combined organic layer was dried over anhydrous sodium sulfate. The resulting filtrate was concentrated under reduced pressure. The resulting crude residue was purified by flash column chromatography (silica gel: diam. 7 cm, ht. 10 cm; eluent: ethyl acetate : hexanes = 2 : 1) to afford **22** (33.25 g, 96%) as a yellow oil.

^{1}H NMR (500 MHz, $CDCl_3$): δ 7.67 – 7.62 (m, 4H), 7.47 – 7.36 (m, 6H), 6.77 (dt, J = 10.3, 2.0 Hz, 1H), 5.90 (dd, J = 10.3, 2.3 Hz, 1H), 5.10 (dd, J = 14.0, 5.0 Hz, 1H), 4.68 – 4.60 (m, 1H), 4.19 – 4.11 (m, 4H), 3.10 – 2.97 (m, 2H), 2.43 (ddd, J = 10.0, 5.1, 2.6 Hz, 1H), 2.26 (ddd, J = 14.1, 11.7, 10.3 Hz, 1H), 1.34 – 1.28 (m, 6H), 1.06 (s, 9H).

^{13}C NMR (126 MHz, $CDCl_3$): δ 192.4, 164.9, 164.9, 154.3, 135.9, 135.9, 133.0, 132.9, 130.4, 130.4, 128.2, 128.1, 126.8, 72.3, 68.0, 63.1, 63.0, 63.0, 62.9, 39.0, 34.6, 33.6, 27.0, 19.3, 16.5, 16.5.

HRMS (ESI): Calculated for $C_{28}H_{37}O_7PSi$ $[M+Na]^+$: 567.1938, found: 567.1948

TLC (ethyl acetate : hexanes = 4 : 1) R*f*: 0.20 (UV, $KMnO_4$).

$[\alpha]_D^{25}$: −2.9 (c 0.5, $CHCl_3$)

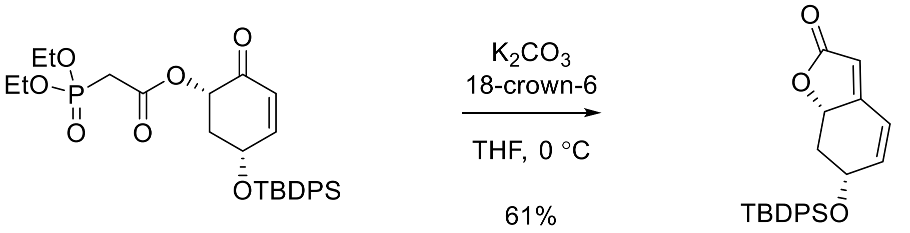

22 **23**

(–)-*O*-TBDPS-menisdaurilide 23:

Potassium carbonate (29.91 g, 212 mmol, 5.0 equiv.) and 18-crown-6 ether (57.02 g, 212 mmol, 5.0 equiv.) were dissolved in tetrahydrofuran (280 mL) at 0 °C under argon atmosphere. After 30 min, **22** (23.11 g, 42 mmol, 1.0 equiv.) solution in tetrahydrofuran (140 mL) was then added via cannula at 0 °C. After 9 h, the reaction mixture was quenched with saturated aqueous sodium bicarbonate solution (400 mL) and the layers were separated. The aqueous layer was extracted with ethyl acetate (2 × 300 mL) and the combined organic layer was dried over anhydrous sodium sulfate. The resulting filtrate was concentrated under reduced pressure. The resulting crude residue was purified by flash column chromatography (silica gel: diam. 7 cm, ht. 18 cm; eluent: ethyl acetate : hexanes = 1 : 6) to afford **23** (10.11 g, 61%) as a white solid.

^{1}H NMR (500 MHz, $CDCl_3$): δ 7.71 – 7.61 (m, 4H), 7.49 – 7.35 (m, 6H), 6.43 (dd, *J* = 9.9, 2.4 Hz, 1H), 6.18 (d, *J* = 10.0 Hz, 1H), 5.73 (s, 1H), 4.61 (ddd, *J* = 13.4, 4.9, 1.9 Hz, 1H), 4.52 (ddt, *J* = 10.0, 5.0, 2.3 Hz, 1H), 2.64 (dtt, *J* = 11.4, 5.1, 1.1 Hz, 1H), 1.76 (ddd, *J* = 13.4, 11.2, 10.0 Hz, 1H), 1.06 (s, 9H).

^{13}C NMR (126 MHz, $CDCl_3$): δ 173.4, 163.2, 144.5, 136.0, 135.9, 133.3, 133.2, 130.4, 130.3, 128.1 (2), 119.5, 111.3, 78.1, 68.3, 40.3, 27.0, 19.3.

HRMS (ESI): Calculated for $C_{24}H_{26}O_3Si$ $[M+Na]^+$: 413.1543, found: 413.1538

TLC (ethyl acetate : hexanes = 1 : 2) R*f*: 0.40 (UV, $KMnO_4$).

$[\alpha]_D^{25}$: –57.7 (c 2.0, $CHCl_3$)

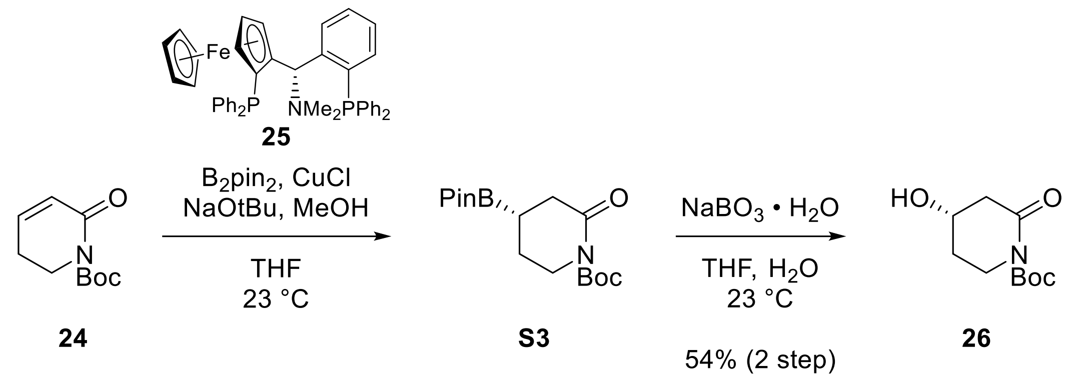

(–)-β-Hydroxy lactam 26:

In a glovebox, Copper chloride (6.2 mg, 0.063 mmol, 0.02 equiv.), sodium *tert*-butoxide (9.1 mg, 0.094 mmol, 0.03 equiv.), and taniaphos **25** (87 mg, 0.13 mmol, 0.04 equiv.) were dissolved in tetrahydrofuran (16 mL). After 30 min, bis(pinacolato)diboron (878mg, 3.46 mmol, 1.1 equiv.) was added. After 10 min, the resulting mixture and methanol (254 μL, 6.29 mmol, 2.0 equiv.) were added to **24** (620 mg, 3.14 mmol, 1.0 equiv.). After 24 h, the resulting mixture was filtered through a pad of celite and concentrated under reduced pressure.

The resulting crude residue was dissolved in tetrahydrofuran (31 mL) and water (31 mL). Sodium perborate monohydrate (1.57 g, 15.7 mmol, 5.0 equiv.) was added. After 1 h, the reaction was quenched with water (100 mL) and the layers were separated. The aqueous layer was extracted with ethyl acetate (3 × 100 mL) and the combined organic layer was dried over anhydrous sodium sulfate. The resulting filtrate was concentrated under reduced pressure. The resulting crude residue was purified by flash column chromatography (silica gel: diam. 4 cm, ht. 12 cm; eluent: acetone : hexanes = 1 : 3) to afford **26** (364 mg, 54% for 2 steps) as a white solid.

^{1}H NMR (400 MHz, $CDCl_3$): δ 4.24 – 4.15 (m, 1H), 3.82 (ddd, *J* = 12.9, 8.2, 4.7 Hz, 1H), 3.55 (ddd, *J* = 12.8, 7.0, 4.8 Hz, 1H), 2.70 (ddd, *J* = 17.1, 4.9, 1.1 Hz, 1H), 2.69 (s, 1H), 2.52 (ddd, *J* = 17.1, 5.9, 1.3 Hz, 1H), 2.08 – 1.95 (m, 1H), 1.91 – 1.81 (m, 1H), 1.48 (s, 9H).

^{13}C NMR (101 MHz, $CDCl_3$): δ 170.0, 152.5, 83.3, 64.3, 43.9, 42.3, 31.0, 28.2.

HRMS (ESI): Calculated for $C_{10}H_{17}NO_4$ $[M+Na]^+$: 238.1050, found: 238.1047

TLC (acetone : hexanes = 1 : 2) R*f*: 0.23 ($KMnO_4$).

$[\alpha]_D^{25}$: −13.1 (c 1.0, $CHCl_3$)

*Note: Taniaphos **25** was purchased from Strem Chemicals.*

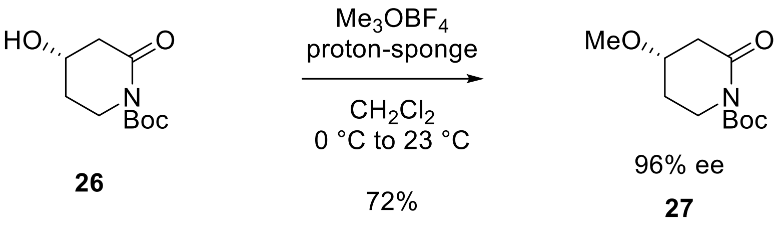

(–)-β-Methoxy lactam 27:

Proton-sponge (1.92 g, 8.98 mmol, 4.0 equiv.) and trimethyloxonium tetrafluoroborate (996 mg, 6.73 mmol, 3.0 equiv.) were added to a solution of **26** (483 mg, 2.24 mmol, 1.0 equiv.) in dichloromethane (22 mL) at 0 °C under argon atmosphere and the reaction mixture was slowly heated to 23 °C. After 4 h, the resulting mixture was diluted with ethyl acetate (100 mL) and filtered through a pad of celite. The resulting filtrate was washed with 10% aqueous citric acid solution (50 mL) and brine (50 mL), and the resulting mixture was dried over anhydrous sodium sulfate. The resulting filtrate was concentrated under reduced pressure. The resulting crude residue was purified by flash column chromatography (silica gel: diam. 2.5 cm, ht. 14 cm; eluent: acetone : hexanes = 1 : 6) to afford **27** (375 mg, 73%) as a colorless oil.

^{1}H NMR (400 MHz, $CDCl_3$): δ (400 MHz, Chloroform-*d*) δ 3.74 (ddd, *J* = 13.2, 8.6, 4.7 Hz, 1H), 3.69 – 3.63 (m, 1H), 3.57 (ddd, *J* = 13.0, 6.5, 4.9 Hz, 1H), 3.31 (s, 3H), 2.70 (ddd, *J* = 16.9, 4.9, 0.9 Hz, 1H), 2.60 (ddd, *J* = 17.0, 5.4, 1.3 Hz, 1H), 2.04 – 1.86 (m, 2H), 1.49 (s, 9H).

^{13}C NMR (101 MHz, $CDCl_3$): δ 169.4, 152.6, 83.2, 73.0, 56.2, 42.1, 40.8, 28.2, 28.0.

HRMS (ESI): Calculated for $C_{11}H_{19}NO_4$ $[M+Na]^+$: 252.1206, found: 252.1203

TLC (acetone : hexanes = 1 : 2) R*f*: 0.50 ($KMnO_4$).

$[\alpha]_D^{25}$: –9.2 (c 1.0, $CHCl_3$)

step. 1

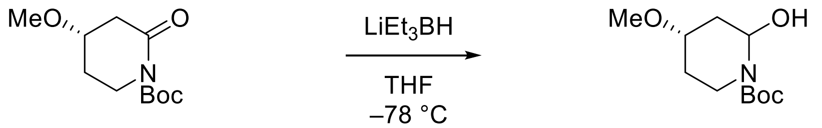

27 **28**

step. 2

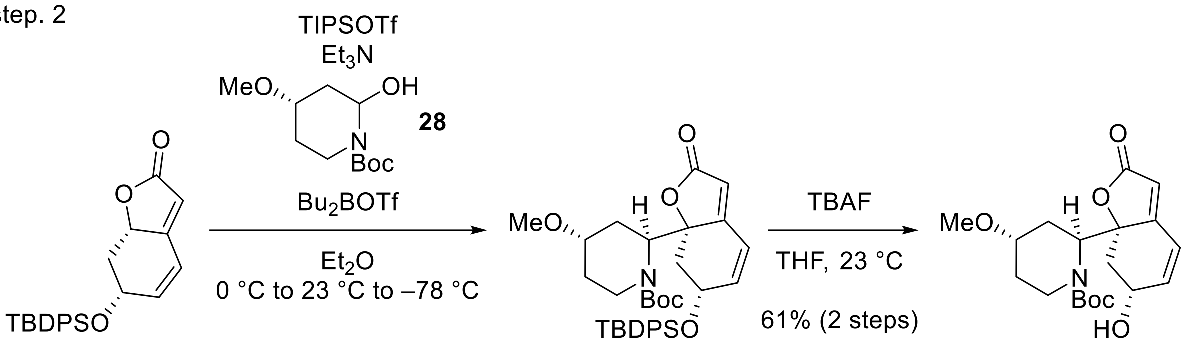

23 **S4** **29**

<u>(–)-ε-Hydroxy butenolide 29:</u>

Superhydride (1.0 M in tetrahydrofuran, 1.05 mL, 1.05 mmol, 1.8 equiv.) was added to a solution of **27** (200 mg, 0.872 mmol, 1.5 equiv.) in tetrahydrofuran (8.7 mL) at –78 °C under argon atmosphere. After 1 h, the reaction was quenched with saturated aqueous sodium bicarbonate solution (10 mL) and the layers were separated. The aqueous layer was extracted with dichloromethane (3 × 20 mL) and the combined organic layer was dried over anhydrous sodium sulfate. The resulting filtrate was concentrated under reduced pressure. The resulting crude of **28** was dissolved in diethyl ether (5.8 mL) under argon atmosphere.

At discrete round bottom flask, triethylamine (162 μL, 1.16 mmol, 2.0 equiv.) was added to a solution of **23** (227 mg, 0.581 mmol, 1.0 equiv.) in diethyl ether (5.8 mL) at 0 °C under argon atmosphere. After 45 min, triisopropylsilyl trifluoromethanesulfonate (187 μL, 0.697 mmol, 1.2 equiv.) was added, and the reaction mixture was slowly heated to 23 °C. After 24 h, **28** crude solution was added, and the reaction mixture was cooled to –78 °C. Then dibutylboryl trifluoromethanesulfonate (1.0 M in dichloromethane, 697 μL, 0.697 mmol, 1.2 equiv.) was added dropwise during 5 min. After additional 5 min, the reaction was quenched with saturated aqueous ammonium chloride solution (20 mL) and the layers were separated. The aqueous layer was extracted with ethyl acetate (3 × 40 mL) and the combined organic layer was dried over anhydrous sodium sulfate. The resulting filtrate was concentrated under reduced pressure.

The resulting crude of **S4** was dissolved in tetrahydrofuran (5.8 mL), and tetra-*n*-butylammonium fluoride (1.0 M in tetrahydrofuran, 1.28 mL, 1.28 mmol, 2.2 equiv.) was added under argon atmosphere. After 1 h, the reaction was quenched with saturated aqueous ammonium chloride solution (20 mL) and the layers were separated. The aqueous layer was extracted with ethyl acetate (3 × 40 mL) and the combined organic layer was dried over anhydrous sodium sulfate. The resulting filtrate was concentrated under reduced pressure. The

resulting crude residue was purified by flash column chromatography (silica gel: diam. 2.5cm, ht. 12 cm; eluent: acetone : hexanes = 1 : 4) to **29** (130 mg, 61% for 2 steps) as a white solid.

^{1}H NMR (400 MHz, $CDCl_3$, major rotamer): δ 6.56 (dd, *J* = 10.0, 2.2 Hz, 1H), 6.19 (d, *J* = 9.3 Hz, 1H), 5.70 (s, 1H), 4.55 (br s, 1H), 4.40 (dd, *J* = 7.0, 3.3 Hz, 1H), 3.95 (ddd, *J* = 14.1, 6.0, 2.1 Hz, 1H), 3.83 – 3.72 (m, 1H), 3.34 (s, 3H), 3.01 (td, *J* = 13.6, 3.8 Hz, 1H), 2.91 (dd, *J* = 12.5, 5.5 Hz, 1H), 2.36 – 2.24 (m, 1H), 2.18 (br s, 1H), 2.01 – 1.89 (m, 1H), 1.74 (dd, *J* = 12.6, 10.5 Hz, 1H), 1.66 (ddd, *J* = 14.6, 10.9, 7.4 Hz, 1H), 1.34 (s, 9H), 1.28 (ddd, *J* = 13.0, 9.2, 6.0 Hz, 1H).

^{13}C NMR (101 MHz, $CDCl_3$, major rotamer): δ 172.3, 165.9, 155.2, 139.1, 122.5, 111.8, 89.8, 80.4, 73.1, 65.8, 56.0, 52.0, 41.8, 41.0, 30.6, 30.2, 28.3.

HRMS (ESI): Calculated for $C_{19}H_{27}NO_6$ $[M+Na]^+$: 388.1731, found: 388.1753

TLC (acetone : hexanes = 1 : 2) R*f*: 0.25 (UV, $KMnO_4$).

$[\boldsymbol{\alpha}]_{\boldsymbol{D}}^{\mathbf{25}}$: –37.4 (c 1.0, $CHCl_3$)

Note: ^{1}H-NMR spectrum shows two sets of signals, due to the presence of two rotamers in 80:20 ratio. This assignation was corroborated with the same ^{1}H-NMR and EXSY experiments, where exchange signals between absorptions of the same proton but corresponding to different rotamers, were observed. This behavior could also be observed in the ^{13}C-NMR spectrum.

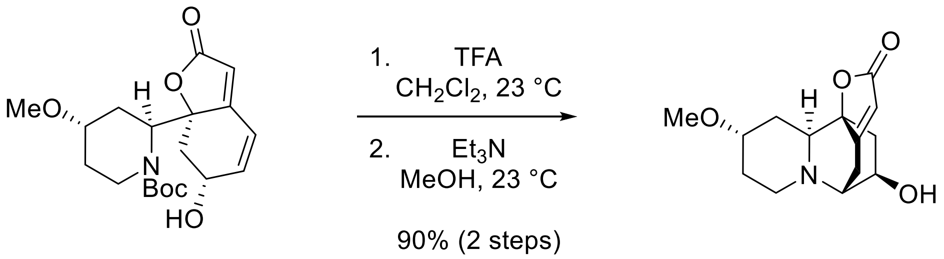

29 **30**

(–)-Azabicyclo[2.2.2]octane 30:

Trifluoroacetic acid (1.2 mL) was added to a solution of **29** (43 mg, 0.118 mmol, 1.0 equiv.) in dichloromethane (1.2 mL). After 30 min, the resulting mixture was concentrated via air blowing. The resulting crude residue was dissolved in methanol (1.2 mL). Triethylamine (0.6 mL) was added and the mixture was heated to 50 °C. After 2 h, the resulting mixture was concentrated under reduced pressure. The resulting crude residue was purified by flash column chromatography (silica gel: diam. 1.5 cm, ht. 12 cm; eluent: acetone : hexanes = 1 : 2) to afford **30** (28 mg, 90% for 2 steps) as a white gum.

^{1}H NMR (500 MHz, $CDCl_3$): δ 5.67 (t, *J* = 1.8 Hz, 1H), 4.36 (dt, *J* = 9.5, 3.7 Hz, 1H), 3.57 – 3.51 (m, 1H), 3.26 (s, 3H), 3.12 (dd, *J* = 11.7, 2.1 Hz, 1H), 2.99 – 2.89 (m, 2H), 2.87 (t, *J* = 3.6 Hz, 1H), 2.81 – 2.74 (m, 1H), 2.73 – 2.63 (m, 2H), 2.33 (br s, 1H), 1.83 (dt, *J* = 12.9, 2.2 Hz, 1H), 1.77 (dt, *J* = 13.7, 2.6 Hz, 1H), 1.69 – 1.59 (m, 1H), 1.43 (dd, *J* = 12.4, 4.7 Hz, 1H), 0.95 (td, *J* = 12.3, 3.0 Hz, 1H).

^{13}C NMR (126 MHz, $CDCl_3$): δ 174.4, 174.2, 111.8, 84.2, 74.3, 65.1, 58.8, 58.7, 56.2, 47.8, 40.8, 31.6, 29.4, 29.3.

HRMS (ESI): Calculated for $C_{14}H_{19}NO_4$ $[M+H]^+$: 266.1387, found: 266.1398

TLC (acetone : hexanes = 1 : 1) R*f*: 0.37 ($KMnO_4$).

$[\alpha]_D^{25}$: −35.6 (c 1.0, $CHCl_3$)

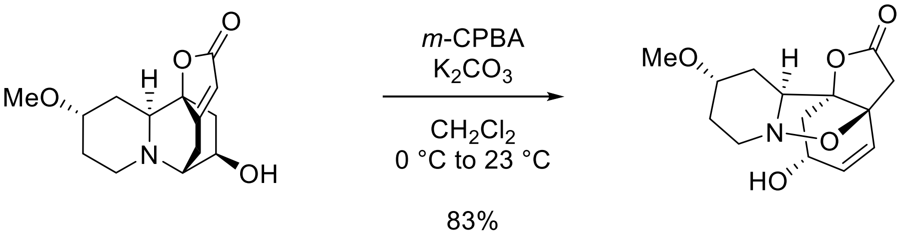

30 **7b** securingine A

(+)-Securingine A (7b):

meta-Chloroperoxybenzoic acid (77%, 15 mg, 0.0663 mmol, 1.1 equiv.) was added to a solution of **30** (16 mg, 0.0603 mmol, 1.0 equiv.) in dichloromethane (1.2 mL) at 0 °C under argon atmosphere. After 10 min, potassium carbonate (15 mg, 0.181 mmol, 3.0 equiv.) was added at 0 °C and the resulting mixture was slowly warmed to 23 °C. After 2.5 h, the reaction was quenched with brine (5 mL) and the layers were separated. The aqueous layer was extracted with ethyl acetate (3 × 10 mL) and the combined organic layer was dried over anhydrous sodium sulfate. The resulting filtrate was concentrated under reduced pressure. The resulting crude residue was purified by flash column chromatography (silica gel: diam. 1.5 cm, ht. 12 cm; eluent: acetone : hexanes = 1 : 3) to afford securingine A (**7b**) (14 mg, 83%) as a white solid.

^{1}H NMR (500 MHz, $CDCl_3$): δ 6.07 (dd, *J* = 10.2, 2.7 Hz, 1H), 5.88 (dd, *J* = 10.1, 1.8 Hz, 1H), 4.38 (s, 1H), 3.58 – 3.52 (m, 1H), 3.29 (s, 3H), 3.17 (ddd, *J* = 9.0, 4.3, 2.6 Hz, 1H), 3.01 (d, *J* = 18.7 Hz, 1H), 2.72 (d, *J* = 18.7 Hz, 1H), 2.73 – 2.66 (m, 1H), 2.46 (dd, *J* = 12.2, 2.6 Hz, 1H), 2.37 (dd, *J* = 13.5, 4.7 Hz, 1H), 2.07 (dd, *J* = 13.8, 2.8 Hz, 1H), 2.06 – 1.98 (m, 1H), 1.96 (d, *J* = 5.0 Hz, 1H), 1.83 (dd, *J* = 13.5, 9.1 Hz, 1H), 1.74 – 1.62 (m, 2H).

^{13}C NMR (126 MHz, $CDCl_3$): δ 174.6, 134.7, 126.9, 93.6, 81.0, 72.3, 67.1, 64.2, 56.1, 49.5, 42.3, 37.5, 28.9, 28.1.

HRMS (ESI): Calculated for $C_{14}H_{19}NO_5$ $[M+Na]^+$: 304.1155, found: 304.1144

TLC (acetone : hexanes = 1 : 2) R*f*: 0.24 ($KMnO_4$).

$[\boldsymbol{\alpha}]_D^{25}$: 142.1 (c 0.1, MeOH) [Lit. 36.0 (c 0.1, MeOH)][3]

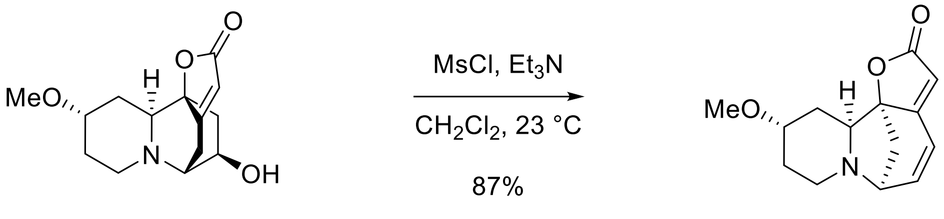

30 **3** securitinine

(–)-Securitinine (3):

Triethylamine (85 µL, 0.611 mmol, 6.0 equiv.) and methanesulfonyl chloride (24 µL, 0.305 mmol, 3.0 equiv.) were added to a solution of **30** (27 mg, 0.102 mmol, 1.0 equiv.) in dichloromethane (1 mL) at 0 °C. After 30 min, the reaction was quenched with saturated aqueous sodium bicarbonate solution (5 mL) and the layers were separated. The aqueous layer was extracted with ethyl acetate (3 × 10 mL) and the combined organic layer was dried over anhydrous sodium sulfate. The resulting filtrate was concentrated under reduced pressure. The resulting crude residue was purified by flash column chromatography (silica gel: diam. 1.5 cm, ht. 10 cm; eluent: acetone : hexanes = 1 : 1) to afford securitinine (**3**) (22 mg, 87%) as a yellow solid.

^{1}H NMR (400 MHz, $CDCl_3$): δ 6.78 (dd, *J* = 9.1, 5.2 Hz, 1H), 6.63 (dd, *J* = 9.1, 1.1 Hz, 1H), 5.72 (s, 1H), 3.89 (dd, *J* = 5.3, 4.5 Hz, 1H), 3.87 (dd, *J* = 13.6, 3.4 Hz, 1H), 3.62 (dddd, *J* = 8.7, 6.0, 4.6, 1.6 Hz, 1H), 3.21 (s, 3H), 2.79 (dt, *J* = 10.7, 4.1 Hz, 1H), 2.68 (dd, *J* = 9.8, 4.4 Hz, 1H), 2.58 (ddd, *J* = 13.3, 10.7, 3.1 Hz, 1H), 2.12 (dddd, *J* = 14.1, 8.5, 3.8, 2.8 Hz, 1H), 1.90 (d, *J* = 9.8 Hz, 1H), 1.68 – 1.52 (m, 2H), 1.17 (dt, *J* = 13.7, 4.7 Hz, 1H).

^{13}C NMR (101 MHz, $CDCl_3$): δ 172.7, 167.6, 149.1, 123.0, 109.5, 91.7, 73.0, 58.9, 56.1 (2), 43.1, 42.4, 30.8, 26.5.

HRMS (ESI): Calculated for $C_{14}H_{17}NO_3$ $[M+H]^+$: 248.1281, found: 248.1290

TLC (acetone : hexanes = 1 : 1) R*f*: 0.22 (UV, $KMnO_4$).

$[\boldsymbol{\alpha}]_D^{25}$: –819.7 (c 1.0, EtOH) [Lit. –952.3 (c 1.0, EtOH)][4]

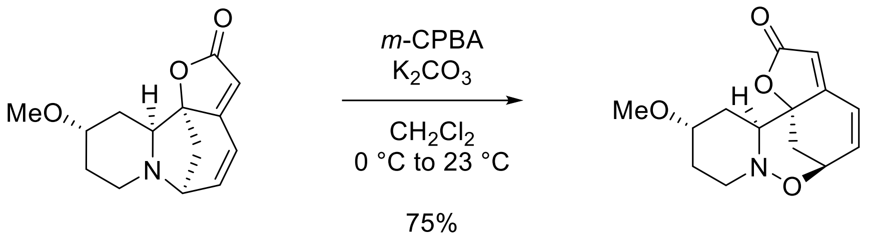

3 securitinine　　　　　　**6** secu'amamine D

(–)-Secu’amamine D (6):

meta-Chloroperoxybenzoic acid (77%, 25 mg, 0.111 mmol, 1.1 equiv.) was added to a solution of securitinine (**3**) (25 mg, 0.101 mmol, 1.0 equiv.) in dichloromethane (5 mL) at 0 °C under argon atmosphere. After 10 min, potassium carbonate (42 mg, 0.303 mmol, 3.0 equiv.) was added at 0 °C and the resulting mixture was slowly warmed to 23 °C. After 12 h, the reaction was quenched with brine (10 mL) and the layers were separated. The aqueous layer was extracted with ethyl acetate (3 × 20 mL) and the combined organic layer was dried over anhydrous sodium sulfate. The resulting filtrate was concentrated under reduced pressure. The resulting crude residue was purified by flash column chromatography (silica gel: diam. 1.5 cm, ht. 12 cm; eluent: acetone : hexanes = 1 : 4) to afford secu’amamine D (**6**) (20 mg, 75%) as a white solid.

^{1}H NMR (400 MHz, $CDCl_3$): δ 6.85 (d, J = 9.4 Hz, 1H), 6.27 (dd, J = 9.4, 5.8 Hz, 1H), 5.82 (s, 1H), 4.71 (ddd, J = 5.9, 3.4, 2.4 Hz, 1H), 3.40 (dt, J = 5.6, 2.8 Hz, 1H), 3.26 (s, 3H), 3.16 (dd, J = 12.0, 2.6 Hz, 1H), 2.99 – 2.91 (m, 1H), 2.95 – 2.84 (m, 1H), 2.51 (dd, J = 11.4, 3.4 Hz, 1H), 2.05 – 1.95 (m, 2H), 1.88 (ddt, J = 14.2, 5.8, 3.0 Hz, 1H), 1.64 (dddd, J = 14.2, 12.6, 5.4, 2.8 Hz, 1H), 0.99 (ddd, J = 13.7, 12.0, 2.6 Hz, 1H).

^{13}C NMR (101 MHz, $CDCl_3$): δ 172.1, 164.4, 134.6, 126.6, 113.6, 82.8, 72.2, 71.1, 65.4, 56.0, 50.4, 40.8, 29.4, 27.2.

HRMS (ESI): Calculated for $C_{14}H_{17}NO_4$ $[M+Na]^+$: 286.1050, found: 286.1067

TLC (acetone : hexanes = 1 : 2) R*f*: 0.42 (UV, $KMnO_4$).

$[\alpha]_D^{25}$: –375.5 (c 0.25, $CHCl_3$) [Lit. –303.9 (c 0.26, $CHCl_3$)][5]

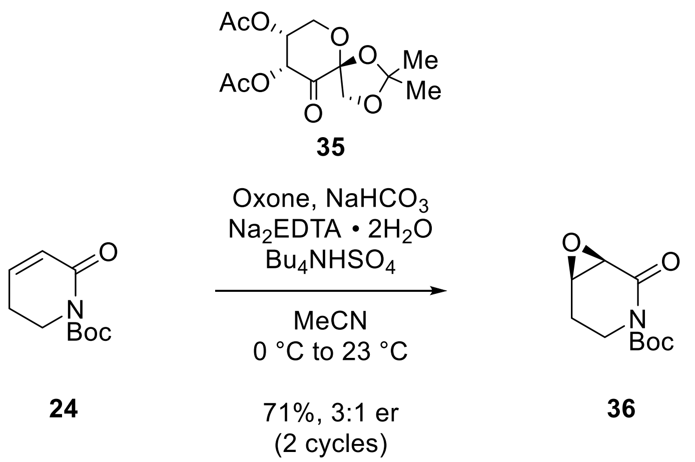

(–)-α,β-Epoxy lactam 36:

Disodium ethylenediaminetetraacetate dihydrate (0.5 mg, 1.27 μmol, 0.0005 equiv.) solution in water (12.7 mL) and tetrabutylammonium hydrogensulfate (86 mg, 0.254 mmol, 0.1 equiv.) were added to a solution of **24** (500 mg, 0.254 mmol, 1.0 equiv.) in acetonitrile (12.7 mL) at 0 °C. A mixture of Oxone (15.6 g, 25.3 mmol, 10.0 equiv.) and sodium bicarbonate (6.39g, 76.1 mmol, 30.0 equiv.) was pulverized, and a small portion of this mixture was added to the reaction mixture for pH > 7. Then, a solution of **35** (383 mg, 1.27 mmol, 0.5 equiv.) in acetonitrile (12.7 mL) was added. The rest of the oxone and sodium bicarbonate mixture was added portionwise over 4.5 h, and the resulting mixture was warmed to 23 °C. After additional 24 h, the reaction was quenched with saturated brine (60 mL) and the layers were separated. The aqueous layer was extracted with ethyl acetate (3 × 100 mL) and the combined organic layer was dried over anhydrous sodium sulfate. The resulting filtrate was concentrated under reduced pressure.

In the resulting crude, the starting material **24** remained and it could not be separated from the product **36**. To solve this problem, the resulting crude residue was subjected to the aforementioned asymmetric epoxidation protocol once more. Then, the resulting crude residue was purified by flash column chromatography (silica gel: diam. 2.5cm, ht. 13 cm; eluent: ethyl acetate : hexanes = 1 : 4) to **36** (383 mg, 71%) as a colorless oil.

^{1}H NMR (400 MHz, $CDCl_3$): δ 3.93 (ddt, *J* = 13.1, 5.7, 1.6 Hz, 1H), 3.64 – 3.57 (m, 1H), 3.51 (d, *J* = 4.1 Hz, 1H), 3.36 (td, *J* = 13.1, 3.9 Hz, 1H), 2.38 – 2.28 (m, 1H), 2.04 (ddd, *J* = 14.9, 13.1, 5.7 Hz, 1H), 1.50 (s, 9H).

^{13}C NMR (101 MHz, $CDCl_3$): δ 167.6, 151.8, 83.9, 53.3, 52.8, 38.4, 28.2, 24.3.

HRMS (ESI): Calculated for $C_{10}H_{15}NO_4$ $[M+Na]^+$: 236.0893, found: 236.0876

TLC (ethyl acetate : hexanes = 1 : 2) R*f*: 0.34 (CAM).

$[\alpha]_D^{25}$: –48.0 (c 1.0, $CHCl_3$)

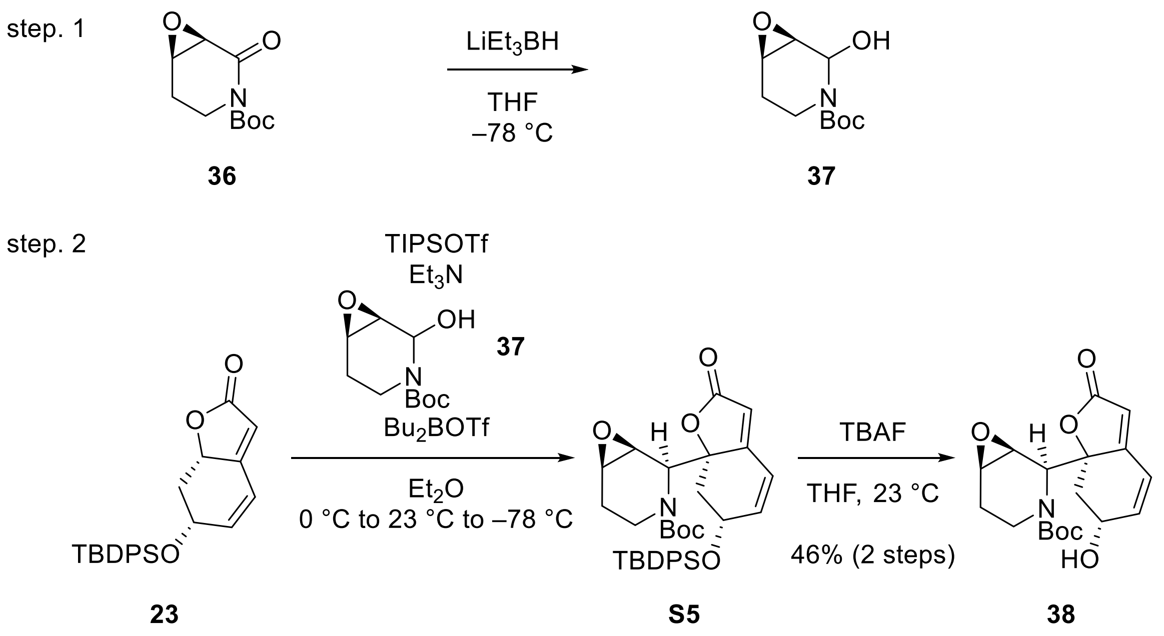

(–)-ε-Hydroxy butenolide 38:

Superhydride (1.0 M in tetrahydrofuran, 1.55 mL, 1.55 mmol, 1.8 equiv.) was added to a solution of **36** (276 mg, 1.29 mmol, 1.5 equiv.) in tetrahydrofuran (13 mL) at –78 °C under argon atmosphere. After 1 h, the reaction was quenched with saturated aqueous sodium bicarbonate solution (10 mL) and the layers were separated. The aqueous layer was extracted with dichloromethane (3 × 20 mL) and the combined organic layer was dried over anhydrous sodium sulfate. The resulting filtrate was concentrated under reduced pressure. The resulting crude of **37** was dissolved in diethyl ether (8.6 mL) under argon atmosphere.

At discrete round bottom flask, triethylamine (241 μL, 1.73 mmol, 2.0 equiv.) was added to a solution of **23** (337 mg, 0.863 mmol, 1.0 equiv.) in diethyl ether (8.6 mL) at 0 °C under argon atmosphere. After 45 min, triisopropylsilyl trifluoromethanesulfonate (278 μL, 1.04 mmol, 1.2 equiv.) was added and the reaction mixture was slowly heated to 23 °C. After 24 h, the reaction mixture was cooled to –78 °C and **37** crude solution was added. Then dibutylboryl trifluoromethanesulfonate (1.0 M in dichloromethane, 1.04 mL, 1.04 mmol, 1.2 equiv.) was added dropwise during 10 min. After additional 10 min, the reaction was quenched with saturated aqueous ammonium chloride solution (20 mL) and the layers were separated. The aqueous layer was extracted with ethyl acetate (3 × 40 mL) and the combined organic layer was dried over anhydrous sodium sulfate. The resulting filtrate was concentrated under reduced pressure.

The resulting crude of **S5** was dissolved in tetrahydrofuran (8.6 mL), and tetra-*n*-butylammonium fluoride (1.0 M in tetrahydrofuran, 1.9 mL, 1.90 mmol, 2.2 equiv.) was added under argon atmosphere. After 1 h, the reaction was quenched with saturated aqueous ammonium chloride solution (20 mL) and the layers were separated. The aqueous layer was extracted with ethyl acetate (3 × 40 mL) and the combined organic layer was dried over anhydrous sodium sulfate. The resulting filtrate was concentrated under reduced pressure. The

resulting crude residue was purified by flash column chromatography (silica gel: diam. 2.5cm, ht. 14 cm; eluent: acetone : hexanes = 1 : 3) to **38** (139 mg, 46% for 2 steps) as a white solid.

^{1}H NMR (400 MHz, $CDCl_3$, major rotamer): δ 6.58 (dd, *J* = 10.0, 2.2 Hz, 1H), 6.25 (d, *J* = 10.0 Hz, 1H), 5.69 (s, 1H), 4.69 (br s, 1H), 4.47 (d, *J* = 4.5 Hz, 1H), 3.69 (dd, *J* = 14.1, 5.0 Hz, 1H), 3.46 (t, *J* = 4.4 Hz, 1H), 3.34 (br s, 1H), 3.21 – 3.00 (m, 2H), 2.77 (br s, 1H), 1.96 (dd, *J* = 14.7, 2.9 Hz, 1H), 1.86 (dd, *J* = 12.6, 10.5 Hz, 1H), 1.82 – 1.68 (m, 1H), 1.32 (s, 9H).

^{13}C NMR (101 MHz, $CDCl_3$, major rotamer): δ 172.5, 164.4, 154.9, 139.7, 121.9, 112.1, 88.6, 80.8, 65.9, 51.7, 51.0, 50.9, 40.8, 37.0, 28.2, 25.0.

HRMS (ESI): Calculated for $C_{18}H_{23}NO_6$ $[M+Na]^+$: 372.1418, found: 372.1404

TLC (acetone : hexanes = 1 : 1) R*f*: 0.43 (UV, $KMnO_4$).

$[\alpha]_D^{25}$: –96.4 (c 1.0, $CHCl_3$)

Note: ^{1}H-NMR spectrum shows two sets of signals, due to the presence of two rotamers in 82:18 ratio. This assignation was corroborated with the same ^{1}H-NMR and EXSY experiments, where exchange signals between absorptions of the same proton but corresponding to different rotamers, were observed. This behavior could also be observed in the ^{13}C-NMR spectrum.

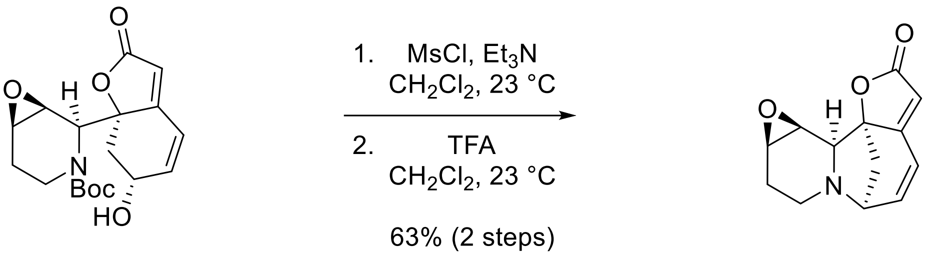

38 **8b** securingine C

(–)-Securingine C (8b):

Triethylamine (235 µL, 1.68 mmol, 6.0 equiv.) and methanesulfonyl chloride (65 µL, 0.842 mmol, 3.0 equiv.) were added to a solution of **38** (98 mg, 0.280 mmol, 1.0 equiv.) in dichloromethane (3 mL) at 0 °C. After 30 min, the reaction was quenched with saturated aqueous sodium bicarbonate solution (10 mL) and the layers were separated. The aqueous layer was extracted with ethyl acetate (3 × 20 mL) and the combined organic layer was dried over anhydrous sodium sulfate. The resulting filtrate was concentrated under reduced pressure.

The resulting crude residue was dissolved in dichloromethane (3 mL), and trifluoroacetic acid (3 mL) was added. After 30 min, the resulting mixture was concentrated via air blowing. The resulting crude residue was diluted in ethyl acetate (3 mL), and saturated aqueous sodium bicarbonate solution (10 mL) was poured. Then, the layers were separated. The aqueous layer was extracted with ethyl acetate (3 × 20 mL) and the combined organic layer was dried over anhydrous sodium sulfate. The resulting filtrate was concentrated under reduced pressure. The resulting crude residue was purified by flash column chromatography (silica gel: diam. 1.5 cm, ht. 14 cm; eluent: acetone : hexanes = 1 : 1) to afford securingine C (**8b**) (41 mg, 63% for 2 steps) as a white solid.

^{1}H NMR (400 MHz, $CDCl_3$): δ 6.80 (dd, *J* = 9.1, 5.1 Hz, 1H), 6.74 (dd, *J* = 9.0, 1.3 Hz, 1H), 5.77 (s, 1H), 3.99 (s, 1H), 3.83 (t, *J* = 4.7 Hz, 1H), 3.22 (t, *J* = 4.0 Hz, 1H), 3.04 (d, *J* = 4.7 Hz, 1H), 2.85 – 2.77 (m, 1H), 2.77 – 2.69 (m, 1H), 2.60 (dd, *J* = 9.9, 4.6 Hz, 1H), 2.09 – 1.98 (m, 2H), 1.85 (d, *J* = 9.9 Hz, 1H).

^{13}C NMR (101 MHz, $CDCl_3$): δ 172.6, 167.8, 148.0, 123.8, 109.1, 90.3, 59.7, 58.8, 49.9, 49.4, 43.3, 42.3, 24.2.

HRMS (ESI): Calculated for $C_{13}H_{13}NO_3$ $[M+H]^+$: 232.0968, found: 232.0946

TLC (acetone : hexanes = 1 : 1) R*f*: 0.24 (UV, $KMnO_4$).

$[\alpha]_D^{25}$: –645.8 (c 0.1, MeOH) [Lit. –243.0 (c 0.1, MeOH)][3]

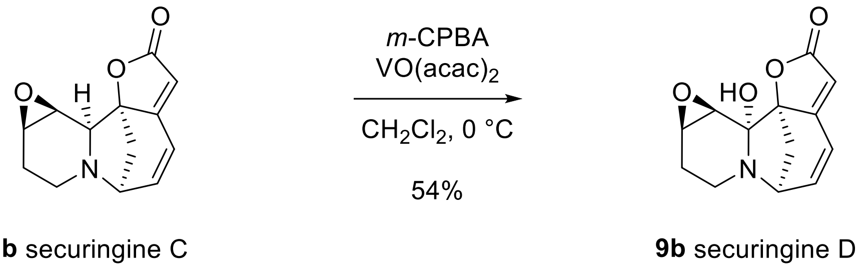

(–)-Securingine D (9b):

meta-Chloroperoxybenzoic acid (77%, 20 mg, 0.0904 mmol, 1.1 equiv.) was added to a solution of securingine C (**8b**) (19 mg, 0.0822 mmol, 1.0 equiv.) in dichloromethane (0.8 mL) at 0 °C under argon atmosphere. After 20 min, vanadyl acetylacetonate (22 mg, 0.0822 mmol, 1.0 equiv.) was added at 0 °C. After 25 min, saturated aqueous sodium bicarbonate solution (3 mL) was poured at 0 °C, and the reaction mixture was warmed to 23 °C for 15 min. Then, the layers were separated. The aqueous layer was extracted with ethyl acetate (3 × 10 mL) and the combined organic layer was dried over anhydrous sodium sulfate. The resulting filtrate was concentrated under reduced pressure. The resulting crude residue was purified by flash column chromatography (silica gel: diam. 1.5 cm, ht. 14 cm; eluent: acetone : hexanes = 1 : 1) to afford securingine D (**9b**) (11 mg, 54%) as a white solid.

^{1}H NMR (400 MHz, $CDCl_3$): δ 6.85 (dd, *J* = 9.0, 5.2 Hz, 1H), 6.72 (dd, *J* = 9.0, 1.3 Hz, 1H), 5.86 (s, 1H), 3.88 (t, *J* = 4.8 Hz, 1H), 3.29 (t, *J* = 4.1 Hz, 1H), 2.97 (d, *J* = 4.5 Hz, 1H), 2.89 (ddd, *J* = 9.5, 5.2, 2.1 Hz, 1H), 2.84 (dd, *J* = 10.0, 4.8 Hz, 1H), 2.77 (ddd, *J* = 13.2, 9.4, 4.0 Hz, 1H), 2.62 (br s, 1H), 2.25 (ddd, *J* = 14.7, 12.7, 5.2 Hz, 1H), 2.18 – 2.05 (m, 1H), 1.85 (d, *J* = 10.0 Hz, 1H).

^{13}C NMR (101 MHz, $CDCl_3$): δ 172.2, 164.1, 147.4, 123.7, 110.1, 91.4, 86.4, 56.3, 51.3, 49.6, 40.7, 40.6, 23.2.

HRMS (ESI): Calculated for $C_{13}H_{13}NO_4$ $[M+Na]^+$: 248.0917, found: 248.0920

TLC (acetone : hexanes = 1 : 1) R*f*: 0.26 (UV, $KMnO_4$).

$[\alpha]_D^{25}$: –418.9 (c 0.1, MeOH) [Lit. –202.7 (c 0.1, MeOH)][3]

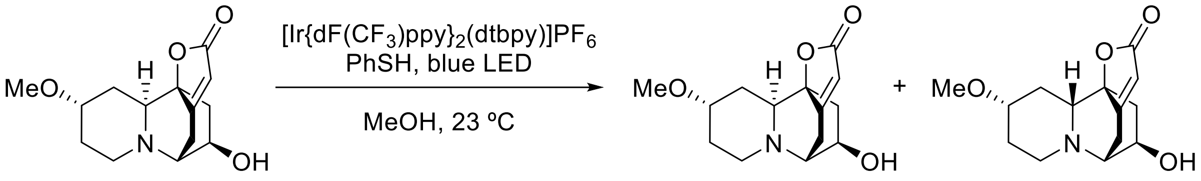

30 30 42

18% 63%

(–)-Azabicyclo[2.2.2]octane 42:

In a glovebox, $[Ir\{dF(CF_3)ppy\}_2(dtbpy)]PF_6$ (0.8 mg, 0.716 μmol, 0.01 equiv.), thiophenol (7.4 μL, 0.0716 mmol, 1.0 equiv.) were added to a solution of **30** (19 mg, 0.0716 mmol, 1.0 equiv.) in methanol (0.36 mL). The reaction mixture was stirred at 23 °C with the irradiation of kessil lamp (PR160L model, wavelength: 427 nm, 25% intensity of light). After 24 h, the resulting mixture was concentrated under reduced pressure. The resulting crude residue was purified by flash column chromatography (silica gel: diam. 1.5 cm, ht. 14 cm; eluent: ethyl acetate) to afford **42** (12 mg, 63%) as a white gum. The starting material **30** (3.5 mg, 18%) was also recovered.

^{1}H NMR (500 MHz, $CDCl_3$): δ 5.63 (t, *J* = 2.0 Hz, 1H), 4.20 (t, *J* = 7.1 Hz, 1H), 3.31 (s, 3H), 3.18 – 3.08 (m, 2H), 2.97 (dt, *J* = 5.0, 2.6 Hz, 1H), 2.88 (ddd, *J* = 11.2, 5.3, 2.5 Hz, 1H), 2.80 – 2.70 (m, 2H), 2.67 (td, *J* = 11.4, 2.7 Hz, 1H), 2.16 (app d, *J* = 10.7 Hz, 2H), 1.95 (ddq, *J* = 11.7, 4.3, 2.2 Hz, 1H), 1.91 (s, 1H), 1.47 (qd, *J* = 11.6, 5.3 Hz, 1H), 1.29 – 1.17 (m, 2H).

^{13}C NMR (101 MHz, $CDCl_3$): δ 176.1, 174.1, 109.3, 84.7, 78.2, 66.7, 59.7, 56.9, 56.0, 49.8, 36.5, 33.2, 31.4, 23.5.

HRMS (ESI): Calculated for $C_{14}H_{19}NO_4$ $[M+H]^+$: 266.1387, found: 266.1402

TLC (ethyl acetate) R*f*: 0.36 ($KMnO_4$).

$[\alpha]_D^{25}$: –56.2 (c 1.0, $CHCl_3$)

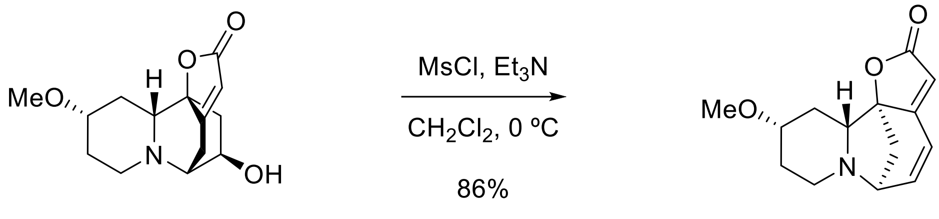

42 **4** 4-*epi*-phyllanthine

(–)-4-*epi*-Phyllanthine (4):

Triethylamine (28 μL, 0.204 mmol, 6.0 equiv.) and methanesulfonyl chloride (8 μL, 0.102 mmol, 3.0 equiv.) were added to a solution of **42** (9 mg, 0.0339 mmol, 1.0 equiv.) in dichloromethane (0.3 mL) at 0 °C. After 30 min, the reaction was quenched with saturated aqueous sodium bicarbonate solution (5 mL) and the layers were separated. The aqueous layer was extracted with ethyl acetate (3 × 10 mL) and the combined organic layer was dried over anhydrous sodium sulfate. The resulting filtrate was concentrated under reduced pressure. The resulting crude residue was purified by flash column chromatography (silica gel: diam. 1.5 cm, ht. 12 cm; eluent: acetone : hexanes = 1 : 2) to afford 4-*epi*-phyllanthine (**4**) (7.2 mg, 86%) as a yellow solid.

^{1}H NMR (500 MHz, $CDCl_3$): δ 6.57 (d, *J* = 9.3 Hz, 1H), 6.41 (dd, *J* = 9.2, 5.3 Hz, 1H), 5.55 (s, 1H), 3.79 (t, *J* = 4.6 Hz, 1H), 3.31 (s, 3H), 3.11 (tt, *J* = 10.2, 4.9 Hz, 1H), 2.98 (ddd, *J* = 10.9, 5.3, 3.0 Hz, 1H), 2.53 (dd, *J* = 9.4, 4.2 Hz, 1H), 2.41 (td, *J* = 10.9, 3.0 Hz, 1H), 2.12 (ddt, *J* = 11.3, 4.6, 1.7 Hz, 1H), 2.07 (dd, *J* = 11.8, 2.3 Hz, 1H), 1.99 – 1.89 (m, 1H), 1.79 (d, *J* = 9.5 Hz, 1H), 1.54 (dtd, *J* = 12.6, 10.7, 5.3 Hz, 1H), 1.43 (td, *J* = 11.6, 10.4 Hz, 1H).

^{13}C NMR (101 MHz, $CDCl_3$): δ 173.6, 169.9, 140.4, 121.7, 105.8, 89.4, 78.3, 60.1, 58.4, 56.0, 45.8, 42.6, 32.7, 32.7.

HRMS (ESI): Calculated for $C_{14}H_{17}NO_3$ $[M+H]^+$: 248.1281, found: 248.1294

TLC (acetone : hexanes = 1 : 1) R*f*: 0.47 (UV, $KMnO_4$).

$[\alpha]_D^{25}$: −734.7 (c 0.1, EtOH) [Lit. −753 (c 0.06, EtOH)][6]

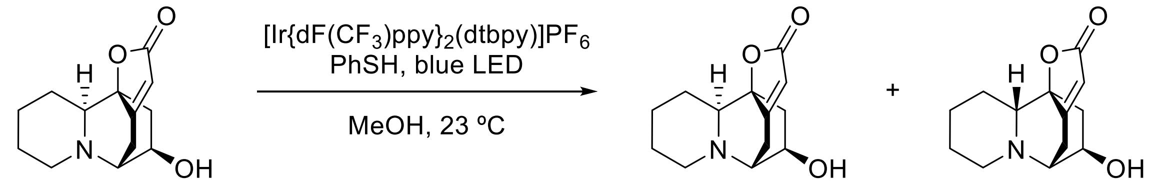

13 secu'amamine E

13 secu'amamine E 44%

44 *ent*-virosine B 38%

(–)-*ent*-Virosine B (44):

In a glovebox, [Ir{dF(CF_3)ppy}$_2$(dtbpy)]PF_6 (0.8 mg, 0.723 μmol, 0.01 equiv.), thiophenol (7.4 μL, 0.0723 mmol, 1.0 equiv.) were added to a solution of secu'amamine E (**13**) (16 mg, 0.0723 mmol, 1.0 equiv.) in methanol (0.36 mL). The reaction mixture was stirred at 23 °C with the irradiation of kessil lamp (PR160L model, wavelength: 427 nm, 25% intensity of light). After 12 h, the resulting mixture was concentrated under reduced pressure. The resulting crude residue was purified by flash column chromatography (silica gel: diam. 1.5 cm, ht. 14 cm; eluent: ethyl acetate : hexanes = 1.5 : 1) to afford *ent*-virosine B (**44**) (6 mg, 38%) as a white gum. The starting material **13** (7 mg, 44%) was also recovered.

^{1}H NMR (500 MHz, $CDCl_3$): δ 5.61 (s, 1H), 4.21 (dd, J = 8.6, 5.1 Hz, 1H), 3.10 (dt, J = 19.3, 2.3 Hz, 1H), 2.90 (dt, J = 5.2, 2.6 Hz, 1H), 2.82 – 2.73 (m, 3H), 2.65 (td, J = 10.4, 4.4 Hz, 1H), 2.21 (d, J = 10.5 Hz, 1H), 1.85 (dt, J = 13.3, 3.5 Hz, 1H), 1.76 (s, 1H), 1.64 (dd, J = 11.5, 3.2 Hz, 1H), 1.55 (td, J = 11.3, 9.9, 5.1 Hz, 2H), 1.35 (qd, J = 11.5, 10.9, 3.4 Hz, 1H), 1.27 (dtd, J = 17.3, 9.5, 8.7, 3.8 Hz, 1H), 1.19 (d, J = 13.2 Hz, 1H).

^{13}C NMR (126 MHz, $CDCl_3$): δ 176.6, 174.4, 109.0, 85.1, 66.9, 63.3, 57.7, 52.7, 36.6, 26.9, 25.9, 24.8, 23.2.

HRMS (ESI): Calculated for $C_{13}H_{17}NO_3$ $[M+Na]^+$: 258.1101, found: 258.1109

TLC (ethyl acetate : hexanes = 4 : 1) R*f*: 0.42 ($KMnO_4$).

$[\alpha]_D^{25}$: −73.1 (c 0.5, $CHCl_3$)

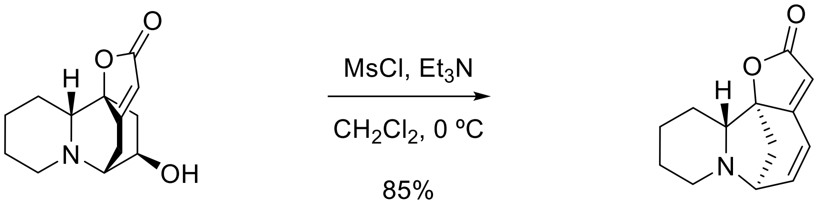

44 *ent*-virosine B **1** securinine

(–)-Securinine (1):

Triethylamine (25 µL, 0.179 mmol, 6.0 equiv.) and methanesulfonyl chloride (7 µL, 0.0893 mmol, 3.0 equiv.) were added to a solution of *ent*-virosine B (**44**) (7 mg, 0.0298 mmol, 1.0 equiv.) in dichloromethane (0.3 mL) at 0 °C. After 30 min, the reaction was quenched with saturated aqueous sodium bicarbonate solution (5 mL) and the layers were separated. The aqueous layer was extracted with ethyl acetate (3 × 10 mL) and the combined organic layer was dried over anhydrous sodium sulfate. The resulting filtrate was concentrated under reduced pressure. The resulting crude residue was purified by flash column chromatography (silica gel: diam. 1.5 cm, ht. 10 cm; eluent: acetone : hexanes = 1 : 2) to afford securinine (**1**) (5.5 mg, 85%) as a yellow solid.

^{1}H NMR (500 MHz, $CDCl_3$): δ 6.57 (d, *J* = 9.1 Hz, 1H), 6.38 (dd, *J* = 9.2, 5.3 Hz, 1H), 5.52 (s, 1H), 3.79 (t, *J* = 4.7 Hz, 1H), 2.94 (dt, *J* = 10.6, 3.8 Hz, 1H), 2.48 (dd, *J* = 9.3, 4.1 Hz, 1H), 2.39 (ddd, *J* = 10.3, 7.3, 5.0 Hz, 1H), 2.08 (dd, *J* = 11.3, 2.5 Hz, 1H), 1.86 (dt, *J* = 13.6, 3.6 Hz, 1H), 1.75 (d, *J* = 9.2 Hz, 1H), 1.62 (ddt, *J* = 11.8, 4.6, 2.4 Hz, 1H), 1.58 – 1.53 (m, 2H), 1.51 (app td, *J* = 12.4, 11.9, 3.8 Hz, 1H), 1.32 – 1.15 (m, 1H).

^{13}C NMR (101 MHz, $CDCl_3$): δ 173.9, 170.3, 140.4, 121.7, 105.3, 89.7, 63.2, 59.0, 49.0, 42.5, 27.5, 26.1, 24.7.

HRMS (ESI): Calculated for $C_{13}H_{15}NO_2$ $[M+H]^+$: 218.1176, found: 218.1176

TLC (acetone : hexanes = 1 : 2) R*f*: 0.40 (UV, $KMnO_4$).

$[\alpha]_D^{25}$: –982.3 (c 0.5, EtOH)

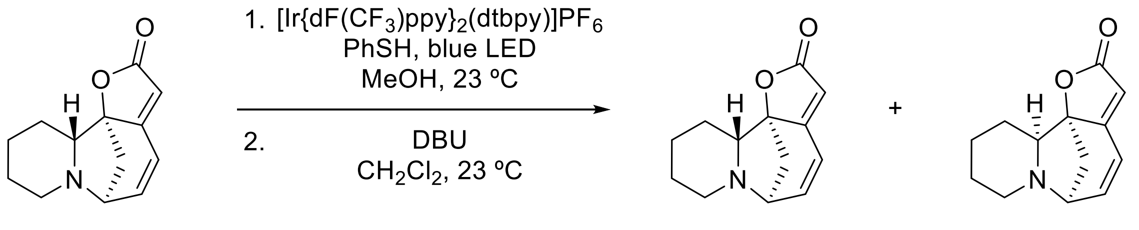

1 securinine

1 securinine
14%

2 allosecurinine
55%

(–)-Allosecurinine (2):

In a glovebox, [Ir{dF(CF_3)ppy}$_2$(dtbpy)]PF_6 (1.1 mg, 0.967 µmol, 0.01 equiv.), thiophenol (20 µL, 0.193 mmol, 2.0 equiv.) were added to a solution of securinine (**1**) (21 mg, 0.0967 mmol, 1.0 equiv.) in methanol (0.5 mL). The reaction mixture was stirred at 23 °C with the irradiation of kessil lamp (PR160L model, wavelength: 427 nm, 25% intensity of light). After 72 h, the reaction was quenched with saturated aqueous sodium thiosulfate solution (5 mL) and the layers were separated. The aqueous layer was extracted with ethyl acetate (3 × 10 mL) and the combined organic layer was dried over anhydrous sodium sulfate. The resulting filtrate was concentrated under reduced pressure.

The resulting crude residue was dissolved in dichloromethane (2 mL), and 1,8-diazabicyclo[5.4.0]undec-7-ene (72 µL, 0.483 mmol, 5.0 equiv.) was added. After 2 h, the resulting mixture was concentrated under reduced pressure. The resulting crude residue was purified by flash column chromatography (silica gel: diam. 1.5 cm, ht. 16 cm; eluent: acetone : hexanes = 1 : 4 to 1 : 1) to afford allosecurinine (**2**) (11.6 mg, 55% for 2 steps) as a yellow solid. The starting material **1** (3 mg, 14%) was also recovered.

^{1}H NMR (500 MHz, $CDCl_3$): δ 6.79 (dd, J = 9.1, 5.3 Hz, 1H), 6.62 (dd, J = 9.1, 1.1 Hz, 1H), 5.69 (s, 1H), 3.87 (t, J = 4.9 Hz, 1H), 3.63 (dd, J = 13.1, 3.4 Hz, 1H), 2.76 – 2.69 (m, 2H), 2.65 (dd, J = 9.8, 4.5 Hz, 1H), 1.89 (d, J = 9.7 Hz, 1H), 1.74 – 1.59 (m, 3H), 1.47 – 1.26 (m, 2H), 1.12 (qd, J = 12.9, 5.8 Hz, 1H).

^{13}C NMR (101 MHz, $CDCl_3$): δ 172.9, 167.7, 148.9, 122.8, 109.2, 91.9, 61.0, 59.0, 43.8, 42.9, 22.4, 21.3, 18.7.

HRMS (ESI): Calculated for $C_{13}H_{15}NO_2$ $[M+H]^+$: 218.1176, found: 218.1171

TLC (acetone : hexanes = 1 : 1) R*f*: 0.26 (UV, $KMnO_4$).

$[\alpha]_D^{25}$: –957.9 (c 1.0, EtOH)

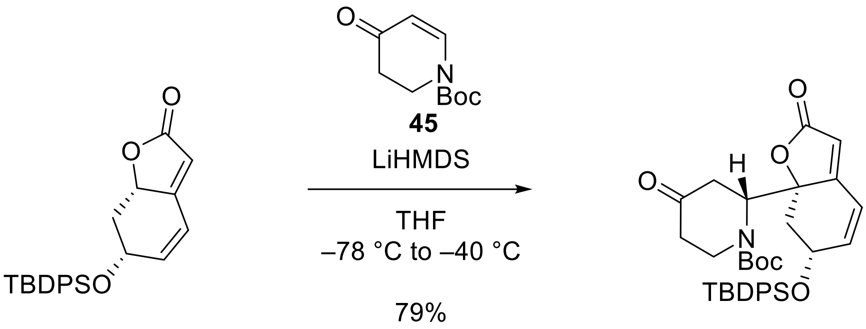

23 46

(+)-γ-Piperidone 46:

A solution of lithium bis(trimethylsilyl)amide (364 mg, 1.47 mmol, 1.2 equiv.) in tetrahydrofuran (1.5 mL) was added to a solution of **23** (480 mg, 1.23 mmol, 1.0 equiv.) in tetrahydrofuran (1.1 mL) at –78 °C under argon atmosphere. After 10 min, a solution of **45** (364 mg, 1.84 mmol, 1.5 equiv.) in tetrahydrofuran (1.5 mL) was added and the reaction mixture was heated to –40 °C. After 24 h, the reaction was quenched with saturated aqueous ammonium chloride solution (20 mL) and the layers were separated. The aqueous layer was extracted with ethyl acetate (3 × 40 mL) and the combined organic layer was dried over anhydrous sodium sulfate. The resulting filtrate was concentrated under reduced pressure. The resulting crude residue was purified by flash column chromatography (silica gel: diam. 4cm, ht. 16 cm; eluent: acetone : hexanes = 1 : 6) to **46** (570 mg, 79%) as a white solid.

^{1}H NMR (400 MHz, $CDCl_3$, major rotamer): δ 7.67 – 7.57 (m, 4H), 7.47 – 7.34 (m, 6H), 6.38 (dd, J = 10.2, 2.1 Hz, 1H), 6.03 (d, J = 10.1 Hz, 1H), 5.83 (s, 1H), 4.96 (t, J = 7.5 Hz, 1H), 4.63 (d, J = 7.1 Hz, 1H), 4.16 (dd, J = 14.2, 7.4 Hz, 1H), 3.47 – 3.35 (m, 1H), 2.62 (dd, J = 12.2, 5.8 Hz, 1H), 2.33 (app t, J = 15.6, 14.2 Hz, 1H), 2.29 – 2.16 (m, 1H), 2.12 – 1.93 (m, 2H), 1.73 (dd, J = 12.1, 9.4 Hz, 1H), 1.31 (s, 9H), 1.03 (s, 9H).

^{13}C NMR (101 MHz, $CDCl_3$, major rotamer): δ 204.7, 171.1, 164.7, 155.3, 143.3, 135.9, 135.9, 133.4 (2), 130.2 (2), 128.0, 128.0, 119.1, 113.4, 89.0, 81.6, 67.2, 54.9, 41.3, 40.5, 39.8, 38.7, 28.3, 27.0, 19.3.

HRMS (ESI): Calculated for $C_{34}H_{41}NO_6Si$ $[M+Na]^+$: 610.2595, found: 610.2633

TLC (acetone : hexanes = 1 : 4) R*f*: 0.23 (UV, $KMnO_4$).

$[\boldsymbol{\alpha}]_{\boldsymbol{D}}^{\mathbf{25}}$: 15.8 (c 1.0, $CHCl_3$)

Note: ^{1}H-NMR spectrum shows two sets of signals, due to the presence of two rotamers in 67:33 ratio. This assignation was corroborated with the same ^{1}H-NMR and EXSY experiments, where exchange signals between absorptions of the same proton but corresponding to different rotamers, were observed. This behavior could also be observed in the ^{13}C-NMR spectrum.

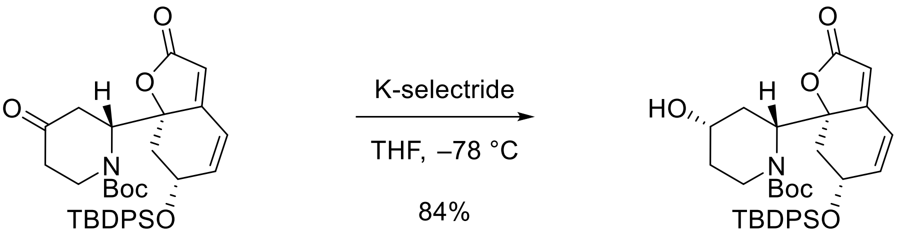

46 **47**

(+)-γ-Hydroxy piperidine 47:

K-selectride (1.0 M in tetrahydrofuran, 1.08 mL, 1.08 mmol, 1.2 equiv.) was added to a solution of **46** (530 mg, 0.902 mmol, 1.0 equiv.) in tetrahydrofuran (9 mL) at –78 °C under argon atmosphere. After 30 min, the reaction was quenched with saturated aqueous ammonium chloride solution (30 mL) and the layers were separated. The aqueous layer was extracted with ethyl acetate (3 × 50 mL) and the combined organic layer was dried over anhydrous sodium sulfate. The resulting filtrate was concentrated under reduced pressure. The resulting crude residue was purified by flash column chromatography (silica gel: diam. 4cm, ht. 12 cm; eluent: ethyl acetate : hexanes = 1 : 1) to **47** (446 mg, 84%) as a white solid.

^{1}H NMR (400 MHz, $CDCl_3$, major rotamer): δ 7.70 – 7.58 (m, 4H), 7.46 – 7.31 (m, 6H), 6.37 (d, *J* = 9.9 Hz, 1H), 5.97 (d, *J* = 10.0 Hz, 1H), 5.80 (s, 1H), 5.22 (br s, 1H), 4.14 (t, *J* = 7.6 Hz, 1H), 3.75 (dd, *J* = 14.3, 6.6 Hz, 1H), 3.67 (br s, 1H), 3.24 (t, *J* = 12.2 Hz, 1H), 2.86 (dd, *J* = 12.6, 6.3 Hz, 1H), 1.99 – 1.89 (m, 1H), 1.86 (br s, 1H), 1.74 (dd, *J* = 11.4, 9.7 Hz, 1H), 1.44 – 1.33 (m, 2H), 1.30 (s, 9H), 1.27 – 1.23 (m, 1H), 1.02 (s, 9H).

^{13}C NMR (101 MHz, $CDCl_3$, major rotamer): δ 172.7, 165.3, 156.5, 143.4, 136.0, 136.0, 133.8, 130.0, 127.9, 119.1, 112.5, 89.7, 80.5, 67.5, 64.2, 53.0, 41.5, 38.1, 32.4, 30.0, 28.3, 27.1, 19.3.

HRMS (ESI): Calculated for $C_{34}H_{43}NO_6Si$ $[M+Na]^+$: 612.2752, found: 612.2801

TLC (ethyl acetate : hexanes = 1 : 1) R*f*: 0.24 (UV, $KMnO_4$).

$[\alpha]_D^{25}$: 60.1 (c 1.0, $CHCl_3$)

Note: ^{1}H-NMR spectrum shows two sets of signals, due to the presence of two rotamers in 89:11 ratio. This assignation was corroborated with the same ^{1}H-NMR and EXSY experiments, where exchange signals between absorptions of the same proton but corresponding to different rotamers, were observed. This behavior could also be observed in the ^{13}C-NMR spectrum.

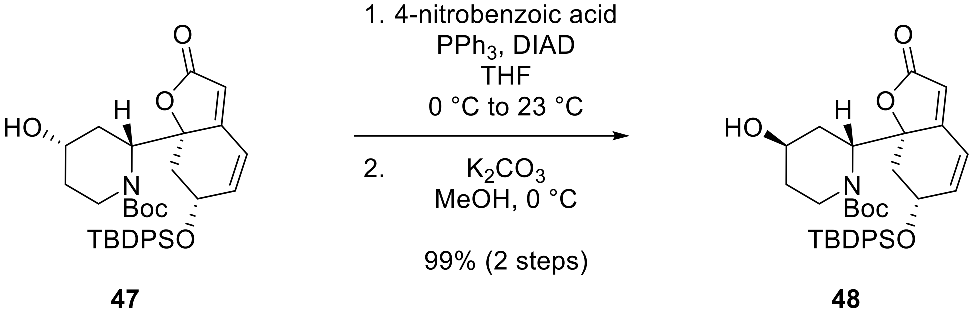

(+)-γ-Hydroxy piperidine 48:

4-Nitrobenzoic acid (233 mg, 1.40 mmol, 2.0 equiv.) and triphenylphosphine (275 mg, 1.05 mmol, 1.5 equiv.) were added to a solution of **47** (412 mg, 0.699 mmol, 1.0 equiv.) in tetrahydrofuran (7 mL) at 0 °C under argon atmosphere. Then, diisopropyl azodicarboxylate (275 μL, 1.40 mmol, 2.0 equiv.) solution in tetrahydrofuran (3.5 mL) was added dropwise and the reaction mixture was heated to 23 °C under light blocked condition by aluminium foil. After 3 h, the reaction was quenched with saturated aqueous sodium bicarbonate solution (20 mL) and the layers were separated. The aqueous layer was extracted with ethyl acetate (3 × 40 mL) and the combined organic layer was dried over anhydrous sodium sulfate. The resulting filtrate was concentrated under reduced pressure.

The resulting crude residue was dissolved in methanol (7 mL). Potassium carbonate (290 mg, 2.10 mmol, 3.0 equiv.) was added at 0 °C. After 30 min, the reaction was quenched with cold saturated aqueous ammonium chloride solution (30 mL) and the layers were separated. The aqueous layer was extracted with ethyl acetate (3 × 50 mL) and the combined organic layer was dried over anhydrous sodium sulfate. The resulting filtrate was concentrated under reduced pressure. The resulting crude residue was purified by flash column chromatography (silica gel: diam. 4 cm, ht. 10 cm; eluent: ethyl acetate : hexanes = 1 : 1) to afford **48** (407 mg, 99% for 2 steps) as a white solid.

^{1}H NMR (400 MHz, $CDCl_3$, major rotamer): δ 7.67 – 7.56 (m, 4H), 7.45 – 7.32 (m, 6H), 6.39 (dd, *J* = 10.2, 2.1 Hz, 1H), 6.03 (d, *J* = 10.2 Hz, 1H), 5.81 (s, 1H), 5.04 (app t, *J* = 7.2 Hz, 1H), 4.39 (d, *J* = 7.5 Hz, 1H), 4.03 (br s, 1H), 3.90 (d, *J* = 13.7 Hz, 1H), 3.04 (app t, *J* = 13.1 Hz, 1H), 2.66 (dd, *J* = 12.2, 6.2 Hz, 1H), 1.89 – 1.80 (m, 1H), 1.71 – 1.61 (m, 2H), 1.51 (dd, *J* = 13.9, 5.5 Hz, 1H), 1.28 (s, 9H), 1.24 – 1.09 (m, 2H), 1.02 (s, 9H).

^{13}C NMR (101 MHz, $CDCl_3$, major rotamer): δ 172.4, 166.4, 155.8, 143.5, 135.9, 134.1, 133.6, 130.1, 130.1, 127.9, 127.9, 119.2, 112.4, 90.6, 80.7, 67.3, 65.5, 53.6, 41.8, 40.3, 34.6, 32.3, 28.3, 27.1, 27.0, 19.3.

HRMS (ESI): Calculated for $C_{34}H_{43}NO_6Si$ $[M+Na]^+$: 612.2752, found: 612.2745

TLC (ethyl acetate : hexanes = 1 : 1) R*f*: 0.24 (UV, $KMnO_4$).

$[\boldsymbol{\alpha}]_{\boldsymbol{D}}^{\mathbf{25}}$: 53.8 (c 1.0, $CHCl_3$)

Note: ^{1}H-NMR spectrum shows two sets of signals, due to the presence of two rotamers in 75:25 ratio. This assignation was corroborated with the same ^{1}H-NMR and EXSY experiments,

where exchange signals between absorptions of the same proton but corresponding to different rotamers, were observed. This behavior could also be observed in the ^{13}C-NMR spectrum.

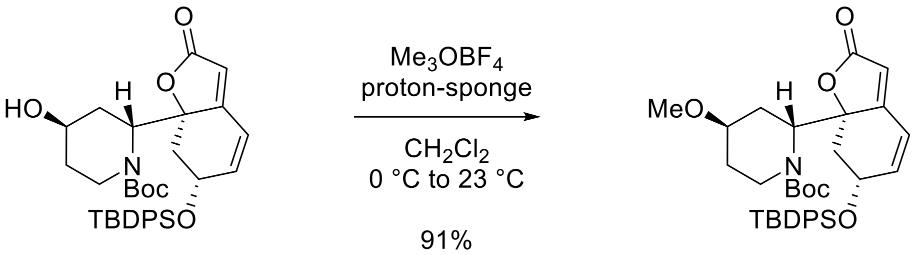

48 **S6**

(+)-γ-Methoxy piperidine S6:

Proton-sponge (592 mg, 2.76 mmol, 4.0 equiv.) and trimethyloxonium tetrafluoroborate (306 mg, 2.07 mmol, 3.0 equiv.) were added to a solution of **48** (407 mg, 0.690 mmol, 1.0 equiv.) in dichloromethane (7 mL) at 0 °C under argon atmosphere and the reaction mixture was slowly heated to 23 °C. After 4 h, the resulting mixture was diluted with ethyl acetate (100 mL) and filtered through a pad of celite. The resulting filtrate was washed with 10% aqueous citric acid solution (50 mL) and brine (50 mL), and the resulting mixture was dried over anhydrous sodium sulfate. The resulting filtrate was concentrated under reduced pressure. The resulting crude residue was purified by flash column chromatography (silica gel: diam. 4 cm, ht. 13 cm; eluent: acetone : hexanes = 1 : 3) to afford **S6** (380 mg, 91%) as a white solid.

^{1}H NMR (400 MHz, $CDCl_3$, major rotamer): δ 7.65 – 7.55 (m, 4H), 7.46 – 7.32 (m, 6H), 6.39 (dd, J = 10.0, 2.2 Hz, 1H), 6.03 (d, J = 10.0 Hz, 1H), 5.82 (s, 1H), 5.03 (ddd, J = 8.6, 5.8, 2.6 Hz, 1H), 4.38 (d, J = 7.2 Hz, 1H), 3.91 (dt, J = 14.2, 2.3 Hz, 1H), 3.55 (tt, J = 10.5, 4.9 Hz, 1H), 3.22 (s, 3H), 3.02 (td, J = 13.7, 2.4 Hz, 1H), 2.65 (dd, J = 12.1, 5.9 Hz, 1H), 1.98 – 1.89 (m, 1H), 1.66 (dd, J = 12.1, 9.1 Hz, 1H), 1.58 (dd, J = 13.6, 4.7 Hz, 1H), 1.27 (s, 9H), 1.17 – 1.05 (m, 2H), 1.02 (s, 9H).

^{13}C NMR (101 MHz, $CDCl_3$, major rotamer): δ 172.3, 166.4, 155.8, 143.6, 135.9 (2), 134.1, 133.6, 130.1, 130.1, 128.0, 127.9, 119.1, 112.4, 90.5, 80.7, 74.4, 67.3, 55.9, 53.5, 41.8, 40.3, 31.4, 29.1, 28.3, 27.0, 19.3.

HRMS (ESI): Calculated for $C_{35}H_{45}NO_6Si$ $[M+Na]^+$: 626.2908, found: 626.2944

TLC (ethyl acetate : hexanes = 1 : 2) R*f*: 0.45 (UV, $KMnO_4$).

$[\boldsymbol{\alpha}]_{\boldsymbol{D}}^{\mathbf{25}}$: 48.4 (c 1.0, $CHCl_3$)

Note: ^{1}H-NMR spectrum shows two sets of signals, due to the presence of two rotamers in 75:25 ratio. This assignation was corroborated with the same ^{1}H-NMR and EXSY experiments, where exchange signals between absorptions of the same proton but corresponding to different rotamers, were observed. This behavior could also be observed in the ^{13}C-NMR spectrum.

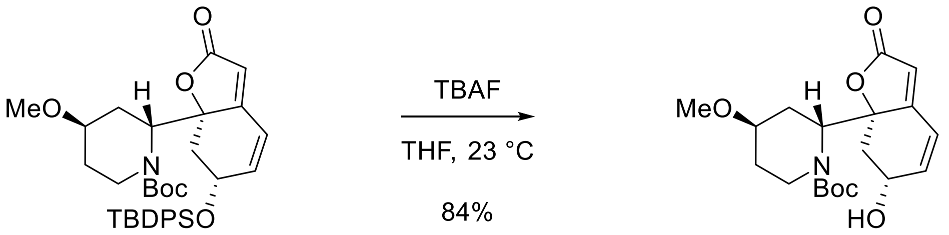

S6 49

(+)-ε-Hydroxy butenolide 49:

Tetra-*n*-butylammonium fluoride (1.0 M in tetrahydrofuran, 576 μL, 0.576 mmol, 1.0 equiv.) was added to a solution of **S6** (348 mg, 0.576 mmol, 1.0 equiv.) in tetrahydrofuran (6 mL) under argon atmosphere. After 1 h, the reaction was quenched with saturated aqueous ammonium chloride solution (20 mL) and the layers were separated. The aqueous layer was extracted with ethyl acetate (3 × 40 mL) and the combined organic layer was dried over anhydrous sodium sulfate. The resulting filtrate was concentrated under reduced pressure. The resulting crude residue was purified by flash column chromatography (silica gel: diam. 4cm, ht. 13 cm; eluent: ethyl acetate : hexanes = 3 : 2) to **49** (179 mg, 84%) as a white solid.

^{1}H NMR (400 MHz, $CDCl_3$, major rotamer): δ 6.54 (dd, *J* = 10.0, 2.3 Hz, 1H), 6.27 (dt, *J* = 10.0, 1.6 Hz, 1H), 5.89 (s, 1H), 4.97 (dt, *J* = 9.5, 6.4 Hz, 1H), 4.51 (d, *J* = 7.2 Hz, 1H), 4.04 (dt, *J* = 13.8, 2.4, 2.0 Hz, 1H), 3.58 (tt, *J* = 10.3, 4.9 Hz, 1H), 3.25 (s, 3H), 3.19 (dd, *J* = 13.6, 2.7 Hz, 1H), 2.86 (dd, *J* = 12.0, 5.9 Hz, 1H), 2.14 (d, *J* = 7.2 Hz, 1H), 1.98 (ddd, *J* = 12.7, 5.0, 2.3 Hz, 1H), 1.71 – 1.63 (m, 1H), 1.58 (dd, *J* = 12.1, 9.5 Hz, 1H), 1.45 (s, 9H), 1.32 – 1.10 (m, 2H).

^{13}C NMR (101 MHz, $CDCl_3$, major rotamer): δ 172.2, 166.1, 156.0, 143.2, 119.8, 112.9, 90.7, 81.0, 74.3, 66.0, 56.0, 53.3, 41.7, 40.3, 31.3, 29.1, 28.5.

HRMS (ESI): Calculated for $C_{19}H_{27}NO_6$ $[M+Na]^+$: 388.1731, found: 388.1771

TLC (ethyl acetate : hexanes = 2 : 1) R*f*: 0.33 (UV, $KMnO_4$).

$[\boldsymbol{\alpha}]_{\boldsymbol{D}}^{\mathbf{25}}$: 76.5 (c 1.0, $CHCl_3$)

Note: ^{1}H-NMR spectrum shows two sets of signals, due to the presence of two rotamers in 81:19 ratio. This assignation was corroborated with the same ^{1}H-NMR and EXSY experiments, where exchange signals between absorptions of the same proton but corresponding to different rotamers, were observed. This behavior could also be observed in the ^{13}C-NMR spectrum.

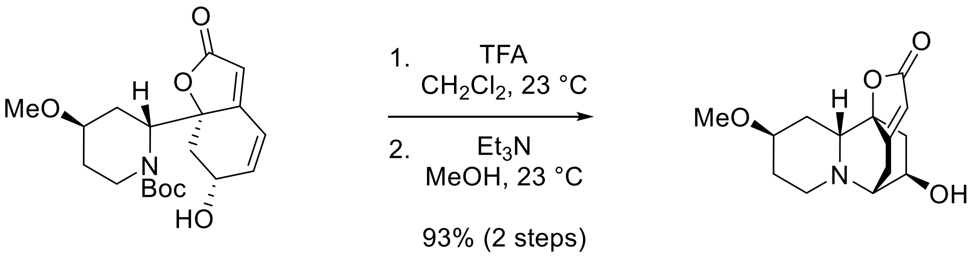

49 50

(–)-Azabicyclo[2.2.2]octane 50:

Trifluoroacetic acid (4.4 mL) was added to a solution of **49** (162 mg, 0.443 mmol, 1.0 equiv.) in dichloromethane (4.4 mL). After 30 min, the resulting mixture was concentrated via air blowing. The resulting crude residue was dissolved in methanol (3 mL). Triethylamine (1.5 mL) was added and the mixture was heated to 50 °C. After 2.5 h, the resulting mixture was concentrated under reduced pressure. The resulting crude residue was purified by flash column chromatography (silica gel: diam. 2.5 cm, ht. 14 cm; eluent: acetone : hexanes = 1 : 2) to afford **50** (109 mg, 93% for 2 steps) as a white gum.

^{1}H NMR (400 MHz, $CDCl_3$): δ 5.61 (t, *J* = 2.0 Hz, 1H), 4.21 (t, *J* = 6.8 Hz, 1H), 3.61 (p, *J* = 3.0 Hz, 1H), 3.25 (s, 3H), 3.10 (dt, *J* = 19.1, 2.1 Hz, 1H), 2.97 – 2.86 (m, 2H), 2.84 – 2.71 (m, 2H), 2.65 (d, *J* = 11.5 Hz, 1H), 2.59 (ddd, *J* = 10.8, 5.4, 2.3 Hz, 1H), 1.93 (ddd, *J* = 12.9, 4.7, 2.3 Hz, 1H), 1.82 (dt, *J* = 13.7, 2.5 Hz, 1H), 1.76 (br s, 1H), 1.70 (dddd, *J* = 13.6, 11.8, 5.4, 3.3 Hz, 1H), 1.47 (ddd, *J* = 12.8, 11.7, 2.8 Hz, 1H), 1.20 (d, *J* = 13.3 Hz, 1H).

^{13}C NMR (101 MHz, $CDCl_3$): δ 176.3, 174.3, 109.1, 84.4, 74.5, 66.9, 57.5, 56.3, 56.2, 47.7, 36.5, 31.4, 29.6, 23.3.

HRMS (ESI): Calculated for $C_{14}H_{19}NO_4$ $[M+H]^+$: 266.1387, found: 266.1402

TLC (acetone : hexanes = 1 : 1) R*f*: 0.58 ($KMnO_4$).

$[\alpha]_D^{25}$: –45.0 (c 1.0, $CHCl_3$)

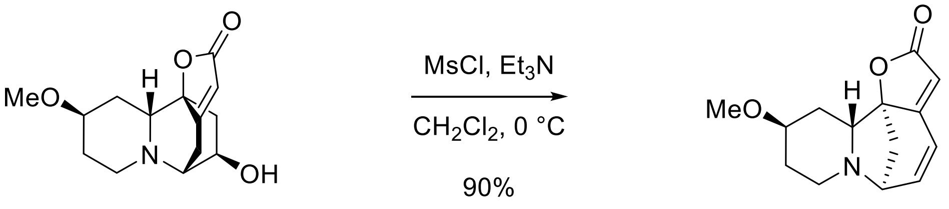

50 **5** phyllanthine

(–)-Phyllanthine (5):

Triethylamine (98 µL, 0.701 mmol, 6.0 equiv.) and methanesulfonyl chloride (27 µL, 0.350 mmol, 3.0 equiv.) were added to a solution of **50** (31 mg, 0.117 mmol, 1.0 equiv.) in dichloromethane (1.2 mL) at 0 °C. After 30 min, the reaction was quenched with saturated aqueous sodium bicarbonate solution (10 mL) and the layers were separated. The aqueous layer was extracted with ethyl acetate (3 × 20 mL) and the combined organic layer was dried over anhydrous sodium sulfate. The resulting filtrate was concentrated under reduced pressure. The resulting crude residue was purified by flash column chromatography (silica gel: diam. 1.5 cm, ht. 12 cm; eluent: acetone : hexanes = 1 : 2) to afford phyllanthine (**5**) (26 mg, 90%) as a yellow solid.

^{1}H NMR (400 MHz, $CDCl_3$): δ 6.56 (d, *J* = 9.1 Hz, 1H), 6.40 (dd, *J* = 9.2, 5.3 Hz, 1H), 5.52 (s, 1H), 3.77 (dd, *J* = 5.3, 4.2 Hz, 1H), 3.60 (p, *J* = 3.0 Hz, 1H), 3.24 (s, 3H), 2.76 (ddd, *J* = 10.6, 5.3, 3.2 Hz, 1H), 2.64 (td, *J* = 10.5, 3.5 Hz, 1H), 2.56 (dd, *J* = 12.3, 2.5 Hz, 1H), 2.49 (dd, *J* = 9.3, 4.2 Hz, 1H), 1.92 (dtd, *J* = 13.1, 2.7, 1.6 Hz, 1H), 1.84 – 1.69 (m, 2H), 1.76 (d, *J* = 9.5 Hz, 2H), 1.65 (ddd, *J* = 13.1, 12.2, 2.9 Hz, 1H).

^{13}C NMR (101 MHz, $CDCl_3$): δ 173.8, 170.3, 140.5, 121.8, 105.6, 89.5, 74.5, 59.0, 56.5, 56.3, 44.8, 42.2, 31.3, 30.9.

HRMS (ESI): Calculated for $C_{14}H_{17}NO_3$ $[M+H]^+$: 248.1281, found: 248.1293

TLC (acetone : hexanes = 1 : 2) R*f*: 0.32 (UV, $KMnO_4$).

$[\alpha]_D^{25}$: –890.2 (c 1.0, $CHCl_3$) [Lit. –898 (c 0.98, $CHCl_3$)][6]

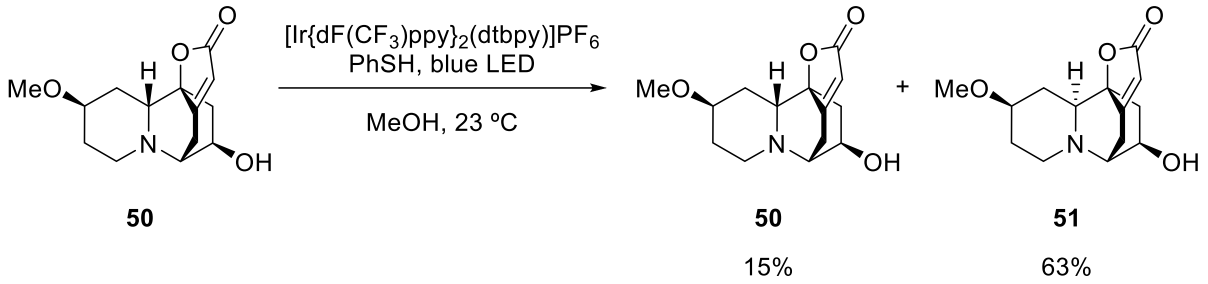

(–)-Azabicyclo[2.2.2]octane 51:

In a glovebox, [Ir{dF(CF_3)ppy}$_2$(dtbpy)]PF_6 (0.4 mg, 0.377 μmol, 0.01 equiv.), thiophenol (3.9 μL, 0.0377 mmol, 1.0 equiv.) were added to a solution of **50** (10 mg, 0.0377 mmol, 1.0 equiv.) in methanol (0.2 mL). The reaction mixture was stirred at 23 °C with the irradiation of kessil lamp (PR160L model, wavelength: 427 nm, 25% intensity of light). After 12 h, the resulting mixture was concentrated under reduced pressure. The resulting crude residue was purified by flash column chromatography (silica gel: diam. 1.5 cm, ht. 14 cm; eluent: ethyl acetate) to afford **51** (6.3 mg, 63%) as a white gum. The starting material **50** (1.5 mg, 15%) was also recovered.

^{1}H NMR (400 MHz, $CDCl_3$): δ 5.71 (t, J = 1.9 Hz, 1H), 4.32 (dt, J = 8.9, 4.0 Hz, 1H), 3.28 (s, 3H), 3.17 (tt, J = 10.3, 4.9 Hz, 1H), 3.04 – 2.91 (m, 3H), 2.81 – 2.70 (m, 3H), 2.67 (dd, J = 12.7, 9.6 Hz, 1H), 2.08 – 1.99 (m, 1H), 1.97 – 1.87 (m, 2H), 1.47 (dd, J = 12.6, 4.4 Hz, 1H), 1.43 – 1.34 (m, 1H), 0.73 (q, J = 11.1 Hz, 1H).

^{13}C NMR (101 MHz, $CDCl_3$): δ 173.8, 173.4, 112.1, 84.2, 77.8, 65.4, 61.3, 58.3, 55.9, 49.9, 41.0, 33.1, 31.3, 29.2.

HRMS (ESI): Calculated for $C_{14}H_{19}NO_4$ $[M+H]^+$: 266.1387, found: 266.1402

TLC (ethyl acetate) R*f*: 0.36 ($KMnO_4$).

$[\alpha]_D^{25}$: –56.2 (c 1.0, $CHCl_3$)

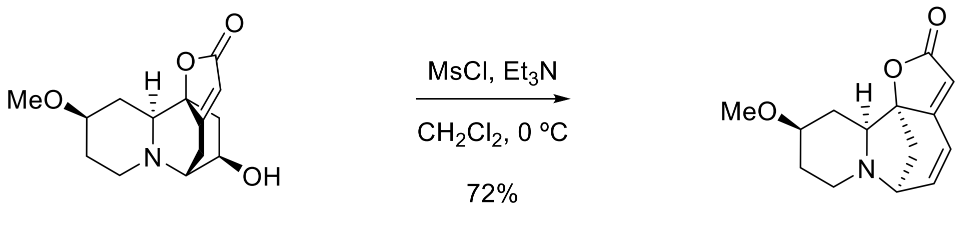

51 **52** 4-*epi*-securitinine

(–)-4-*epi*-Securitinine (52):

Triethylamine (50 µL, 0.362 mmol, 6.0 equiv.) and methanesulfonyl chloride (14 µL, 0.181 mmol, 3.0 equiv.) were added to a solution of **51** (16 mg, 0.0603 mmol, 1.0 equiv.) in dichloromethane (0.6 mL) at 0 °C. After 30 min, the reaction was quenched with saturated aqueous sodium bicarbonate solution (50 mL) and the layers were separated. The aqueous layer was extracted with ethyl acetate (3 × 10 mL) and the combined organic layer was dried over anhydrous sodium sulfate. The resulting filtrate was concentrated under reduced pressure. The resulting crude residue was purified by flash column chromatography (silica gel: diam. 1.5 cm, ht. 12 cm; eluent: acetone : hexanes = 1 : 1) to afford 4-*epi*-securitinine (**52**) (11 mg, 74%) as a yellow solid.

^{1}H NMR (400 MHz, $CDCl_3$): δ 6.80 (dd, *J* = 9.1, 5.3 Hz, 1H), 6.64 (dd, *J* = 9.0, 1.1 Hz, 1H), 5.73 (s, 1H), 3.87 (t, *J* = 4.9 Hz, 1H), 3.60 (dd, *J* = 13.8, 3.4 Hz, 1H), 3.45 (ddt, *J* = 10.8, 7.3, 3.4 Hz, 1H), 3.24 (s, 3H), 2.90 (td, *J* = 11.4, 3.1 Hz, 1H), 2.75 (dt, *J* = 10.8, 4.2 Hz, 1H), 2.68 (dd, *J* = 9.9, 4.4 Hz, 1H), 2.01 – 1.90 (m, 1H), 1.95 (d, *J* = 10.0 Hz, 1H), 1.79 (ddd, *J* = 12.6, 6.4, 3.4 Hz, 1H), 1.69 (dq, *J* = 14.6, 3.2 Hz, 1H), 1.08 (ddd, *J* = 13.8, 12.5, 10.3 Hz, 1H).

^{13}C NMR (101 MHz, $CDCl_3$): δ 172.6, 167.4, 148.5, 123.1, 109.5, 91.4, 74.2, 58.8, 58.2, 56.2, 43.3, 42.1, 30.8, 28.3.

HRMS (ESI): Calculated for $C_{14}H_{17}NO_3$ $[M+H]^+$: 248.1281, found: 248.1295

TLC (acetone : hexanes = 1 : 1) R*f*: 0.23 (UV, $KMnO_4$).

$[\alpha]_D^{25}$: –972.2 (c 1.0, $CHCl_3$)

Supplementary Discussion

3. Determination of Enantiomeric Excess (% ee)

Chiral HPLC spectra of (–)-Nitrobenzoic ester S1:

HPLC conditions:

Column: Daicel Chiralpak® AS-H, 4.6 mm x 250 mm

Flow rate: 0.6 mL/min

Eluent: 0–10 min: 100% hexanes

10–70 min: 100% hexanes → 99% hexanes, 1% iPrOH

Injection volume: 2 µL

Monitored at λ: 254.16 nm

Supplementary Figure 1. HPLC trace for the determination of enantiomeric excess of **S1**.

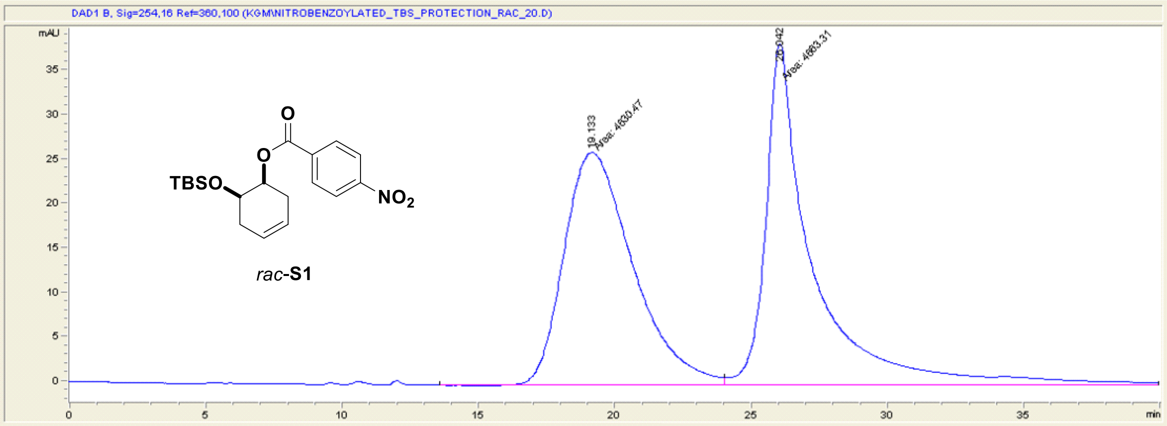

#	Time	Area	Height	Width	Area%	Symmetry
1	19.133	4630.5	26.2	2.9461	49.823	0.652
2	26.042	4663.3	38.4	2.0255	50.177	0.42

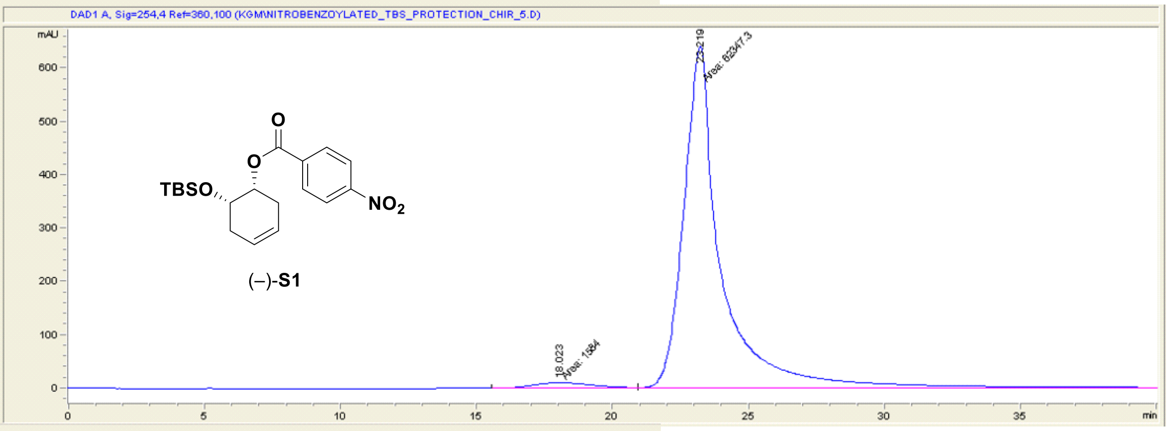

#	Time	Area	Height	Width	Area%	Symmetry
1	18.023	1584	10.3	2.5581	2.478	0
2	23.219	62347.3	641.5	1.6199	97.522	0.729

Chiral HPLC spectra of (–)-*O*-TBDPS-menisdaurilide 23:

HPLC conditions:

Column: Daicel Chiralpak® AS-H, 4.6 mm x 250 mm

Flow rate: 1 mL/min

Eluent: 97% hexanes, 3% iPrOH

Injection volume: 5 µL

Monitored at λ: 254.16 nm

Supplementary Figure 2. HPLC trace for the determination of enantiomeric excess of **23**.

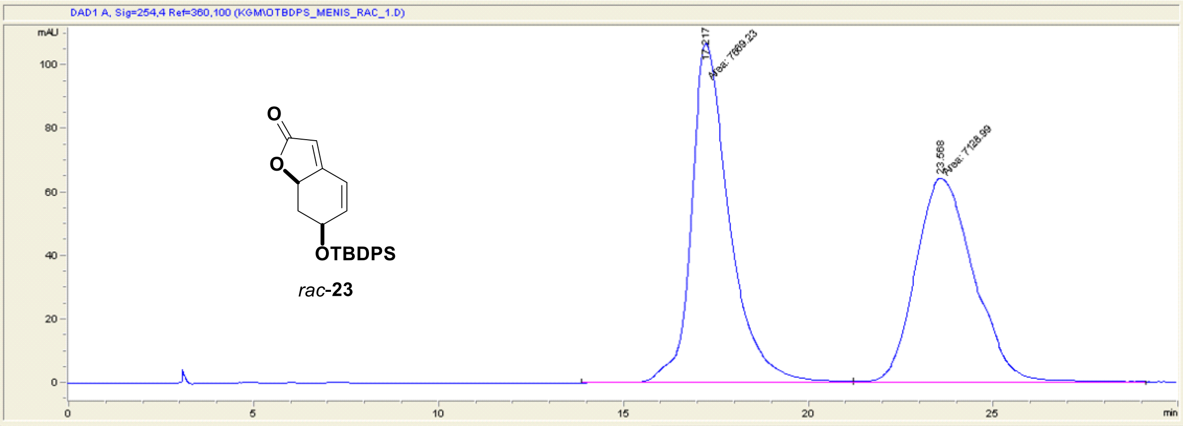

#	Time	Area	Height	Width	Area%	Symmetry
1	17.217	7669.2	106.7	1.1984	51.825	0
2	23.568	7129	64.3	1.8472	48.175	0.739

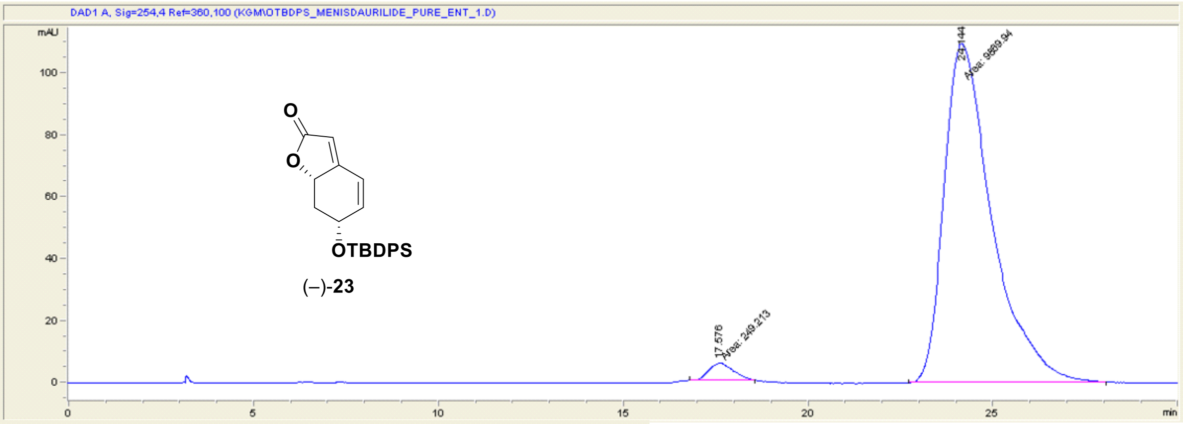

#	Time	Area	Height	Width	Area%	Symmetry
1	17.576	249.2	5.5	0.7566	2.463	0.702
2	24.144	9869.9	109.5	1.5029	97.537	0.575

Chiral ^{1}H NMR with the Co complex of (–)-β-Methoxy lactam 27:

Supplementary Figure 3. Chiral ^{1}H NMR analysis of **27** with [Co-L1-S1]BArF in $CDCl_3$.[7]

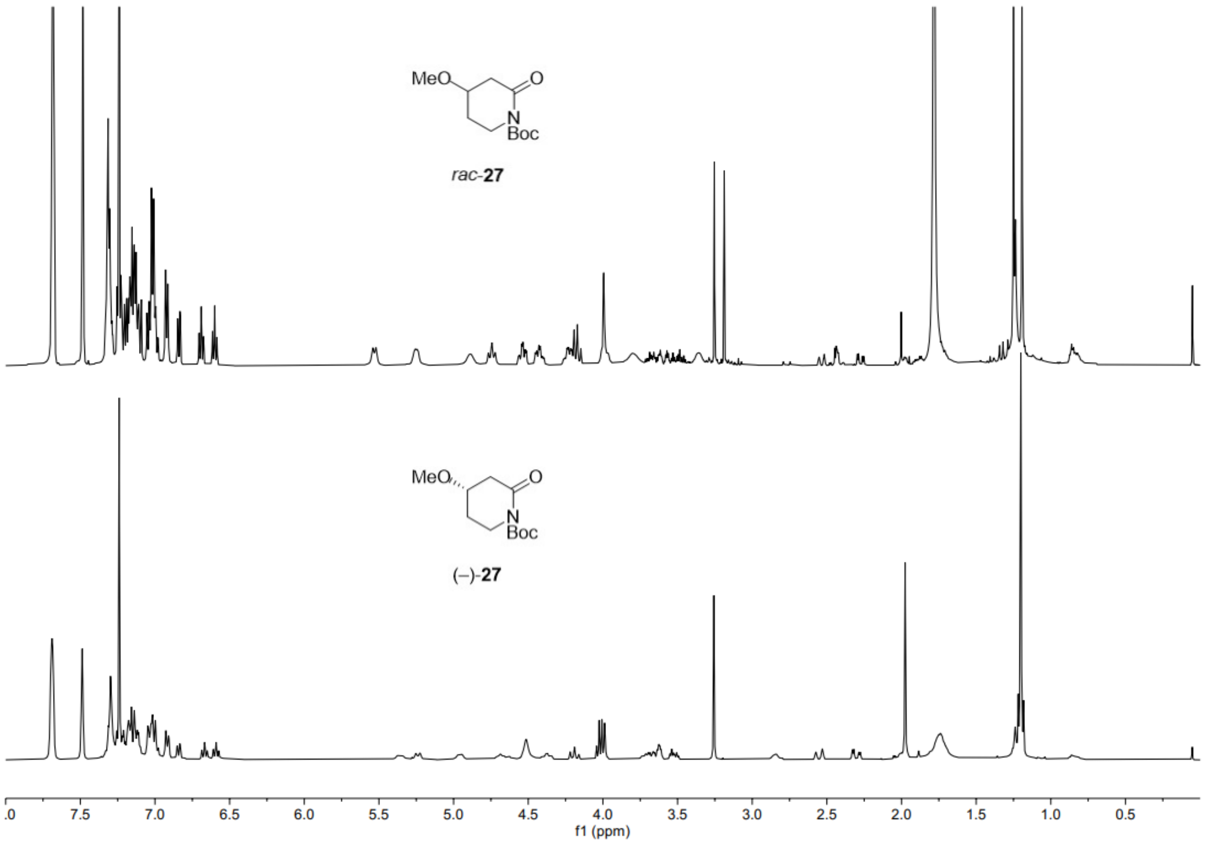

Supplementary Figure 4. Selected region for the determination of enantiomeric excess of **27**.

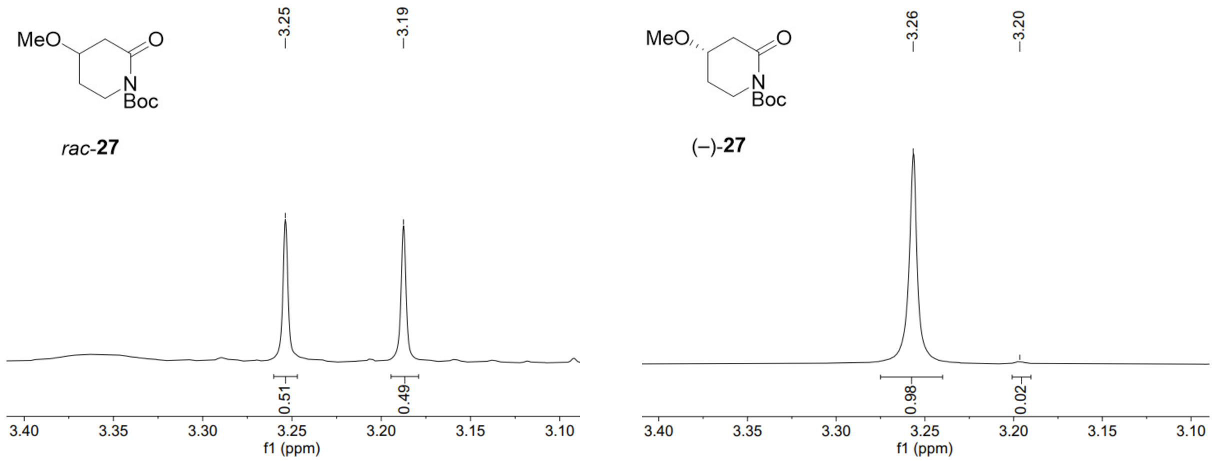

Chiral ^{1}H NMR with the Co complex of (–)-α,β-Epoxy lactam 36:

Supplementary Figure 5. Chiral ^{1}H NMR analysis of **36** with [Co-L1-S1]BArF in CD_2Cl_2.[7]

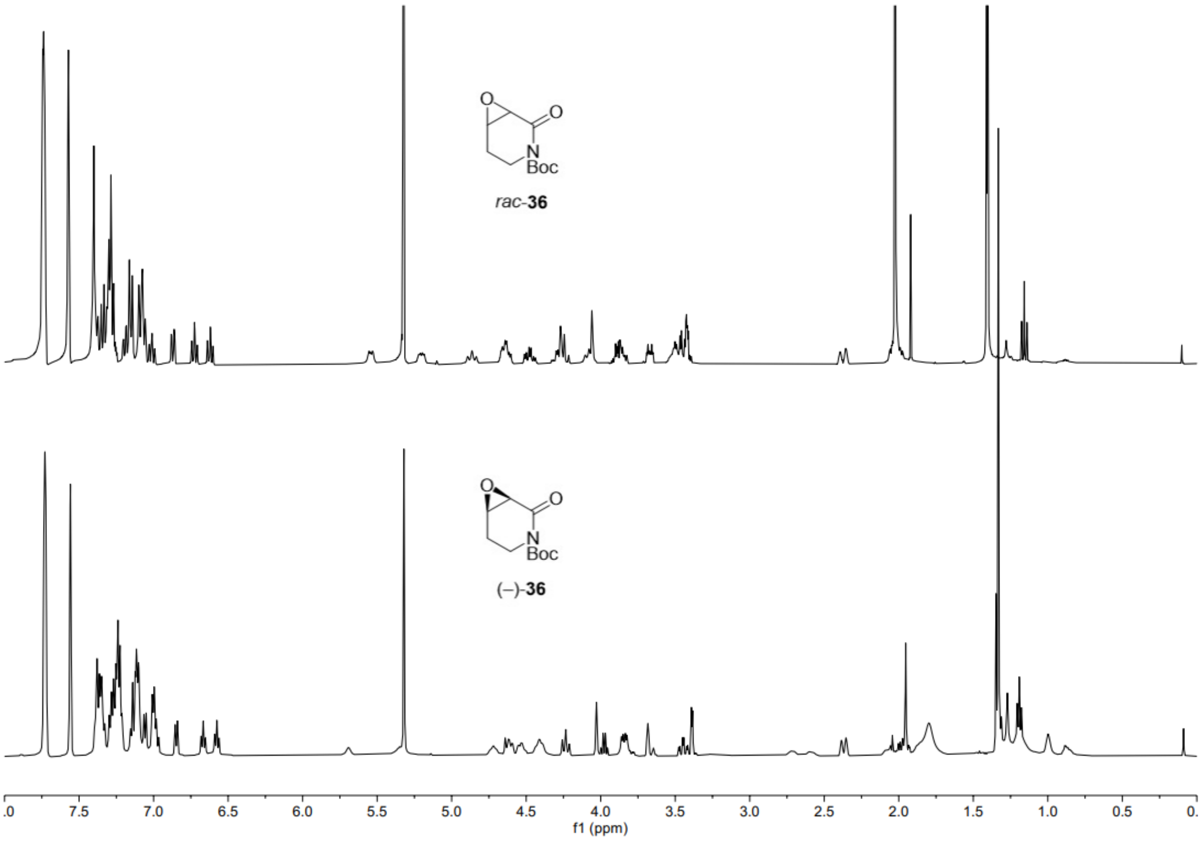

Supplementary Figure 6. Selected region for the determination of enantiomeric excess of **36**.

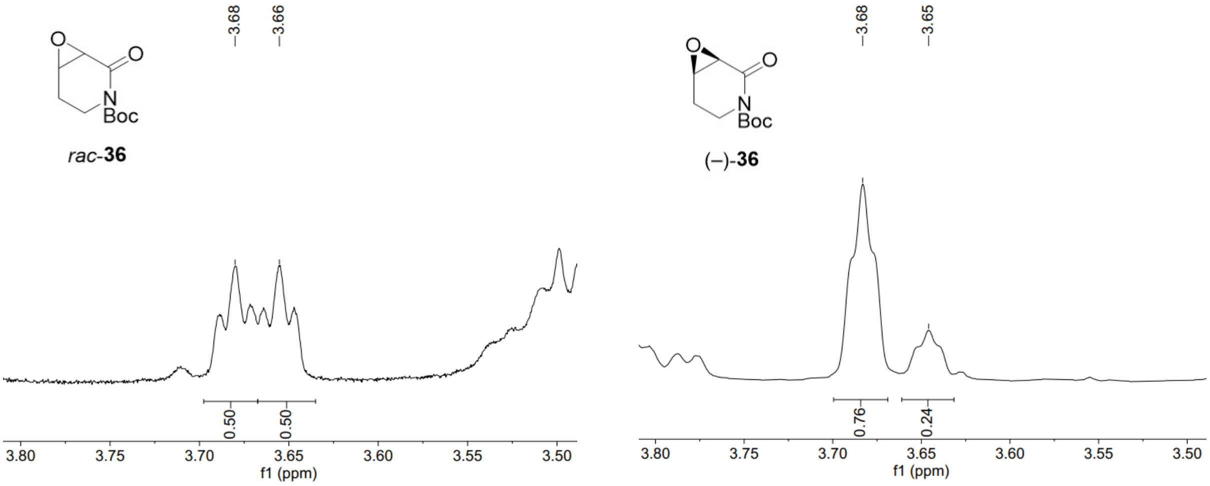

4. Comparison of Spectral Data of Synthetic Natural Products with Other Reports

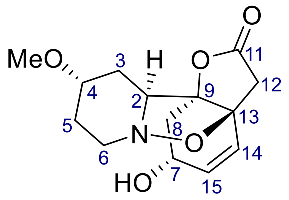

7b securingine A

Supplementary Table 1. Comparison of ^{1}H-NMR spectroscopic data of natural and synthetic securingine A (**7**)

position	isolation report[3]<br>δ_1 (ppm ; multi, *J* in Hz)	this work<br>δ_2 (ppm ; multi, *J* in Hz)[a]	deviation<br>$\Delta\delta = \delta_1 - \delta_2$ (ppm)
2	2.51 (d, 13.1)	2.46 (dd, 12.2, 2.6)	0.05
3	a 2.12 (dd, 13.8, 2.7)	a 2.07 (dd, 13.8, 2.7)	0.05
	b 1.77 (m)	b 1.72 (m)	0.05
4	3.59 (m)	3.55 (m)	0.04
5	a 2.07 (m)	a 2.02 (m)	0.05
	b 1.73 (m)	b 1.68 (m)	0.05
6	a 3.22 (ddd, 8.9, 4.3, 2.6)	a 3.17 (ddd, 9.0, 4.3, 2.6)	0.05
	b 2.75 (overlap)	b 2.70 (overlap)	0.05
7	4.43 (br s)	4.38 (br s)	0.05
8	2.42 (dd, 13.5, 4.6)	2.37 (dd, 13.5, 4.7)	0.05
	1.88 (dd, 13.5, 9.1)	1.83 (dd, 13.5, 9.1)	0.05
12	3.06 (d, 18.7)	3.01 (d, 18.7z)	0.05
	2.76 (d ,18.7)	2.72 (d, 18.7)	0.04
14	5.93 (dd, 10.1, 1.7)	5.88 (dd, 10.1, 1.8)	0.05
15	6.11 (dd, 10.1, 2.6)	6.07 (dd, 10.2, 2.7)	0.04
OMe-4	3.34 (s)	3.29 (s)	0.05
OH-7		1.96 (d)	

[a] The chemical shift were recorded with respect to the deuterated solvent shift ($CHCl_3$, δ 7.24 ppm for the proton).

Supplementary Table 2. Comparison of ^{13}C NMR spectroscopic data of natural and synthetic securingine A (**7**)

position	isolation report[3] δ_1 (ppm)	this work δ_2 (ppm)[a]	deviation $\Delta\delta = \delta_1 - \delta_2$ (ppm)
2	67.1	67.1	0
3	28.9	28.9	0
4	72.2	72.3	–0.1
5	28.1	28.1	0
6	49.5	49.5	0
7	64.2	64.2	0
8	37.6	37.5	0.1
9	93.5	93.6	–0.1
11	174.4	174.6	–0.2
12	42.2	42.3	–0.1
13	80.9	81.0	–0.1
14	126.9	126.9	0
15	134.6	134.7	–0.1
OMe-4	56.0	56.1	–0.1

[a] The chemical shift were recorded with respect to the deuterated solvent shift ($CDCl_3$, δ 77.23 ppm for the carbon).

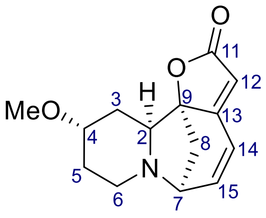

3 securitinine

Supplementary Table 3. Comparison of ^{1}H-NMR spectroscopic data of natural and synthetic securitinine (**3**)

position	isolation report[6] δ_1 (ppm ; multi, *J* in Hz)	this work δ_2 (ppm ; multi, *J* in Hz)[a]	deviation $\Delta\delta = \delta_1 - \delta_2$ (ppm)
2	3.90 (dd, 13.5, 3.5)	3.87 (dd, 13.6, 3.4)	0.03
3	a 1.57 – 1.68 (m)	a 1.68 – 1.52 (m)	
	b 1.20 (dt, 14.0, 13.5, 4.4)	1.17 (dt, 13.7, 4.7)	0.03
4	3.65 (dddd, 8.8, 6.0, 4.4, 1.7)	3.62 (dddd, 8.7, 6.0, 4.6, 1.6)	0.03
5	a 1.57 – 1.68 (m)	a 1.68 – 1.52 (m)	
	b 2.16 (dddd, 14.0, 8.8, 4.0, 2.5)	b 2.12 (dddd, 14.1, 8.5, 3.8, 2.8)	0.04
6	a 2.83 (dt, 10.5, 4.0, 4.0)	a 2.79 (dt, 10.7, 4.1)	0.04
	b 2.61 (ddd, 13.0, 10.5, 2.5)	b 2.58 (ddd, 13.3, 10.7, 3.1)	0.03
7	3.93 (dd, 5.3, 4.5)	3.89 (dd, 5.3, 4.5)	0.04
8	a 2.72 (dd, 9.8, 4.5)	a 2.68 (dd, 9.8, 4.4)	0.04
	b 1.94 (d, 9.8)	b 1.90 (d, 9.8)	0.04
12	5.75 (s)	5.72 (s)	0.03
14	6.67 (dd, 9.1, 1.0)	6.63 (dd, 9.1, 1.1)	0.04
15	6.80 (dd, 9.1, 5.3)	6.78 (dd, 9.1, 5.2)	0.02
OMe-4	3.24 (s)	3.21 (s)	0.03

[a] The chemical shift were recorded with respect to the deuterated solvent shift ($CHCl_3$, δ 7.24 ppm for the proton).

Supplementary Table 4. Comparison of ^{13}C NMR spectroscopic data of natural and synthetic securitinine (**3**)

position	isolation report[6] δ_1 (ppm)	this work δ_2 (ppm)[a]	deviation $\Delta\delta = \delta_1 - \delta_2$ (ppm)
2	55.9	56.1	–0.2
3	26.3	26.5	–0.2
4	72.8	73.0	–0.2
5	30.6	30.8	–0.2
6	42.2	42.4	–0.2
7	58.7	58.9	–0.2
8	42.8	43.1	–0.3
9	91.4	91.7	–0.3
11	172.5	172.7	–0.2
12	109.3	109.5	–0.2
13	167.3	167.6	–0.3
14	122.9	123.0	–0.1
15	148.6	149.1	–0.5
OMe-4	55.9	56.1	–0.2

[a] The chemical shift were recorded with respect to the deuterated solvent shift ($CDCl_3$, δ 77.23 ppm for the carbon).

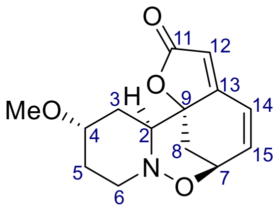

6 secu'amamine D

Supplementary Table 5. Comparison of ^{1}H-NMR spectroscopic data of natural and synthetic secu'amamine D (**6**)

position	isolation report[5] δ_1 (ppm ; multi, J in Hz)	this work δ_2 (ppm ; multi, J in Hz)[a]	deviation $\Delta\delta = \delta_1 - \delta_2$ (ppm)
2	3.19 (dd, 12.0, 2.5)	3.16 (dd, 12.0, 2.6)	0.03
3	a 1.02 (ddd, 14.1, 12.0, 2.6)	a 0.99 (ddd, 13.7, 12.0, 2.6)	0.03
	b 2.04 (m)	b 2.00 (m)	0.04
4	3.43 (br dt, 5.5, 2.7)	3.40 (dt, 5.6, 2.8)	0.03
5	a 1.67 (tq, 10.9, 2.8)	a 1.64 (dddd, 14.2, 12.6, 5.4, 2.8)	0.03
	b 1.91 (ddt, 14.2, 5.8, 2.9)	b 1.88 (ddt, 14.2, 5.8, 3.0)	0.03
6	a 2.96 (m)	a 2.94 (m)	0.02
	b 2.96 (m)	b 2.90 (m)	0.06
7	4.74 (dt, 5.8, 3.4)	4.71 (ddd, 5.9, 3.4, 2.4)	0.03
8	a 2.05 (m)	a 2.00 (m)	0.05
	b 2.54 (dd, 11.4, 3.4)	b 2.51 (dd, 11.4, 3.4)	0.03
12	5.85 (s)	5.82 (s)	0.03
14	6.88 (d, 9.4)	6.85 (d, 9.4)	0.03
15	6.30 (dd, 9.4, 5.8)	6.27 (dd, 9.4, 5.8)	0.03
OMe-4	3.29 (s)	3.26 (s)	0.03

[a] The chemical shift were recorded with respect to the deuterated solvent shift ($CHCl_3$, δ 7.24 ppm for the proton).

Supplementary Table 6. Comparison of ^{13}C NMR spectroscopic data of natural and synthetic secu'amamine D (**6**)

position	isolation report[5] δ_1 (ppm)	this work δ_2 (ppm)[a]	deviation $\Delta\delta = \delta_1 - \delta_2$ (ppm)
2	65.2	65.4	–0.2
3	27.1	27.2	–0.1
4	72.0	72.2	–0.2
5	29.3	29.4	–0.1
6	50.3	50.4	–0.1
7	70.9	71.1	–0.2
8	40.6	40.8	–0.2
9	82.6	82.8	–0.2
11	171.9	172.1	–0.2
12	113.4	113.6	–0.2
13	164.2	164.4	–0.2
14	126.4	126.6	–0.2
15	134.4	134.6	–0.2
OMe-4	55.8	56.0	–0.2

[a] The chemical shift were recorded with respect to the deuterated solvent shift ($CDCl_3$, δ 77.23 ppm for the carbon).

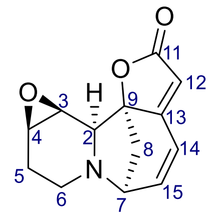

8b securingine C

Supplementary Table 7. Comparison of ^{1}H-NMR spectroscopic data of natural and synthetic securingine C (**8**)

position	isolation report[3] δ_1 (ppm ; multi, *J* in Hz)	this work δ_2 (ppm ; multi, *J* in Hz)[a]	deviation $\Delta\delta = \delta_1 - \delta_2$ (ppm)
2	4.05 (br s)	3.99 (s)	0.06
3	3.09 (d, 4.7)	3.04 (d, 4.7)	0.05
4	3.28 (t, 4.0)	3.22 (t, 4.0)	0.06
5	a 2.09 (m)	a 2.03 (m)	0.06
	b 2.09 (m)	b 2.03 (m)	0.06
6	a 2.86 (m)	a 2.80 (m)	0.06
	b 2.80 (m)	b 2.74 (m)	0.06
7	3.88 (t, 4.8)	3.83 (t, 4.7)	0.05
8	a 2.66 (dd, 9.9, 4.8)	a 2.60 (dd, 9.9, 4.6)	0.06
	b 1.91 (d, 9.9)	b 1.85 (d, 9.9)	0.06
12	5.83 (s)	5.77 (s)	0.06
14	6.79 (dd, 9.1, 1.0)	6.74 (dd, 9.0, 1.3)	0.05
15	6.85 (dd, 9.1, 5.2)	6.80 (dd, 9.1, 5.1)	0.05

[a] The chemical shift were recorded with respect to the deuterated solvent shift ($CHCl_3$, δ 7.24 ppm for the proton).

Supplementary Table 8. Comparison of ^{13}C NMR spectroscopic data of natural and synthetic securingine C (**8**)

position	isolation report[3] δ_1 (ppm)	this work δ_2 (ppm)[a]	deviation $\Delta\delta = \delta_1 - \delta_2$ (ppm)
2	59.7	59.7	0
3	49.9	49.9	0
4	49.3	49.4	–0.1
5	24.2	24.2	0
6	42.3	42.3	0
7	58.8	58.8	0
8	43.3	43.3	0
9	90.3	90.3	0
11	172.6	172.6	0
12	109.2	109.1	0.1
13	167.8	167.8	0
14	123.8	123.8	0
15	147.9	148.0	–0.1

[a] The chemical shift were recorded with respect to the deuterated solvent shift ($CDCl_3$, δ 77.23 ppm for the carbon).

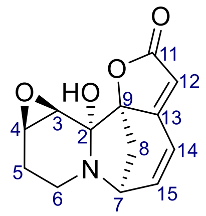

9b securingine D

Supplementary Table 9. Comparison of ^{1}H-NMR spectroscopic data of natural and synthetic securingine D (**9**)

position	isolation report[3] δ_1 (ppm ; multi, *J* in Hz)	this work δ_2 (ppm ; multi, *J* in Hz)[a]	deviation $\Delta\delta = \delta_1 - \delta_2$ (ppm)
3	3.01 (d, 4.5)	2.97 (d, 4.5)	0.04
4	3.34 (t, 4.2)	3.29 (t, 4.1)	0.05
5	a 2.29 (m)	a 2.25 (ddd, 14.7, 12.7, 5.2)	0.04
	b 2.16 (m)	b 2.11 (m)	0.05
6	a 2.93 (m)	a 2.89 (ddd, 9.5, 5.2, 2.1)	0.04
	b 2.82 (m)	b 2.77 (ddd, 13.2, 9.4, 4.0)	0.05
7	3.92 (br t, 4.8)	3.88 (t, 4.8)	0.04
8	a 2.89 (dd, 10.0, 4.8)	a 2.84 (dd, 10.0, 4.8)	0.05
	b 1.90 (d, 10.0)	b 1.85 (d, 10.0)	0.05
12	5.90 (s)	5.86 (s)	0.04
14	6.76 (dd, 9.0, 1.2)	6.72 (dd, 9.0, 1.3)	0.04
15	6.90 (dd, 9.0, 5.2)	6.85 (dd, 9.0, 5.2)	0.05
OH-2		2.62 (br s)	

[a] The chemical shift were recorded with respect to the deuterated solvent shift ($CHCl_3$, δ 7.24 ppm for the proton).

Supplementary Table 10. Comparison of ^{13}C NMR spectroscopic data of natural and synthetic securingine D (**9**)

position	isolation report[3] δ_1 (ppm)	this work δ_2 (ppm)[a]	deviation $\Delta\delta = \delta_1 - \delta_2$ (ppm)
2	86.3	86.4	–0.1
3	51.3	51.3	0
4	49.6	49.6	0
5	23.2	23.2	0
6	40.6	40.7	–0.1
7	56.3	56.3	0
8	40.5	40.6	–0.1
9	91.4	91.4	0
11	172.1	172.2	–0.1
12	110.1	110.1	0
13	164.0	164.1	–0.1
14	123.7	123.7	0
15	147.4	147.4	0

[a]The chemical shift were recorded with respect to the deuterated solvent shift ($CDCl_3$, δ 77.23 ppm for the carbon).

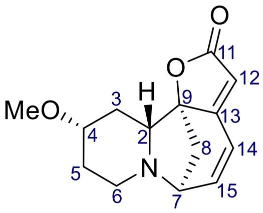

4 4-*epi*-phyllanthine

Supplementary Table 11. Comparison of ^{1}H-NMR spectroscopic data of natural and synthetic 4-*epi*-phyllanthine (**4**)

position	isolation report[6] δ_1 (ppm ; multi, *J* in Hz)	this work δ_2 (ppm ; multi, *J* in Hz)[a]	deviation $\Delta\delta = \delta_1 - \delta_2$ (ppm)
2	2.09 (dd, 11.2, 2.0)	2.07 (dd, 11.8, 2.3)	0.02
3	a 1.45 (app q, 11.2, 10.9, 10.9)	a 1.43 (td, 11.6, 10.4)	0.02
	b 2.15 (m)	2.12 (ddt, 11.3, 4.6, 1.7)	0.03
4	3.12 (app tt, 10.9, 10.2, 4.0, 4.0)	3.11 (tt, 10.2, 4.9)	0.01
5	a 1.57 (dddd, 12.5, 10.9, 10.2, 5.3)	a 1.54 (dtd, 12.6, 10.7, 5.3)	0.03
	b 1.97 (m)	b 1.93 (m)	0.04
6	a 3.00 (ddd, 10.9, 5.3, 3.1)	a 2.98 (ddd, 10.9, 5.3, 3.0)	0.02
	b 2.42 (td, 10.9, 2.9)	b 2.41 (td, 10.9, 3.0)	0.01
7	3.81 (t, 4.6)	3.79 (t, 4.6)	0.02
8	a 2.54 (dd, 9.4, 4.6)	a 2.53 (dd, 9.4, 4.2)	0.01
	b 1.81 (d, 9.4)	b 1.79 (d, 9.5)	0.02
12	5.56 (s)	5.55 (s)	0.01
14	6.59 (d, 9.2)	6.57 (d, 9.3)	0.02
15	6.45 (dd, 9.2, 4.6)	6.41 (dd, 9.2, 5.3)	0.04
OMe-4	3.32 (s)	3.31 (s)	0.01

[a] The chemical shift were recorded with respect to the deuterated solvent shift ($CHCl_3$, δ 7.24 ppm for the proton).

Supplementary Table 12. Comparison of ^{13}C NMR spectroscopic data of natural and synthetic 4-*epi*-phyllanthine (**4**)

position	isolation report[6] δ_1 (ppm)	this work δ_2 (ppm)[a]	deviation $\Delta\delta = \delta_1 - \delta_2$ (ppm)
2	59.8	60.1	–0.3
3	32.4	32.7	–0.3
4	78.0	78.3	–0.3
5	32.4	32.7	–0.3
6	45.6	45.8	–0.2
7	58.2	58.4	–0.2
8	42.3	42.6	–0.3
9	89.2	89.4	–0.2
11	173.4	173.6	–0.2
12	105.5	105.8	–0.3
13	169.7	169.9	–0.2
14	121.5	121.7	–0.2
15	140.2	140.4	–0.2
OMe-4	55.8	56.0	–0.2

[a] The chemical shift were recorded with respect to the deuterated solvent shift ($CDCl_3$, δ 77.23 ppm for the carbon).

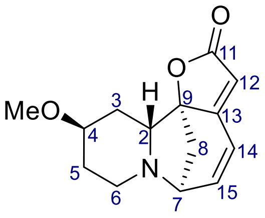

5 phyllanthine

Supplementary Table 13. Comparison of ^{1}H-NMR spectroscopic data of natural and synthetic phyllanthine (**5**)

position	isolation report[6] (ppm ; multi, *J* in Hz)	Weinreb group[8] (ppm ; multi, *J* in Hz)	this work (ppm ; multi, *J* in Hz)[a]
2	2.59 (dd, 12.2, 2.4)	2.59 (dd, 12.2, 2.4)	2.56 (dd, 12.3, 2.5)
3	a 1.68 (ddd, 13.1, 12.2, 2.9)	a 1.68 (ddd, 12.4, 12.4, 2.9)	a 1.65 (ddd, 13.1, 12.2, 2.9)
	b 1.95 (dddd, 13.1, 2.6, 2.4, 1.4)	b 1.94 (dddd, 13.1, 2.5, 2.5, 1.4)	b 1.92 (dtd, 13.1, 2.7, 1.6)
4	3.63 (dddd, ca. 3, ca. 3, 2.9, 2.6)	3.63 (dddd, 3.1, 3.1, 3.0, 3.0)	3.60 (p, 3.0)
5	1.70 – 1.90 (m)	1.87 – 1.73 (m)	1.84 – 1.69 (m)
	1.70 – 1.90 (m)	1.87 – 1.73 (m)	1.84 – 1.69 (m)
6	a 2.79 (ddd, 10.4, 5.3, 3.4)	a 2.79 (ddd, 10.6, 5.4, 3.3)	a 2.76 (ddd, 10.6, 5.3, 3.2)
	b 2.67 (td, 10.4, 3.4)	b 2.67 (ddd, 10.2, 10.2, 3.8)	b 2.64 (td, 10.5, 3.5)
7	3.81 (dd, 5.3, 4.2)	3.80 (t, 4.7)	3.77 (dd, 5.3, 4.2)
8	a 2.52 (dd, 9.4, 4.2)	a 2.52 (dd, 9.3, 4.1)	a 2.49 (dd, 9.3, 4.2)
	b 1.78 (d, 9.4)	b 1.78 (d, 8.9)	b 1.76 (d, 9.5)
12	5.55 (s)	5.55 (s)	5.52 (s)
14	6.58 (d, 9.1)	6.58 (d, 9.2)	6.56 (d, 9.1)
15	6.43 (dd, 9.1, 5.3)	6.43 (dd, 9.2, 5.3)	6.40 (dd, 9.2, 5.3)
OMe-4	3.27 (s)	3.26 (s)	3.24 (s)

[a] The chemical shift were recorded with respect to the deuterated solvent shift ($CHCl_3$, δ 7.24 ppm for the proton).

Supplementary Table 14. Comparison of ^{13}C NMR spectroscopic data of natural and synthetic phyllanthine (**5**)

position	isolation report[6] (ppm)	Weinreb group[8,a] (ppm)	this work (ppm)[b]
2	56.2	56.7	56.5
3	31.1	31.5	31.3
4	74.2	74.7	74.5
5	30.6	31.1	30.9
6	44.5	44.9	44.8
7	58.8	59.2	59.0
8	41.9	42.4	42.2
9	89.3		89.5
11	173.6		173.8
12	105.4	105.8	105.6
13	170.1		170.3
14	121.6	122.1	121.8
15	140.3	140.7	140.5
OMe-4	56.0	56.5	56.3

[a] The assignment was based on ^{13}C NMR DEPT experiment.

[b] The chemical shift were recorded with respect to the deuterated solvent shift ($CDCl_3$, δ 77.23 ppm for the carbon).

5. Comparison of NMR spectra of authentic and synthetic natural products (only for cases where NMR spectra are provided in the isolation report).

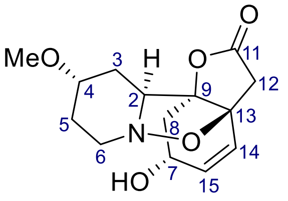

7b securingine A

Supplementary Figure 7. ^{1}H NMR of securingine A in $CDCl_3$ from the isolation report.[3]

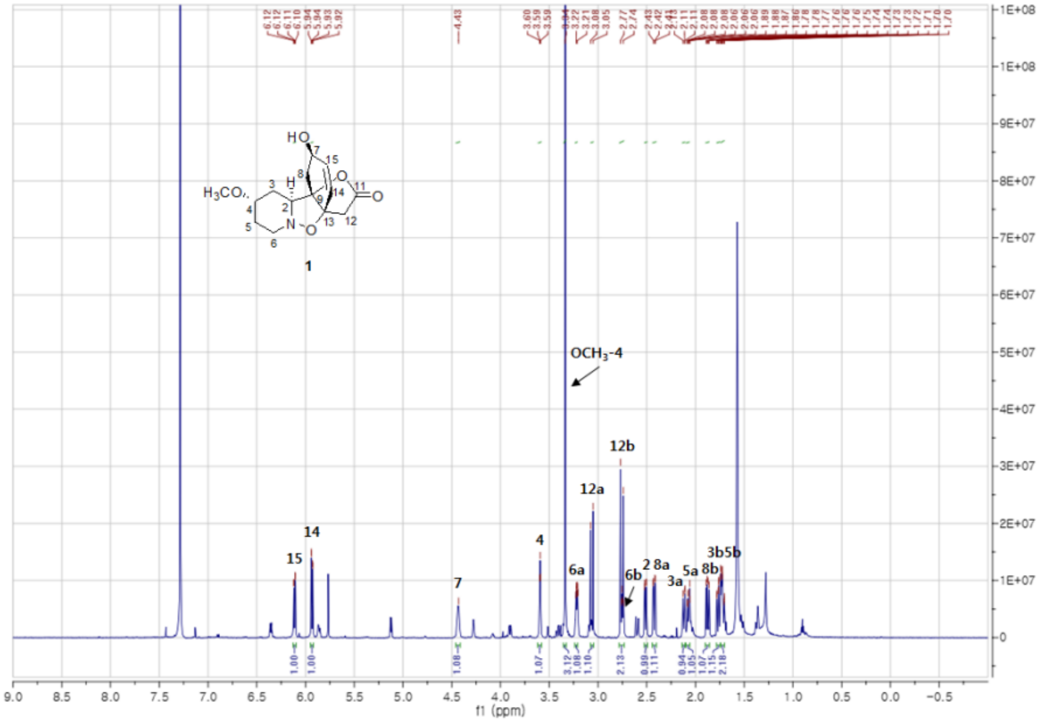

Supplementary Figure 8. ^{1}H NMR of our synthetic securingine A in $CDCl_3$.

Parameter	Value
Solvent	CDCl3
Spectrometer Frequency	500.23
Nucleus	1H

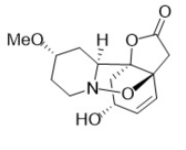

7b securingine A

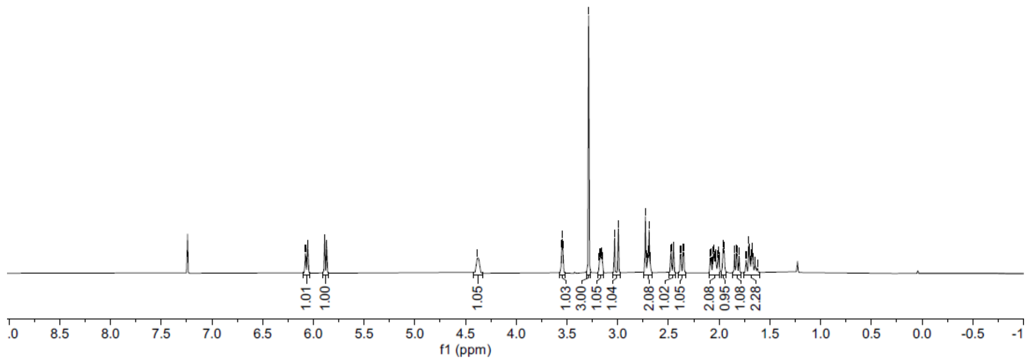

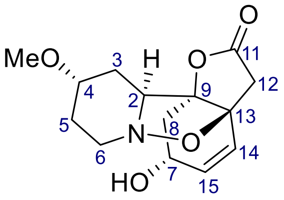

7b securingine A

Supplementary Figure 9. ^{13}C NMR of securingine A in $CDCl_3$ from the isolation report.[3]

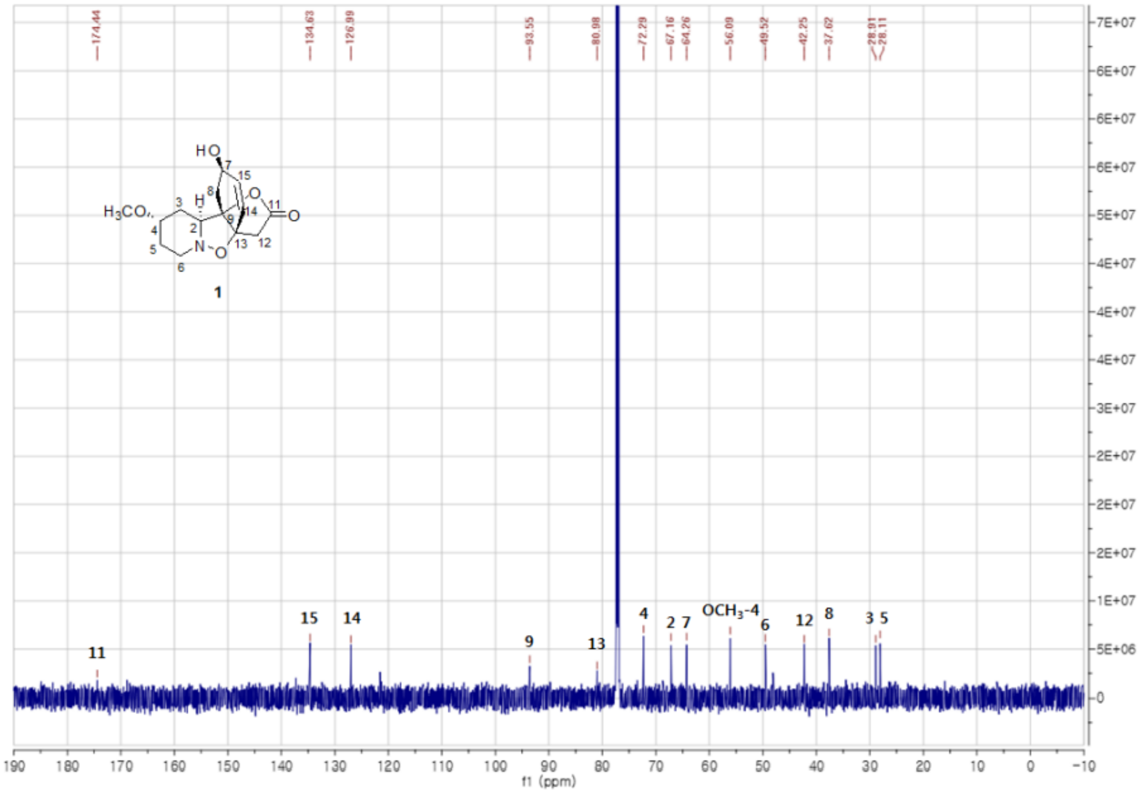

Supplementary Figure 10. ^{13}C NMR of our synthetic securingine A in $CDCl_3$.

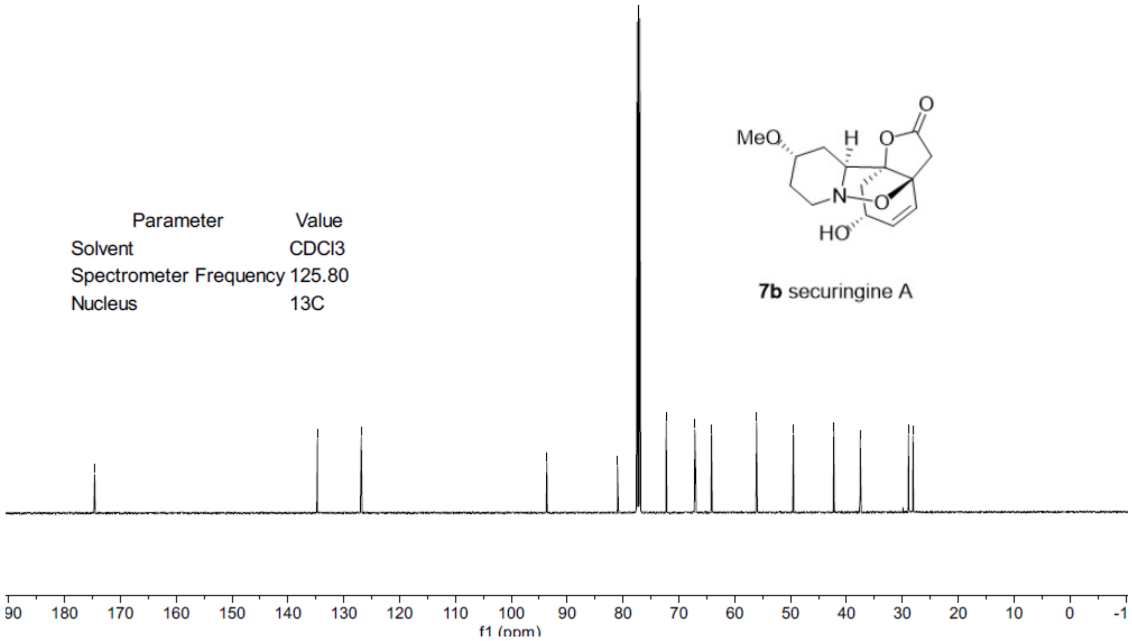

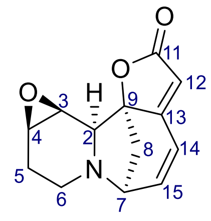

8b securingine C

Supplementary Figure 11. ^{1}H NMR of securingine C in $CDCl_3$ from the isolation report.[3]

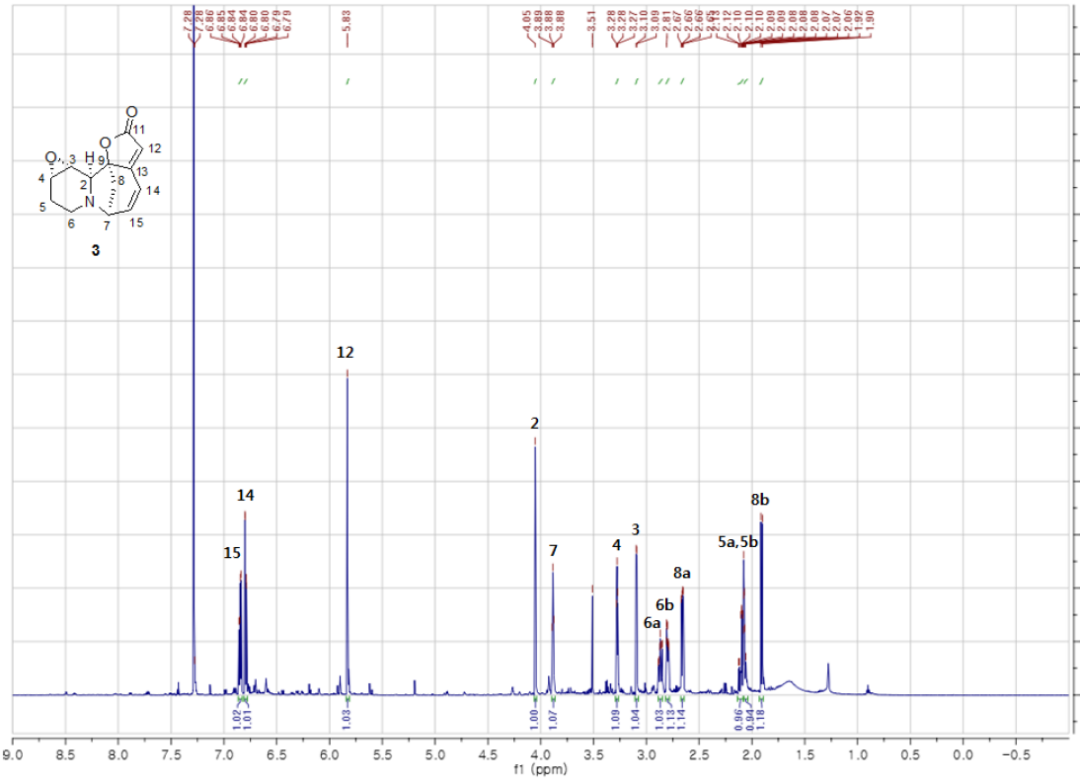

Supplementary Figure 12. ^{1}H NMR of our synthetic securingine C in $CDCl_3$.

Parameter	Value
Solvent	CDCl3
Spectrometer Frequency	400.23
Nucleus	1H

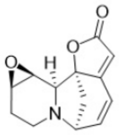

8b securingine C

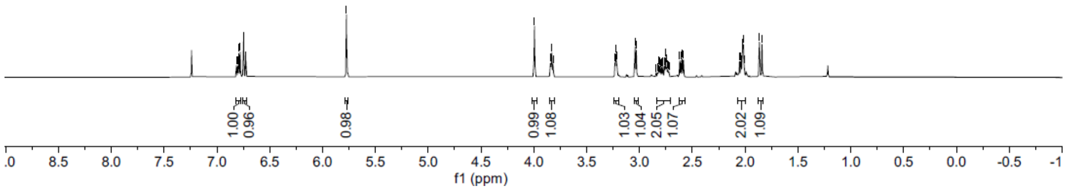

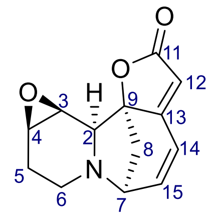

8b securingine C

Supplementary Figure 13. ^{13}C NMR of securingine C in $CDCl_3$ from the isolation report.[3]

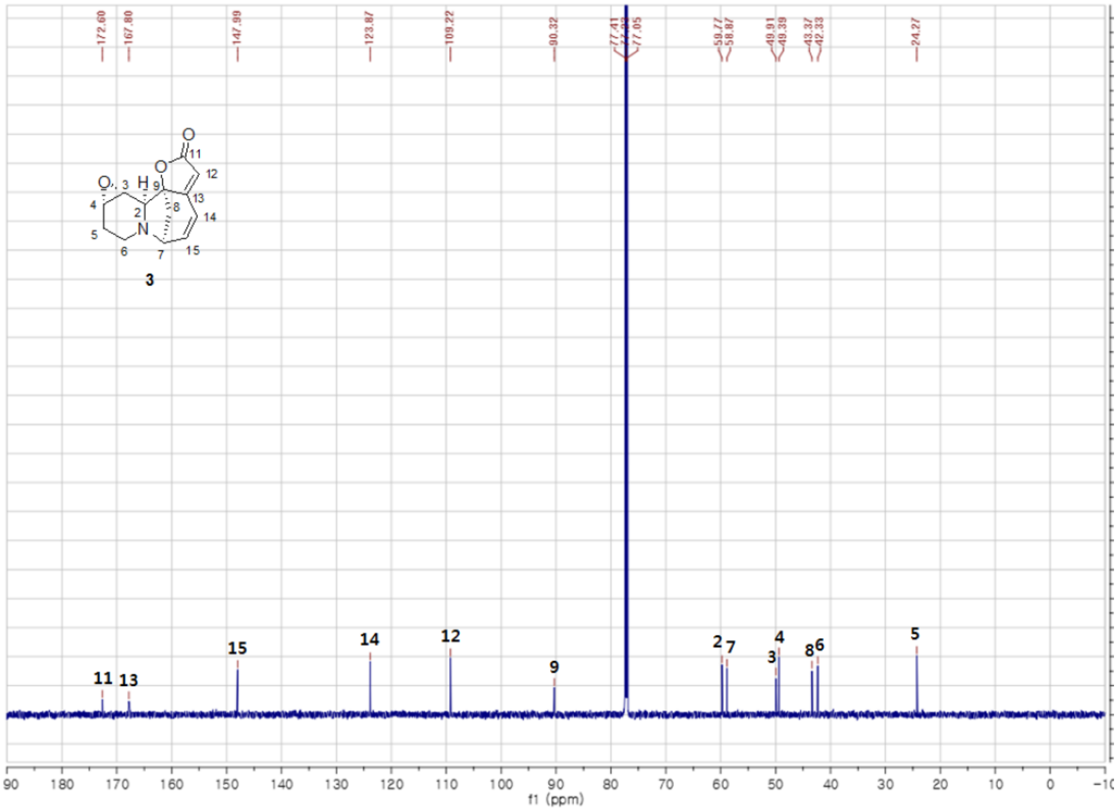

Supplementary Figure 14. ^{13}C NMR of our synthetic securingine C in $CDCl_3$.

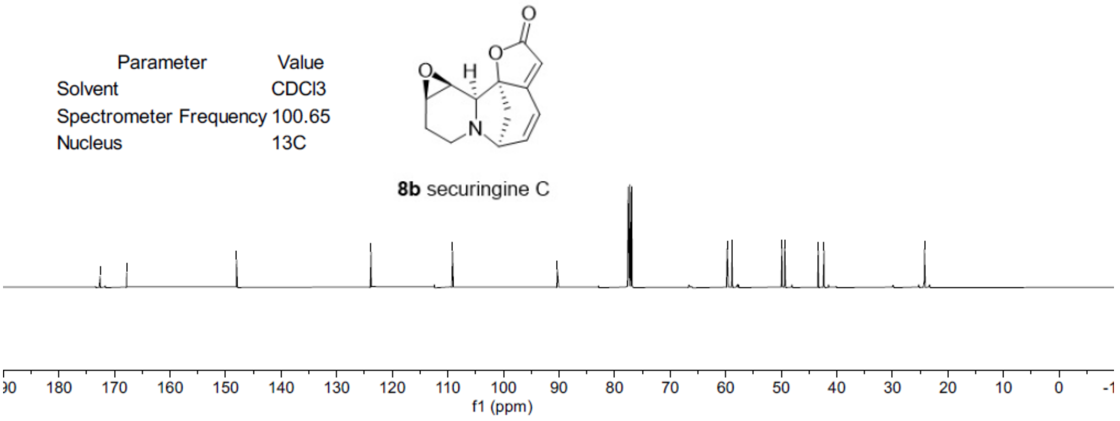

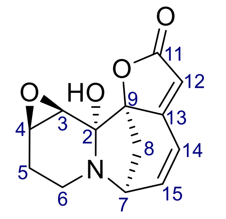

9b securingine D

Supplementary Figure 15. ^{13}C NMR of securingine D in CDCl$_3$ from the isolation report.[3]

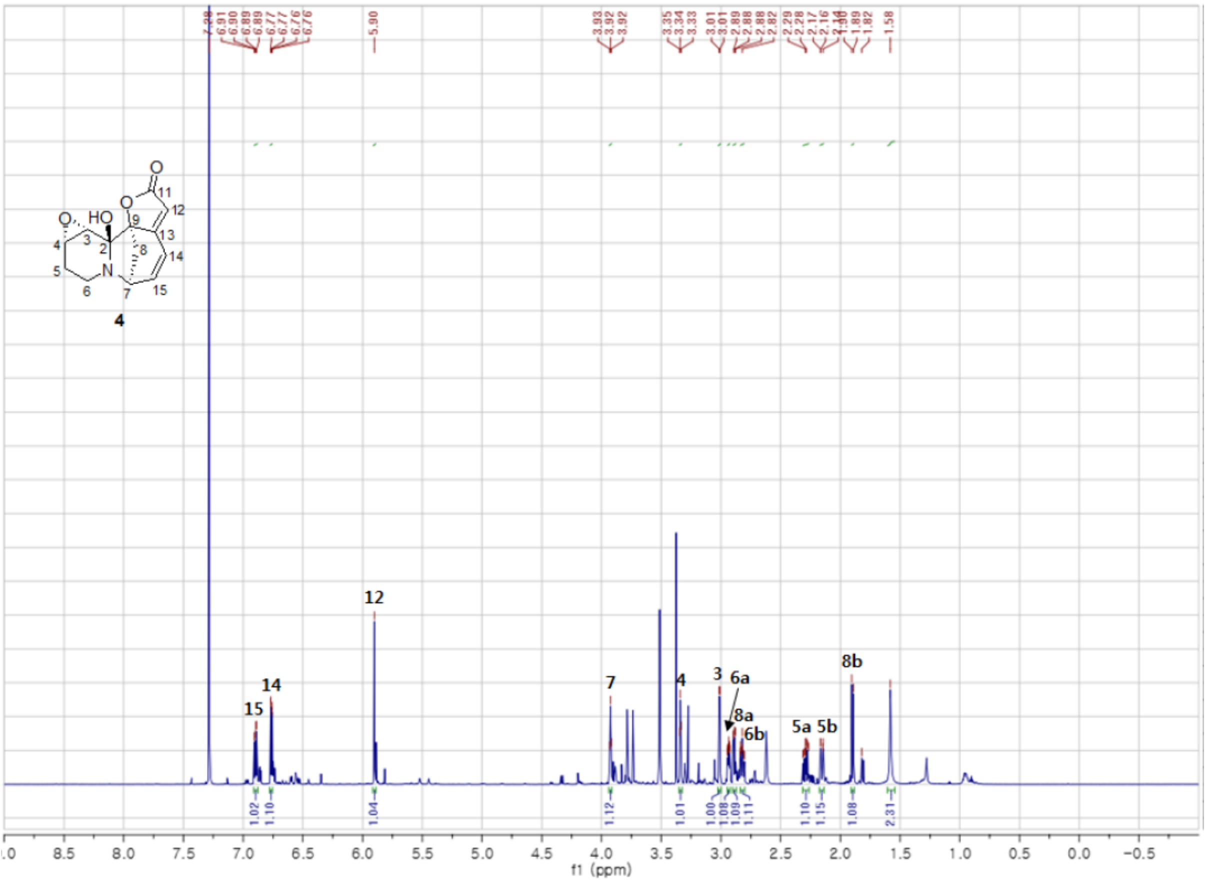

Supplementary Figure 16. ^{13}C NMR of our synthetic securingine D in CDCl$_3$.

Parameter	Value
Solvent	CDCl3
Spectrometer Frequency	400.12
Nucleus	1H

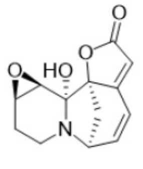

9b securingine D

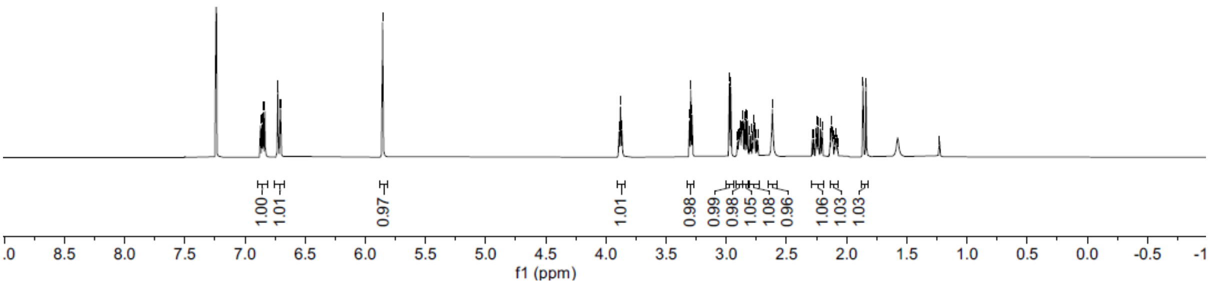

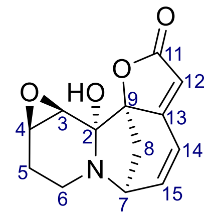

9b securingine D

Supplementary Figure 17. ^{13}C NMR of securingine D in $CDCl_3$ from the isolation report.[3]

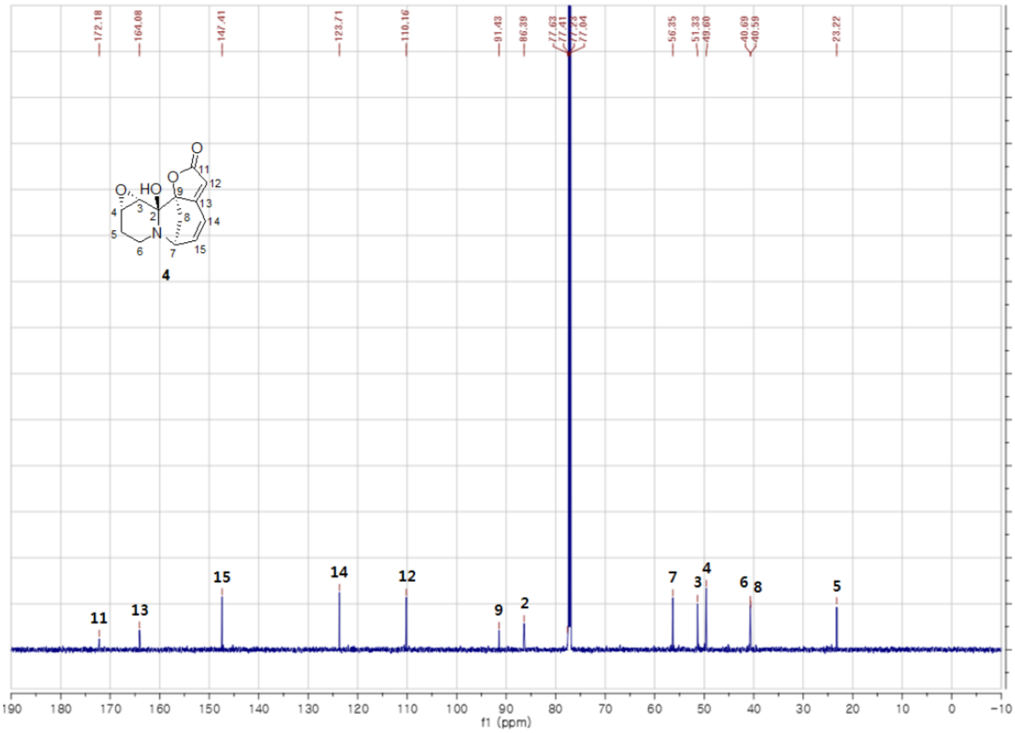

Supplementary Figure 18. ^{13}C NMR of our synthetic securingine D in $CDCl_3$.

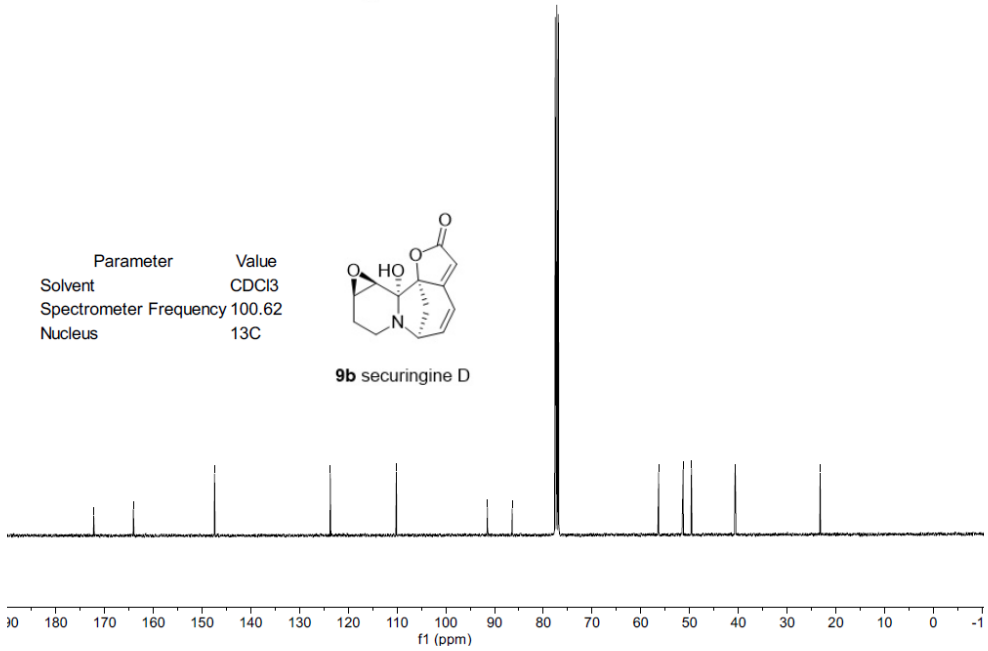

6. Computational Studies Regarding the Thermodynamics of C2-Epimerization

6.1. Computational Details

All calculations except single point calculations were conducted using DFT[9] as implemented in the Jaguar 9.1 suite[10] of *ab initio* quantum chemistry programs with M06-2X levels of theory.[11] Geometry optimizations were proceeded using the 6-31G** basis set. More accurate single point energies were computed from the optimized geometries using Dunning's correlation-consistent triple-ζ basis set, cc-pVTZ(-f)[12] that includes a double set of polarization functions. Analytical vibrational frequencies within the harmonic approximation were calculated using the 6-31G** basis to confirm proper convergence to well-defined minima or saddle points on the potential energy surface. Solvation energies were calculated using a self-consistent reaction field (SCRF)[13,14,15] approach based on accurate numerical solutions of the Poisson-Boltzmann equation and were performed with the 6-31G** basis at the optimized gas phase geometry with the dielectric constant of $\varepsilon = 32.36$ for methanol. As is the case for all continuum models, the solvation energies are subject to empirical parametrization of the atomic radii that are used to generate the solute surface. The standard set of optimized radii in Jaguar was used for H (1.150 Å), C (1.900 Å), N (1.600 Å), O (1.600 Å).[16]

The Gibbs free energies in solution phase G(sol) were computed with the following protocol.

$$G(sol) = G(gas) + G^{solv} \quad (1)$$

$$G(gas) = H(gas) - TS(gas) \quad (2)$$

$$H(gas) = E(scf) + ZPE \quad (3)$$

G(gas) is the free energy in gas phase; G^{solv} is the free energy of solvation; H(gas) is the enthalpy in gas phase; T is the temperature (298.15K); S(gas) is the entropy in gas phase; E(SCF) is "raw" electronic energy as computed from the SCF procedure which is the self-consistent field energy, and ZPE is the zero point energy. The entropy we refer is specifically vibrational/rotational/translational entropy of the solute(s), and the entropy of the solvent is implicitly comprised in the continuum solvation model.

6.2. Ground-state conformation and thermodynamics of C2-epimerization

Supplementary Figure 19. (a) Calculated structure of the ground-state conformer of **30**. (b) Calculated structure of the ground-state conformer of **42**.

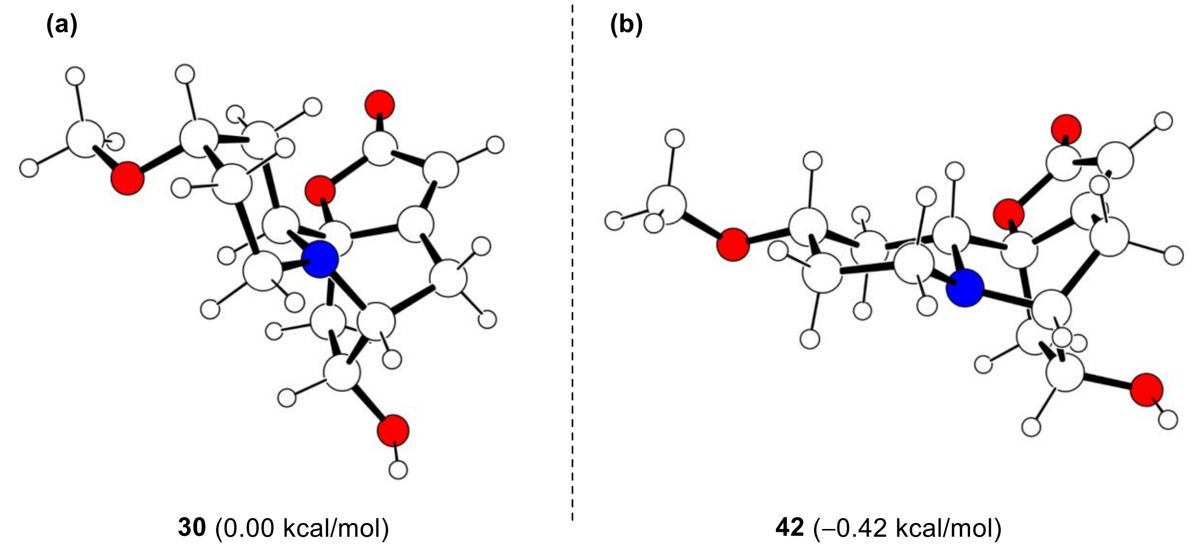

30 (0.00 kcal/mol) **42** (–0.42 kcal/mol)

Supplementary Figure 19 shows ground-state conformer of compound **30** and **42**, calculated difference of solution-phase free energy indicates slight thermodynamic preference toward **42** over **30** in equilibrium. The C4-methoxy group on the A ring of **30** possesses the axial position of the cyclohexane ring whereas the C4-methoxy group of **42** resides in the equatorial position.

Supplementary Figure 20. (a) Calculated structure of the ground-state conformer of **50**. (b) Calculated structure of the ground-state conformer of **51**.

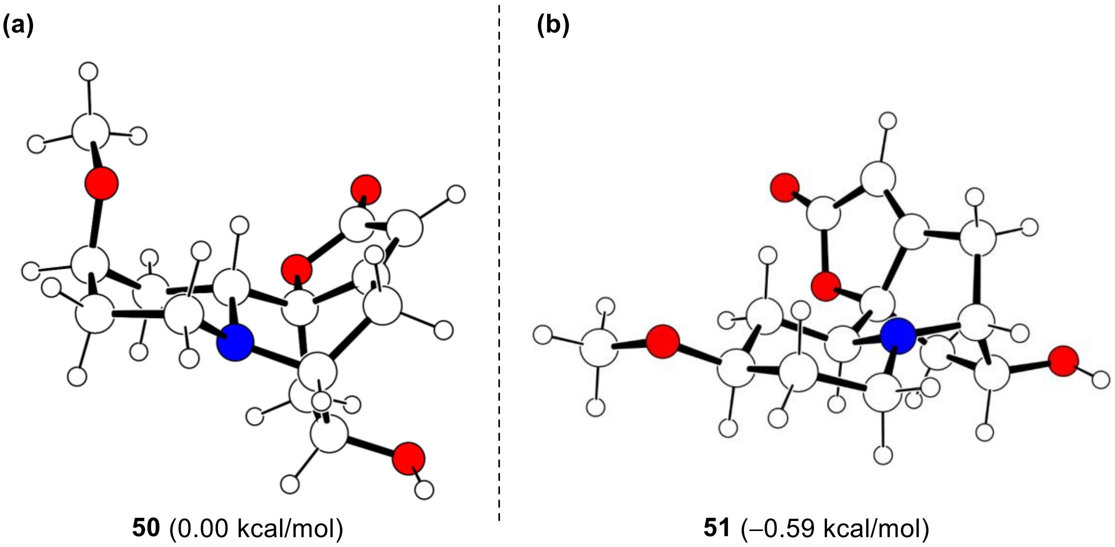

50 (0.00 kcal/mol) **51** (–0.59 kcal/mol)

Supplementary Figure 20 shows ground-state conformer of compound **50** and **51**, calculated difference of solution-phase free energy indicates slight thermodynamic preference toward **51** over **50** in equilibrium. The C4-methoxy group on the A ring of **50** possesses the axial position of the cyclohexane ring whereas the C4-methoxy group of **51** resides in the equatorial position.

Supplementary Figure 21. (a) Calculated structure of the ground-state conformer of secu'amamine E (**13**). (b) Calculated structure of the ground-state conformer of *ent*-virosine B (**44**).

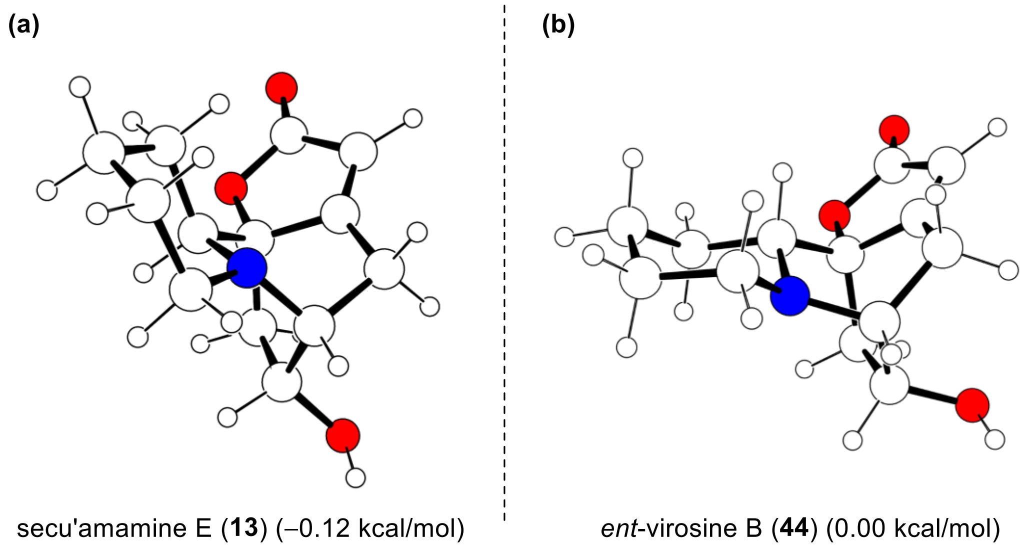

secu'amamine E (**13**) (–0.12 kcal/mol) *ent*-virosine B (**44**) (0.00 kcal/mol)

Supplementary Figure 21 shows ground-state conformer of compound secu'amamine E (**13**) and *ent*-virosine B (**44**). The small calculated difference (0.12 kcal/mol) of solution-phase free energy between **13** and **44** is in line with experimentally observed comparable formation of **13** and **44** during their C2-epimerization reactions. This result indicates that the configuration of the C4-methoxy group is the key factor in the thermodynamics of the C2-epimerization reaction.

6.3. DFT-optimized structure's energy components

Supplementary Table 15. Computed energy components for optimized structures.

	**E(SCF)/(eV)**	**ZPE/(kcal/mol)**	**S(gas)/(cal/mol·K)**	**G^{solv}/(kcal/mol)**
	M06-2X/cc-pVTZ(-f)	M06-2X/6-31G**	M06-2X/6-31G**	M06-2X/6-31G**
**30**	-24504.084	205.16	125.15	-16.35
**42**	-24504.031	204.97	125.71	-17.63
**50**	-24504.082	205.29	124.31	-16.56
**51**	-24504.051	204.89	125.29	-17.17
**13**	-21387.895	184.27	112.04	-14.42
**44**	-21387.893	184.40	111.77	-14.56

7. Single Crystal X-Ray Diffraction (SCXD) Analysis of Securingine D (9b)

Supplementary Figure 22. Thermal ellipsoid representation of securingine D (**9b**) shown at the 50% probability level

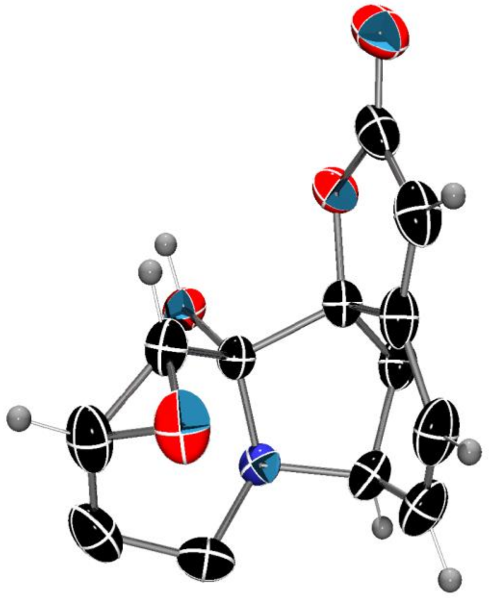

front view

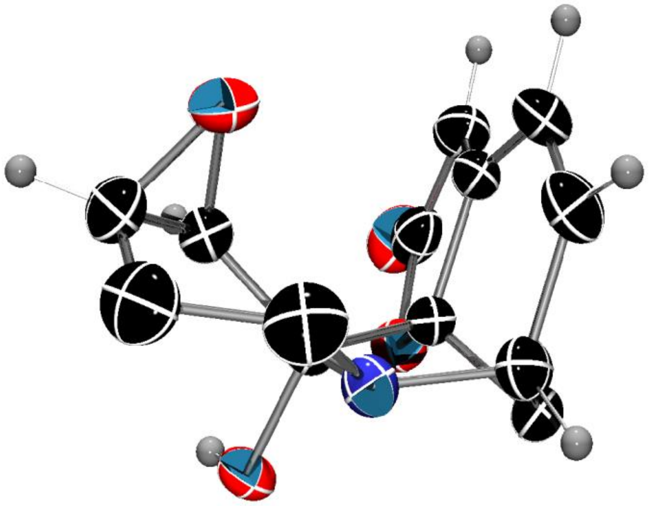

bottom view

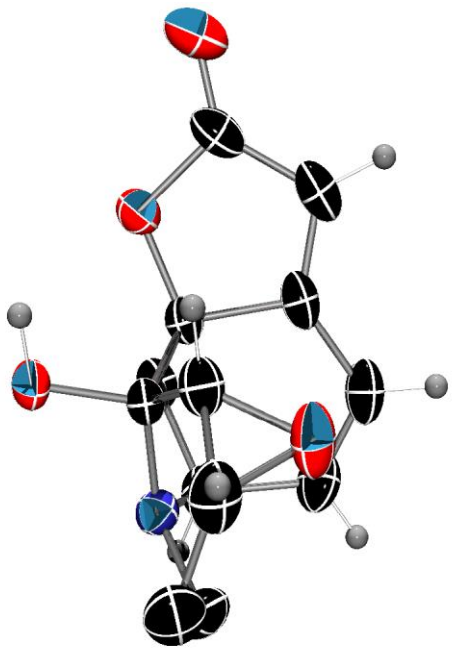

side view

Supplementary Table 16. Crystal data and structure refinement for securingine D (**9b**).

Empirical formula	$C_{13} H_{13} N O_4$	
Formula weight	247.24	
Temperature	296(2) K	
Wavelength	0.71073 Å	
Crystal system	Orthorhombic	
Space group	$P2_12_12_1$	
Unit cell dimensions	a = 7.0060(4) Å	α = 90°
	b = 8.1826(5) Å	β = 90°
	c = 19.5547(10) Å	γ = 90°
Volume	1121.02(11) $Å^3$	
Z	4	
Density (calculated)	1.465 Mg/m^3	
Absorption coefficient	0.110 mm^{-1}	
F(000)	520	
Crystal size	0.351 x 0.103 x 0.079 mm^3	
Theta range for data collection	2.698 to 28.388°.	
Index ranges	–9<=h<=9, –10<=k<=10, –26<=l<=26	
Reflections collected	52894	
Independent reflections	2803 [R(int) = 0.0502]	
Completeness to theta = 25.242°	99.9 %	
Absorption correction	Semi-empirical from equivalents	
Max. and min. transmission	0.7457 and 0.7036	
Refinement method	Full-matrix least-squares on F^2	
Data / restraints / parameters	2803 / 0 / 175	
Goodness-of-fit on F^2	1.076	
Final R indices [I>2sigma(I)]	R1 = 0.0309, wR2 = 0.0784	
R indices (all data)	R1 = 0.0335, wR2 = 0.0802	
Absolute structure parameter	0.1(2)	
Largest diff. peak and hole	0.154 and –0.165 $e·Å^{–3}$	

Supplementary Table 17. Atomic coordinates (x 10^4) and equivalent isotropic displacement parameters ($Å^2$ x 10^3) for securingine D (**9b**). U(eq) is defined as one third of the trace of the orthogonalized U^{ij} tensor.

	x	y	z	U(eq)
O(1)	3535(2)	9813(1)	4095(1)	36(1)
O(2)	6177(2)	11070(2)	3727(1)	54(1)
C(3)	4899(3)	10140(2)	3603(1)	40(1)
C(4)	4414(3)	9238(2)	2986(1)	44(1)
C(5)	2792(3)	8415(2)	3094(1)	36(1)
C(6)	2170(2)	8693(2)	3821(1)	30(1)
C(7)	2068(2)	7105(2)	4257(1)	28(1)
O(8)	2144(2)	7526(2)	4963(1)	34(1)
C(9)	3668(3)	5907(2)	4136(1)	36(1)
C(10)	3249(3)	4179(2)	4231(1)	48(1)
O(11)	3549(2)	4851(2)	3550(1)	51(1)
C(12)	1241(4)	3713(2)	4401(1)	54(1)
C(13)	-170(3)	4757(2)	4009(1)	49(1)
N(14)	151(2)	6507(2)	4124(1)	32(1)
C(15)	96(2)	9249(2)	3819(1)	35(1)
C(16)	-773(2)	7608(2)	3620(1)	38(1)
C(17)	-235(3)	7200(3)	2885(1)	47(1)
C(18)	1495(3)	7552(3)	2648(1)	45(1)

Supplementary Table 18. Bond lengths [Å] and angles [°] for securingine D (**9b**).

O(1)-C(3)	1.382(2)
O(1)-C(6)	1.429(2)
O(2)-C(3)	1.200(2)
C(3)-C(4)	1.456(3)
C(4)-C(5)	1.337(3)
C(4)-H(4)	0.9300
C(5)-C(18)	1.444(3)
C(5)-C(6)	1.505(2)
C(6)-C(15)	1.523(2)
C(6)-C(7)	1.556(2)
C(7)-O(8)	1.4237(17)
C(7)-N(14)	1.453(2)
C(7)-C(9)	1.507(2)
O(8)-H(8)	0.90(3)
C(9)-O(11)	1.438(2)
C(9)-C(10)	1.456(3)
C(9)-H(9)	0.94(2)
C(10)-O(11)	1.456(2)
C(10)-C(12)	1.495(3)
C(10)-H(10)	1.00(3)
C(12)-C(13)	1.514(3)
C(12)-H(12A)	0.9700
C(12)-H(12B)	0.9700
C(13)-N(14)	1.466(2)
C(13)-H(13A)	0.9700
C(13)-H(13B)	0.9700
N(14)-C(16)	1.484(2)
C(15)-C(16)	1.525(3)
C(15)-H(15A)	0.9700
C(15)-H(15B)	0.9700
C(16)-C(17)	1.523(2)
C(16)-H(16)	1.03(2)
C(17)-C(18)	1.329(3)
C(17)-H(17)	0.9300

C(18)-H(18)	0.9300
C(3)-O(1)-C(6)	109.03(13)
O(2)-C(3)-O(1)	119.94(18)
O(2)-C(3)-C(4)	131.56(17)
O(1)-C(3)-C(4)	108.49(15)
C(5)-C(4)-C(3)	108.87(15)
C(5)-C(4)-H(4)	125.6
C(3)-C(4)-H(4)	125.6
C(4)-C(5)-C(18)	133.33(16)
C(4)-C(5)-C(6)	108.60(16)
C(18)-C(5)-C(6)	117.51(16)
O(1)-C(6)-C(5)	104.93(13)
O(1)-C(6)-C(15)	116.59(13)
C(5)-C(6)-C(15)	108.62(13)
O(1)-C(6)-C(7)	111.14(12)
C(5)-C(6)-C(7)	113.92(12)
C(15)-C(6)-C(7)	101.95(12)
O(8)-C(7)-N(14)	106.84(12)
O(8)-C(7)-C(9)	106.34(12)
N(14)-C(7)-C(9)	116.10(13)
O(8)-C(7)-C(6)	109.14(12)
N(14)-C(7)-C(6)	103.04(12)
C(9)-C(7)-C(6)	115.02(13)
C(7)-O(8)-H(8)	108.7(15)
O(11)-C(9)-C(10)	60.37(12)
O(11)-C(9)-C(7)	118.20(15)
C(10)-C(9)-C(7)	117.50(17)
O(11)-C(9)-H(9)	114.7(14)
C(10)-C(9)-H(9)	120.4(13)
C(7)-C(9)-H(9)	114.8(14)
O(11)-C(10)-C(9)	59.20(11)
O(11)-C(10)-C(12)	115.84(18)
C(9)-C(10)-C(12)	117.75(17)
O(11)-C(10)-H(10)	111.8(14)
C(9)-C(10)-H(10)	116.7(15)
C(12)-C(10)-H(10)	120.3(14)
C(9)-O(11)-C(10)	60.43(12)

C(10)-C(12)-C(13)	110.96(17)
C(10)-C(12)-H(12A)	109.4
C(13)-C(12)-H(12A)	109.4
C(10)-C(12)-H(12B)	109.4
C(13)-C(12)-H(12B)	109.4
H(12A)-C(12)-H(12B)	108.0
N(14)-C(13)-C(12)	111.94(16)
N(14)-C(13)-H(13A)	109.2
C(12)-C(13)-H(13A)	109.2
N(14)-C(13)-H(13B)	109.2
C(12)-C(13)-H(13B)	109.2
H(13A)-C(13)-H(13B)	107.9
C(7)-N(14)-C(13)	119.88(14)
C(7)-N(14)-C(16)	108.53(12)
C(13)-N(14)-C(16)	115.10(14)
C(6)-C(15)-C(16)	96.80(13)
C(6)-C(15)-H(15A)	112.4
C(16)-C(15)-H(15A)	112.4
C(6)-C(15)-H(15B)	112.4
C(16)-C(15)-H(15B)	112.4
H(15A)-C(15)-H(15B)	110.0
N(14)-C(16)-C(17)	112.68(15)
N(14)-C(16)-C(15)	101.01(12)
C(17)-C(16)-C(15)	109.55(15)
N(14)-C(16)-H(16)	107.9(12)
C(17)-C(16)-H(16)	113.0(13)
C(15)-C(16)-H(16)	112.1(12)
C(18)-C(17)-C(16)	120.49(17)
C(18)-C(17)-H(17)	119.8
C(16)-C(17)-H(17)	119.8
C(17)-C(18)-C(5)	118.01(16)
C(17)-C(18)-H(18)	121.0
C(5)-C(18)-H(18)	121.0

Symmetry transformations used to generate equivalent atoms:

Supplementary Table 19. Anisotropic displacement parameters ($Å^2 \times 10^3$) for securingine D (**9b**). The anisotropic displacement factor exponent takes the form: $-2\pi^2[h^2a^{*2}U^{11} + ... + 2 h k a^* b^* U^{12}]$.

	U^{11}	U^{22}	U^{33}	U^{23}	U^{13}	U^{12}
O(1)	35(1)	34(1)	37(1)	1(1)	7(1)	-2(1)
O(2)	41(1)	50(1)	71(1)	10(1)	12(1)	-5(1)
C(3)	32(1)	38(1)	50(1)	13(1)	11(1)	6(1)
C(4)	45(1)	49(1)	38(1)	10(1)	15(1)	10(1)
C(5)	42(1)	40(1)	26(1)	5(1)	6(1)	13(1)
C(6)	31(1)	31(1)	27(1)	0(1)	3(1)	4(1)
C(7)	30(1)	30(1)	24(1)	-1(1)	2(1)	2(1)
O(8)	35(1)	43(1)	23(1)	-2(1)	0(1)	-3(1)
C(9)	39(1)	39(1)	31(1)	1(1)	2(1)	9(1)
C(10)	65(1)	35(1)	43(1)	0(1)	3(1)	16(1)
O(11)	70(1)	47(1)	37(1)	-7(1)	10(1)	17(1)
C(12)	76(2)	28(1)	58(1)	0(1)	7(1)	-3(1)
C(13)	53(1)	40(1)	55(1)	-11(1)	-3(1)	-10(1)
N(14)	33(1)	33(1)	31(1)	-1(1)	-2(1)	-2(1)
C(15)	33(1)	38(1)	34(1)	3(1)	2(1)	9(1)
C(16)	32(1)	49(1)	32(1)	0(1)	-4(1)	4(1)
C(17)	52(1)	59(1)	31(1)	-4(1)	-12(1)	3(1)
C(18)	58(1)	55(1)	24(1)	-1(1)	-1(1)	12(1)

Supplementary Table 20. Hydrogen coordinates (x 10^4) and isotropic displacement parameters ($Å^2$ x 10^3) for securingine D (**9b**).

	x	y	z	U(eq)
H(4)	5117	9234	2583	52
H(8)	3330(40)	7850(30)	5064(12)	51
H(9)	4900(40)	6320(30)	4228(11)	43
H(10)	4330(40)	3470(30)	4376(12)	58
H(12A)	1030	3847	4888	65
H(12B)	1038	2571	4288	65
H(13A)	-56	4524	3525	59
H(13B)	-1456	4478	4151	59
H(15A)	-147	10090	3481	42
H(15B)	-326	9616	4266	42
H(16)	-2220(30)	7570(30)	3705(11)	45
H(17)	-1124	6702	2600	57
H(18)	1859	7250	2208	54

Supplementary Table 21. Hydrogen bonds for securingine D (**9b**) [Å and °].

D-H...A	d(D-H)	d(H...A)	d(D...A)	<(DHA)
O(8)-H(8)...N(14)#1	0.90(3)	2.10(3)	2.8730(18)	144(2)

Symmetry transformations used to generate equivalent atoms:
#1 x+1/2,-y+3/2,-z+1

8. Copies of NMR spectra of newly synthesized compounds

Supplementary Figure 23. ^{1}H NMR spectrum of **18** (500MHz, $CDCl_3$)

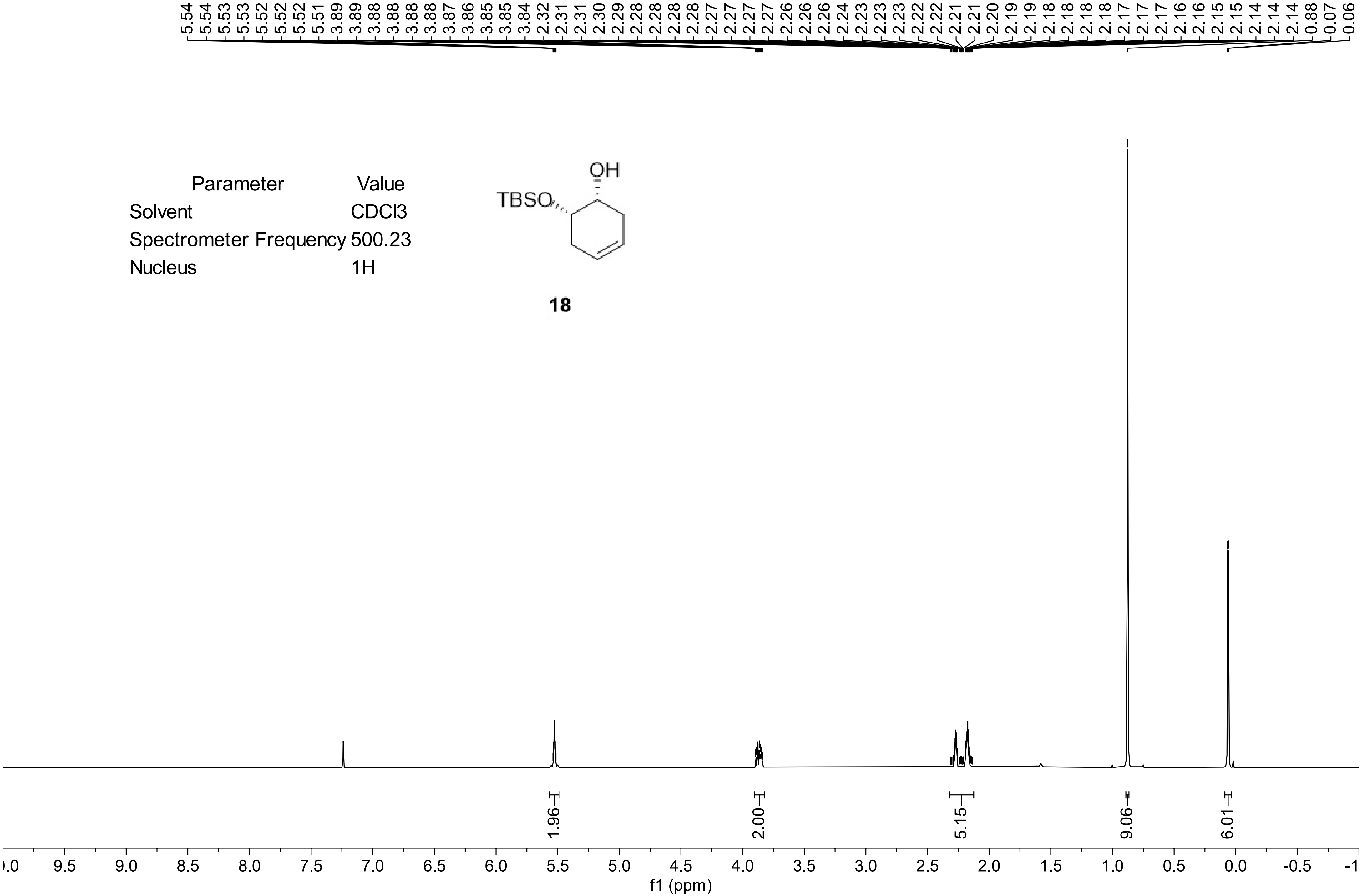

Supplementary Figure 24. ^{13}C NMR spectrum of **18** (126MHz, $CDCl_3$)

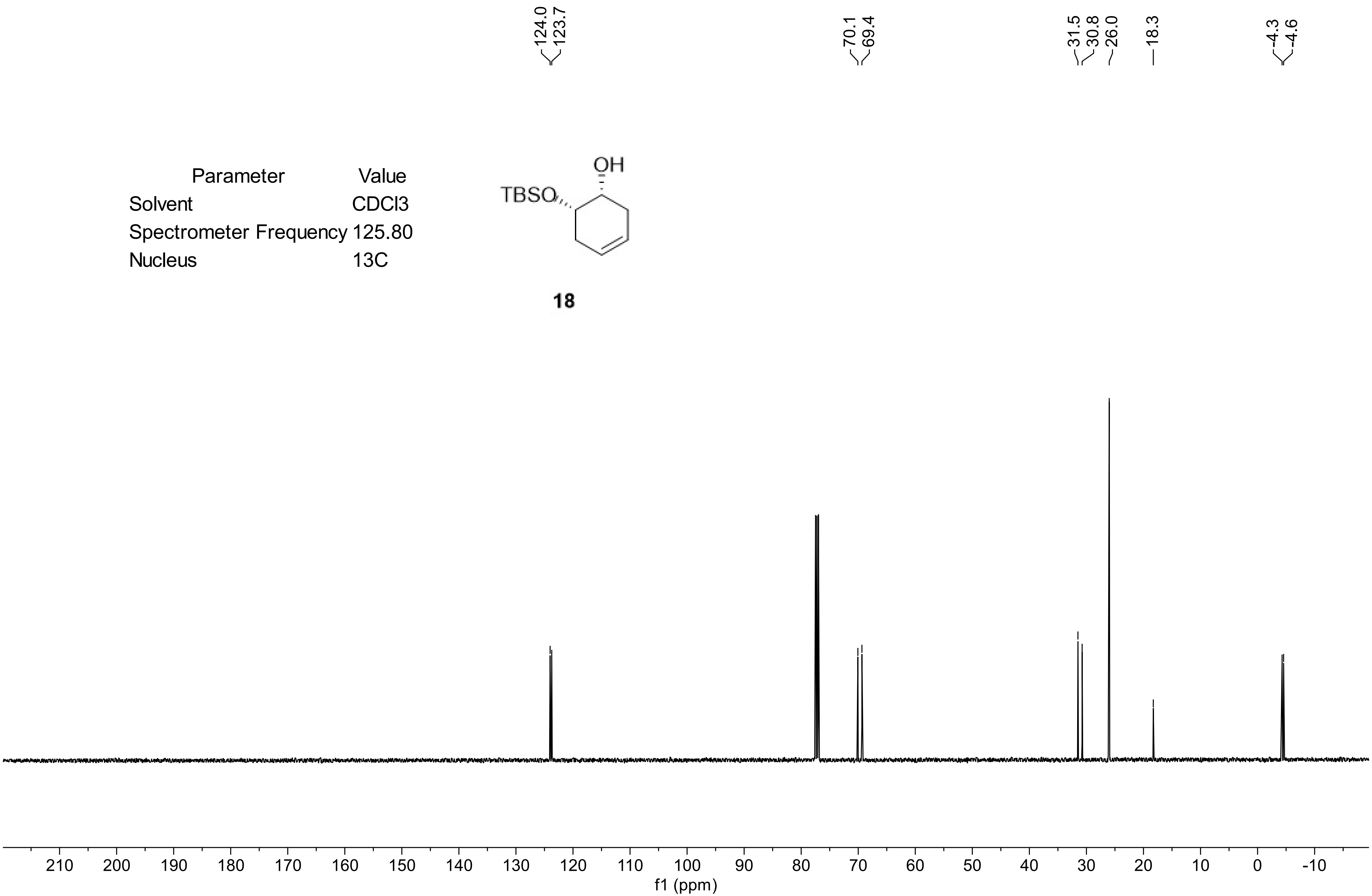

Supplementary Figure 25. ^{1}H NMR spectrum of **S1** (500MHz, $CDCl_3$)

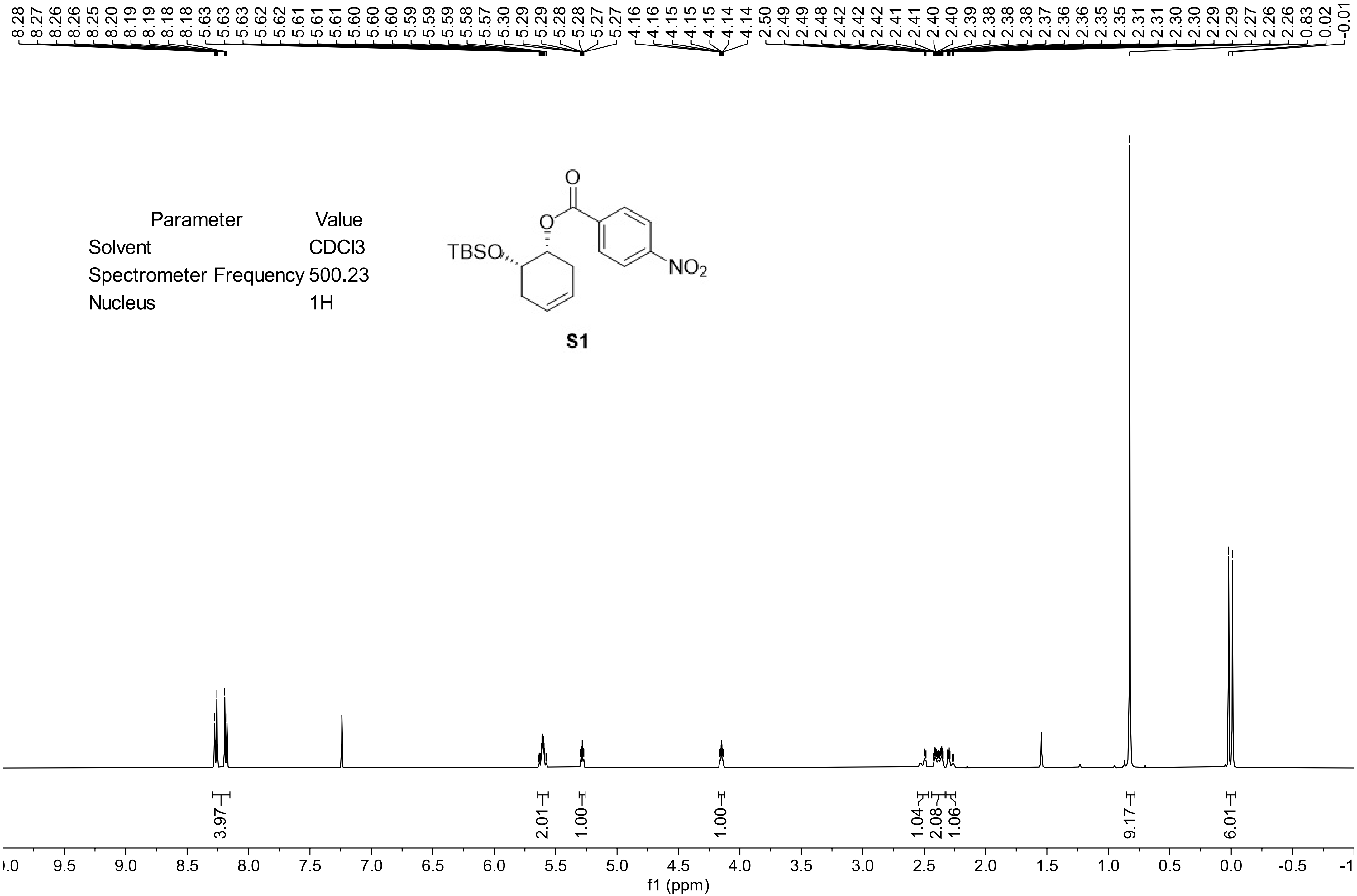

Supplementary Figure 26. ^{13}C NMR spectrum of **S1** (126MHz, $CDCl_3$)

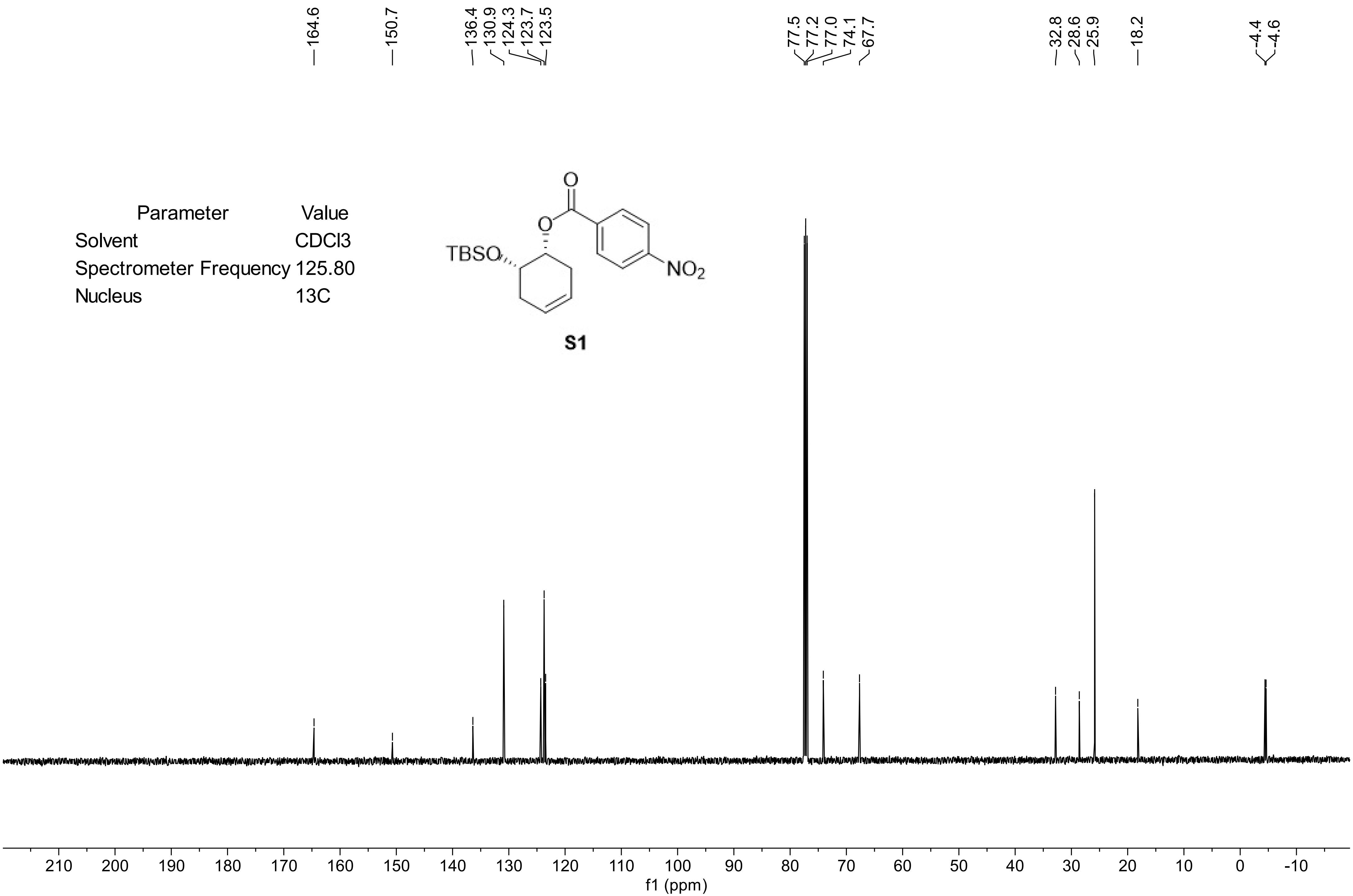

Supplementary Figure 27. ^{1}H NMR spectrum of **19** (500MHz, $CDCl_3$)

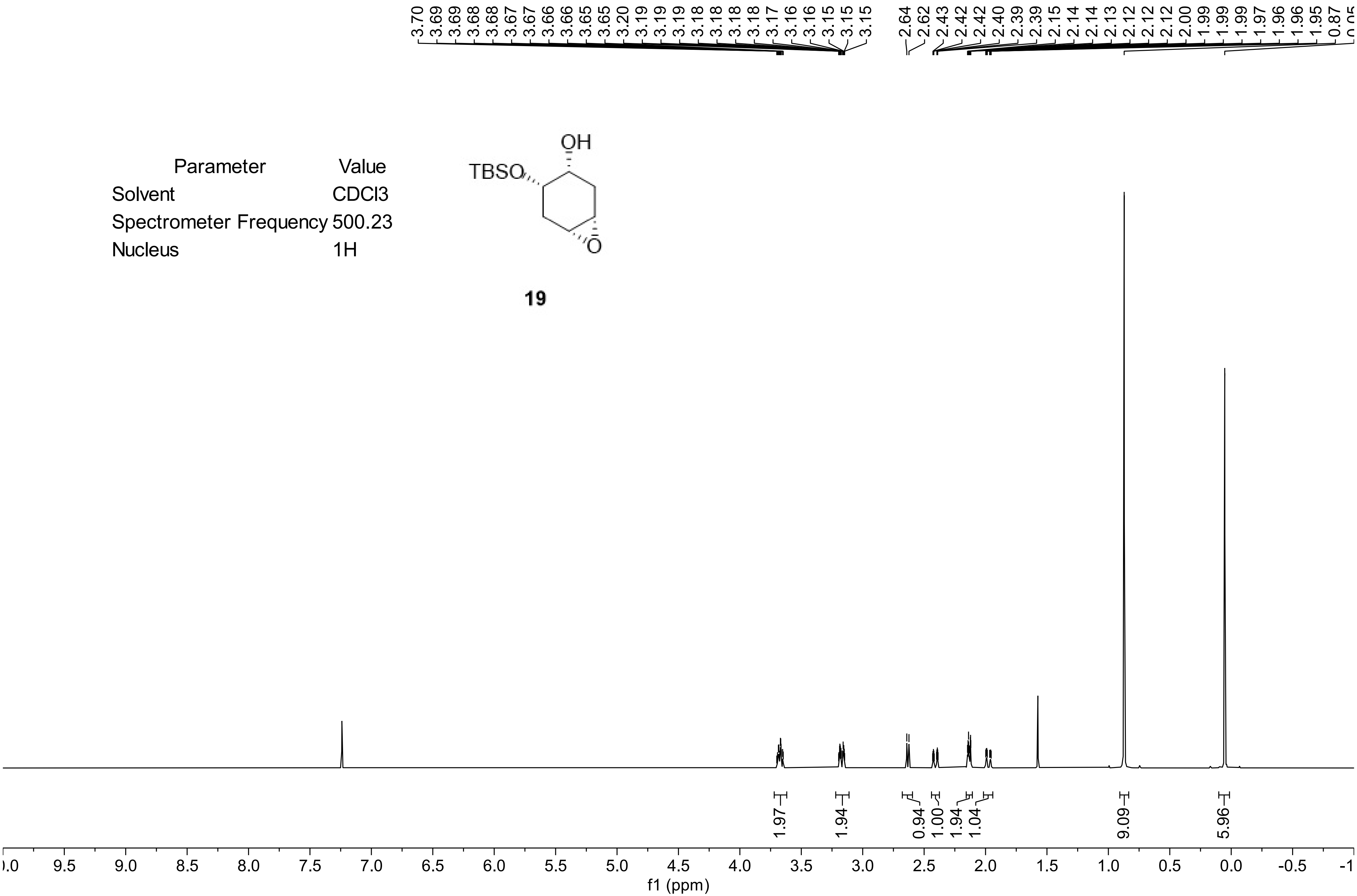

Supplementary Figure 28. ^{13}C NMR spectrum of **19** (126MHz, $CDCl_3$)

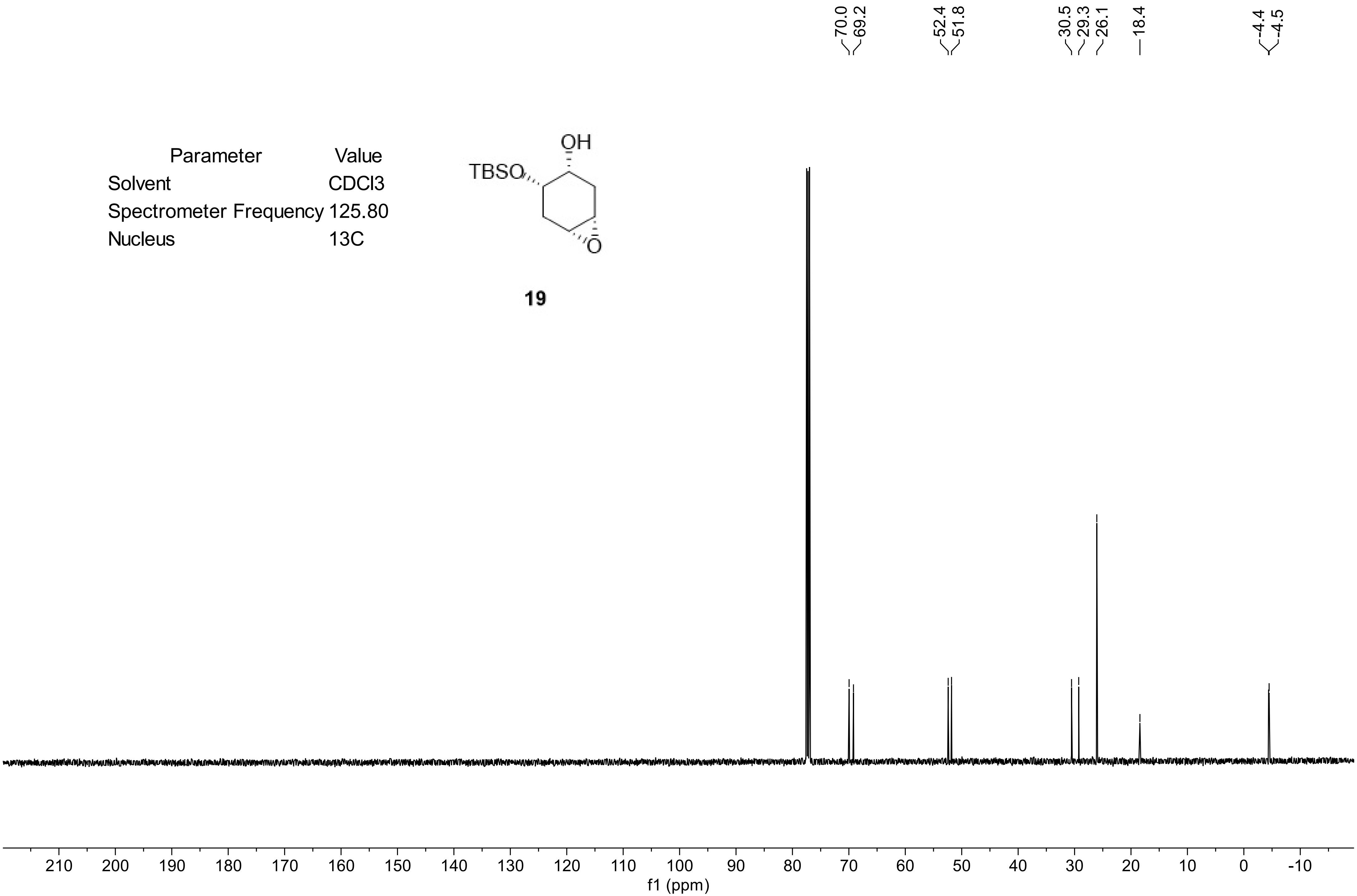

Supplementary Figure 29. ^{1}H NMR spectrum of **20** (500MHz, $CDCl_3$)

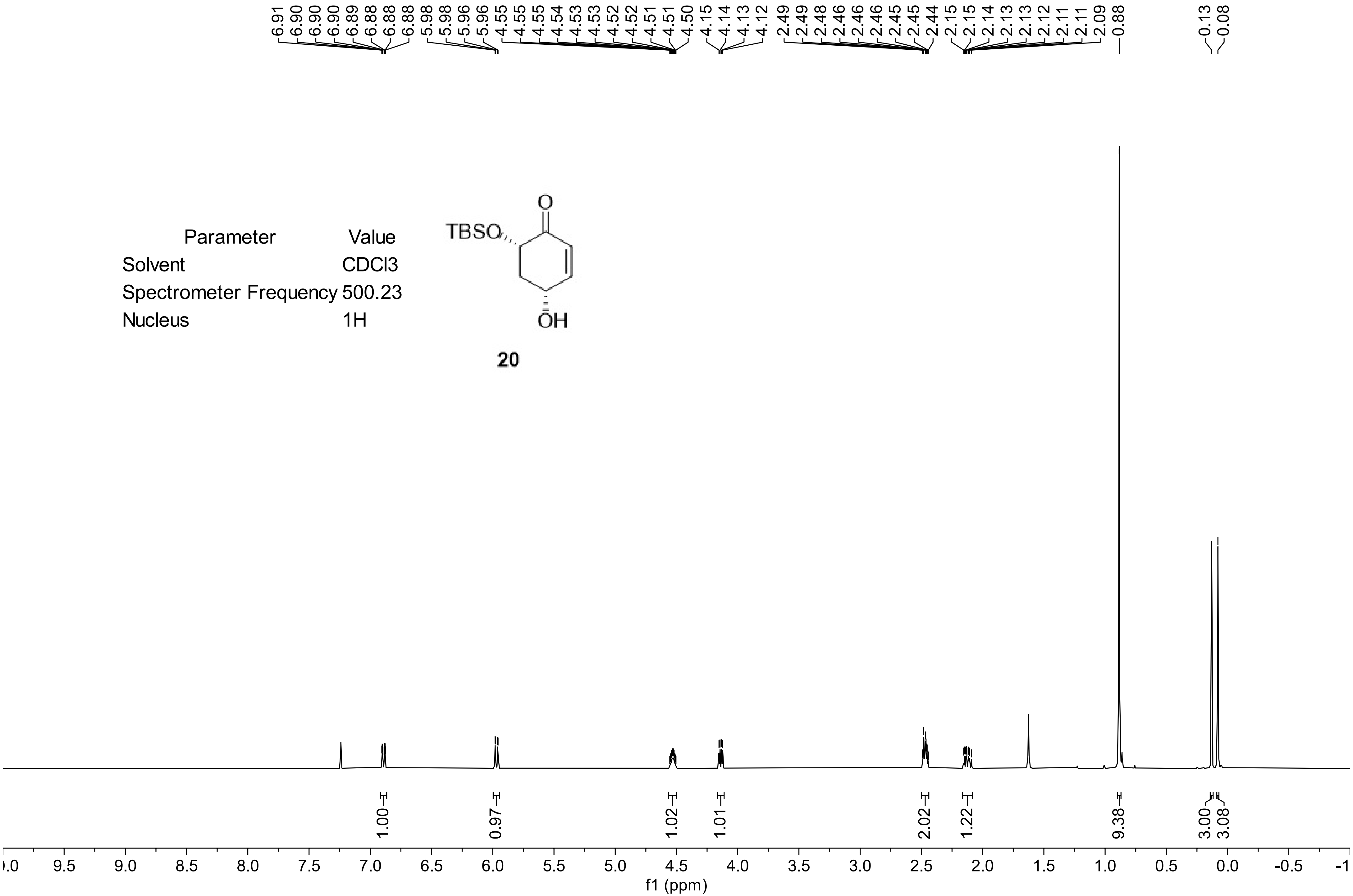

Supplementary Figure 30. ^{13}C NMR spectrum of **20** (126MHz, $CDCl_3$)

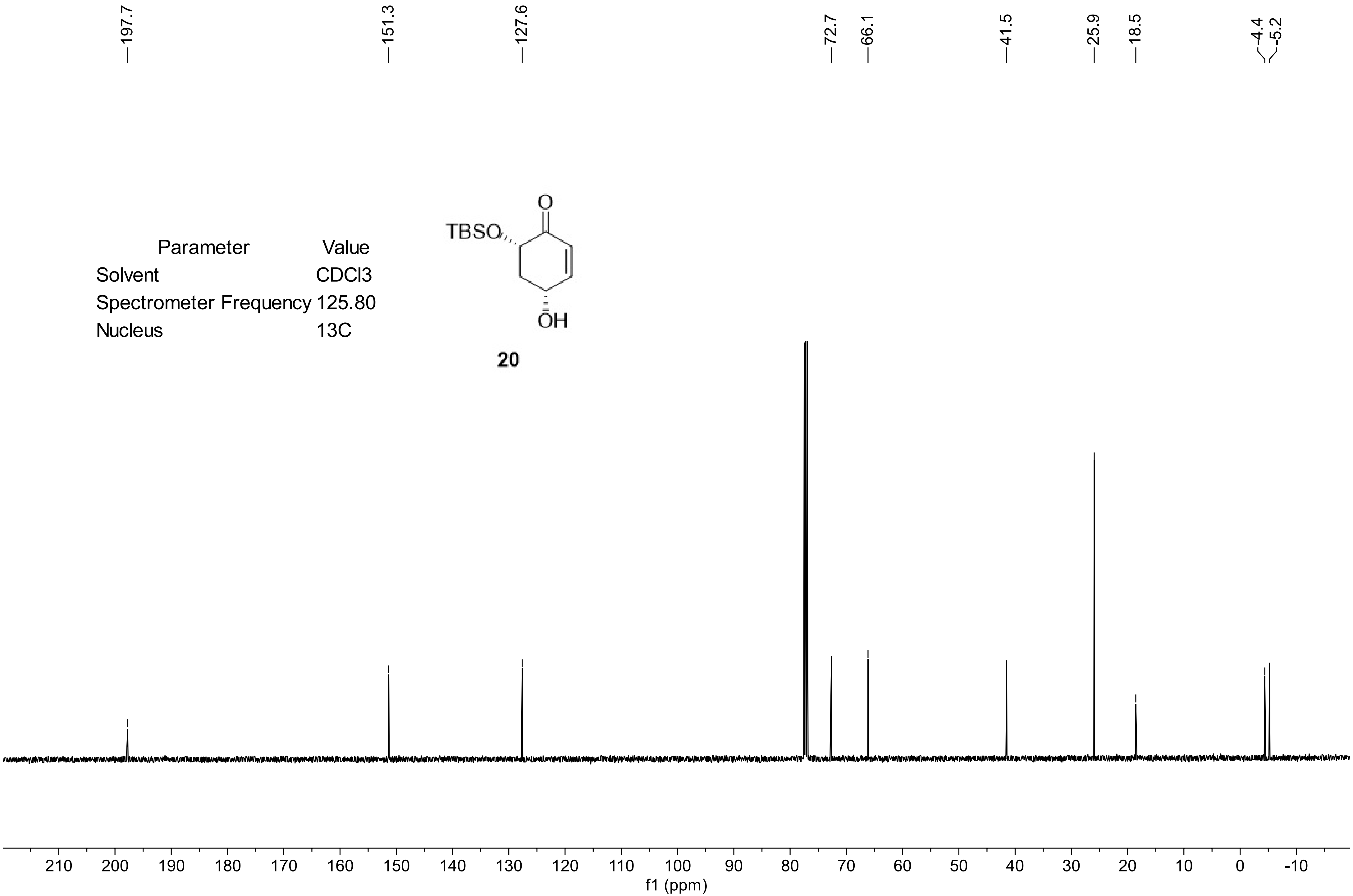

Supplementary Figure 31. ^{1}H NMR spectrum of **S2** (500MHz, $CDCl_3$)

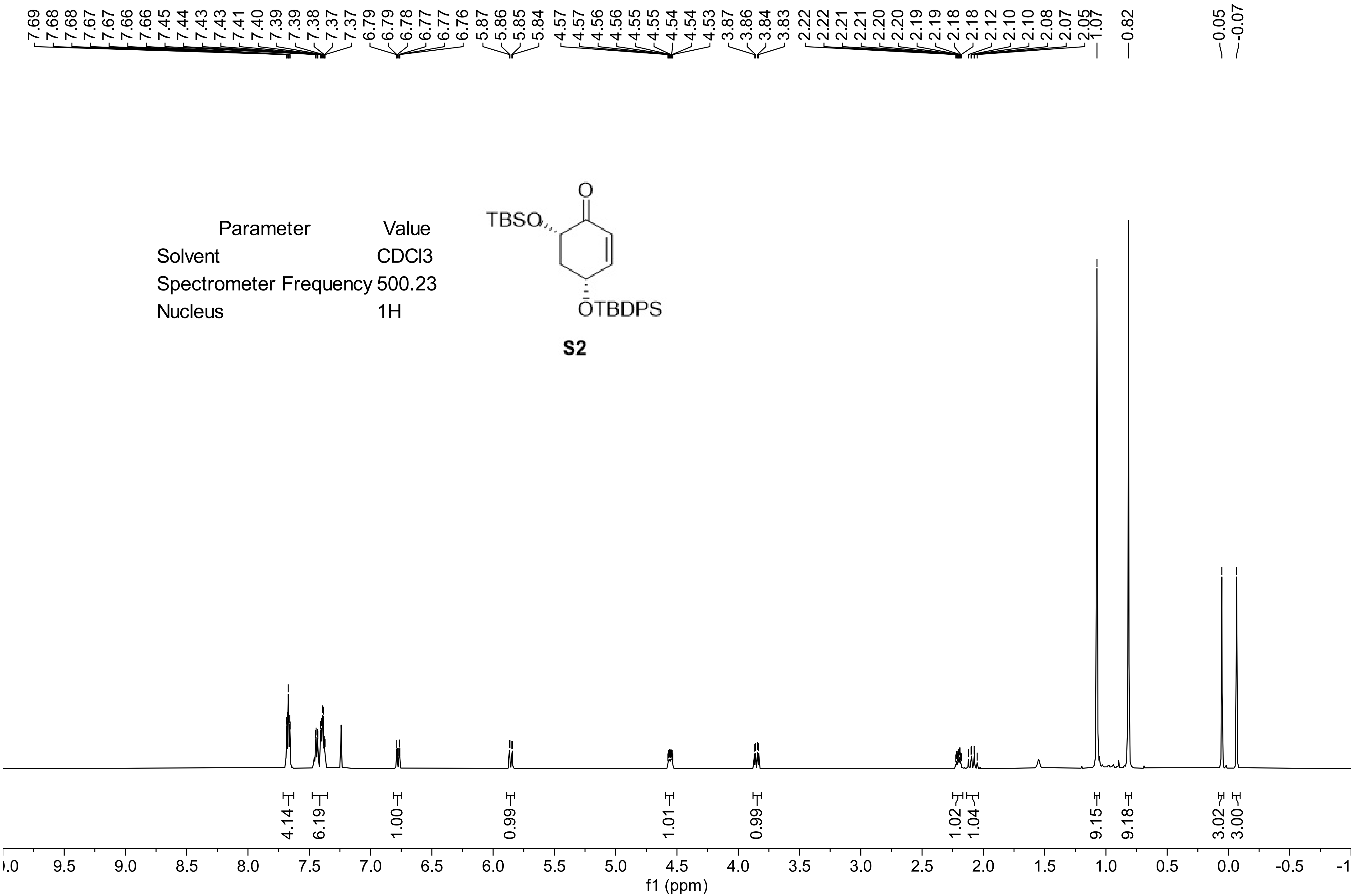

Supplementary Figure 32. ^{13}C NMR spectrum of **S2** (126MHz, $CDCl_3$)

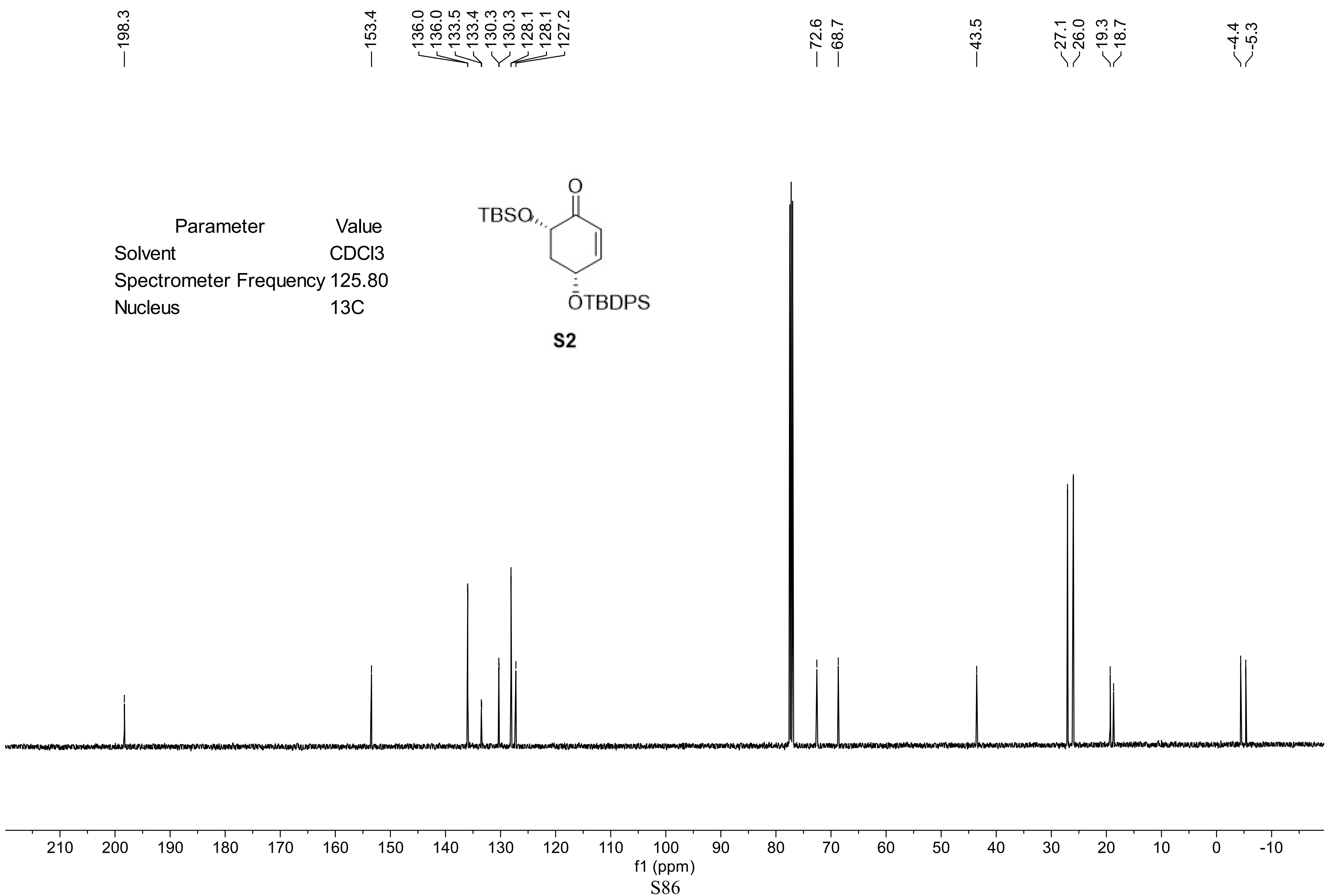

Supplementary Figure 33. ^{1}H NMR spectrum of **21** (500MHz, $CDCl_3$)

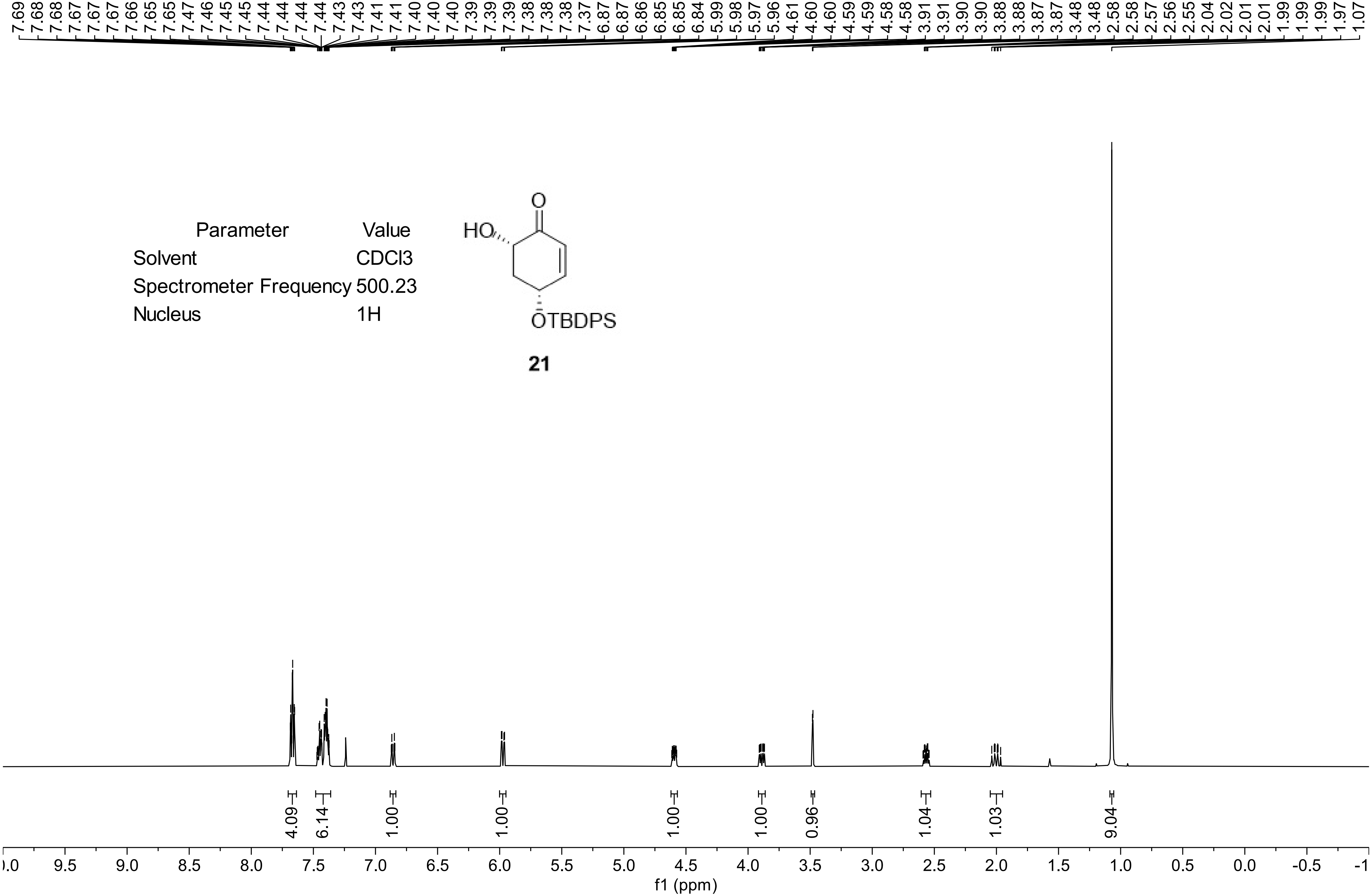

Supplementary Figure 34. ^{13}C NMR spectrum of **21** (126MHz, $CDCl_3$)

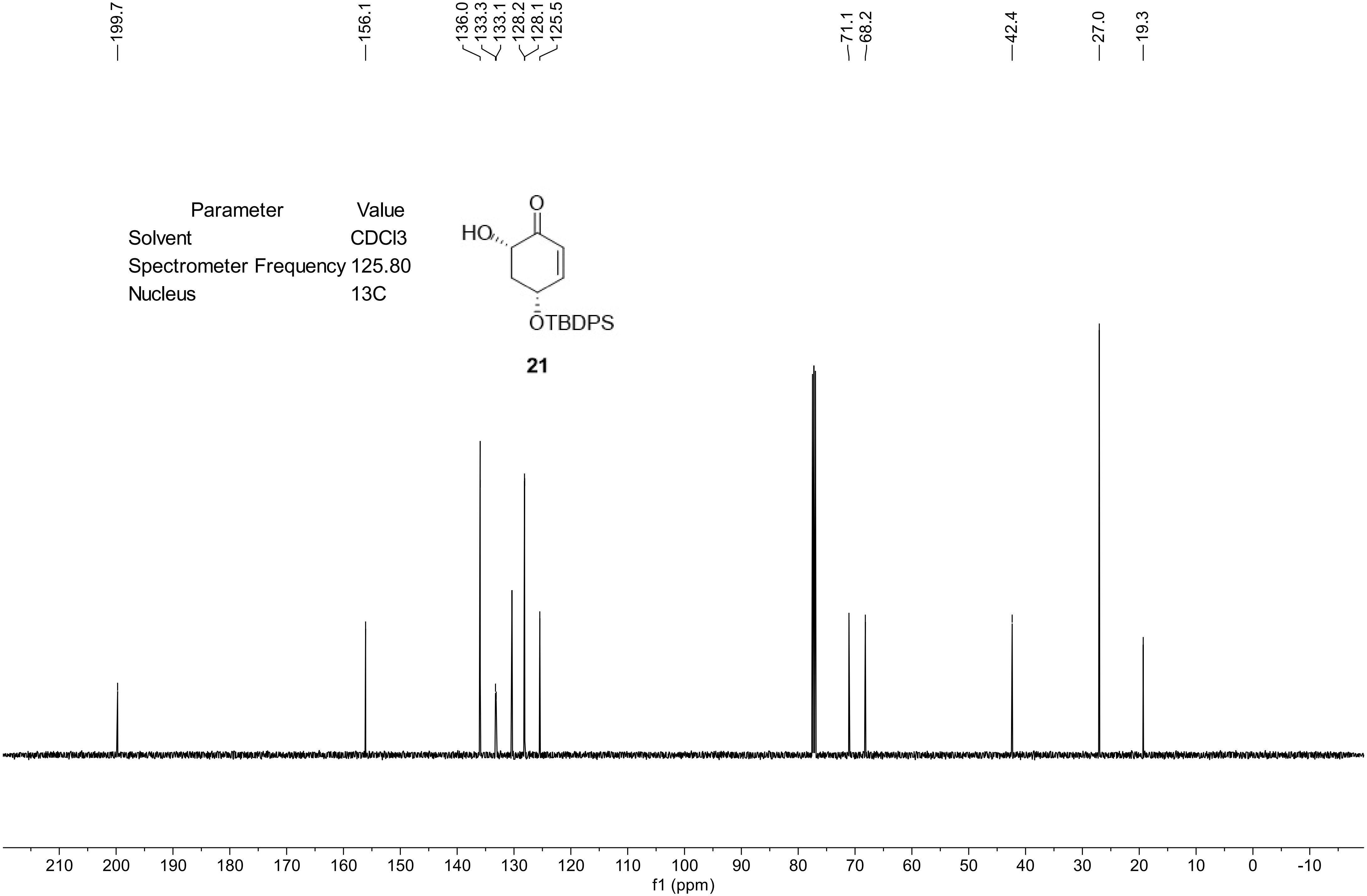

Supplementary Figure 35. ^{1}H NMR spectrum of **22** (500MHz, $CDCl_3$)

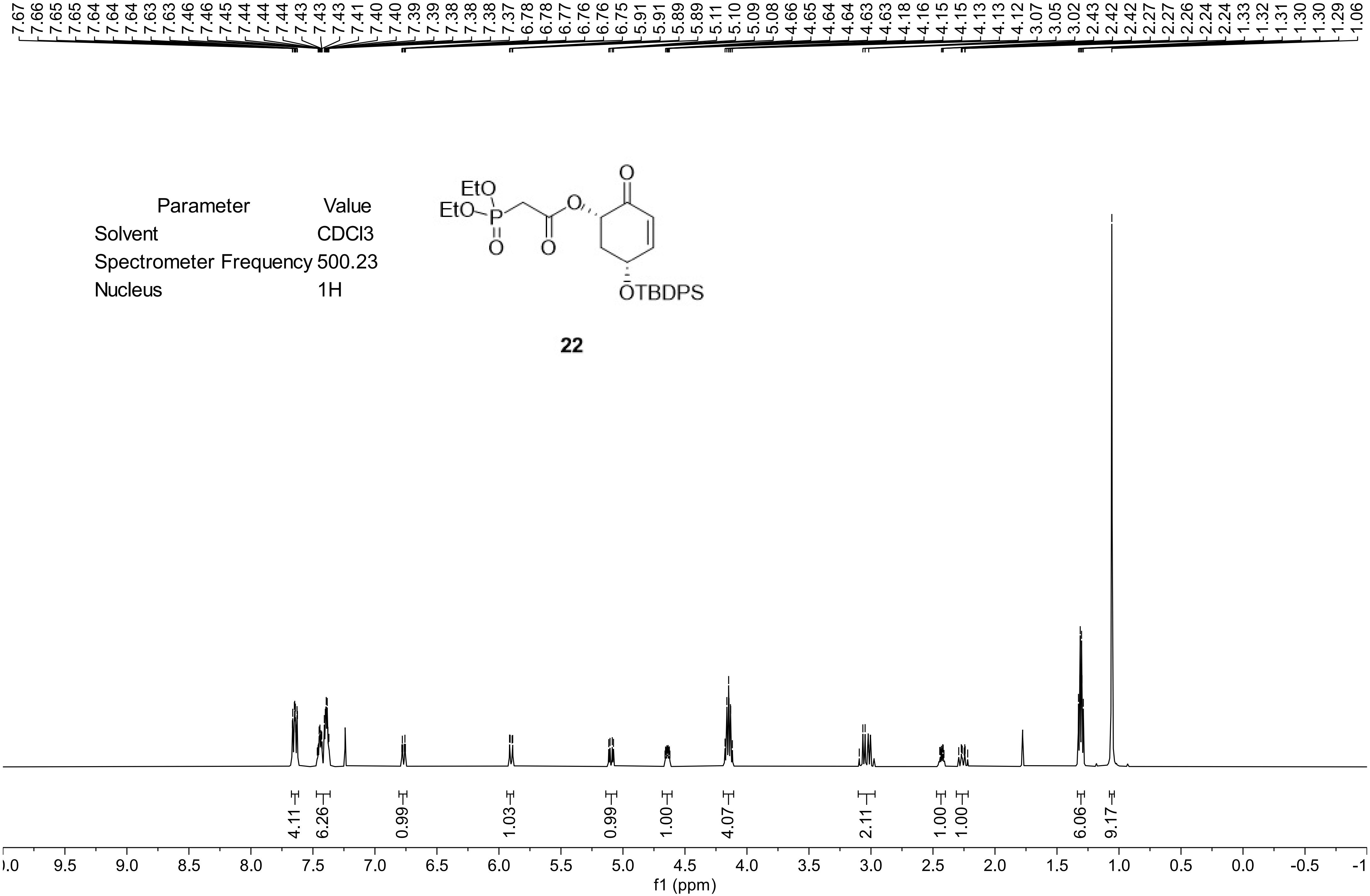

Supplementary Figure 36. ^{13}C NMR spectrum of **22** (126MHz, $CDCl_3$)

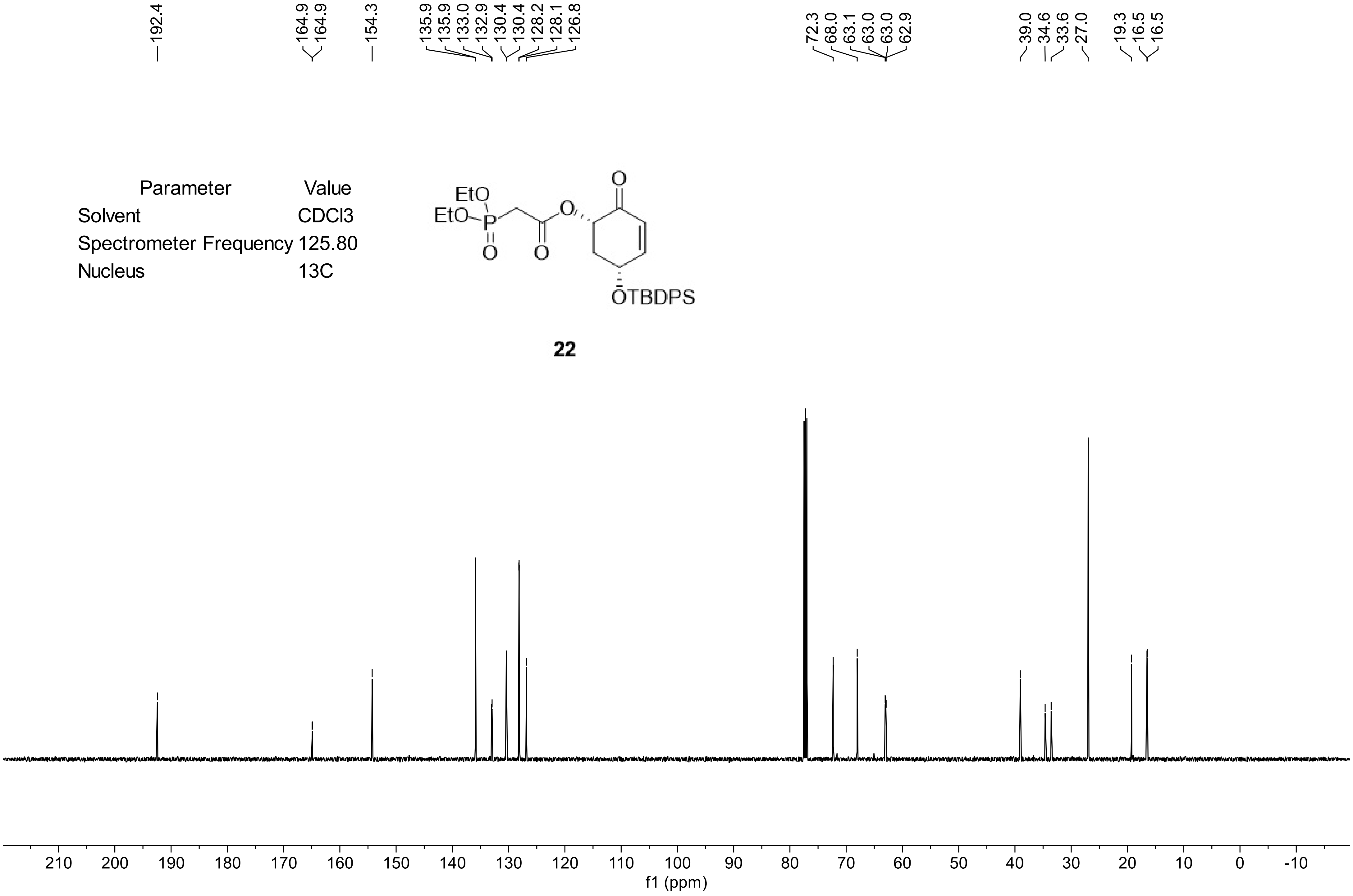

Supplementary Figure 37. ^{1}H NMR spectrum of **23** (500MHz, $CDCl_3$)

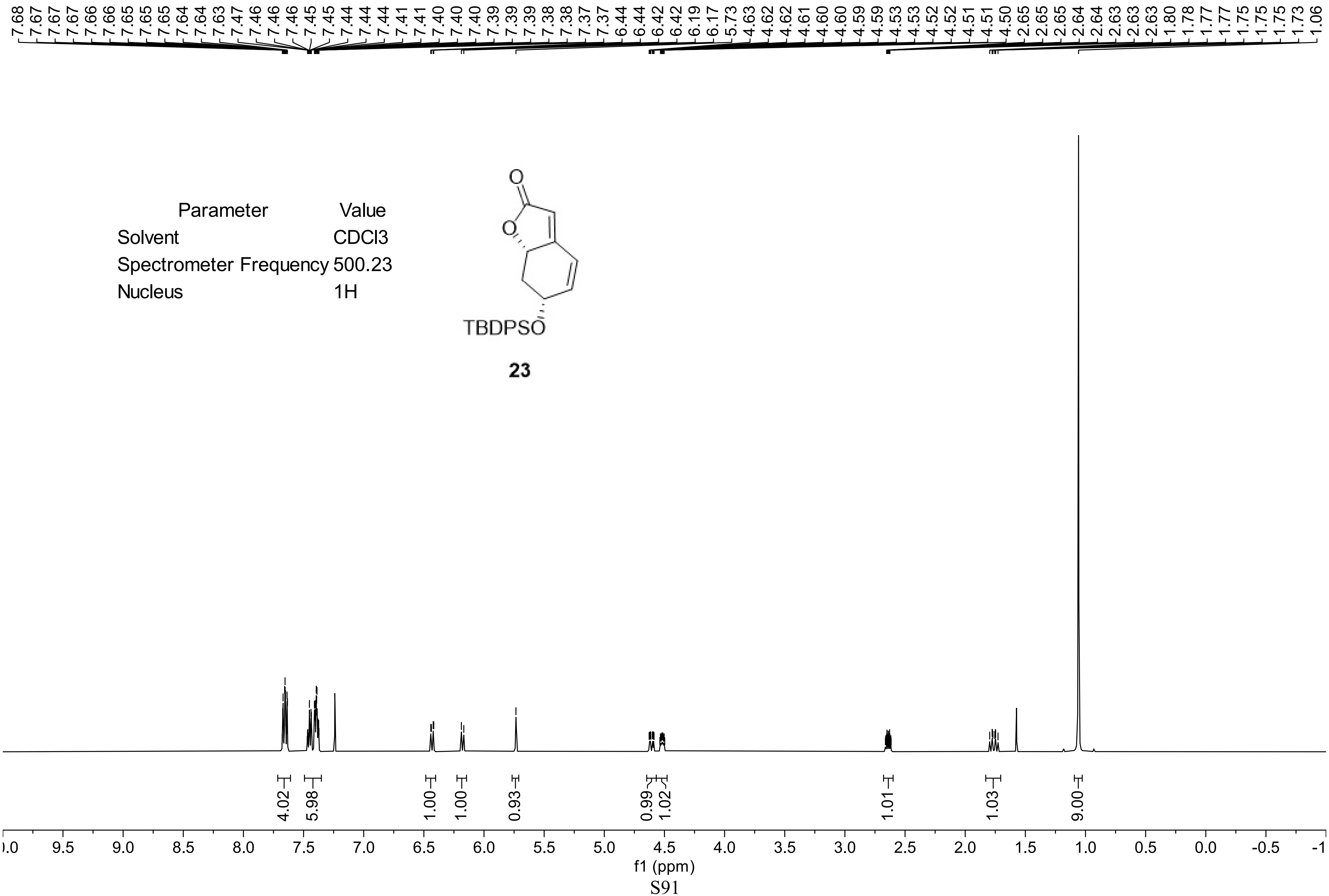

Supplementary Figure 38. ^{13}C NMR spectrum of **23** (126MHz, $CDCl_3$)

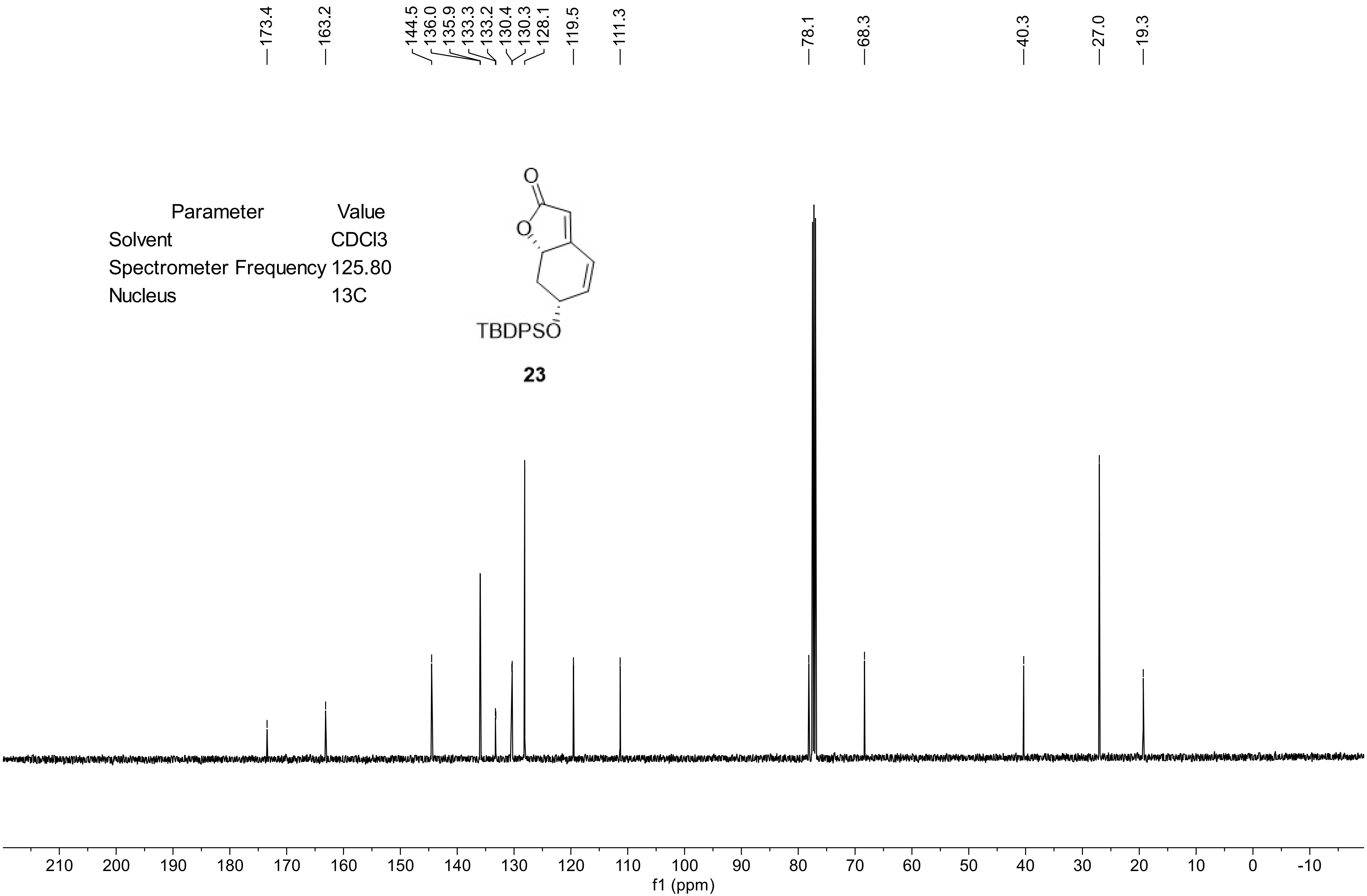

Supplementary Figure 39. ^{1}H NMR spectrum of **26** (400MHz, $CDCl_3$)

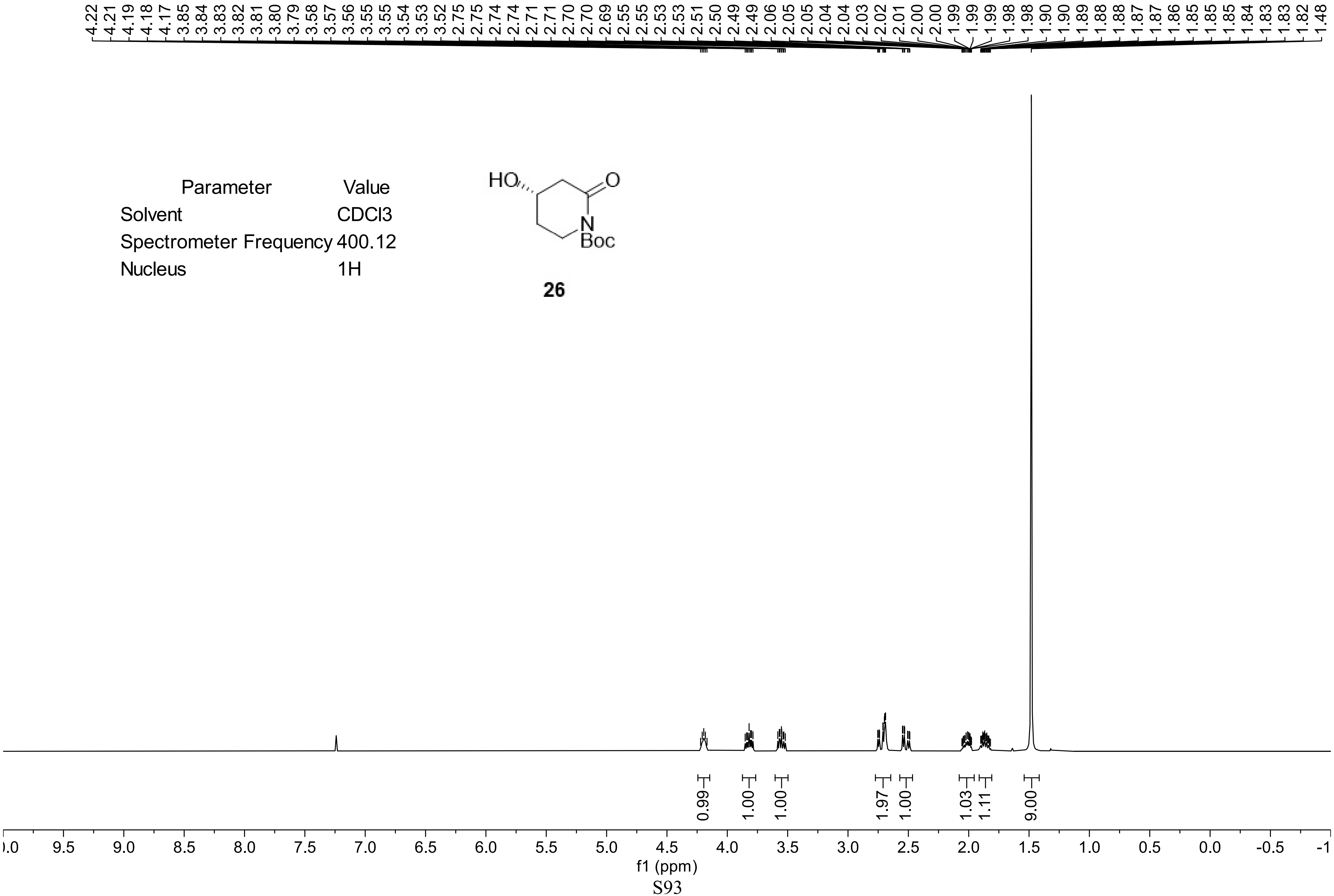

Supplementary Figure 40. ^{13}C NMR spectrum of **26** (101MHz, $CDCl_3$)

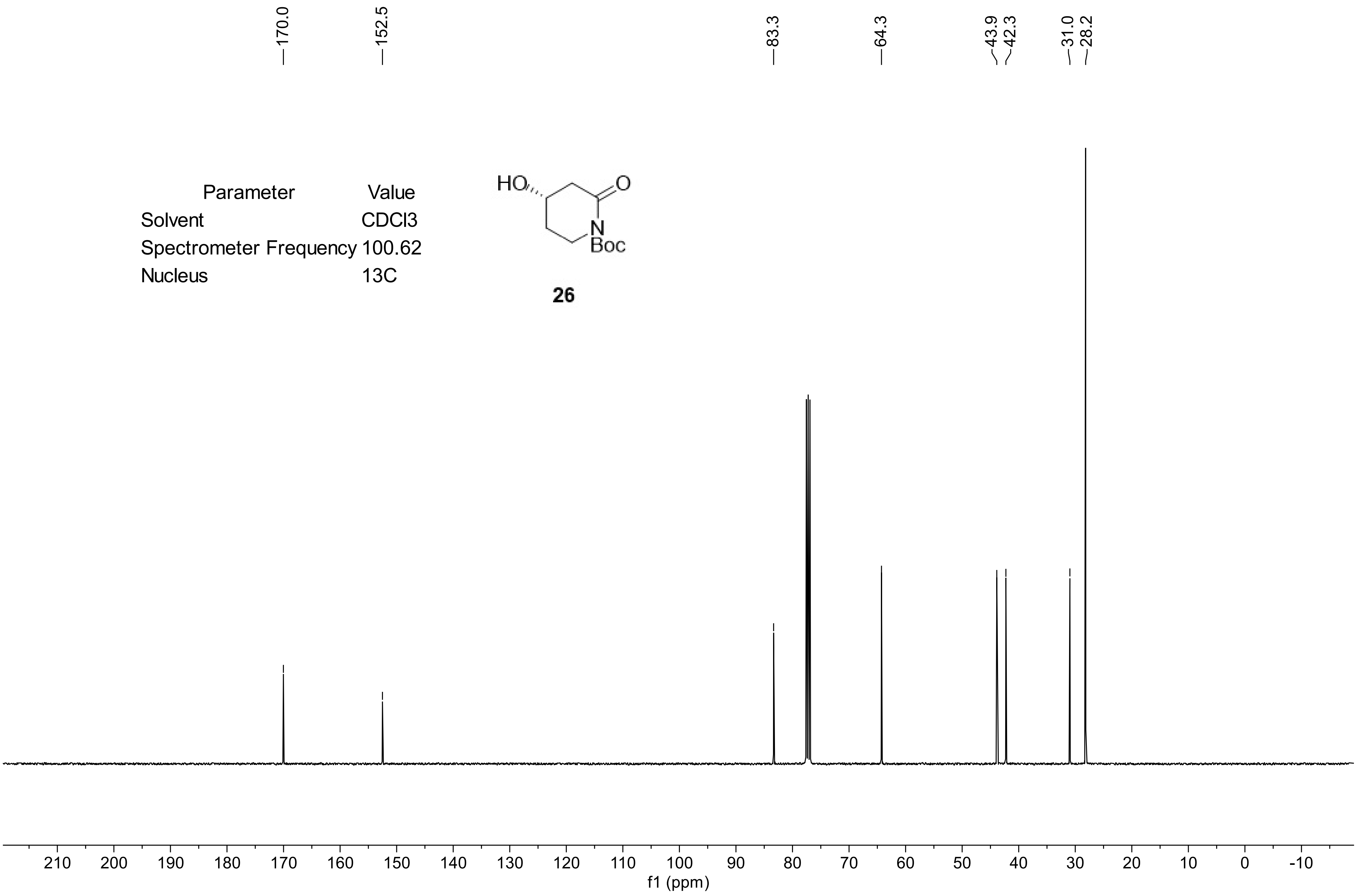

Supplementary Figure 41. ^{1}H NMR spectrum of **27** (400MHz, $CDCl_3$)

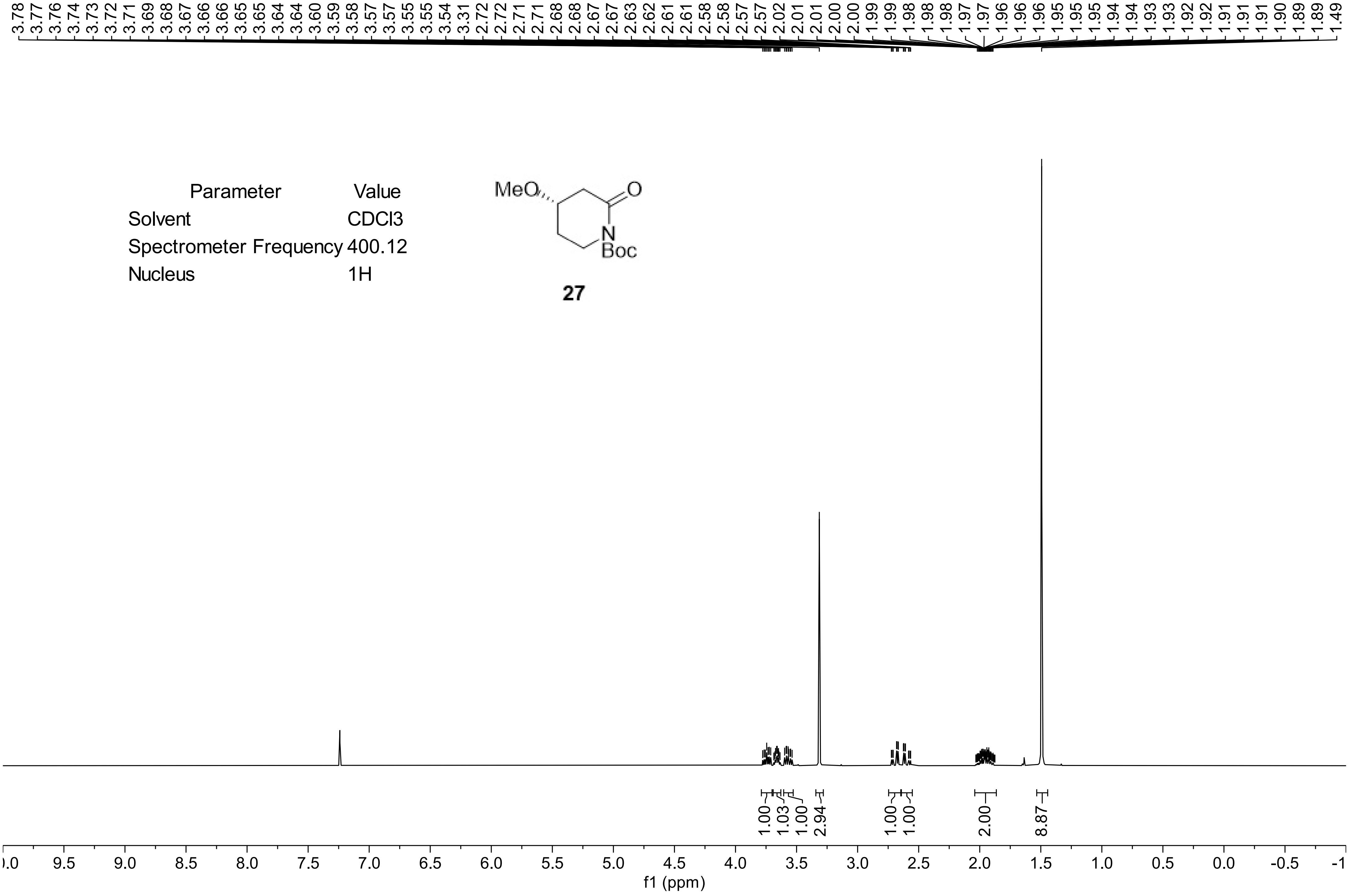

Supplementary Figure 42. ^{13}C NMR spectrum of **27** (101MHz, $CDCl_3$)

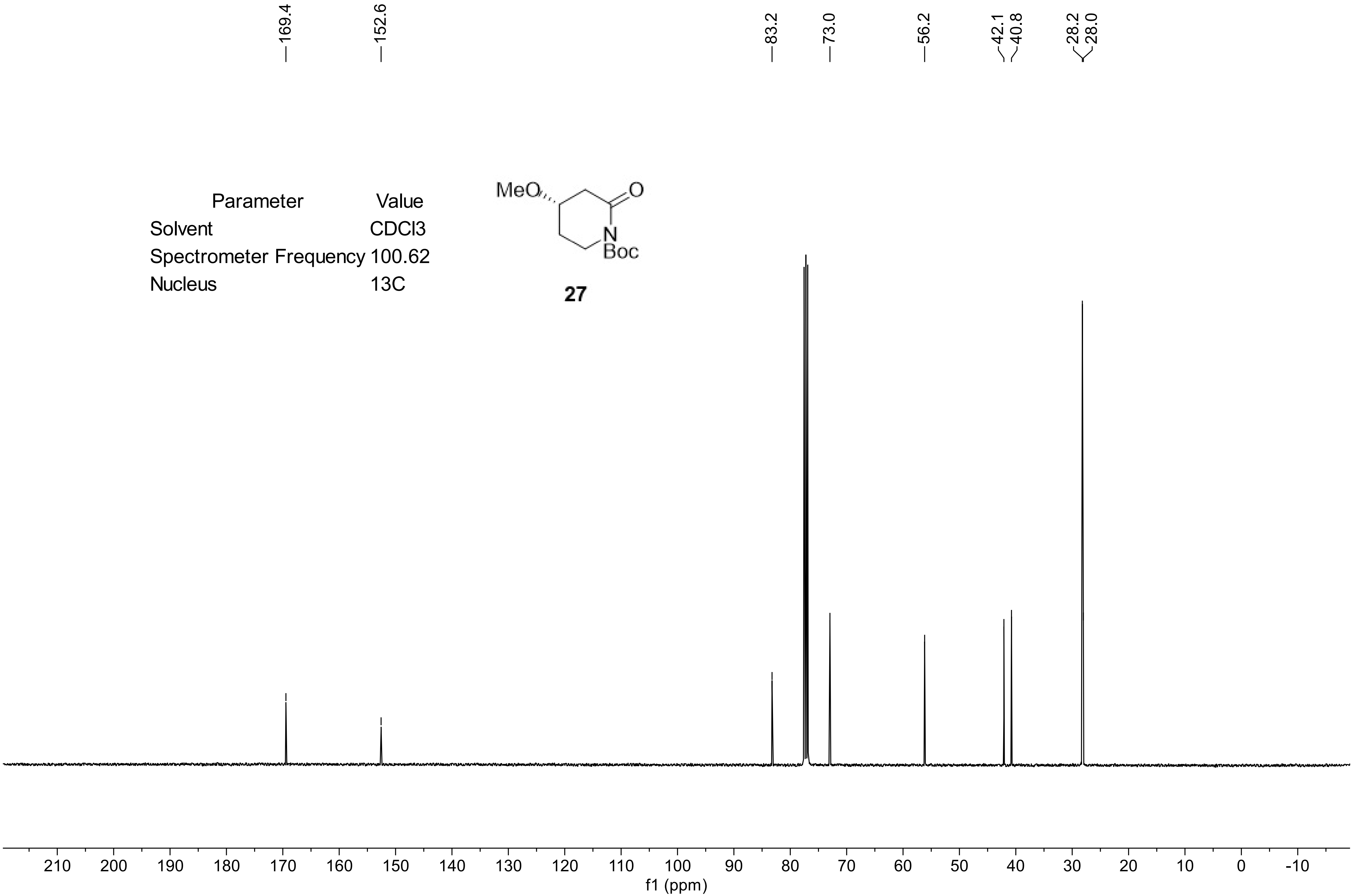

Supplementary Figure 43. ^{1}H NMR spectrum of **29** (400MHz, $CDCl_3$)

Parameter	Value
Solvent	CDCl3
Spectrometer Frequency	400.12
Nucleus	1H

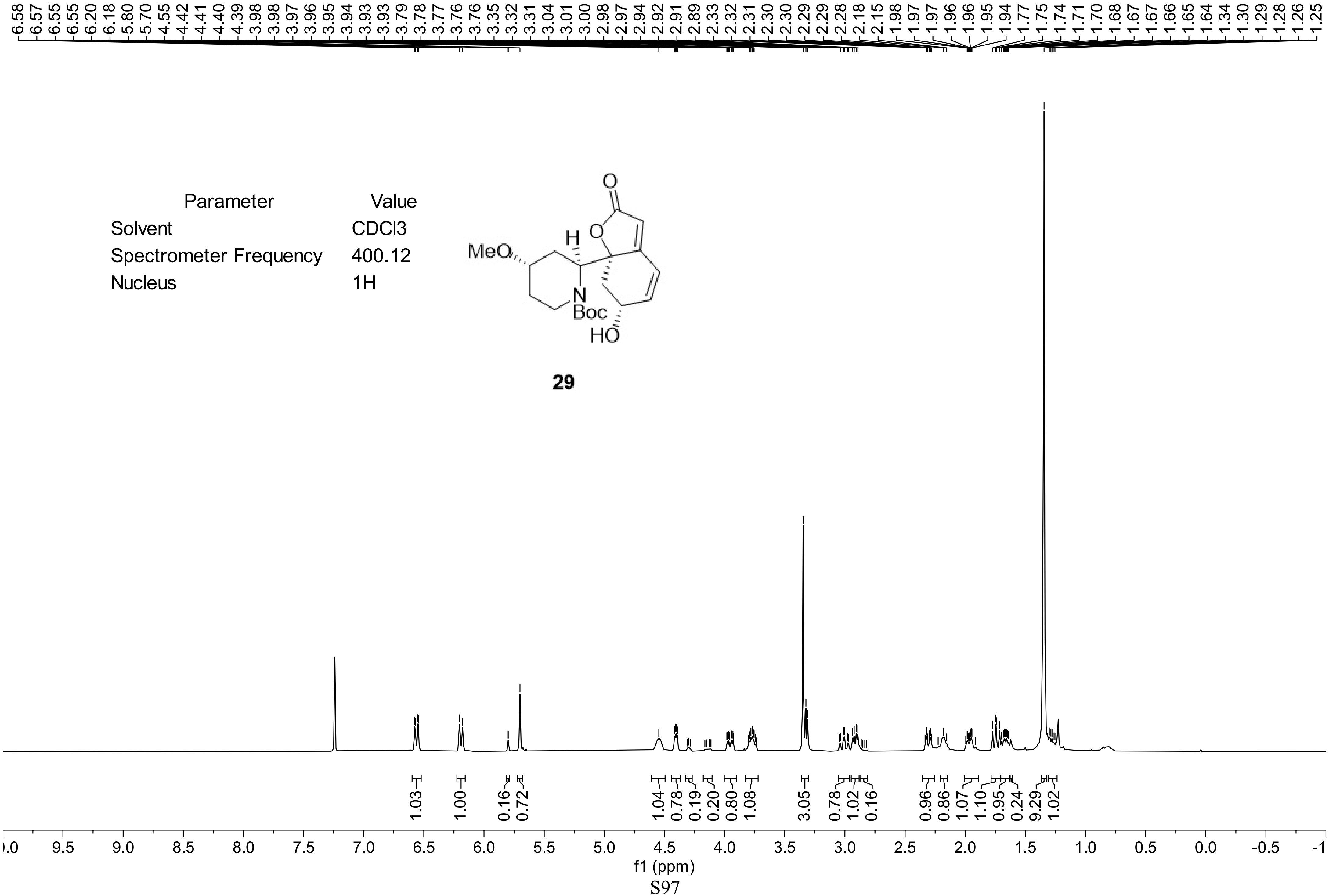

Supplementary Figure 44. ^{13}C NMR spectrum of **29** (101MHz, $CDCl_3$)

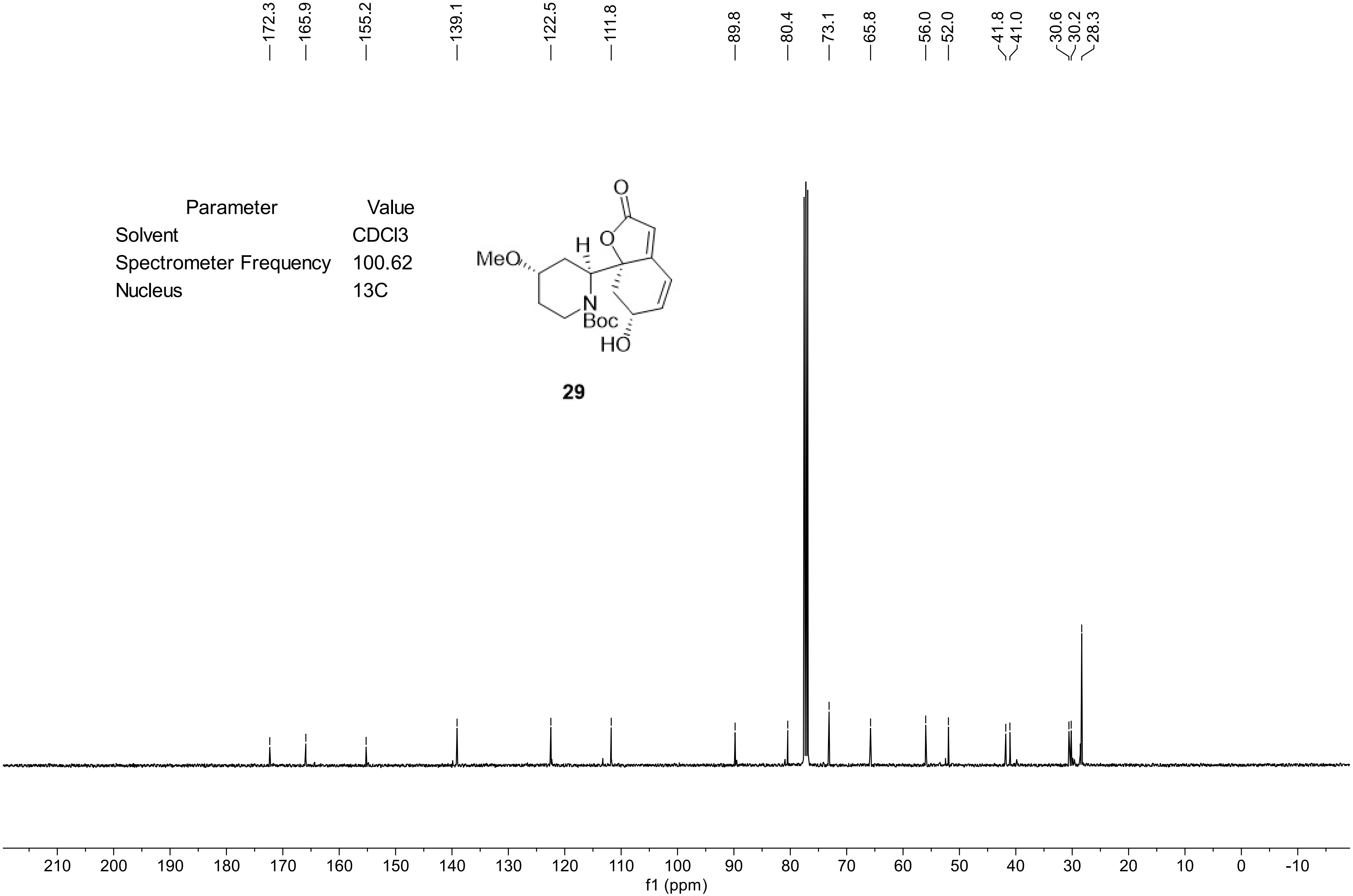

Supplementary Figure 45. NOESY NMR spectrum of **29** (400MHz, $CDCl_3$)

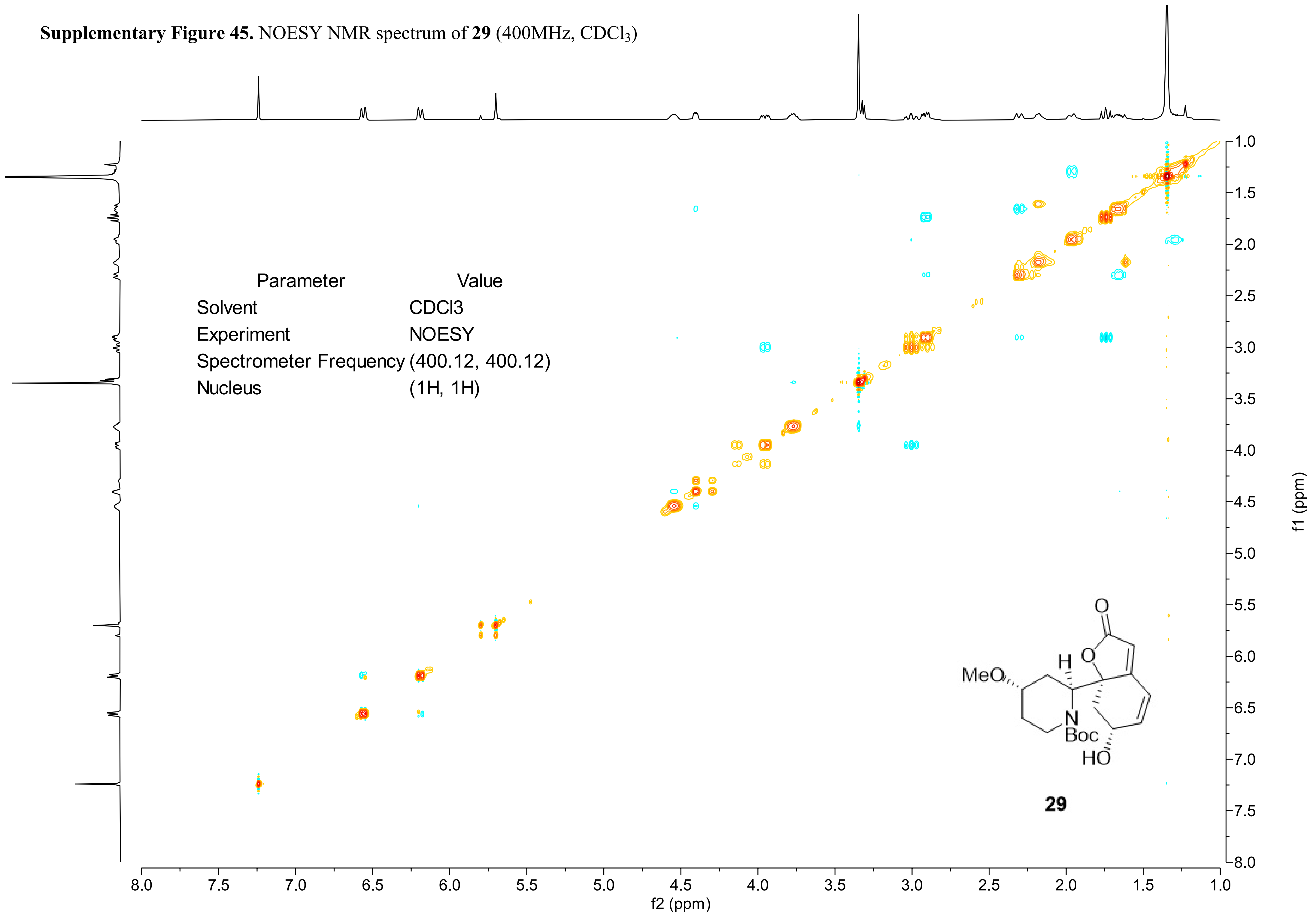

Supplementary Figure 46. ^{1}H NMR spectrum of **30** (500MHz, $CDCl_3$)

Parameter	Value
Solvent	CDCl3
Spectrometer Frequency	500.23
Nucleus	1H

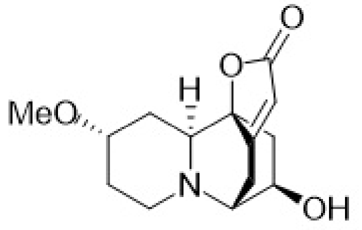

30

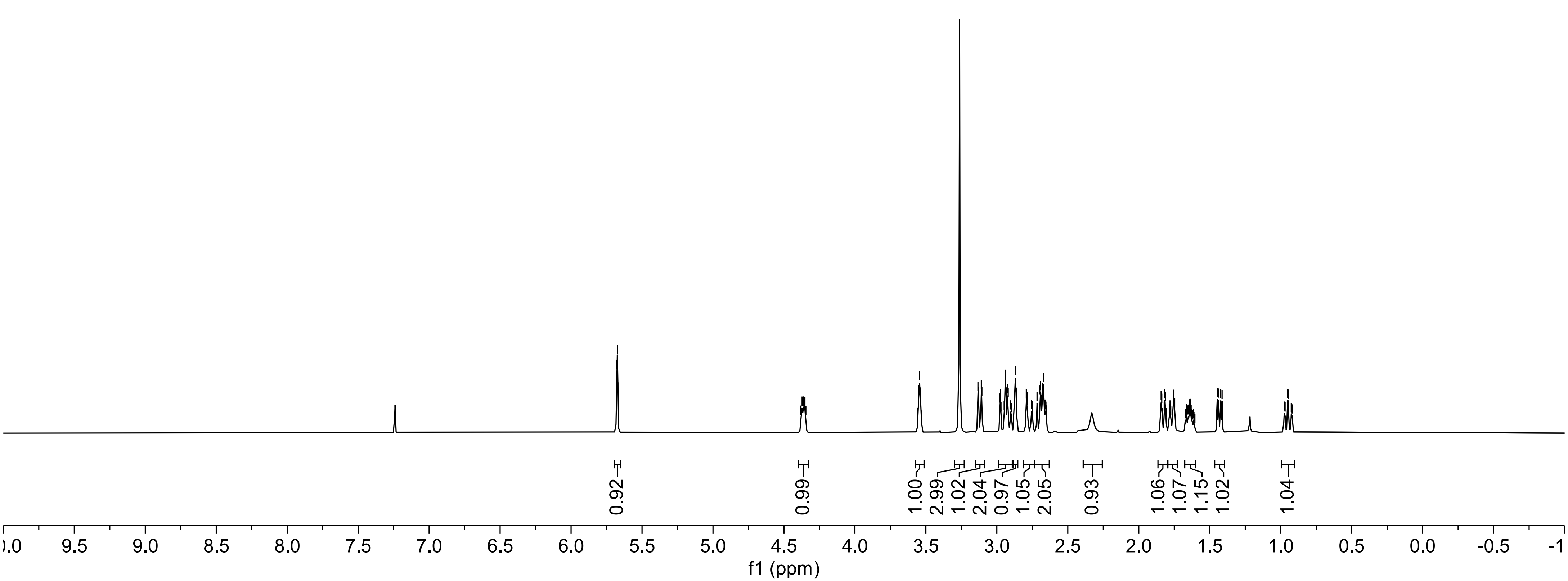

Supplementary Figure 47. ^{13}C NMR spectrum of **30** (126MHz, $CDCl_3$)

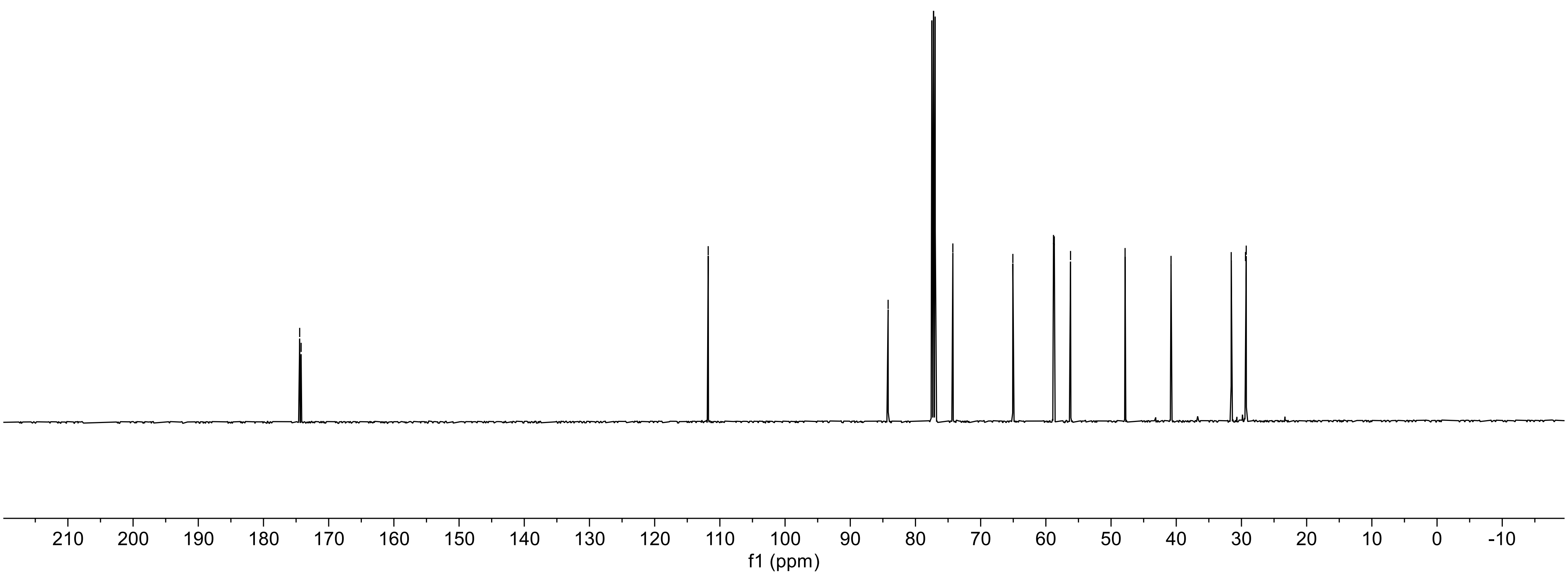

Parameter	Value
Solvent	CDCl3
Spectrometer Frequency	125.80
Nucleus	13C

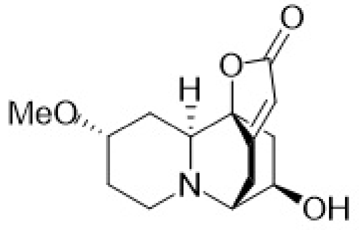

30

Supplementary Figure 48. ^{1}H NMR spectrum of securingine A (**7b**) (500MHz, $CDCl_3$)

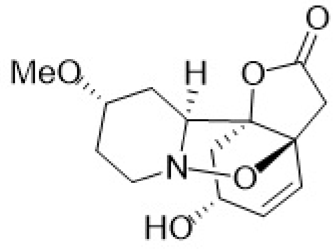

7b securingine A

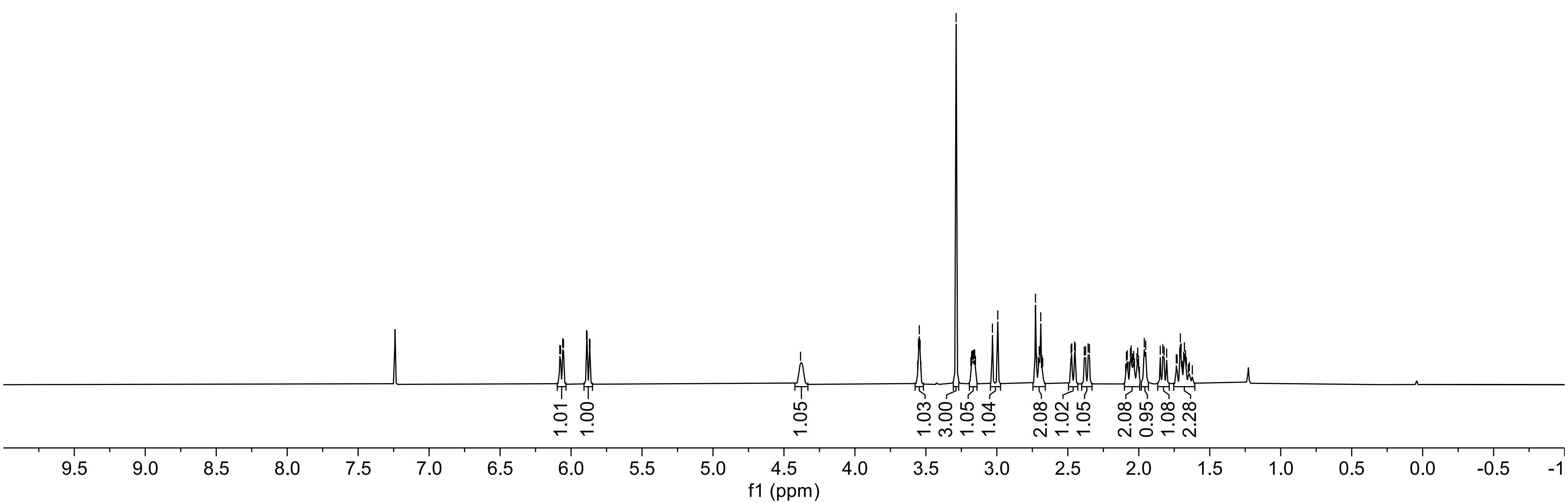

Supplementary Figure 49. ^{13}C NMR spectrum of securingine A (**7b**) (126MHz, $CDCl_3$)

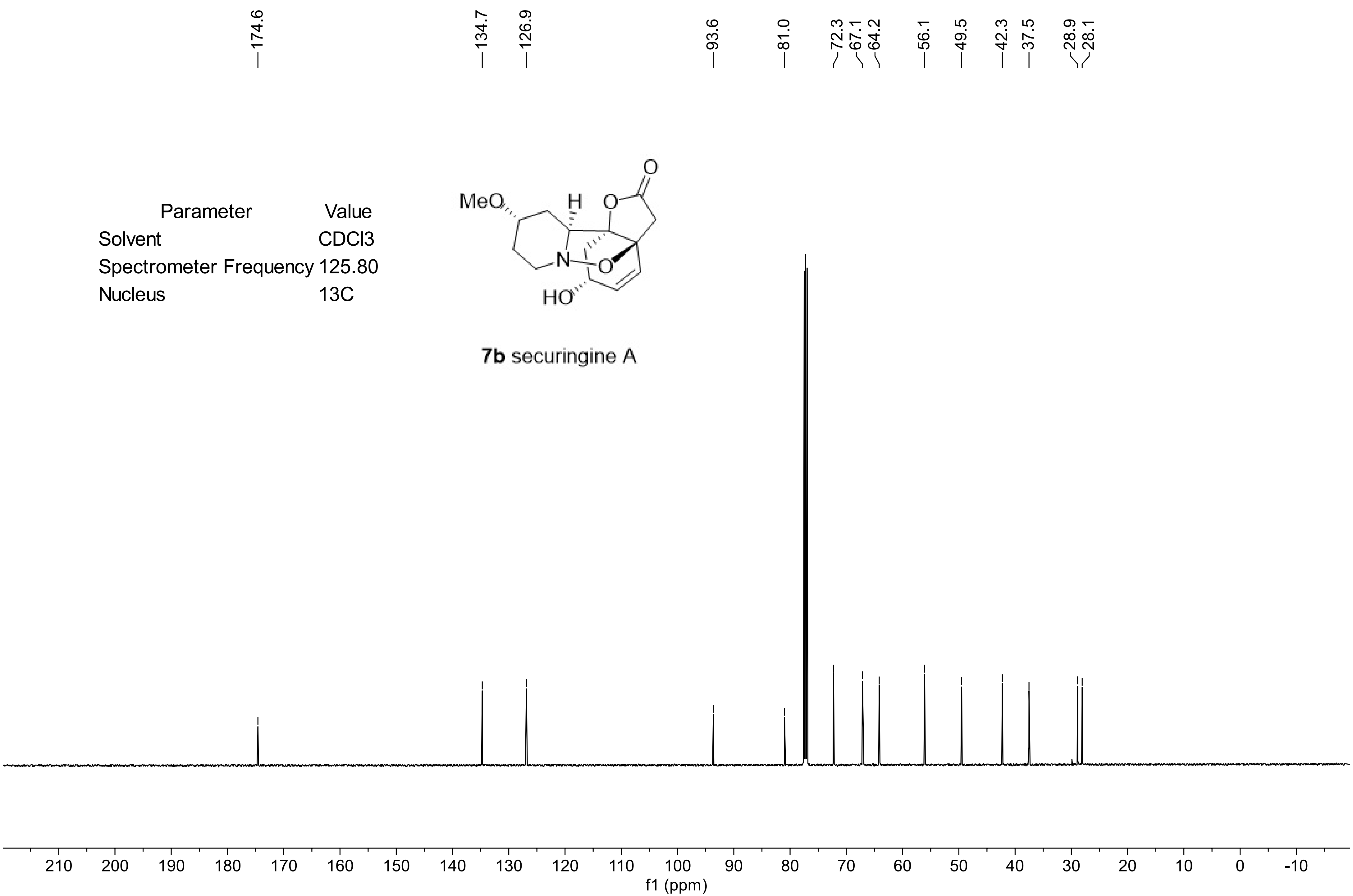

Supplementary Figure 50. ^{1}H NMR spectrum of securitinine (**3**) (400MHz, $CDCl_3$)

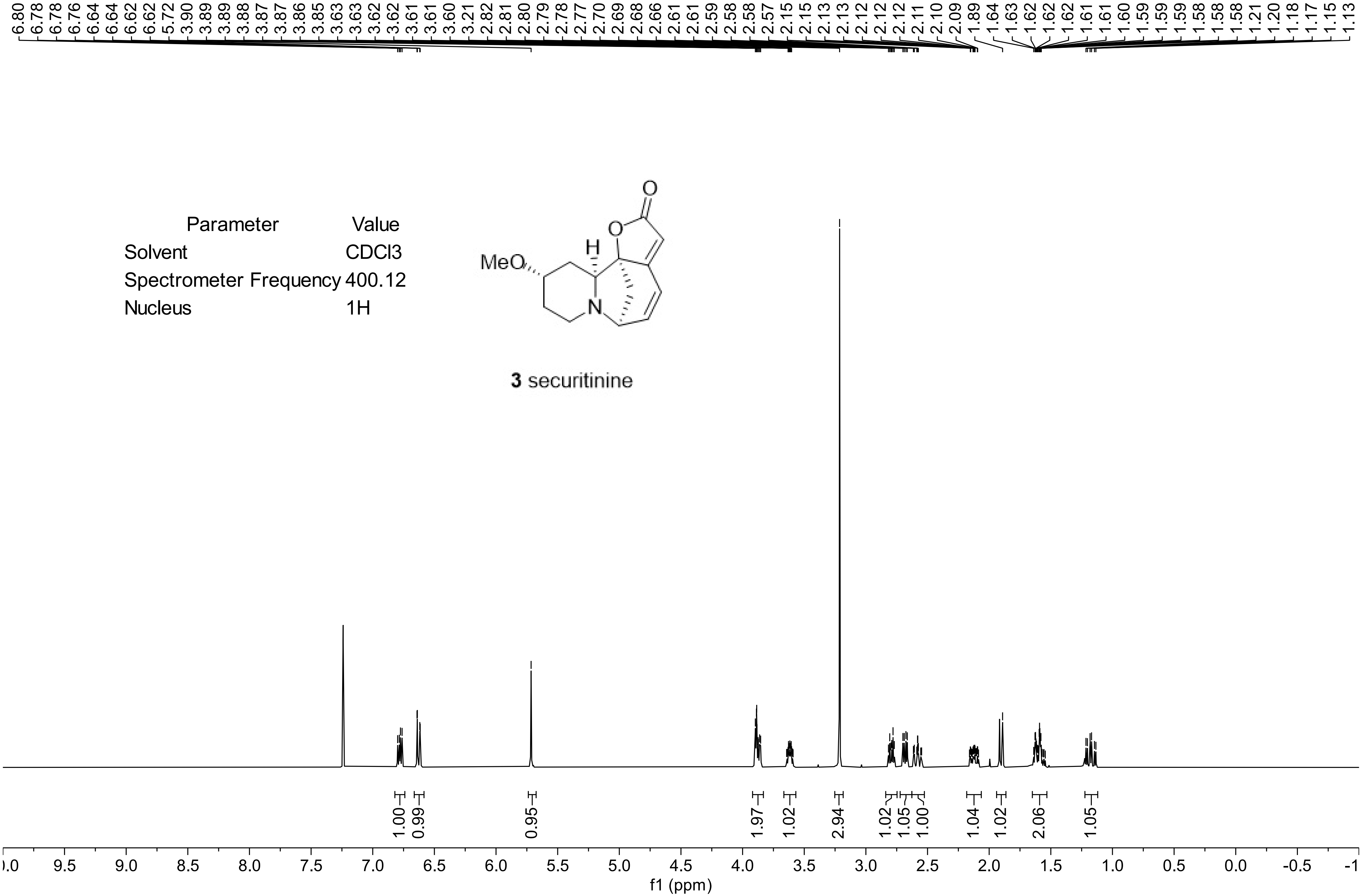

Supplementary Figure 51. ^{13}C NMR spectrum of securitinine (**3**) (101MHz, $CDCl_3$)

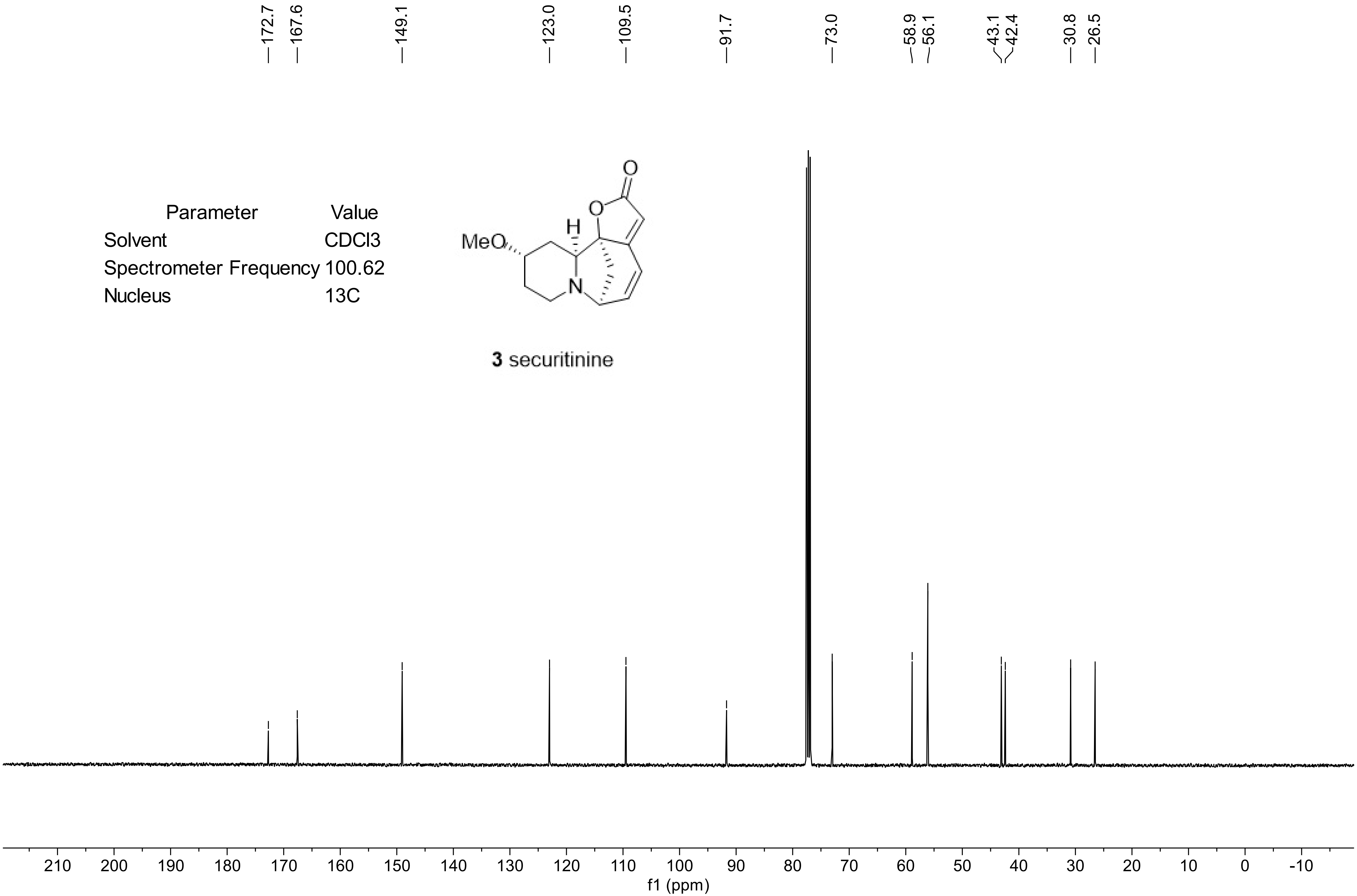

Supplementary Figure 52. ^{1}H NMR spectrum of secu'amamine D (**6**) (400MHz, $CDCl_3$)

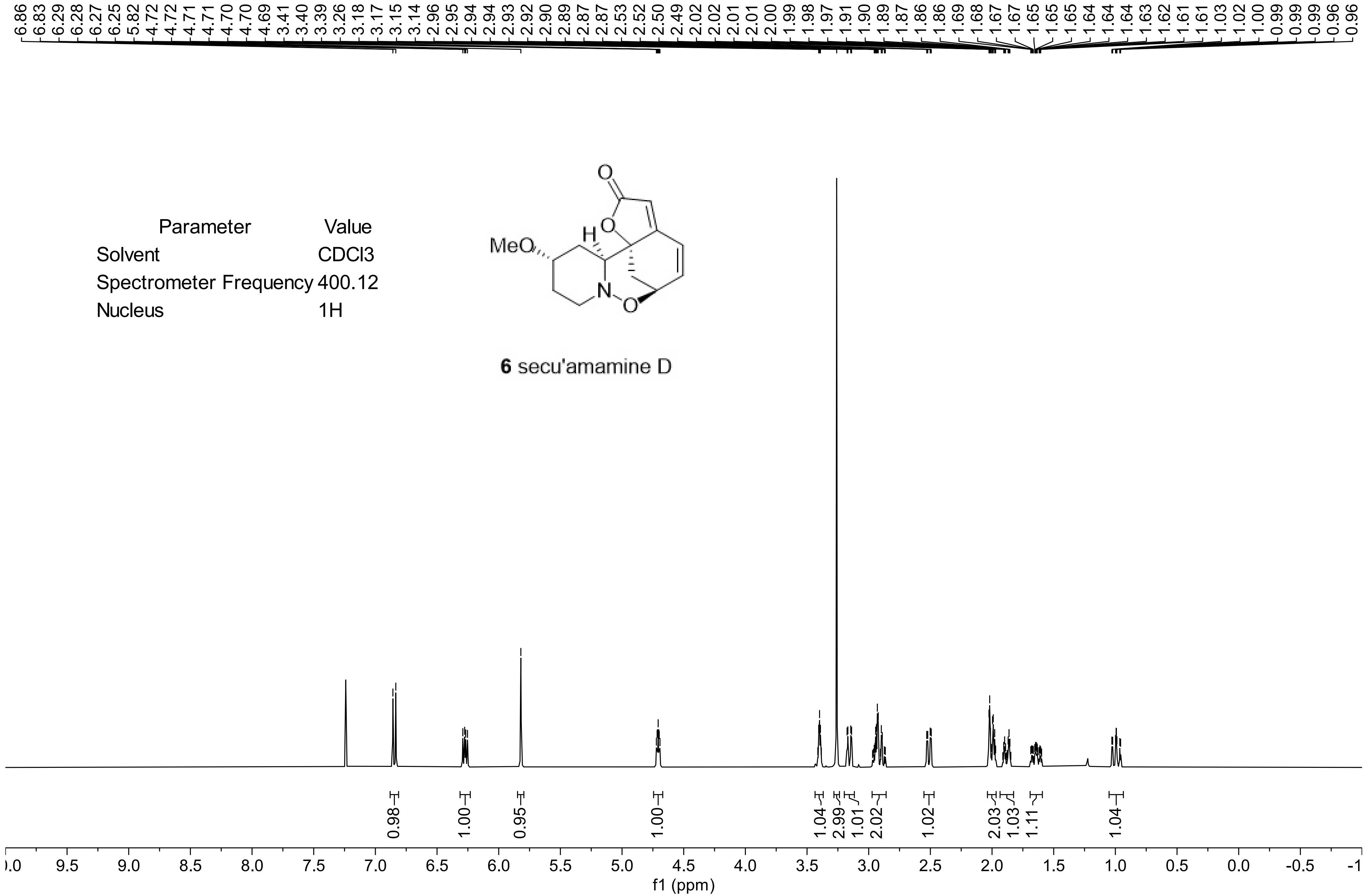

Supplementary Figure 53. ^{13}C NMR spectrum of secu'amamine D (**6**) (101MHz, $CDCl_3$)

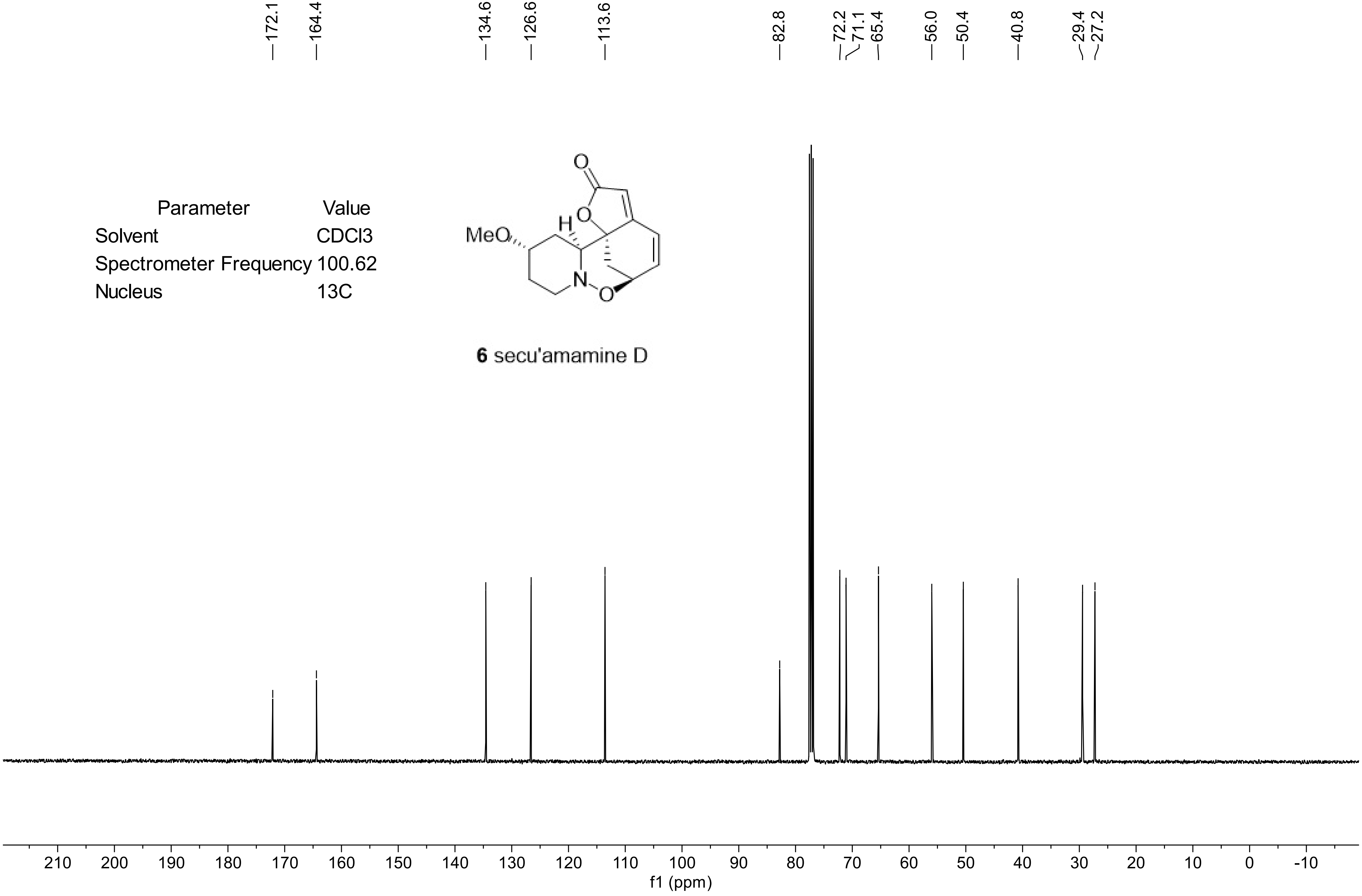

Supplementary Figure 54. ^{1}H NMR spectrum of **36** (400MHz, $CDCl_3$)

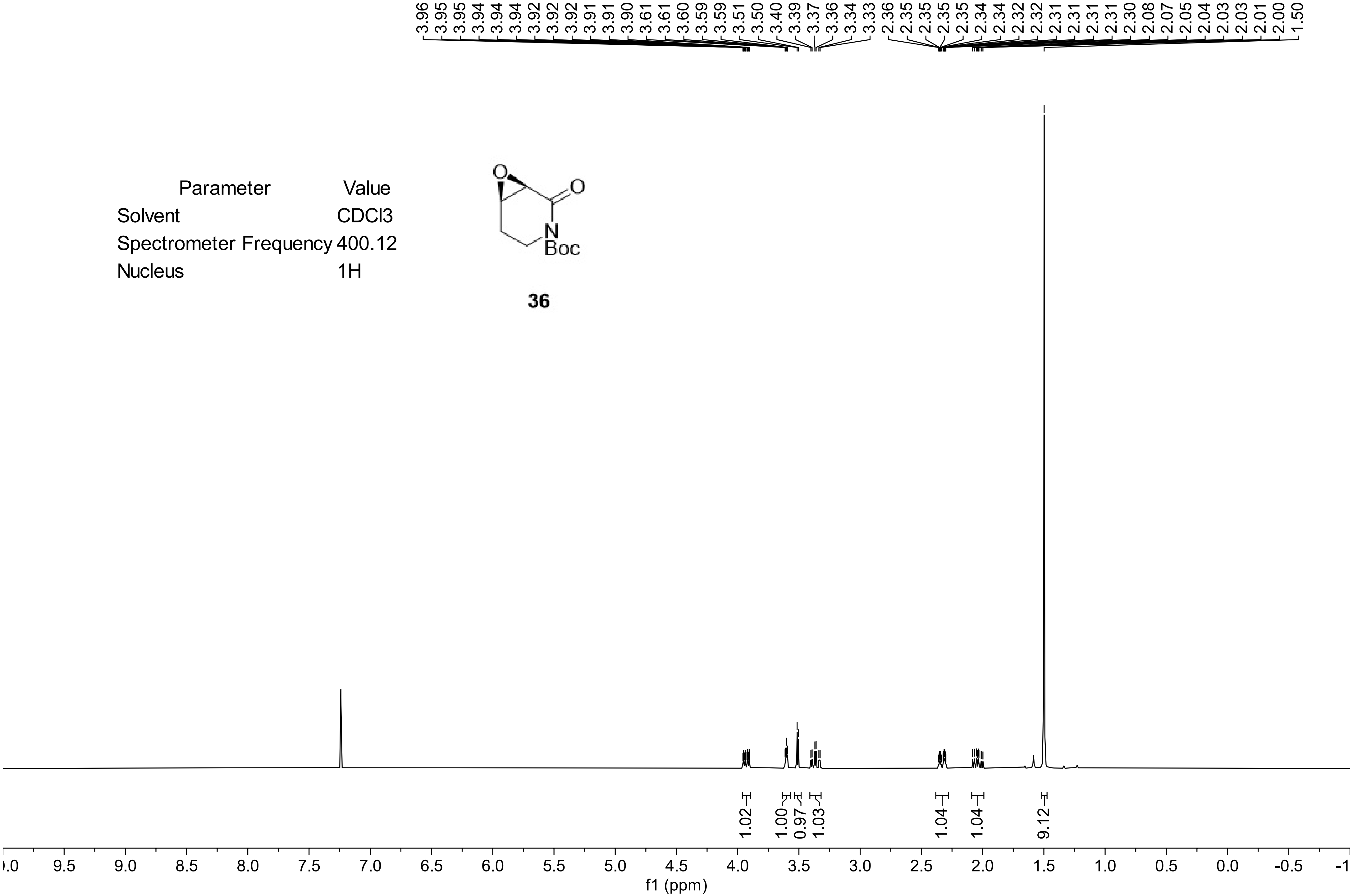

36

Supplementary Figure 55. ^{13}C NMR spectrum of **36** (101MHz, $CDCl_3$)

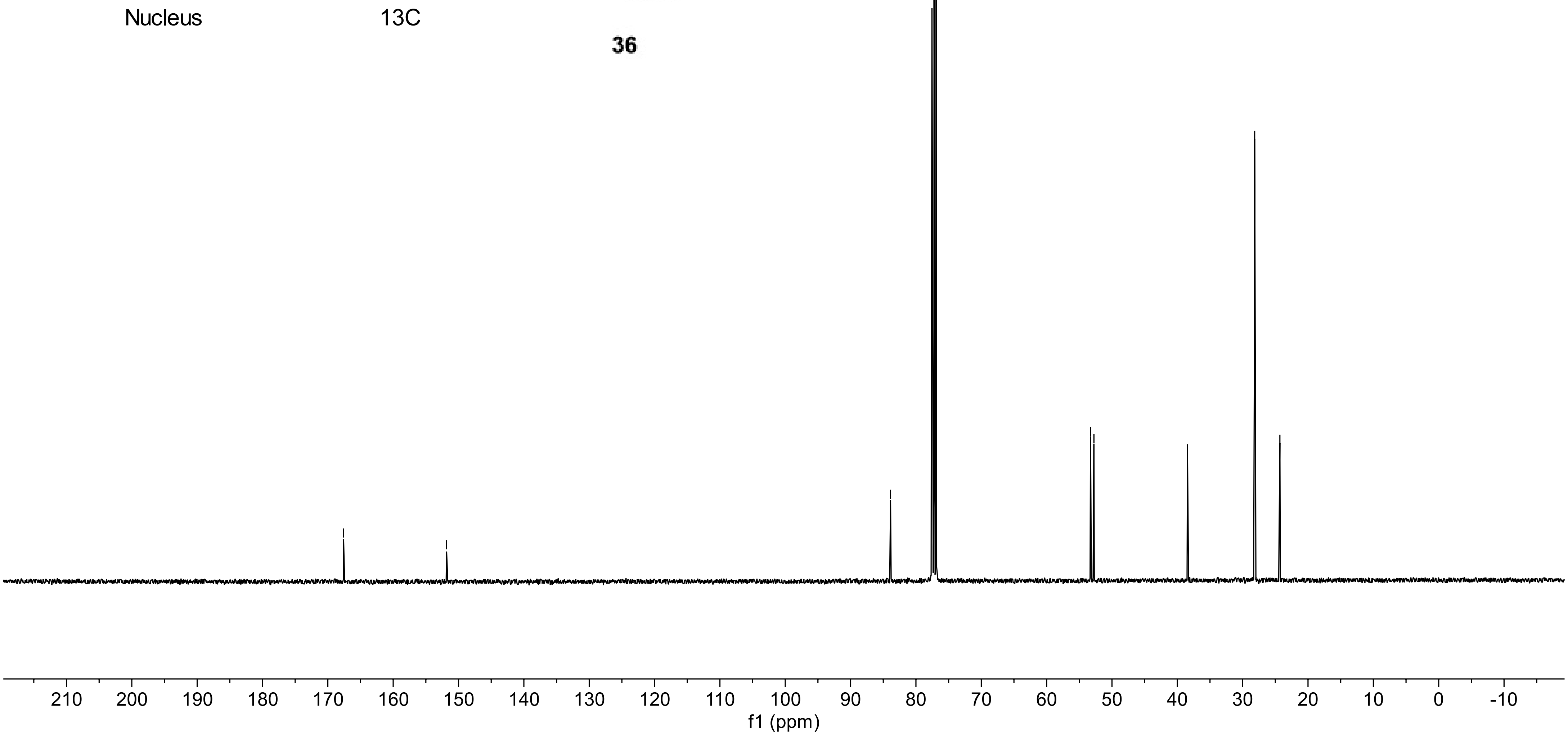

Parameter	Value
Solvent	CDCl3
Spectrometer Frequency	100.62
Nucleus	13C

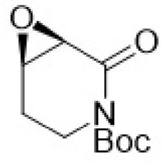

36

Supplementary Figure 56. ^{1}H NMR spectrum of **38** (400MHz, $CDCl_3$)

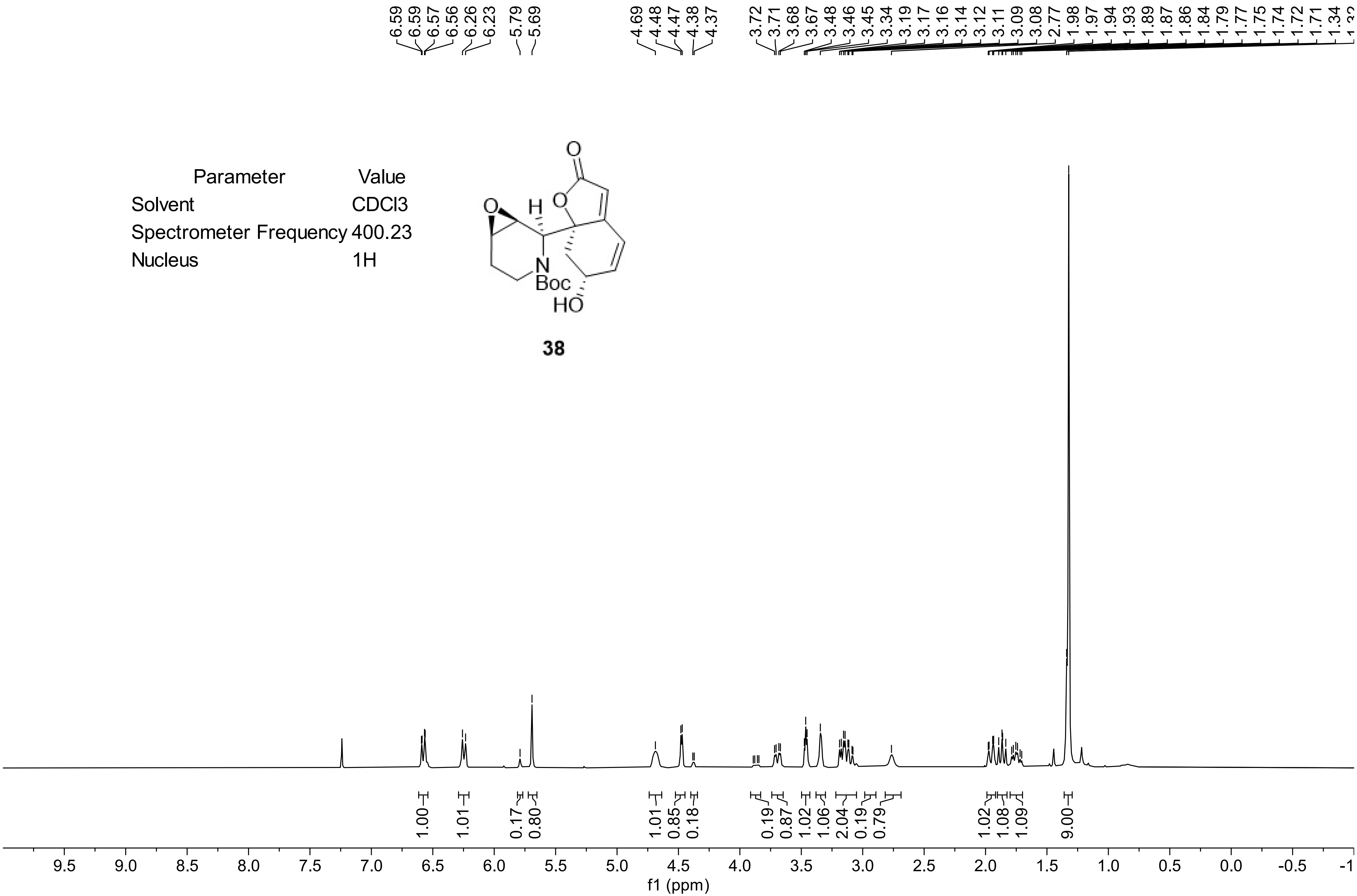

Supplementary Figure 57. ^{13}C NMR spectrum of **38** (101MHz, $CDCl_3$)

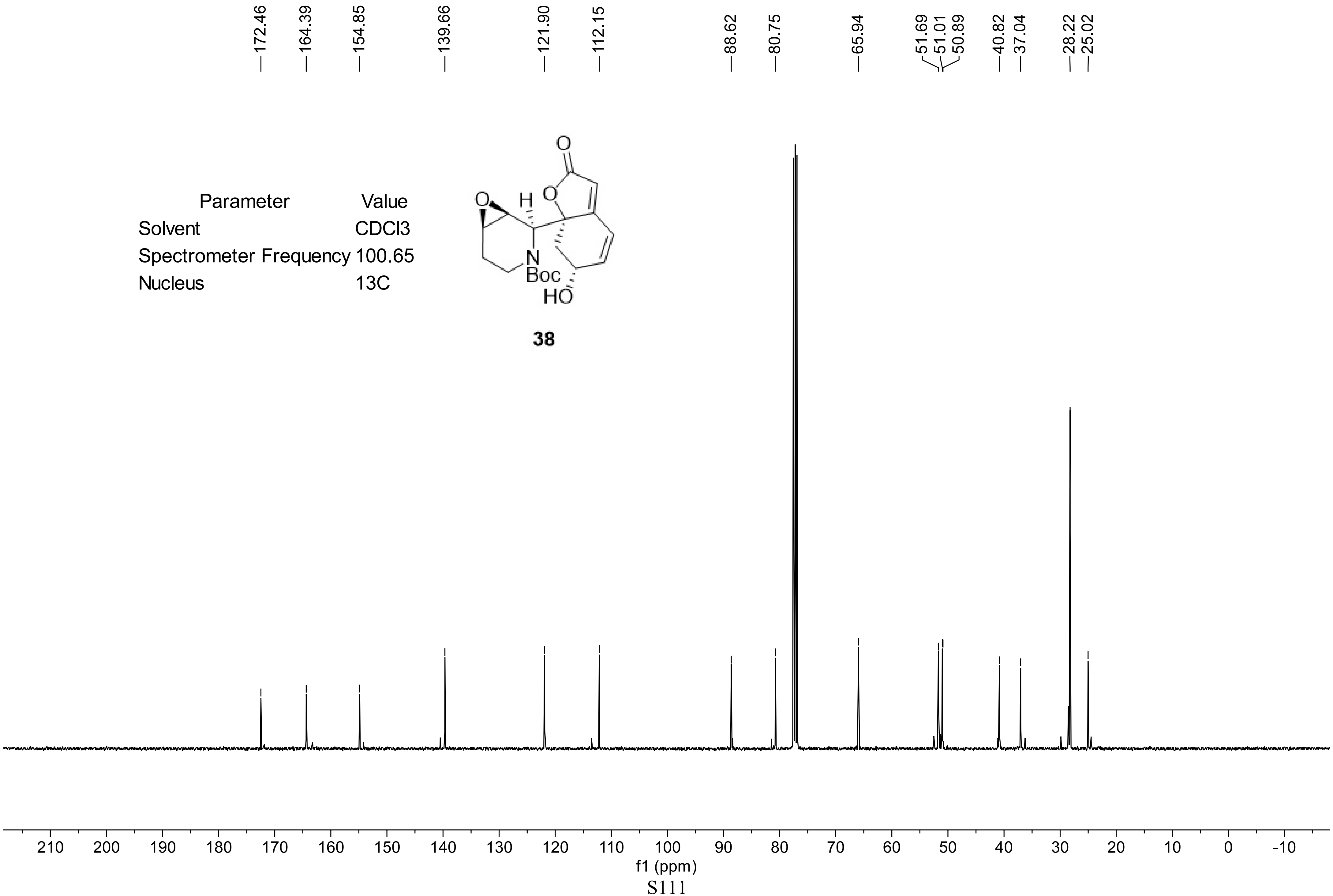

Supplementary Figure 58. NOESY NMR spectrum of **38** (500MHz, $CDCl_3$)

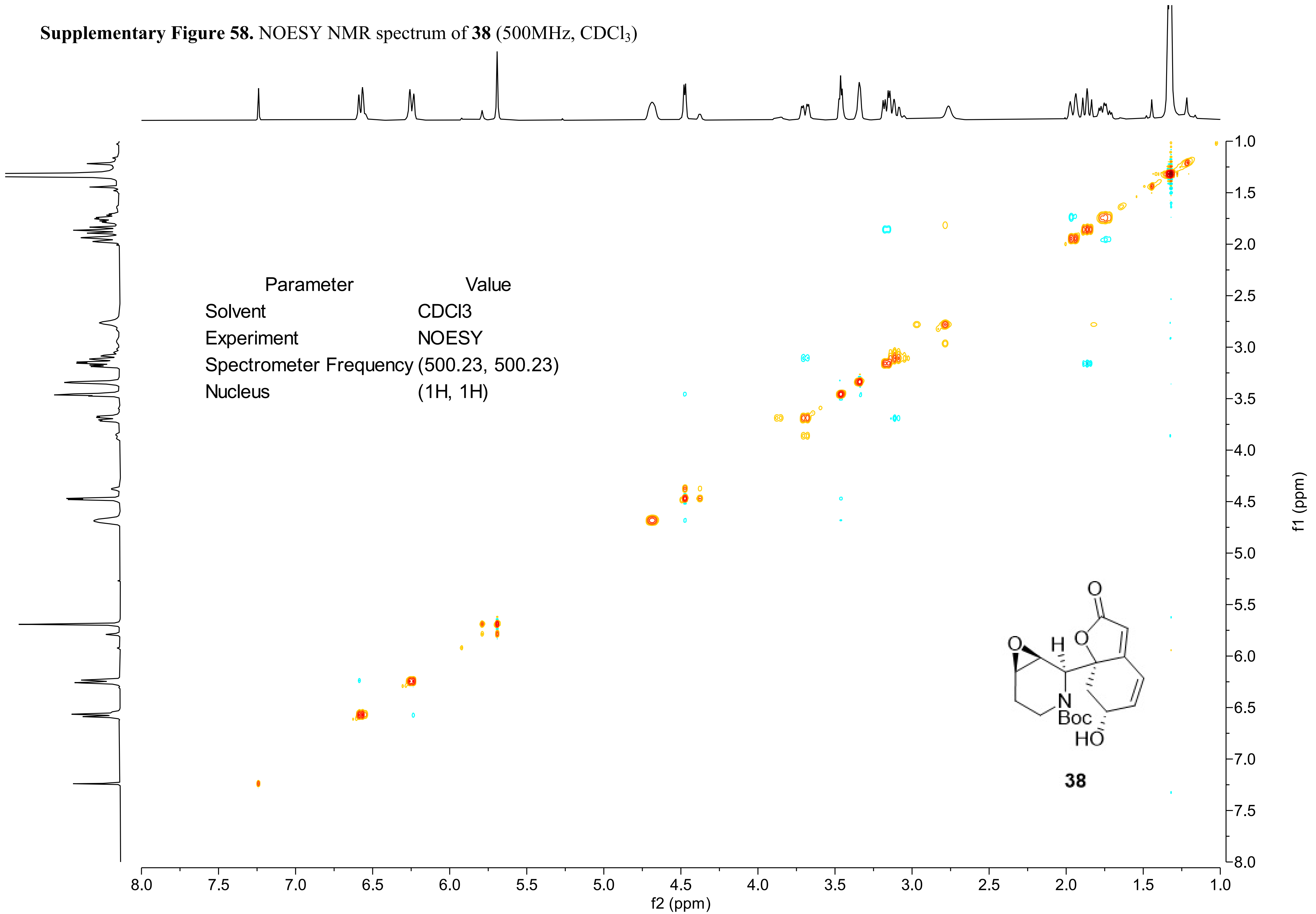

Parameter	Value
Solvent	CDCl3
Experiment	NOESY
Spectrometer Frequency	(500.23, 500.23)
Nucleus	(1H, 1H)

Supplementary Figure 59. ^{1}H NMR spectrum of securingine C (**8b**) (400MHz, $CDCl_3$)

Parameter	Value
Solvent	CDCl3
Spectrometer Frequency	400.23
Nucleus	1H

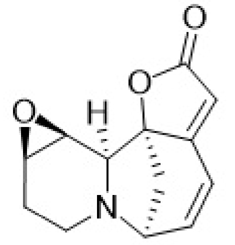

8b securingine C

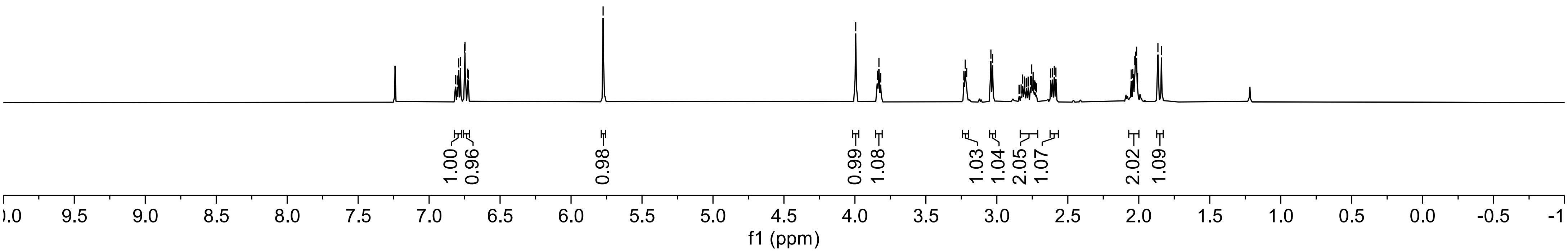

Supplementary Figure 60. ^{13}C NMR spectrum of securingine C (**8b**) (101MHz, $CDCl_3$)

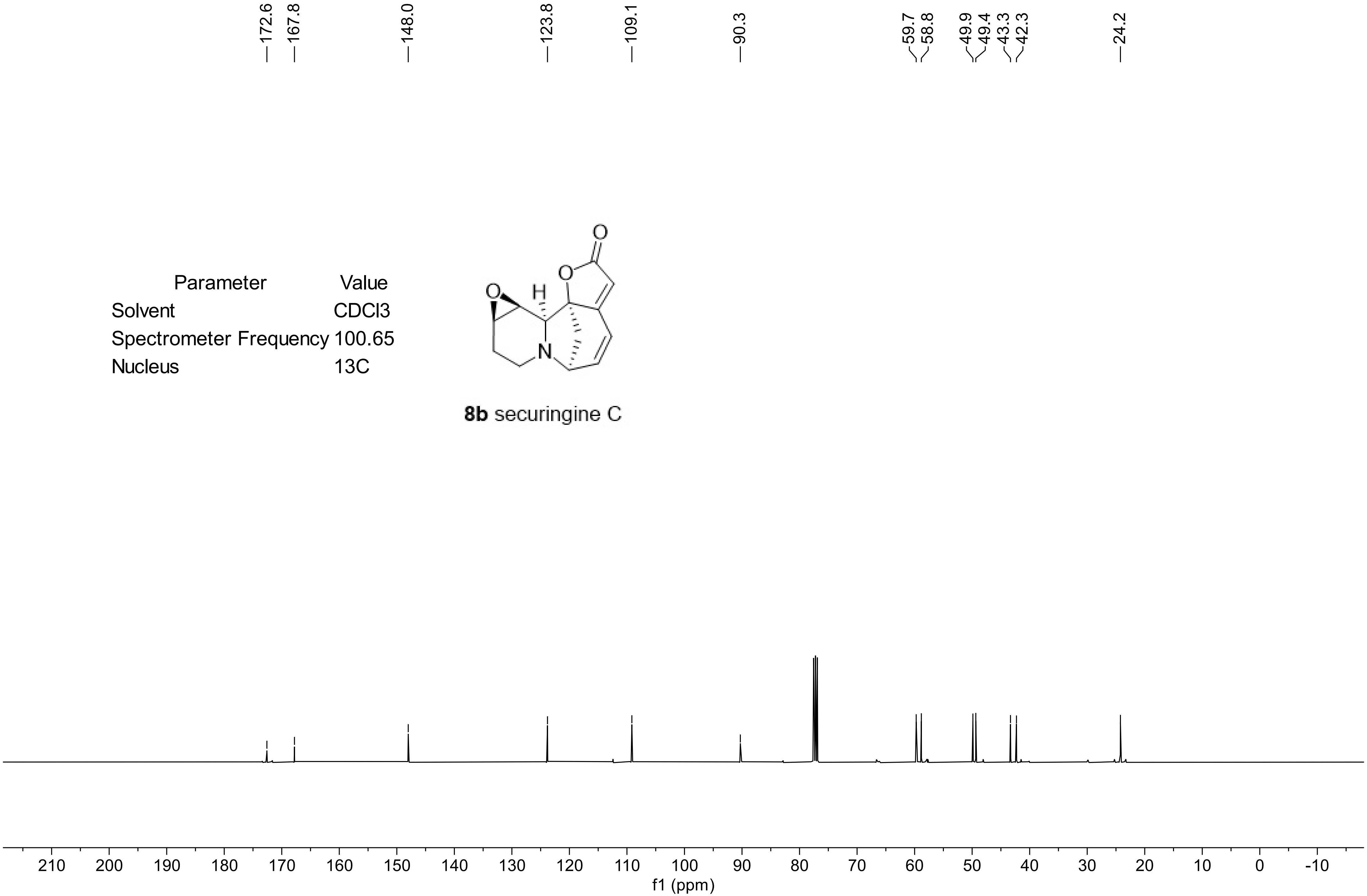

Parameter	Value
Solvent	CDCl3
Spectrometer Frequency	100.65
Nucleus	13C

8b securingine C

Supplementary Figure 61. ^{1}H NMR spectrum of securingine D (**9b**) (400MHz, $CDCl_3$)

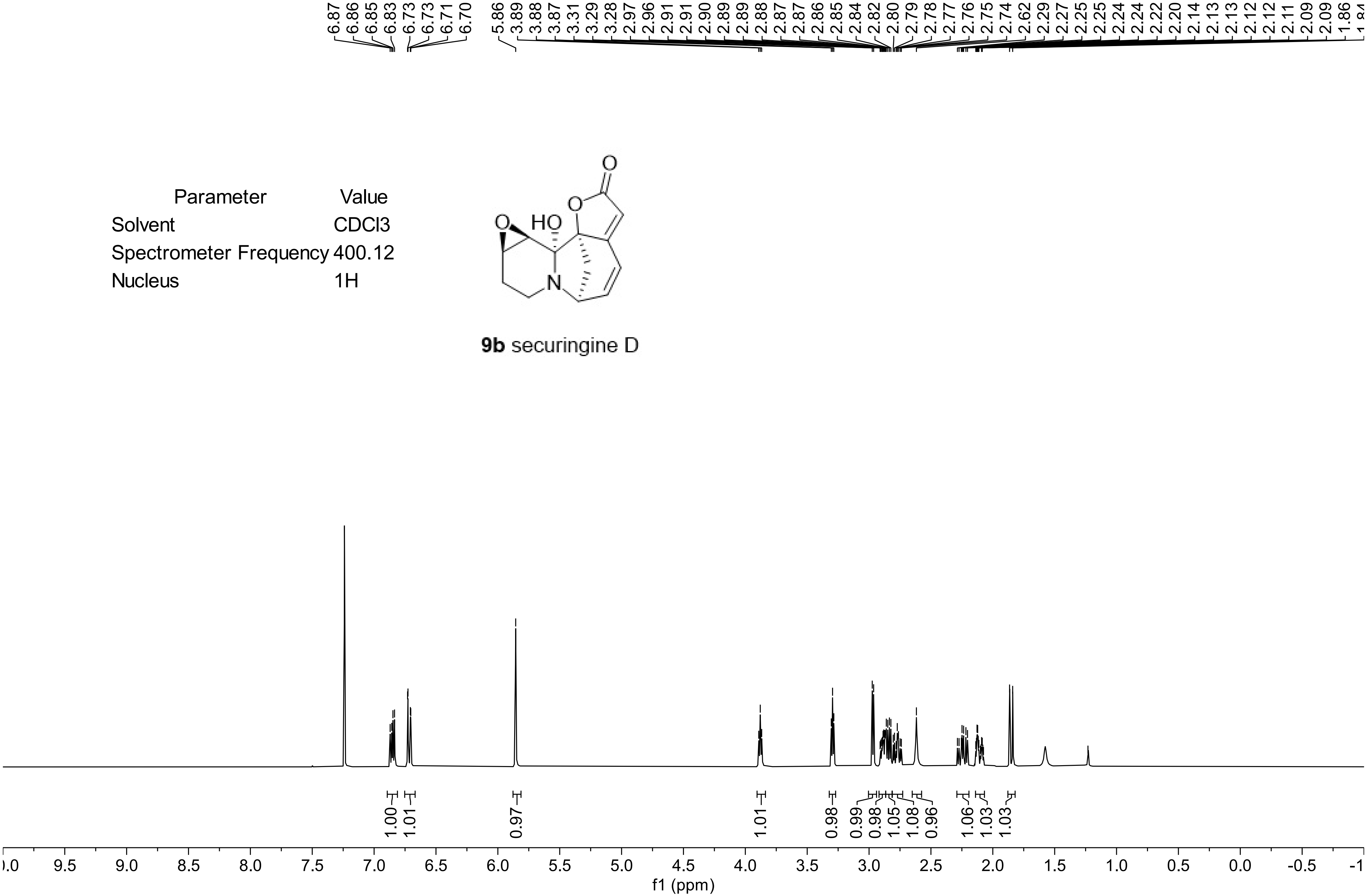

Supplementary Figure 62. ^{13}C NMR spectrum of securingine D (**9b**) (101MHz, $CDCl_3$)

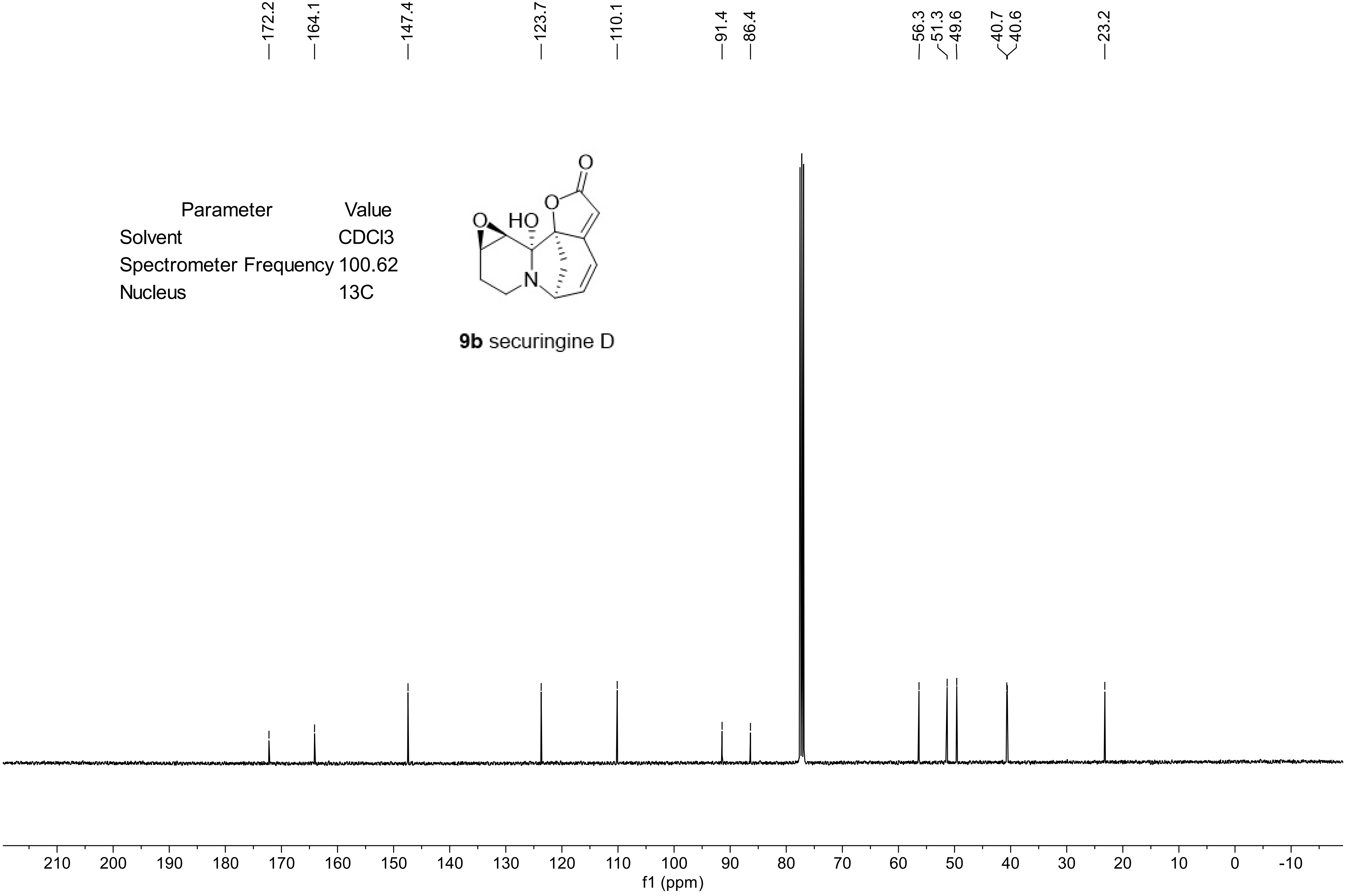

Supplementary Figure 63. ^{1}H NMR spectrum of **42** (400MHz, $CDCl_3$)

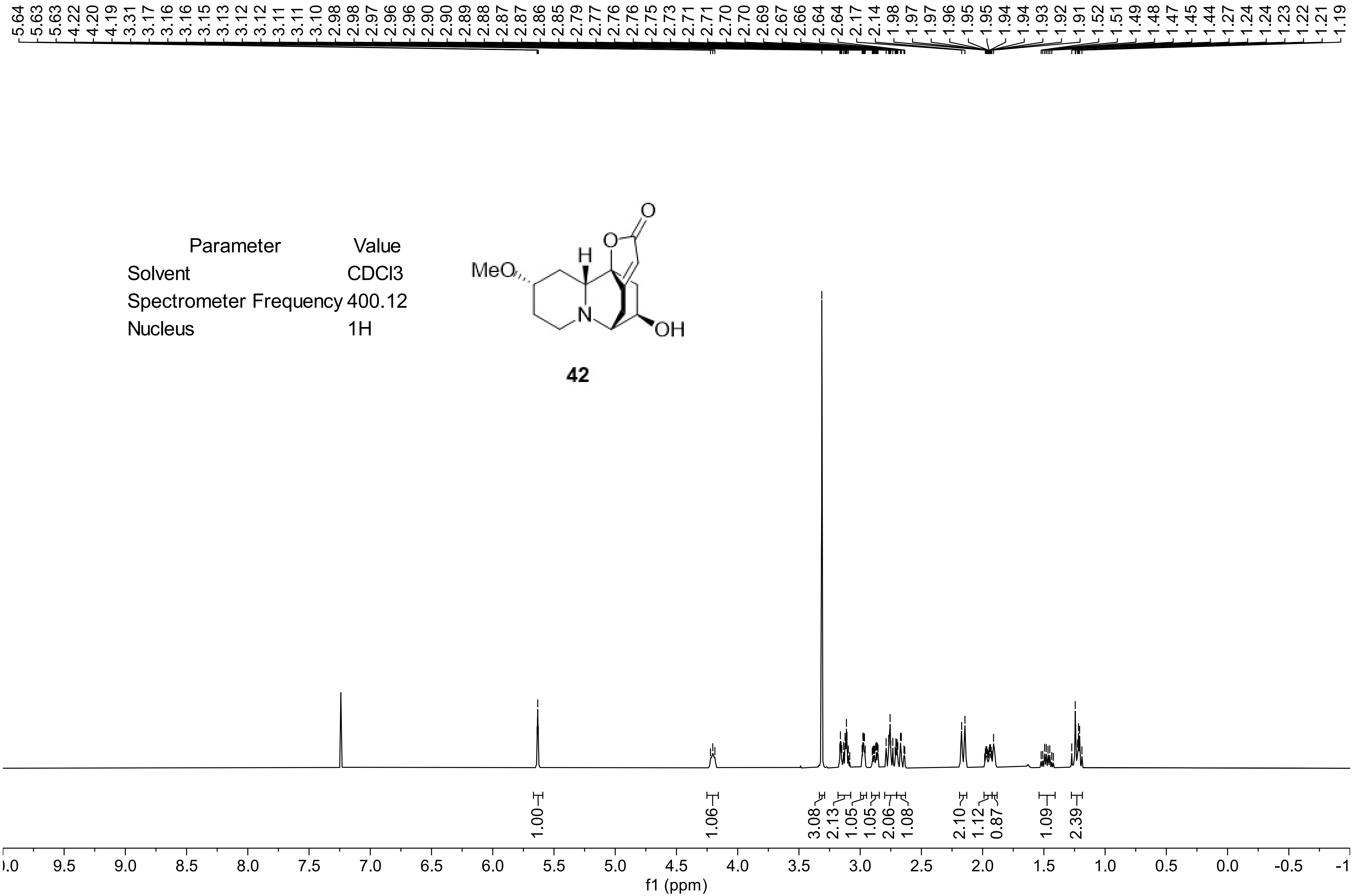

Supplementary Figure 64. ^{13}C NMR spectrum of **42** (101MHz, $CDCl_3$)

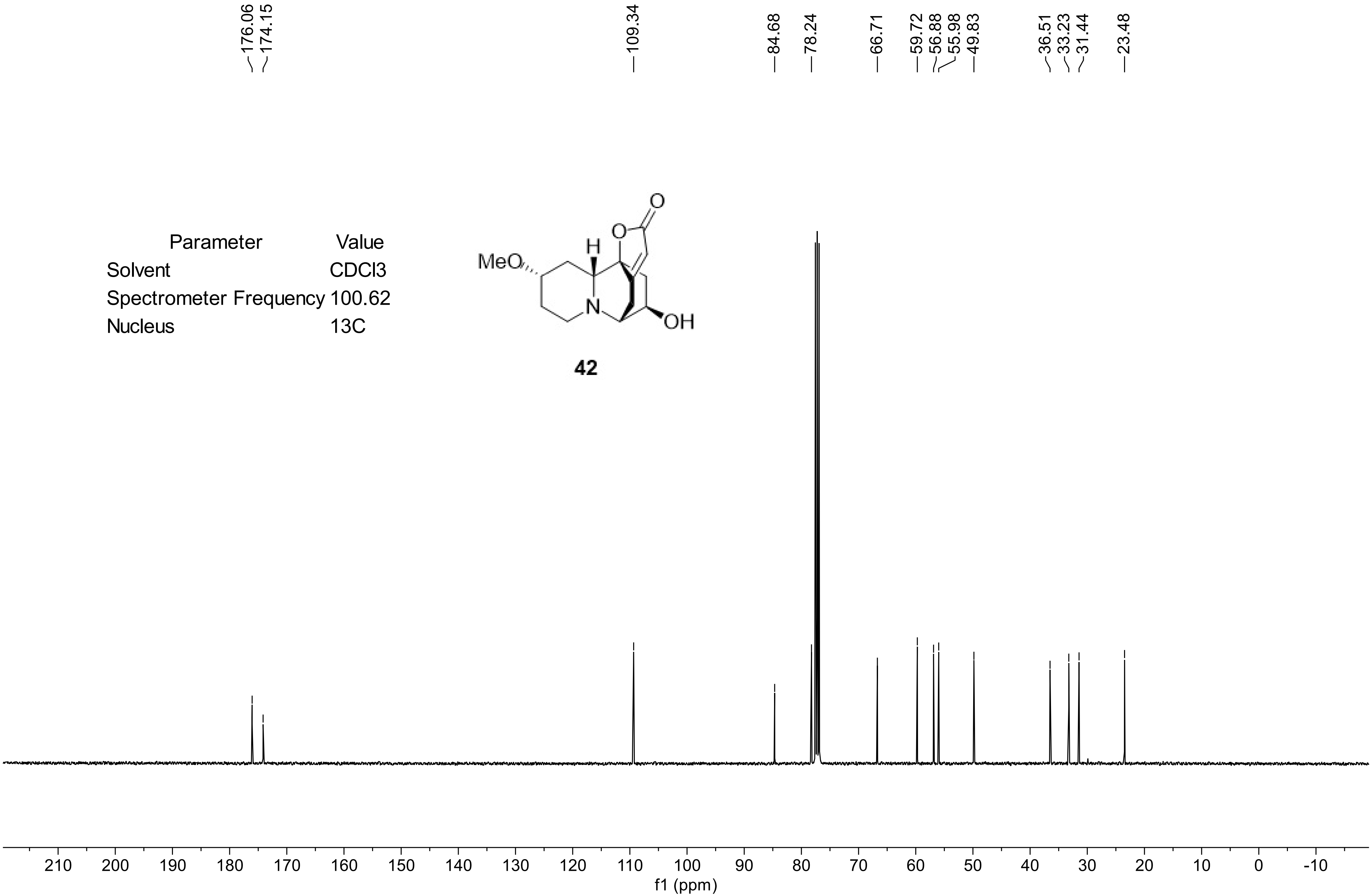

Supplementary Figure 65. ^{1}H NMR spectrum of 4-*epi*-phyllanthine (**4**) (400MHz, $CDCl_3$)

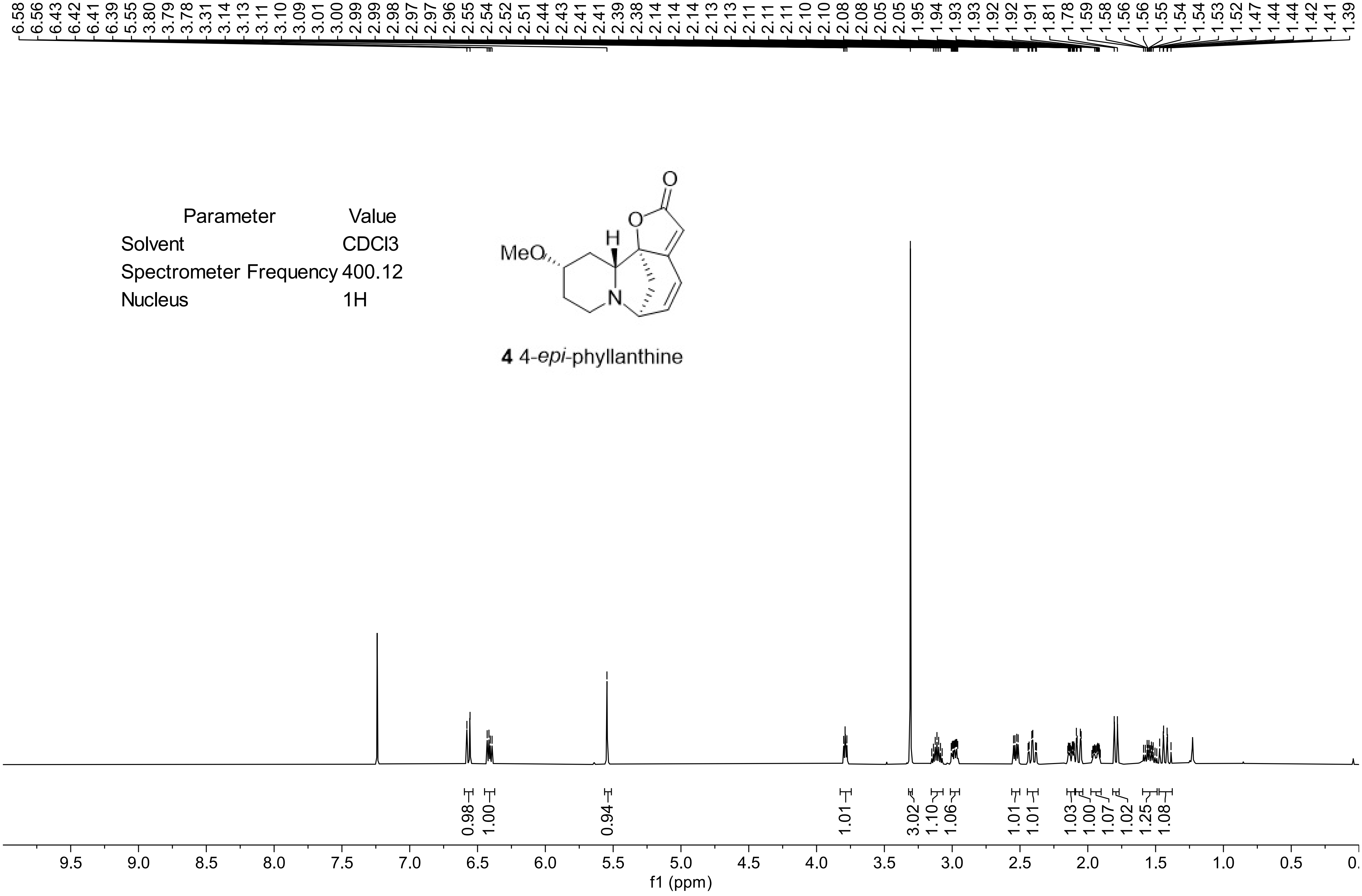

Supplementary Figure 66. ^{13}C NMR spectrum of 4-*epi*-phyllanthine (**4**) (101MHz, $CDCl_3$)

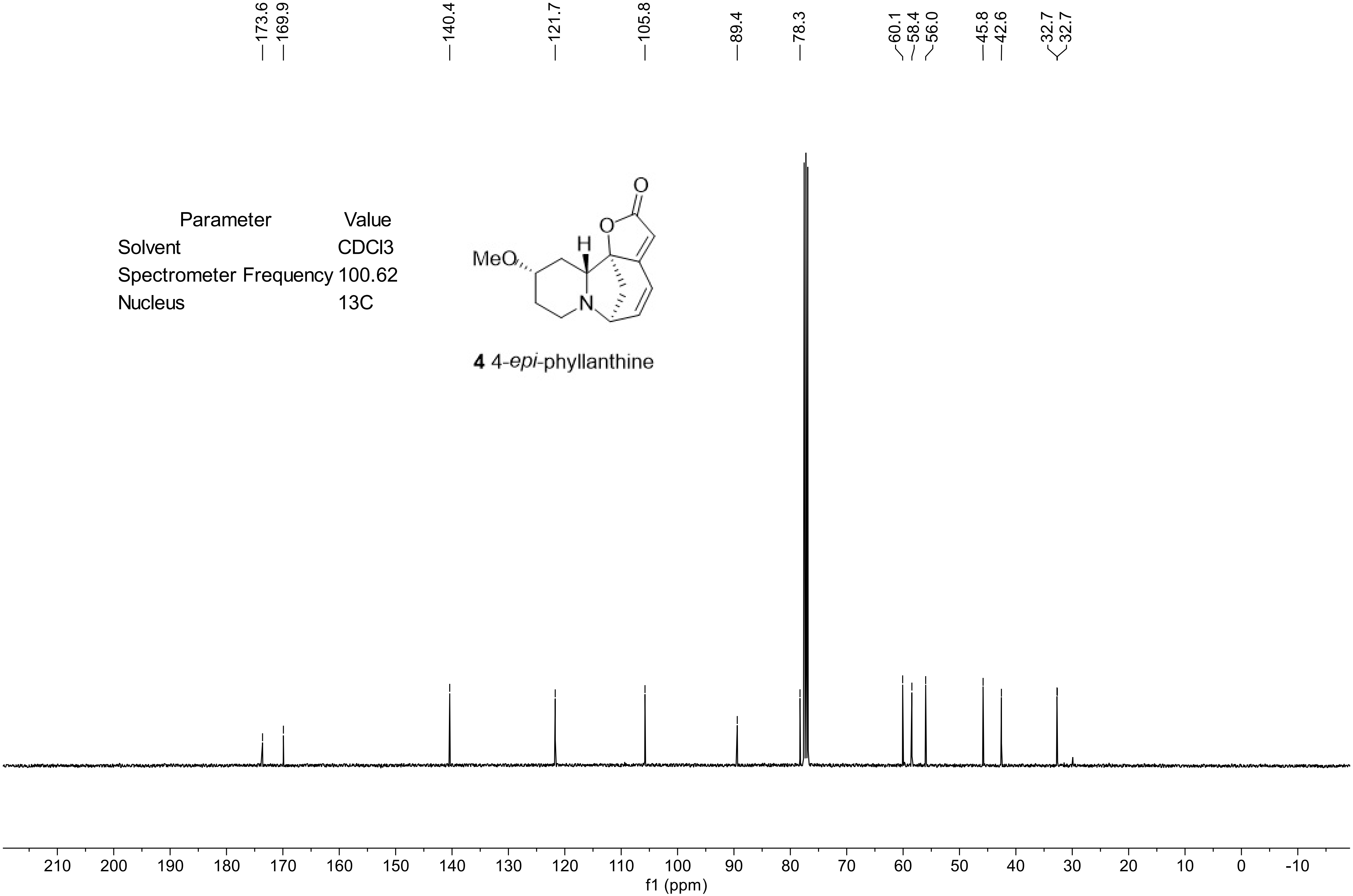

Supplementary Figure 67. ^{1}H NMR spectrum of *ent*-virosine B (**44**) (500MHz, $CDCl_3$)

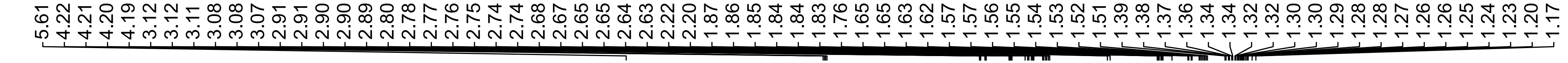

Parameter	Value
Solvent	CDCl3
Spectrometer Frequency	500.23
Nucleus	1H

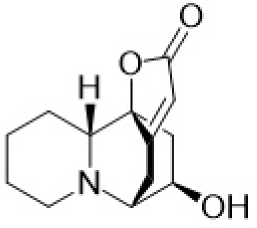

44 *ent*-virosine B

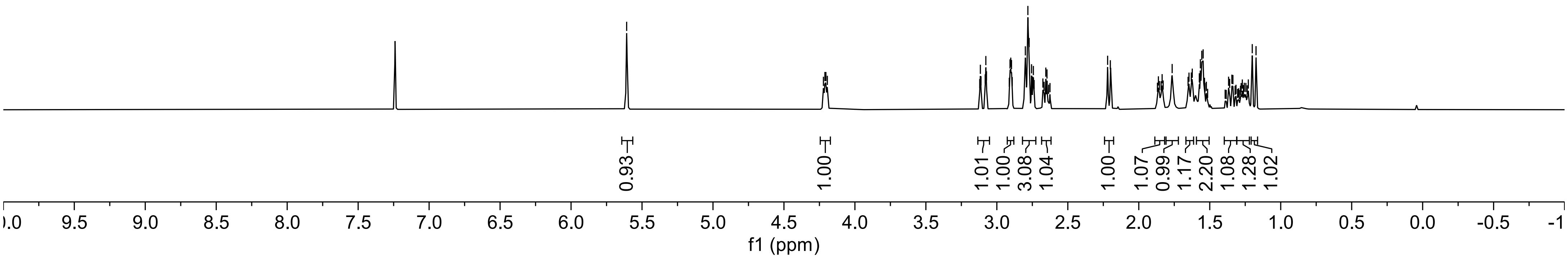

Supplementary Figure 68. ^{13}C NMR spectrum of *ent*-virosine B (**44**) (126MHz, $CDCl_3$)

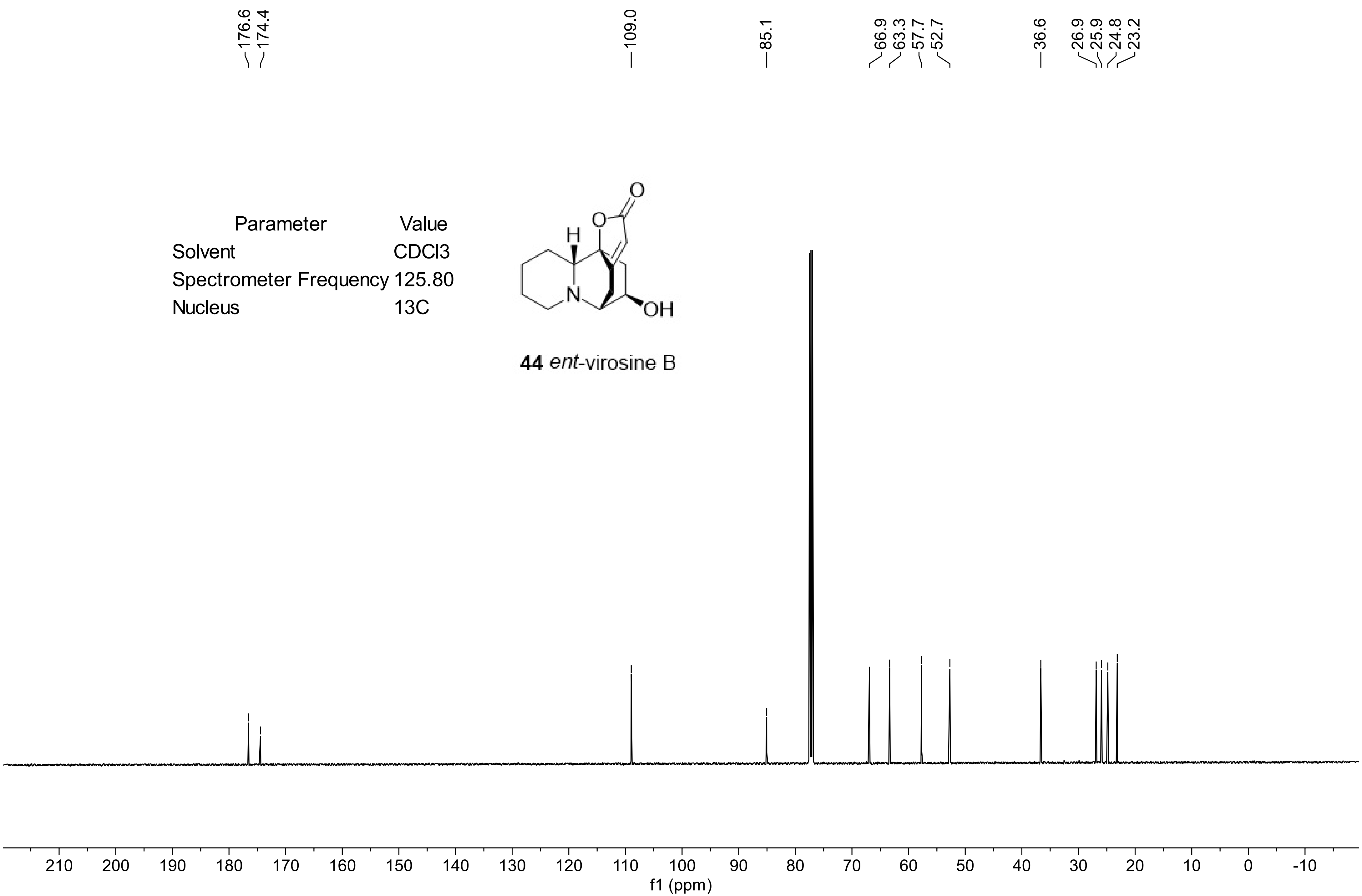

Supplementary Figure 69. ^{1}H NMR spectrum of securinine (**1**) (400MHz, $CDCl_3$)

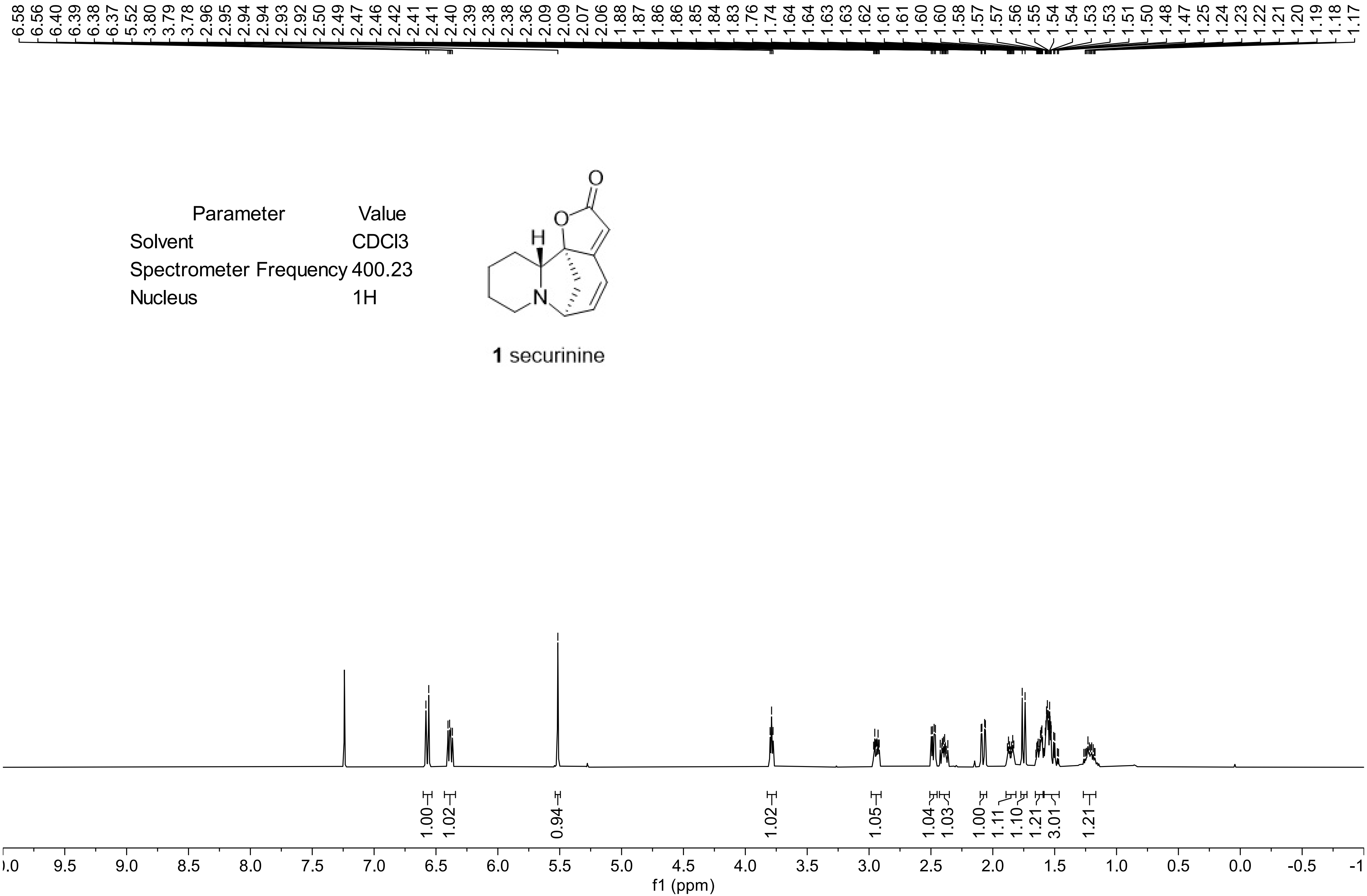

Parameter	Value
Solvent	CDCl3
Spectrometer Frequency	400.23
Nucleus	1H

1 securinine

Supplementary Figure 70. ^{13}C NMR spectrum of securinine (**1**) (101MHz, $CDCl_3$)

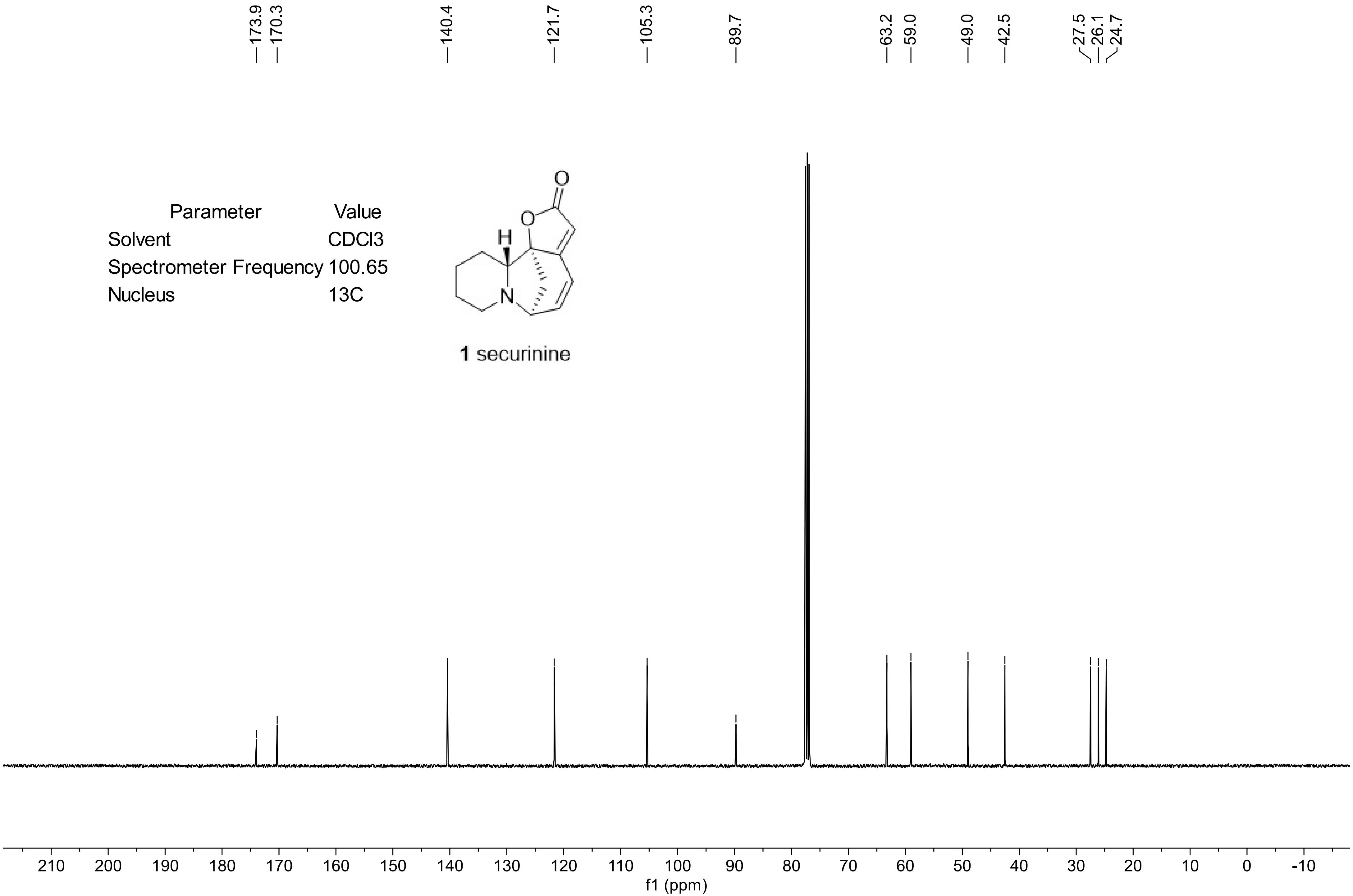

Supplementary Figure 71. ^{1}H NMR spectrum of allosecurinine (**2**) (400MHz, $CDCl_3$)

Parameter	Value
Solvent	CDCl3
Spectrometer Frequency	400.12
Nucleus	1H

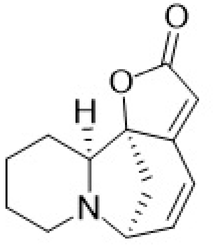

2 allosecurinine

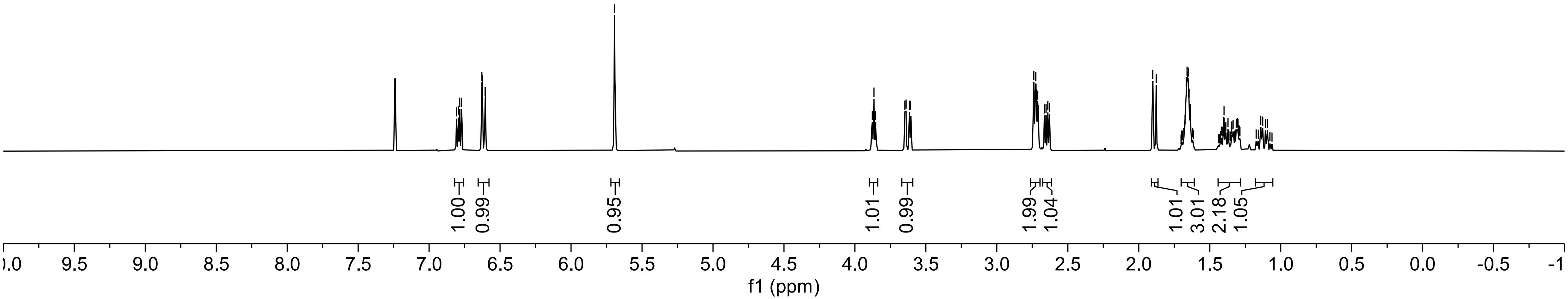

Supplementary Figure 72. ^{13}C NMR spectrum of allosecurinine (**2**) (101MHz, $CDCl_3$)

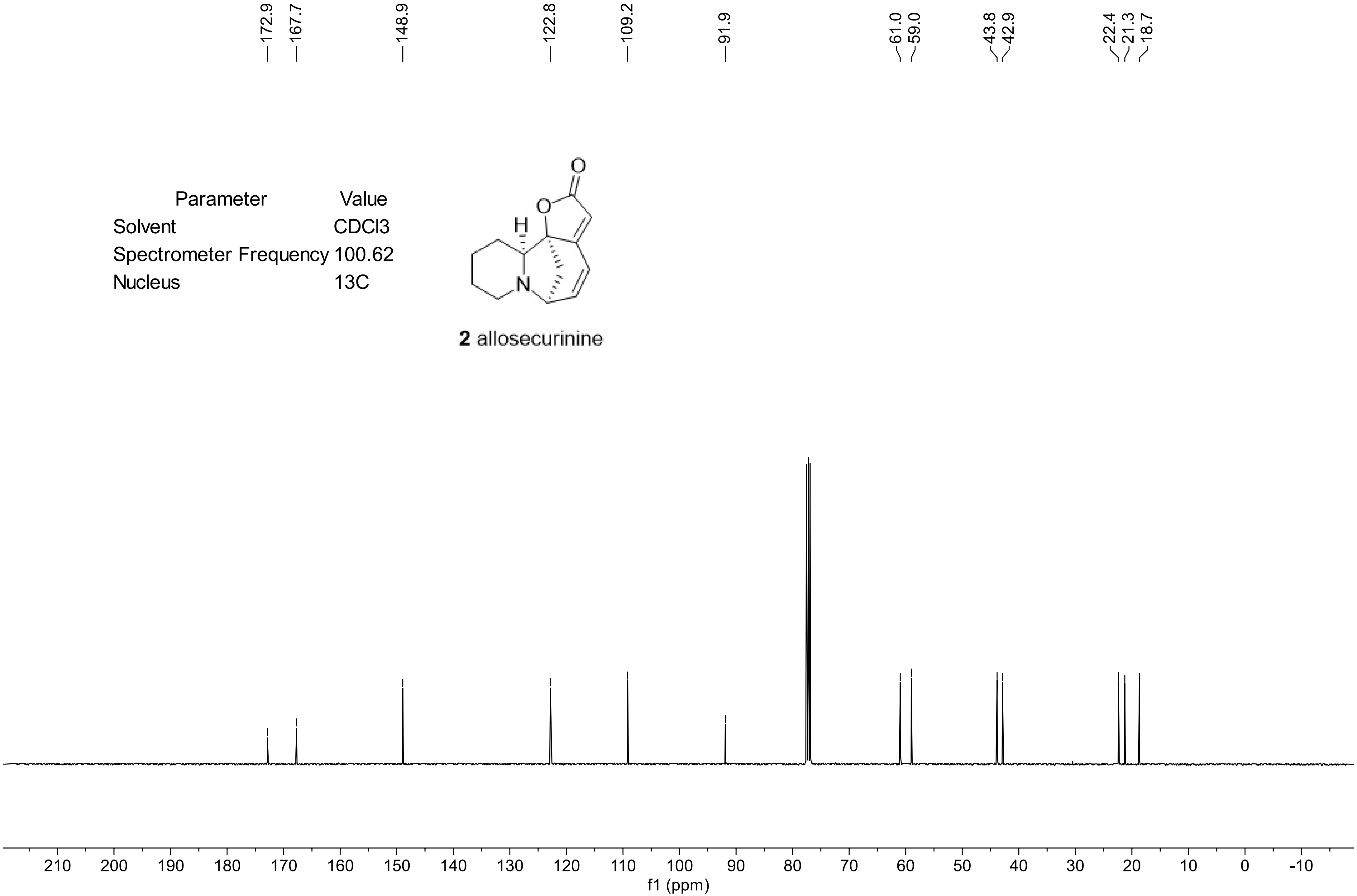

Supplementary Figure 73. ^{1}H NMR spectrum of **46** (400MHz, $CDCl_3$)

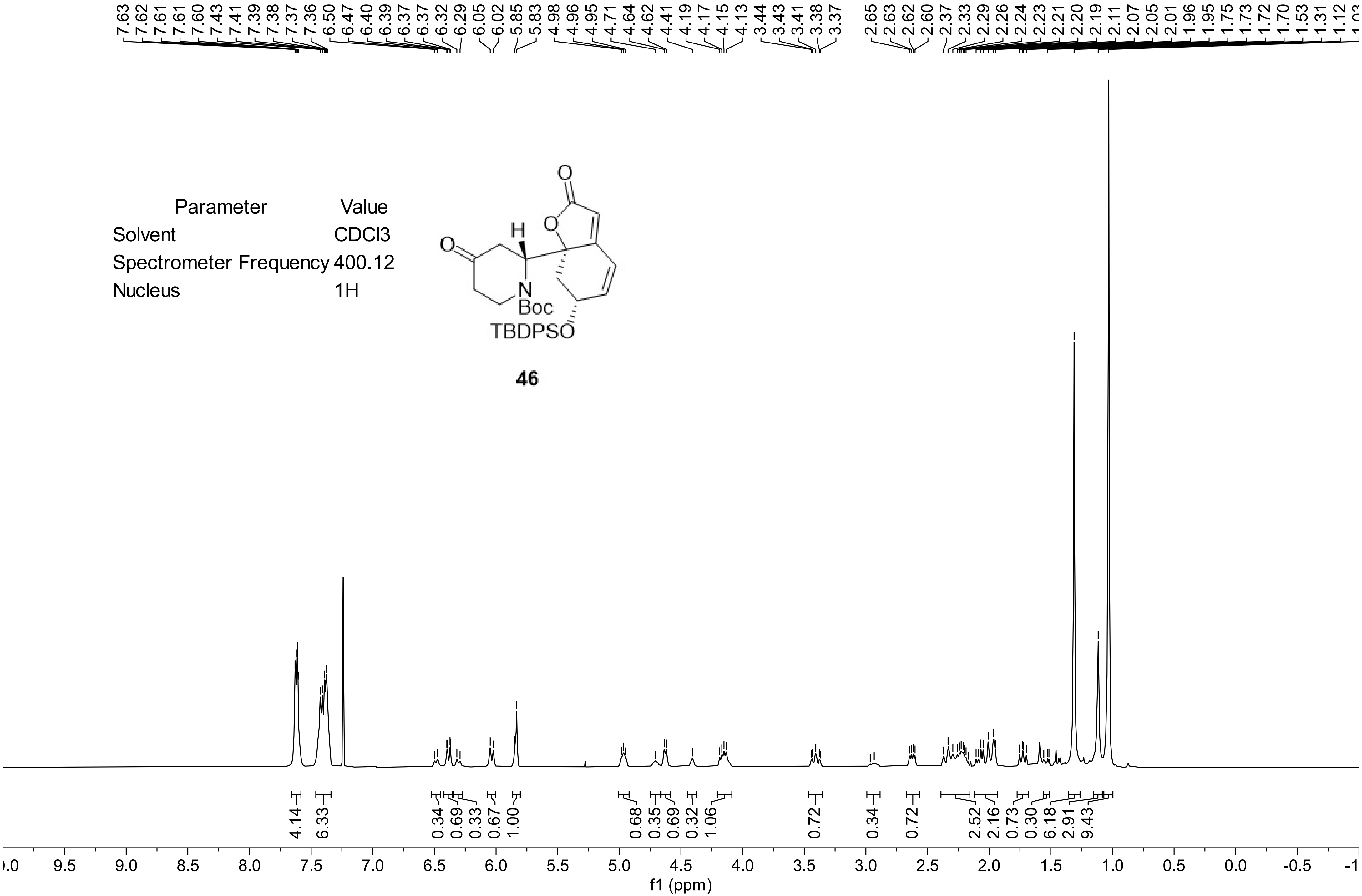

Supplementary Figure 74. ^{13}C NMR spectrum of **46** (101MHz, $CDCl_3$)

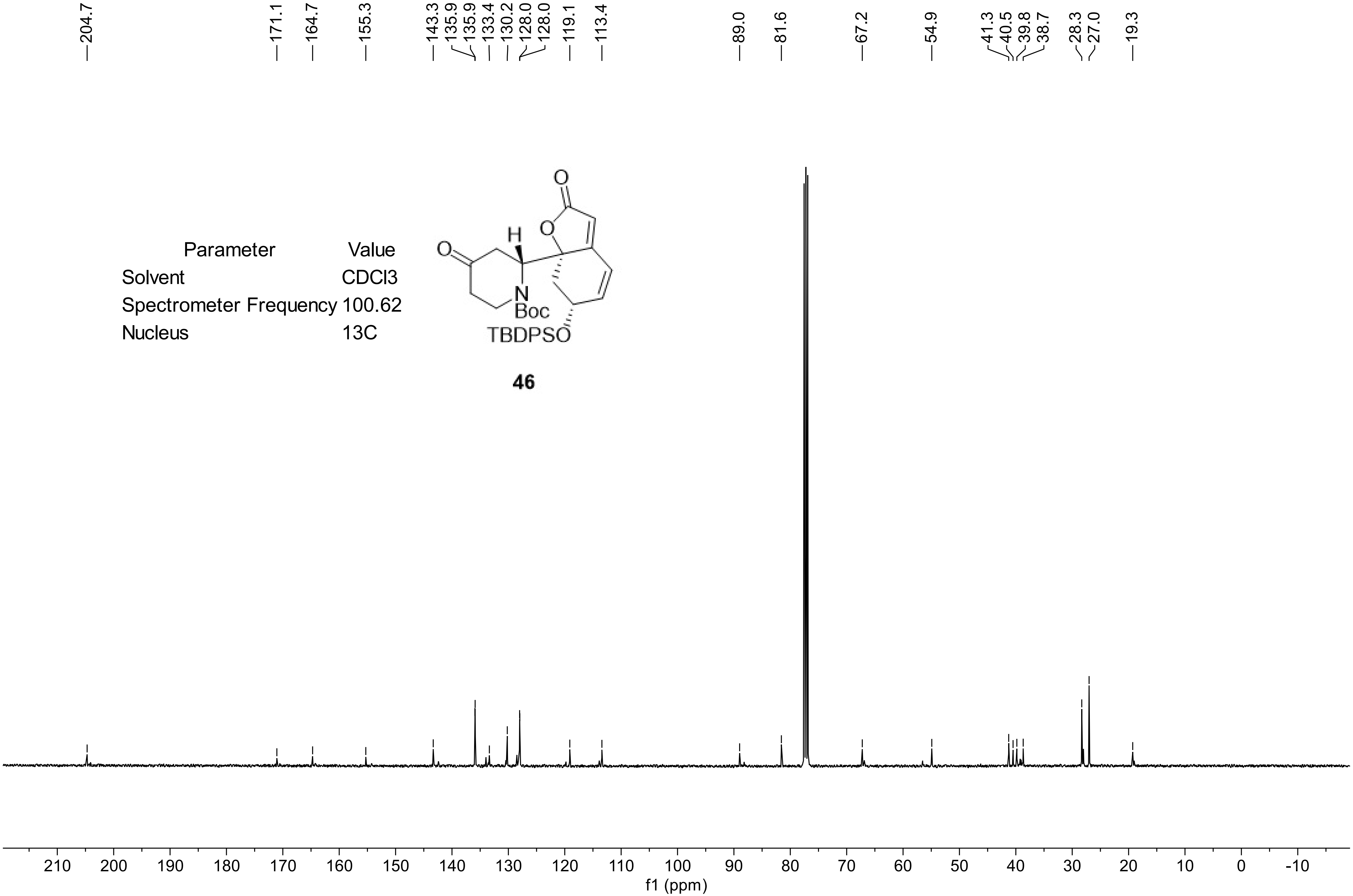

Supplementary Figure 75. NOESY NMR spectrum of **46** (400MHz, $CDCl_3$)

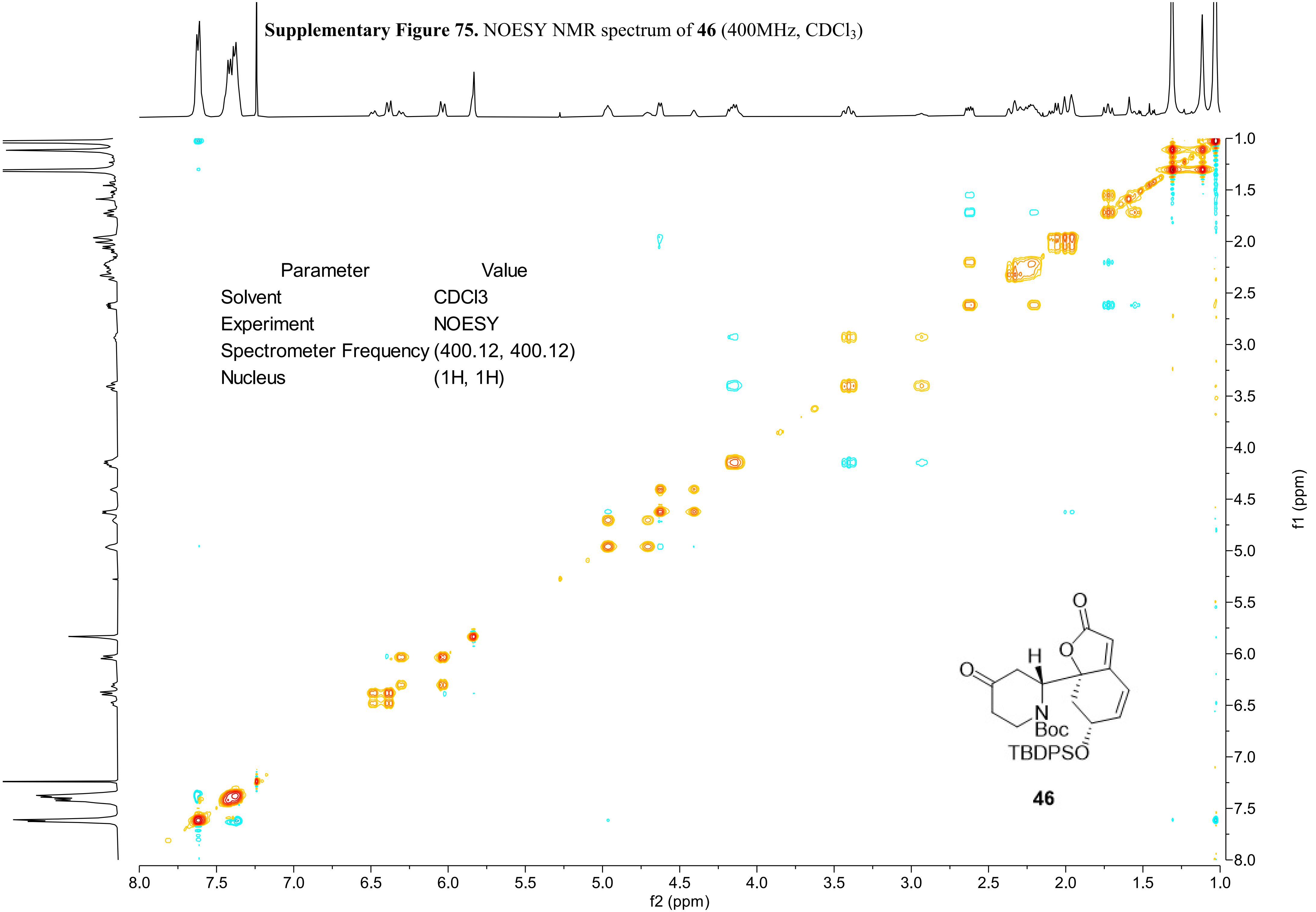

Supplementary Figure 76. ^{1}H NMR spectrum of **47** (400MHz, $CDCl_3$)

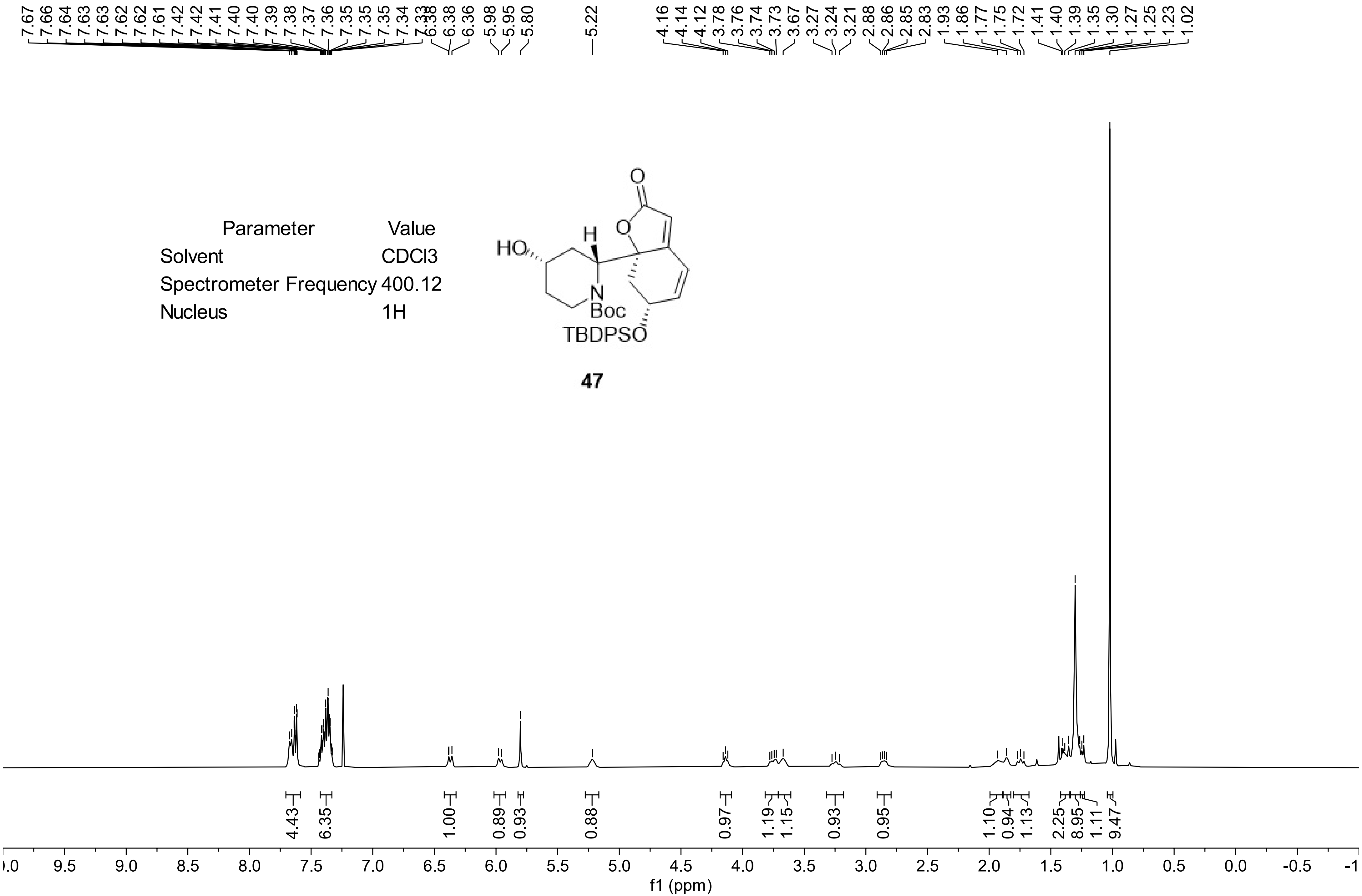

Parameter	Value
Solvent	CDCl3
Spectrometer Frequency	400.12
Nucleus	1H

Supplementary Figure 77. ^{13}C NMR spectrum of **47** (101MHz, $CDCl_3$)

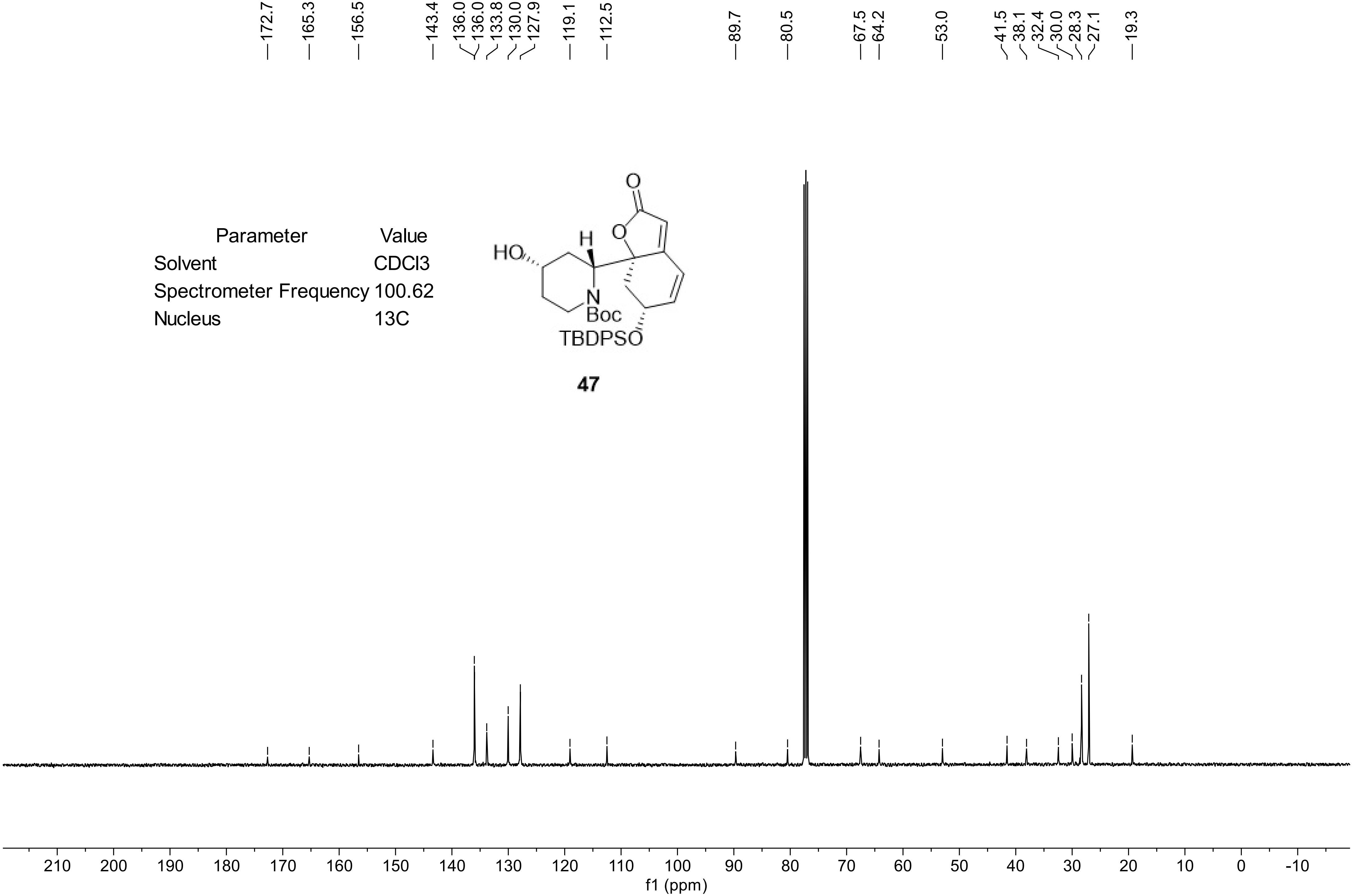

Supplementary Figure 78. NOESY NMR spectrum of **47** (400MHz, $CDCl_3$)

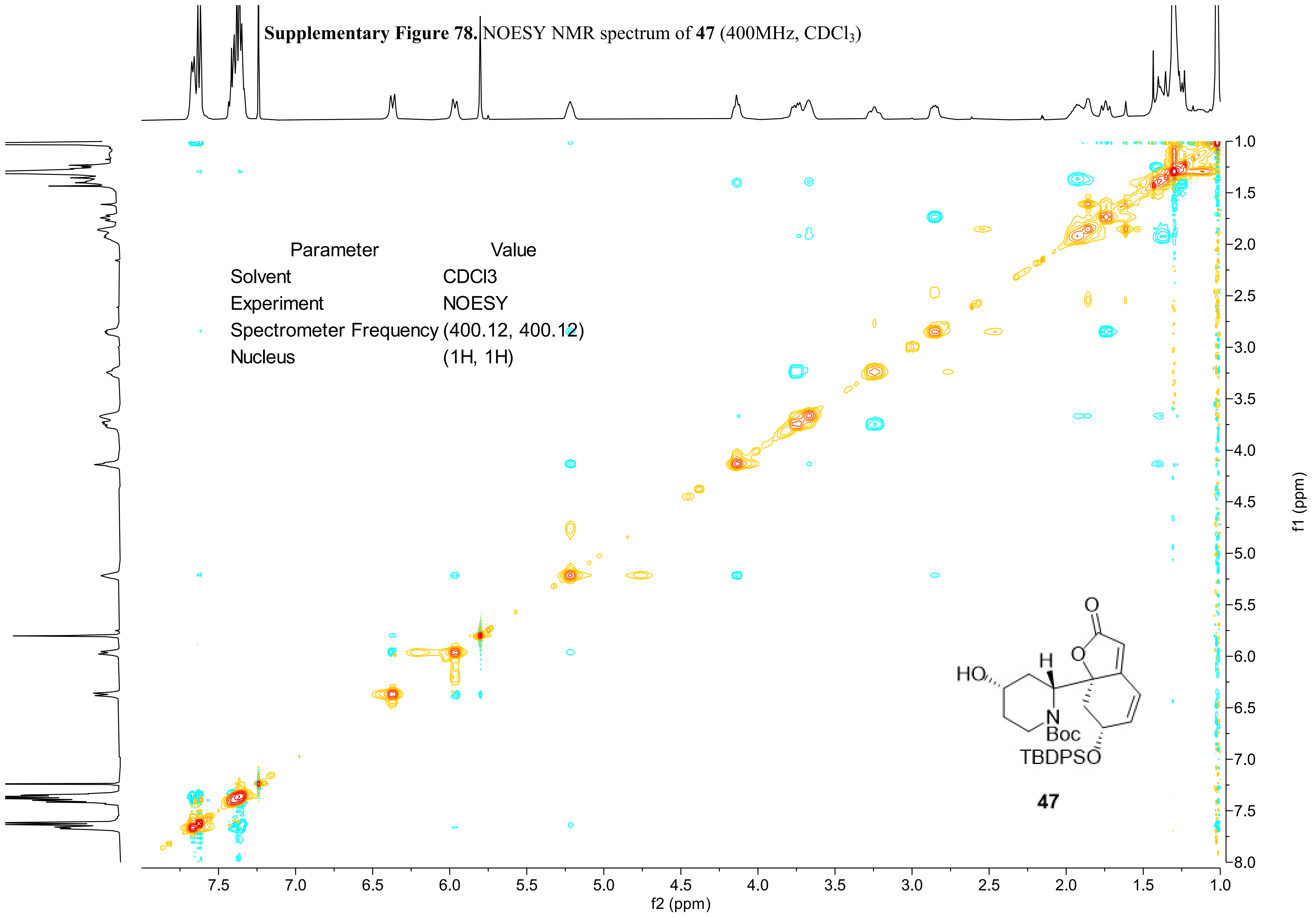

Parameter	Value
Solvent	CDCl3
Experiment	NOESY
Spectrometer Frequency	(400.12, 400.12)
Nucleus	(1H, 1H)

Supplementary Figure 79. ^{1}H NMR spectrum of **48** (400MHz, $CDCl_3$)

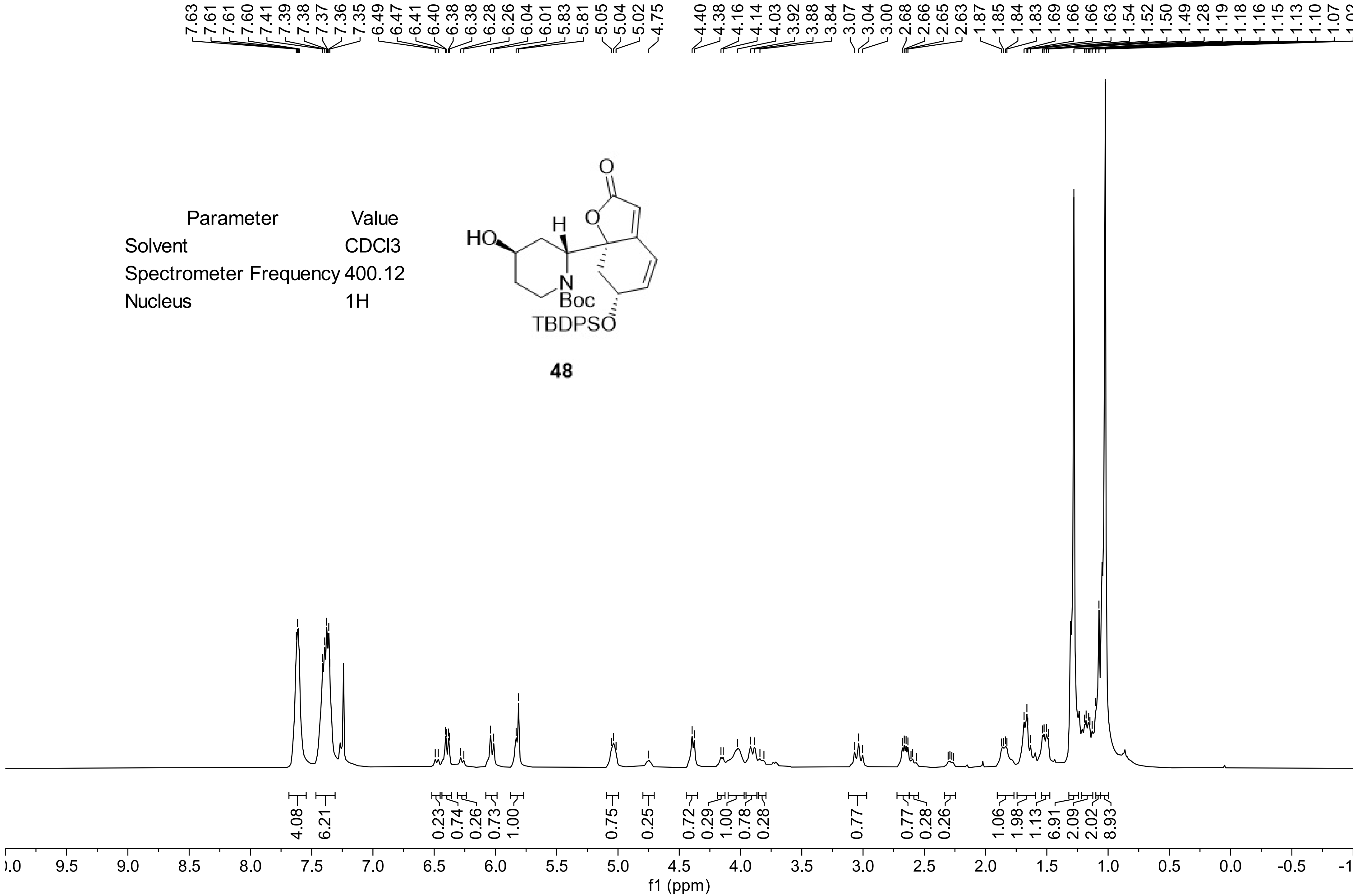

Supplementary Figure 80. ^{13}C NMR spectrum of **48** (126MHz, $CDCl_3$)

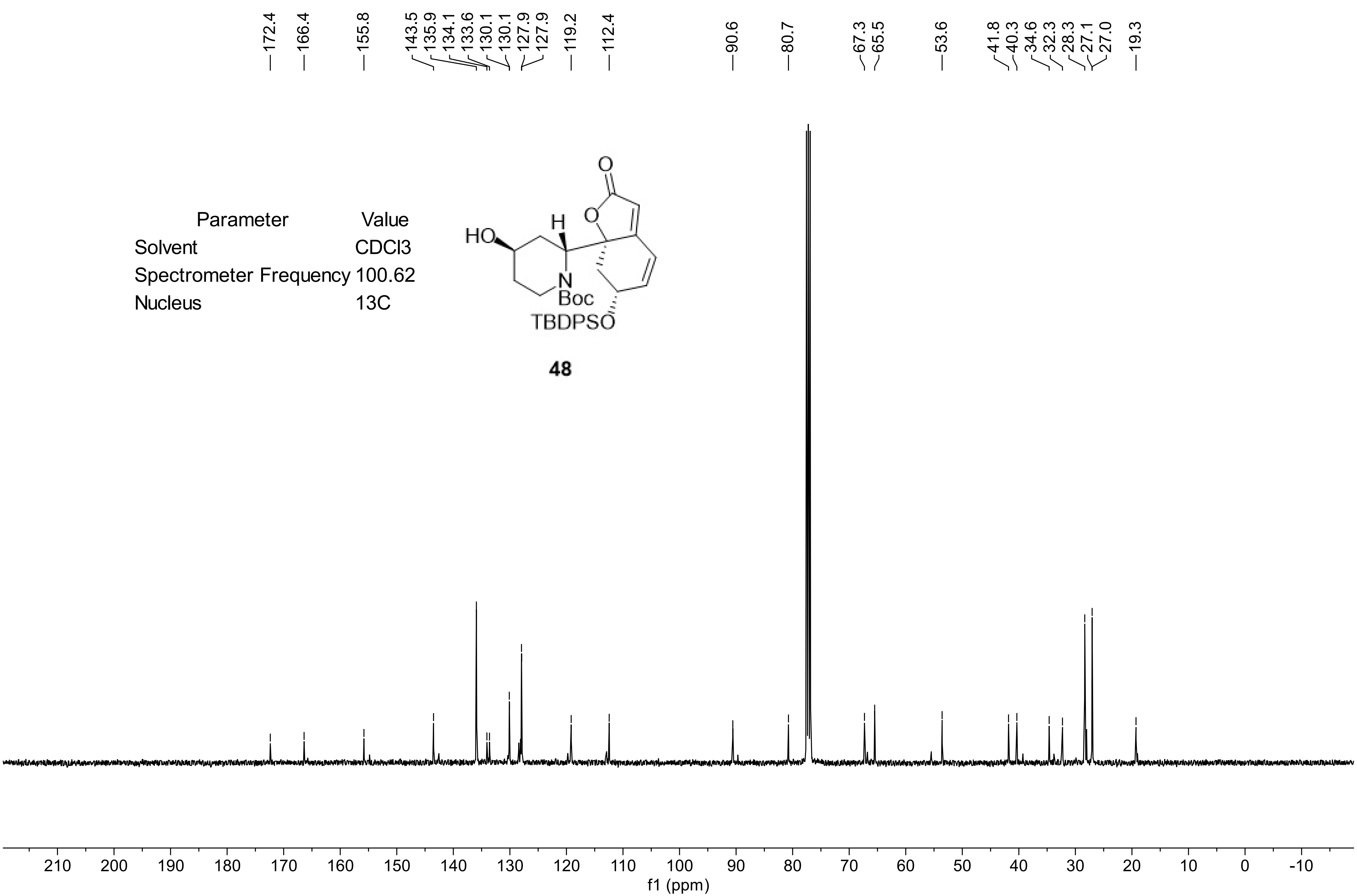

Supplementary Figure 81. NOESY NMR spectrum of **48** (400MHz, $CDCl_3$)

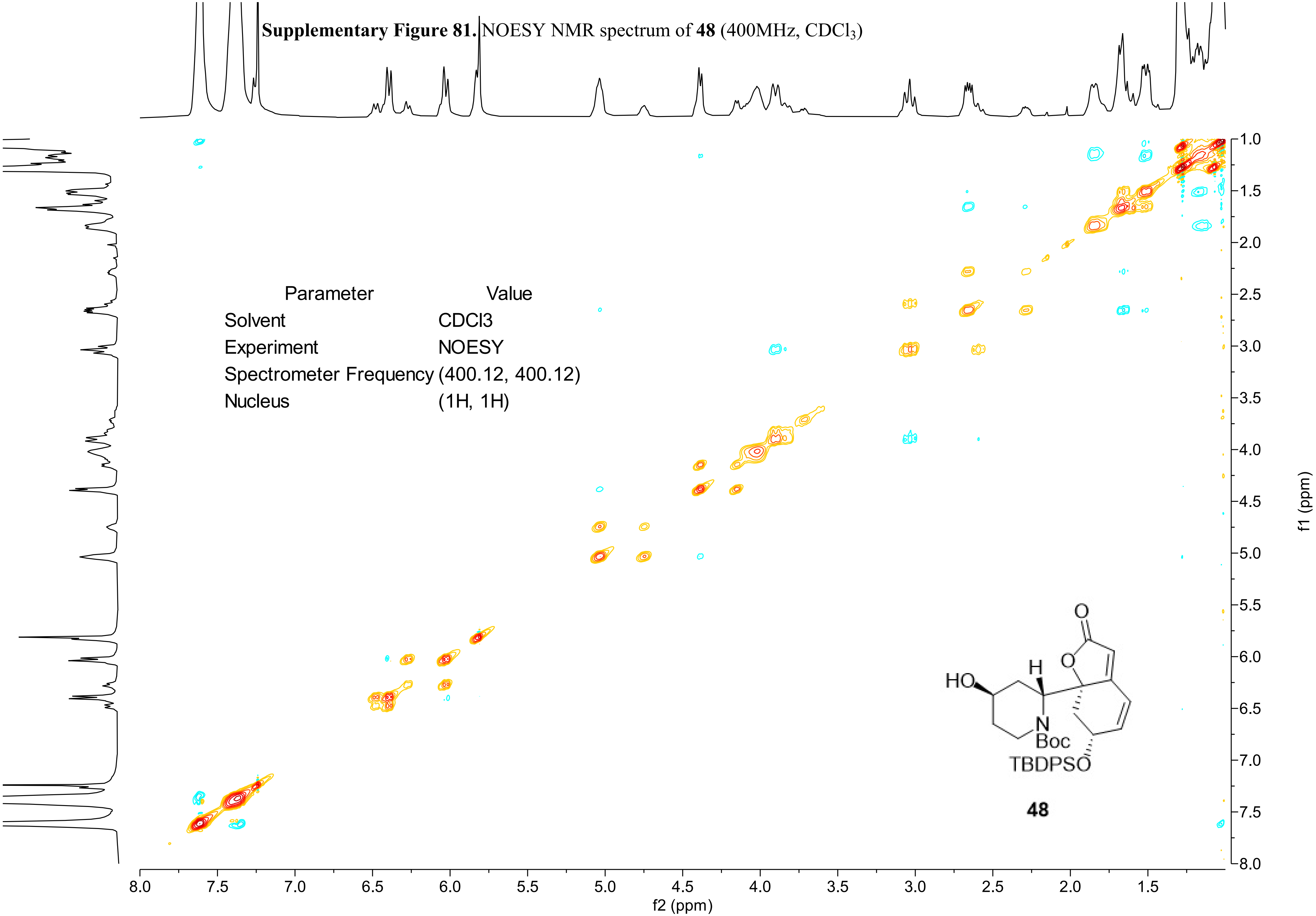

Supplementary Figure 82. ^{1}H NMR spectrum of **S6** (400MHz, $CDCl_3$)

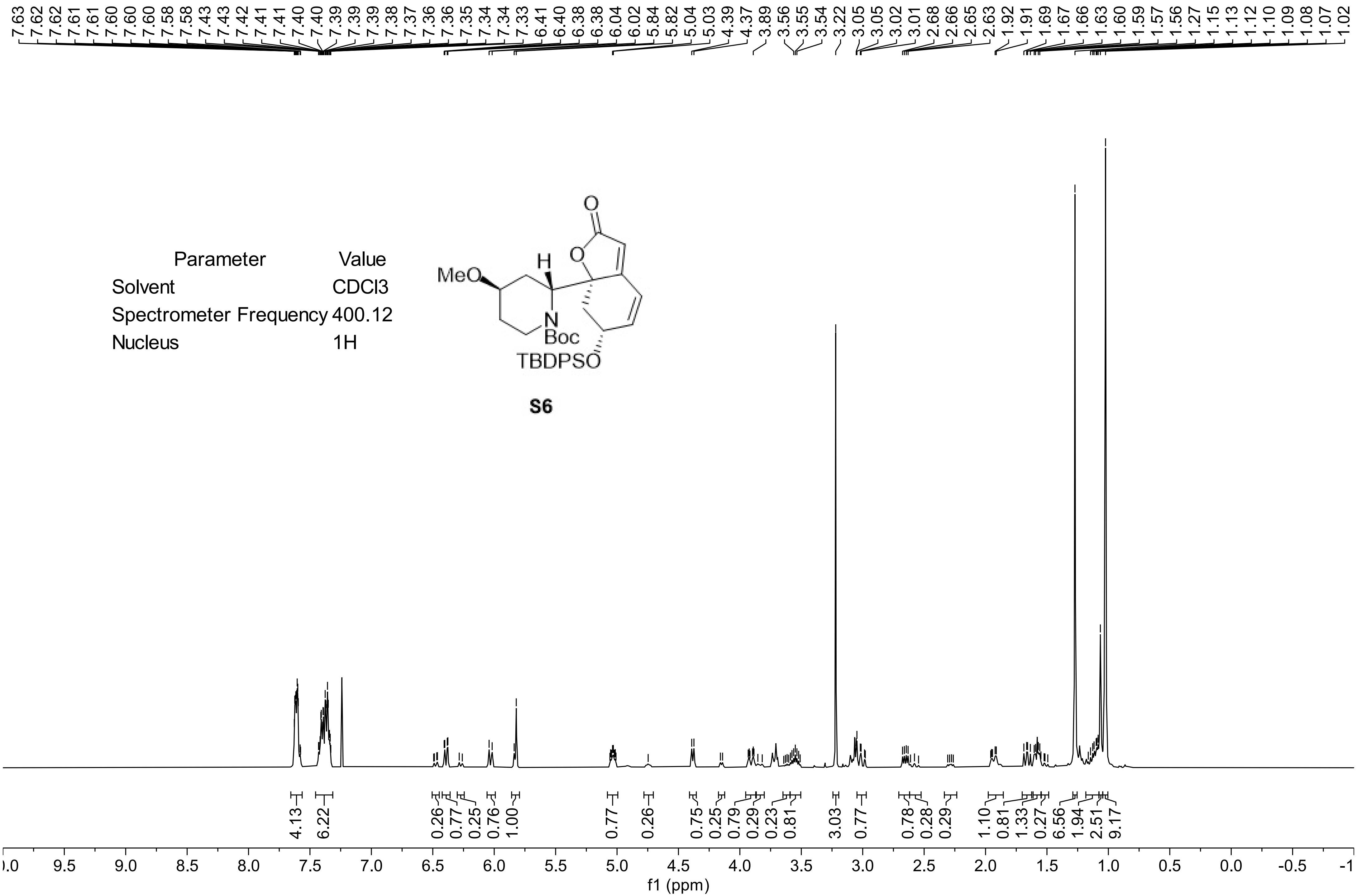

Parameter	Value
Solvent	CDCl3
Spectrometer Frequency	400.12
Nucleus	1H

S6

Supplementary Figure 83. ^{13}C NMR spectrum of **S6** (101MHz, $CDCl_3$)

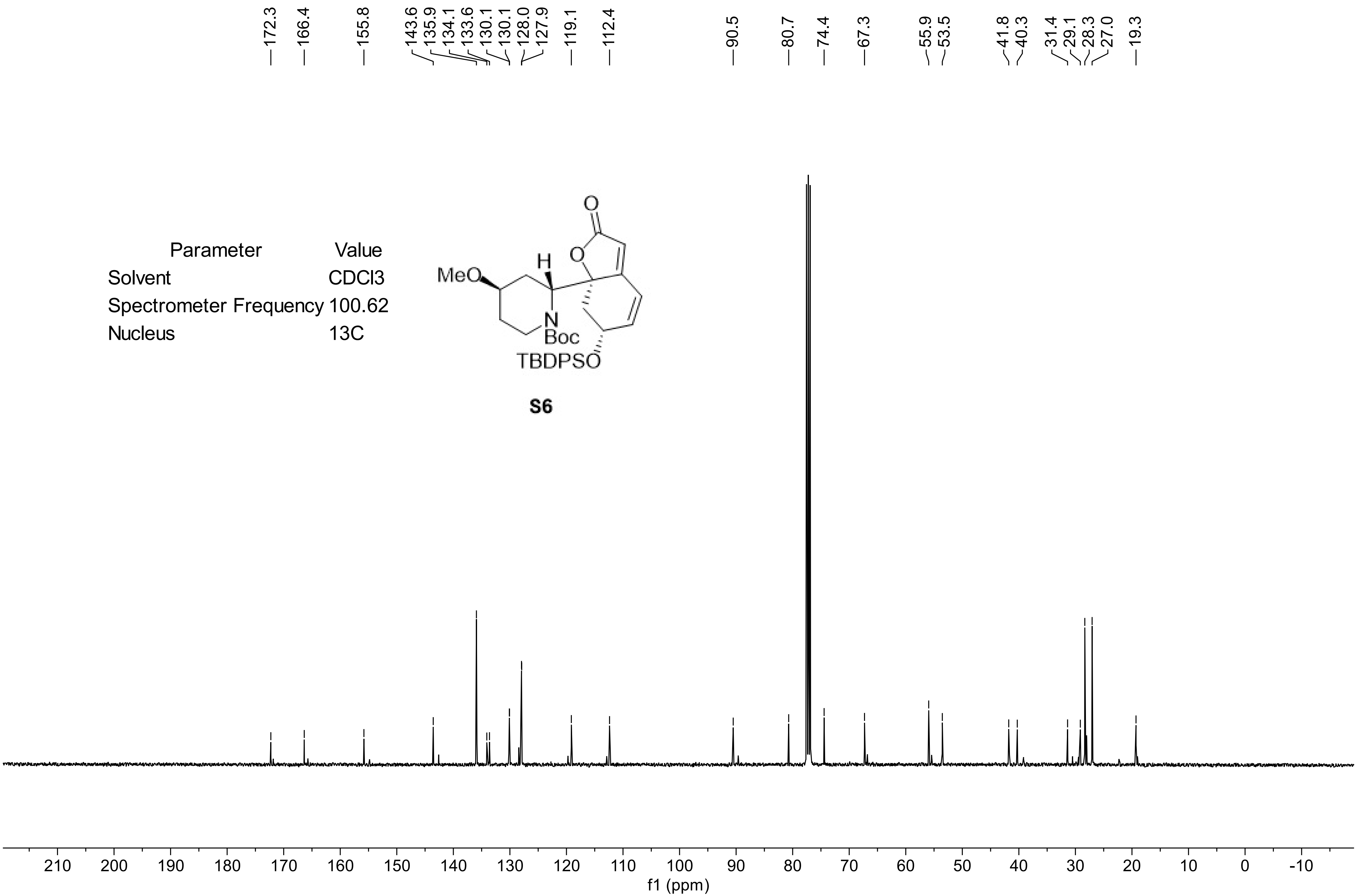

Supplementary Figure 84. NOESY NMR spectrum of **S6** (400MHz, $CDCl_3$)

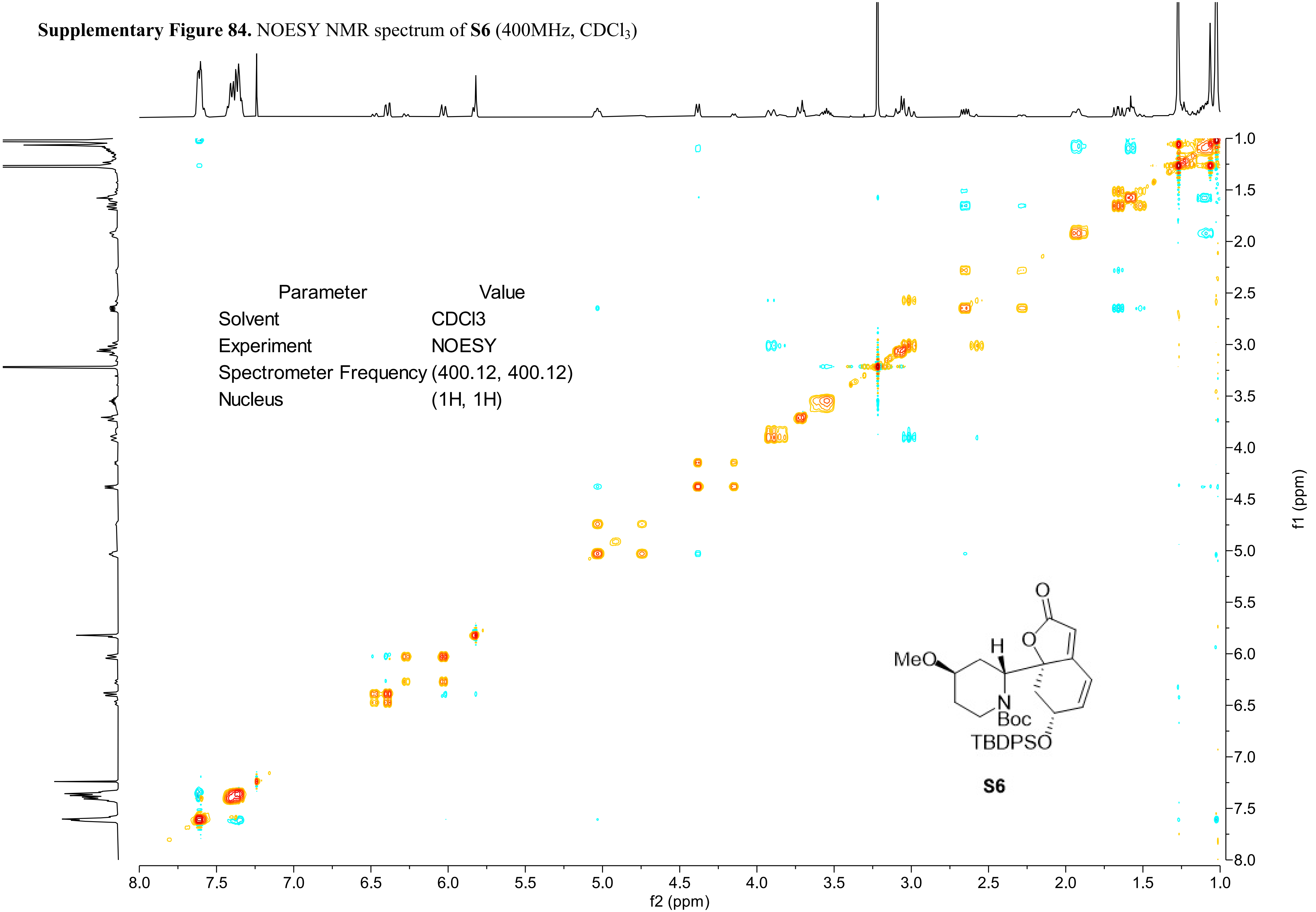

Supplementary Figure 85. ^{1}H NMR spectrum of **49** (400MHz, $CDCl_3$)

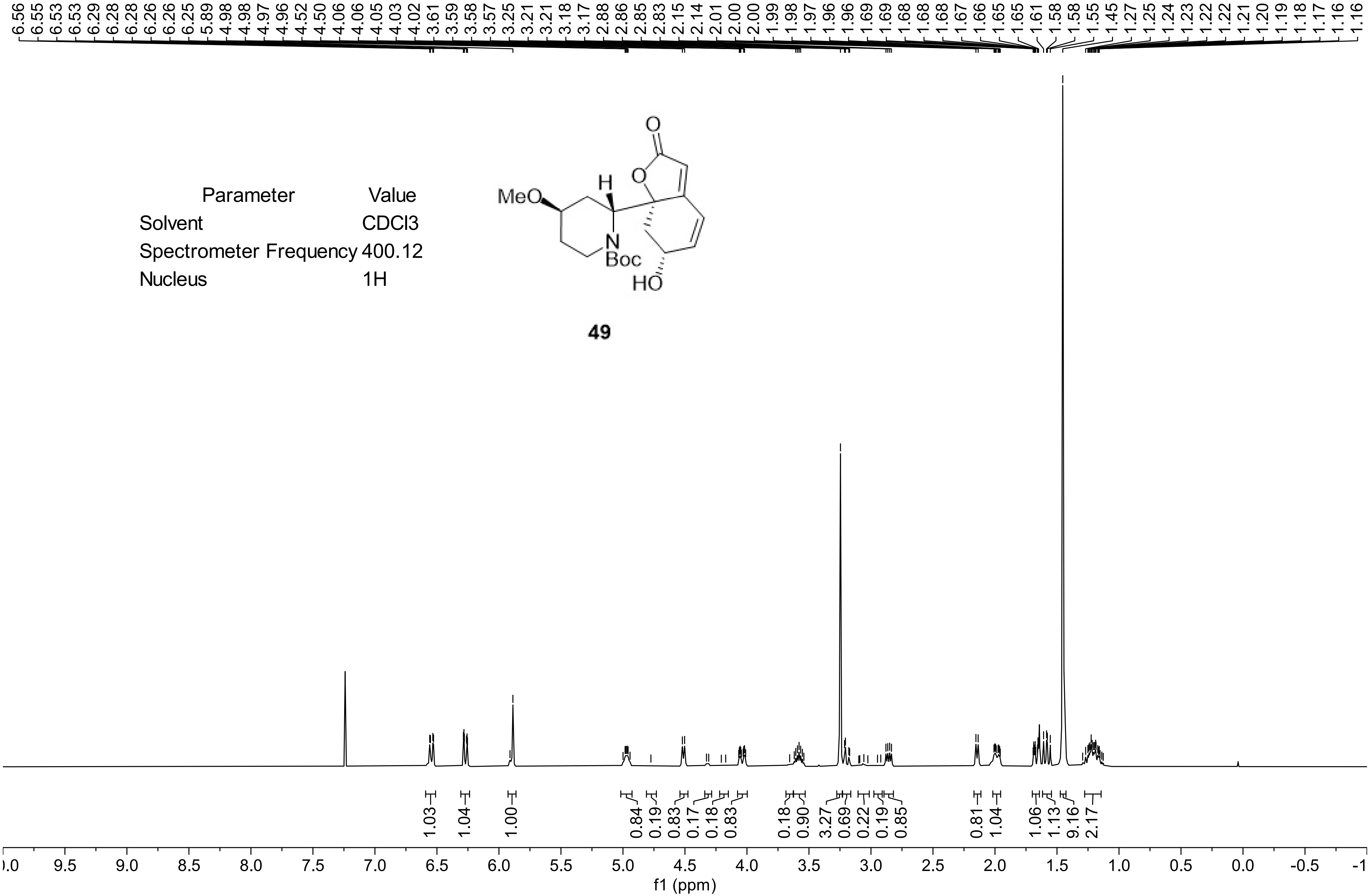

Supplementary Figure 86. ^{13}C NMR spectrum of **49** (101MHz, $CDCl_3$)

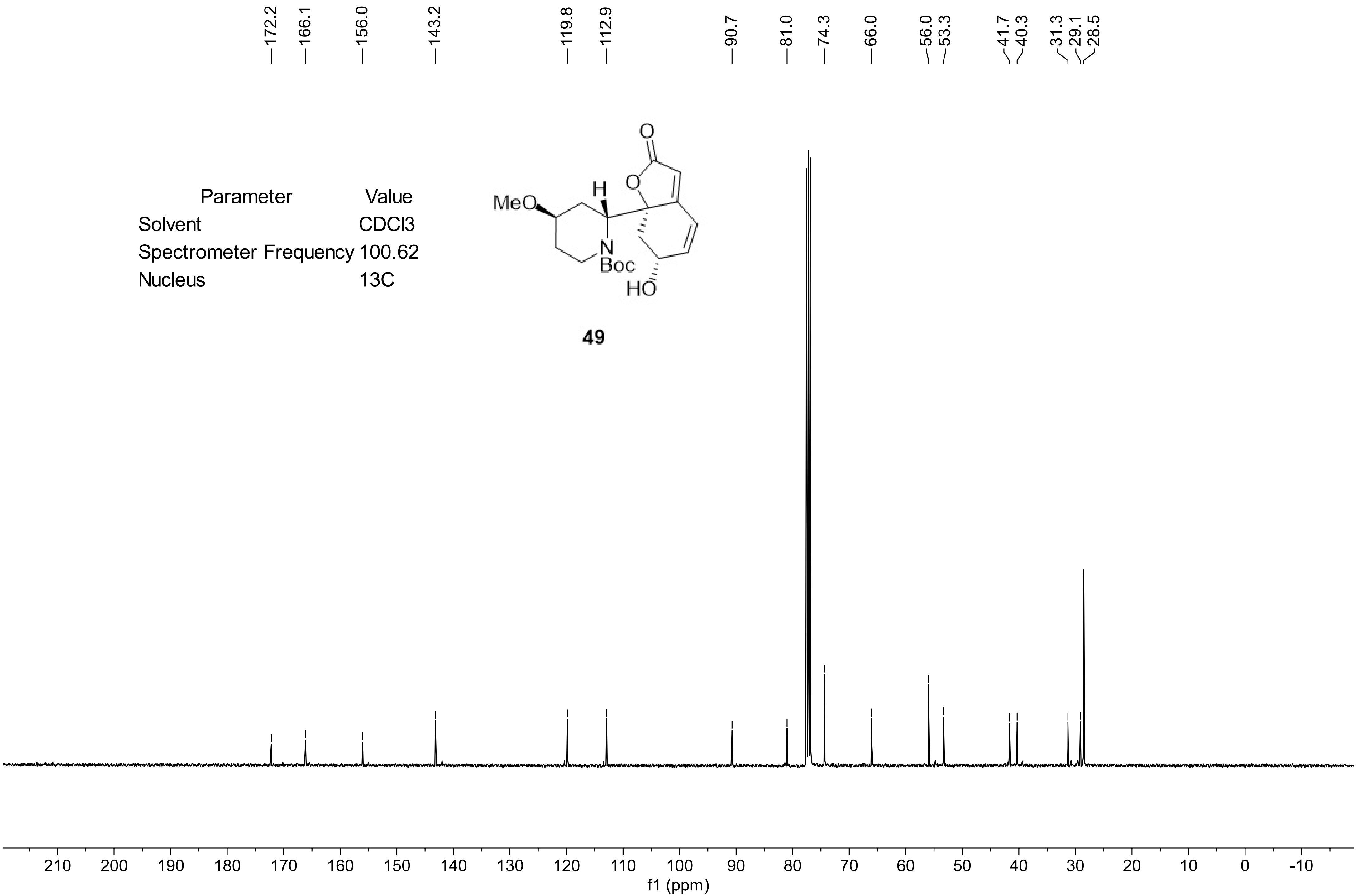

Supplementary Figure 87. NOESY NMR spectrum of **49** (400MHz, $CDCl_3$)

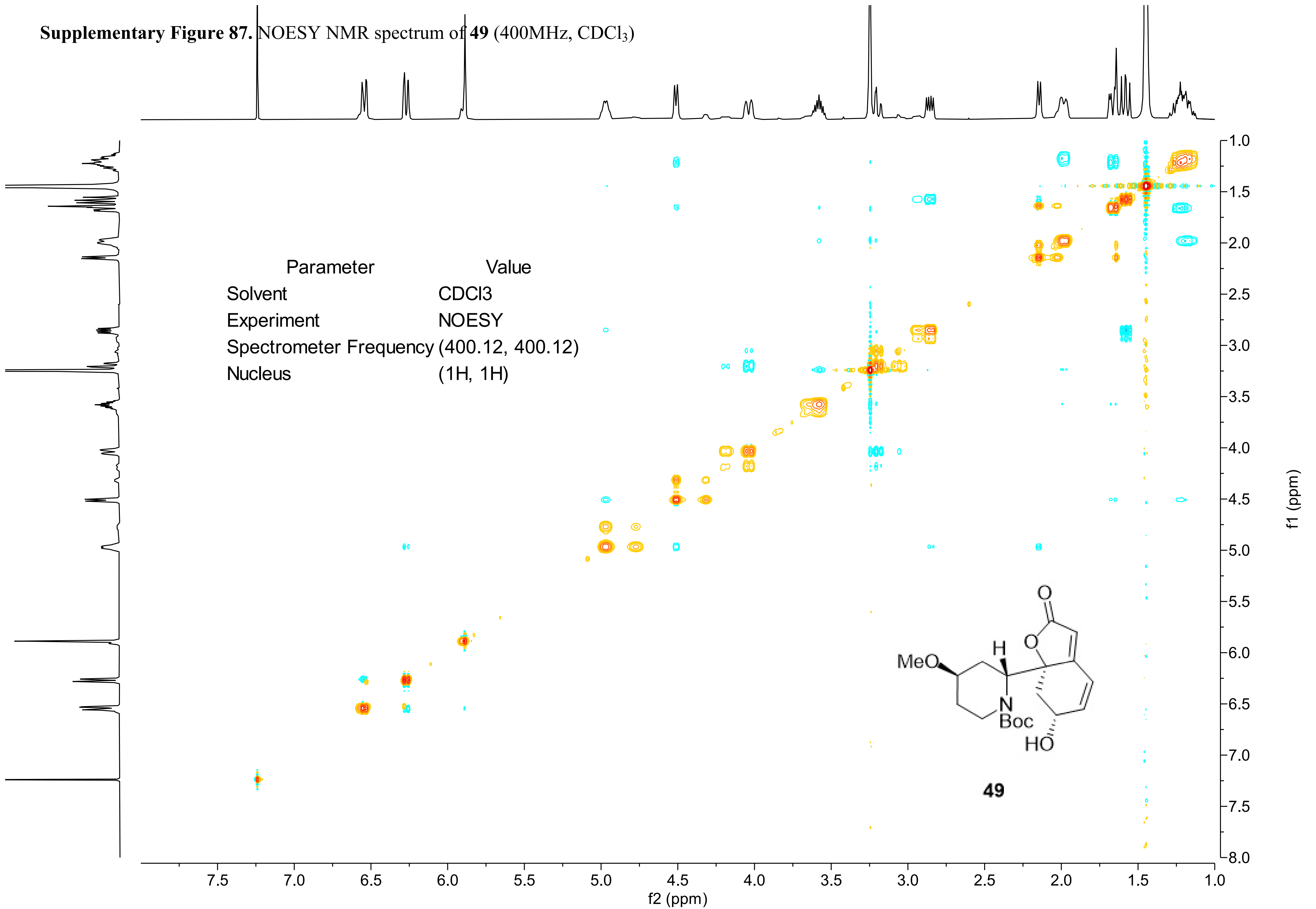

Supplementary Figure 88. ^{1}H NMR spectrum of **50** (400MHz, $CDCl_3$)

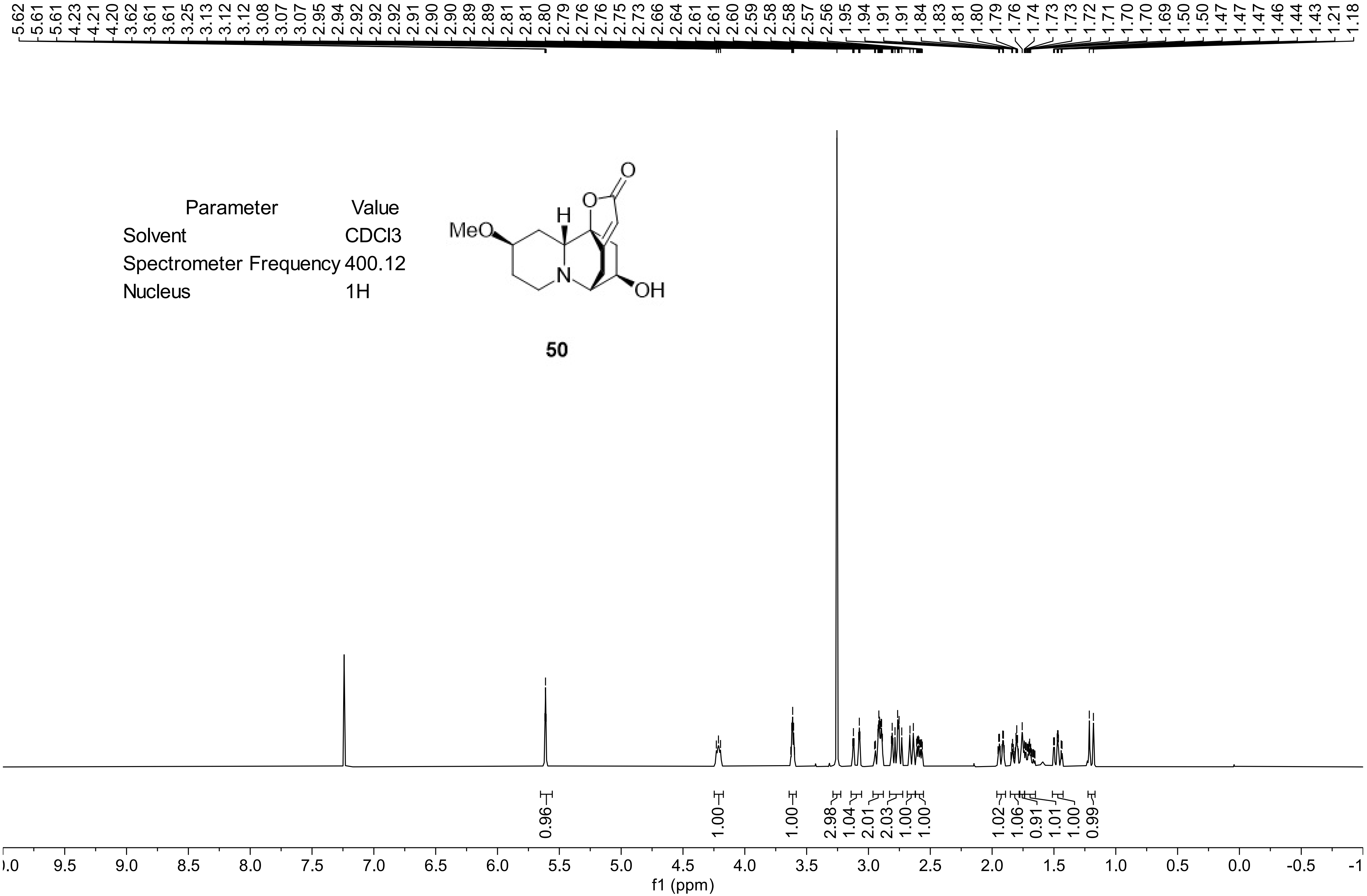

Supplementary Figure 89. ^{13}C NMR spectrum of **50** (101MHz, $CDCl_3$)

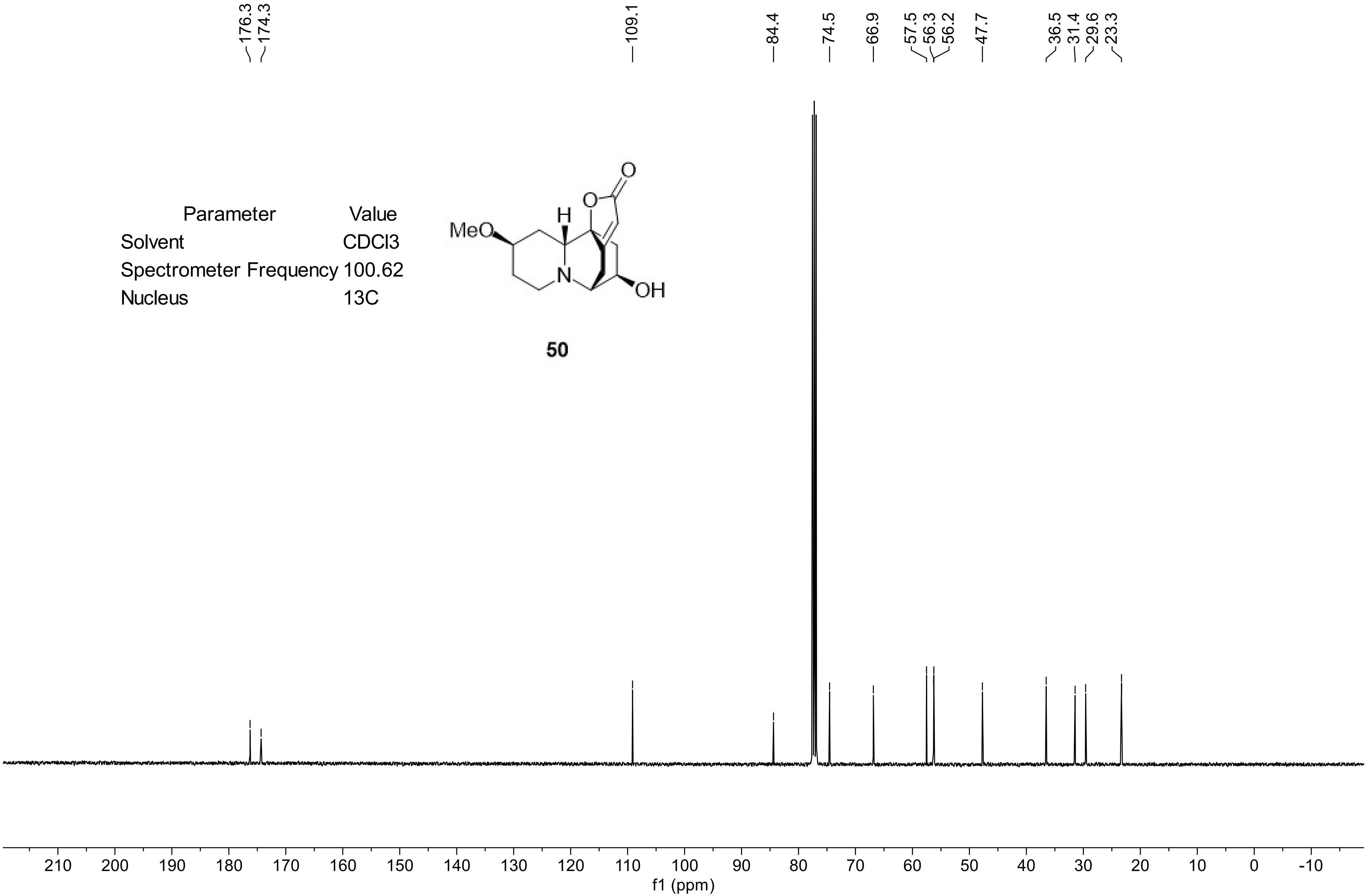

Supplementary Figure 90. ^{1}H NMR spectrum of phyllanthine (**5**) (400MHz, $CDCl_3$)

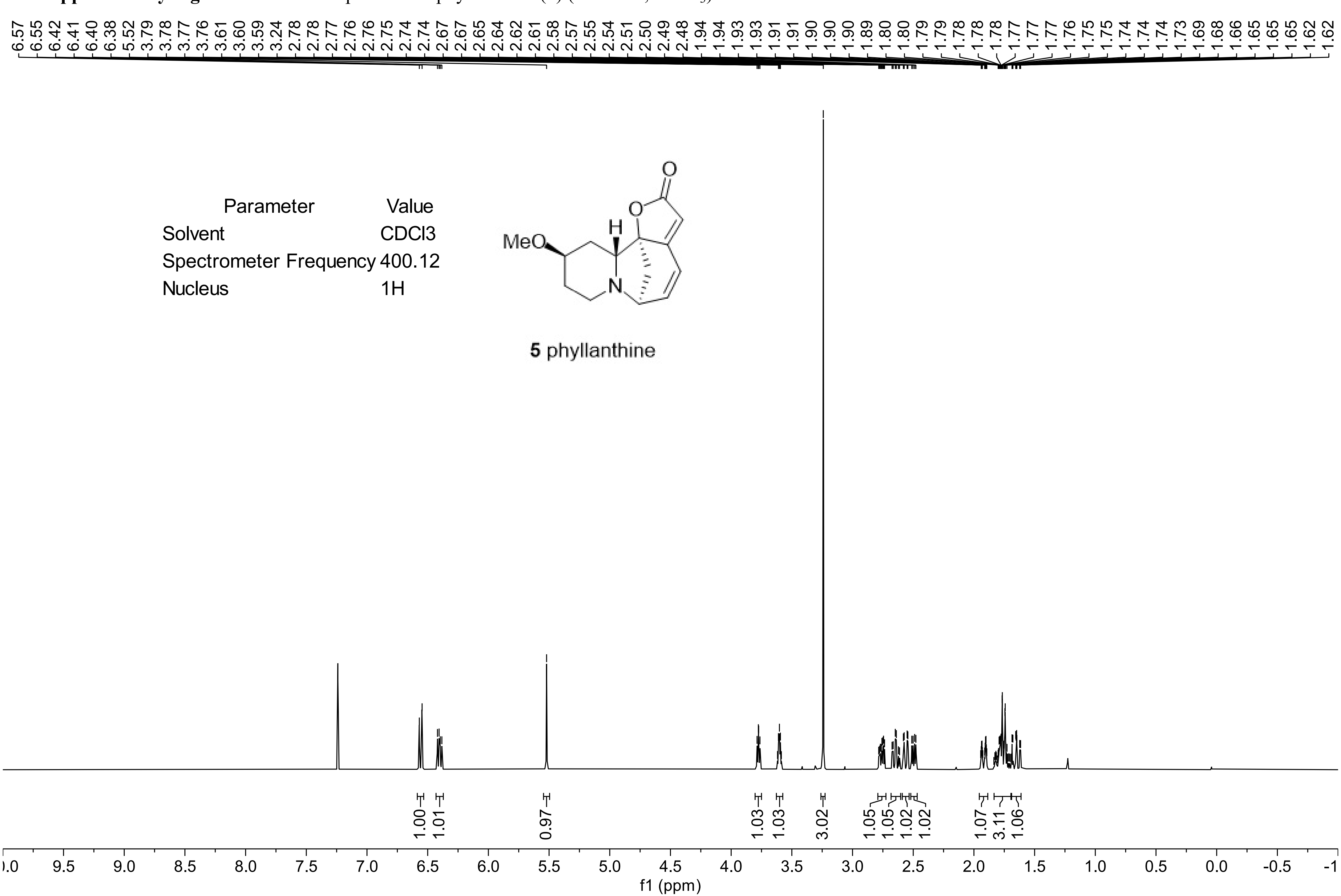

Supplementary Figure 91. ^{13}C NMR spectrum of phyllanthine (**5**) (101MHz, $CDCl_3$)

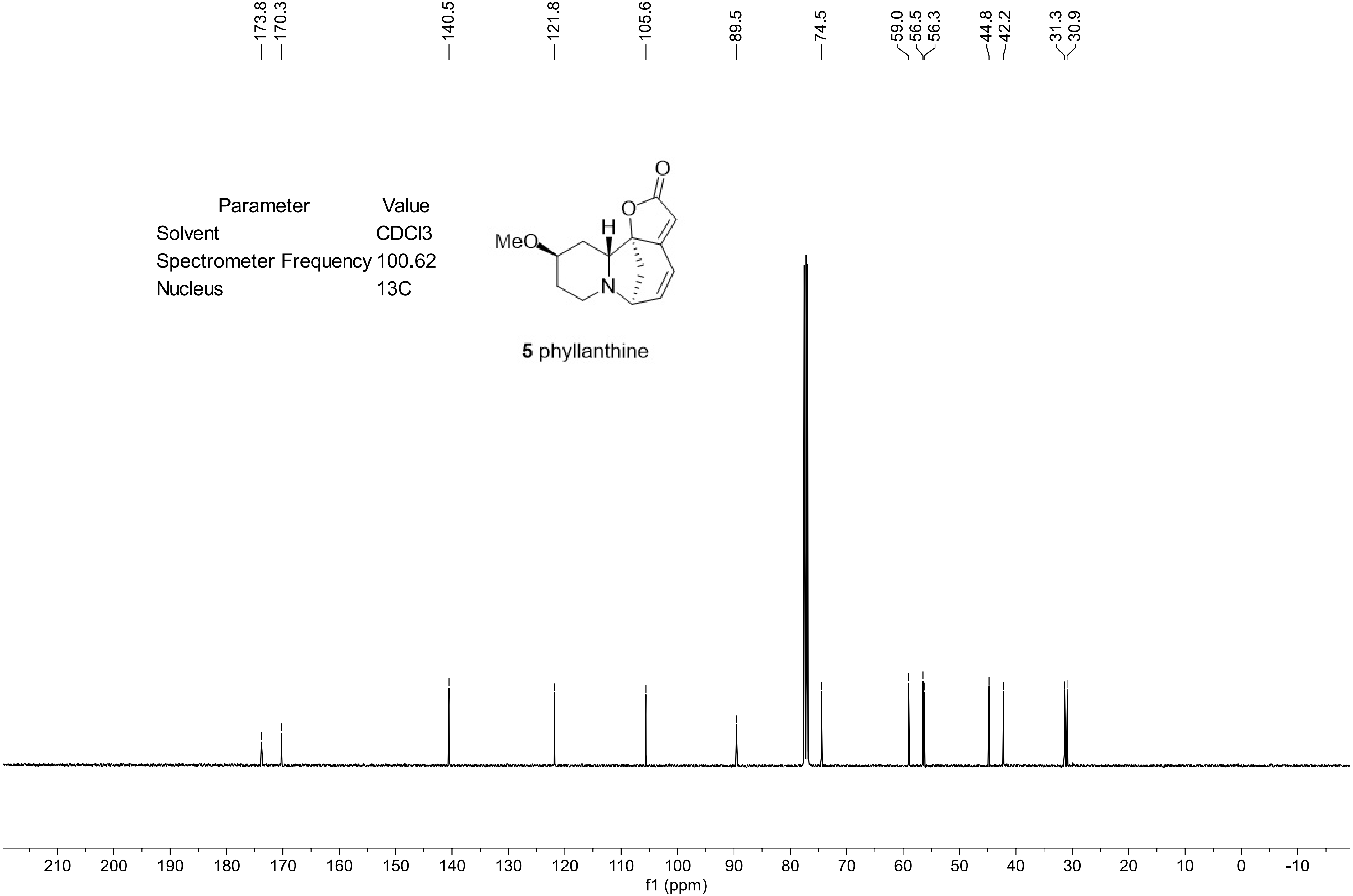

Supplementary Figure 92. ^{1}H NMR spectrum of **51** (400MHz, $CDCl_3$)

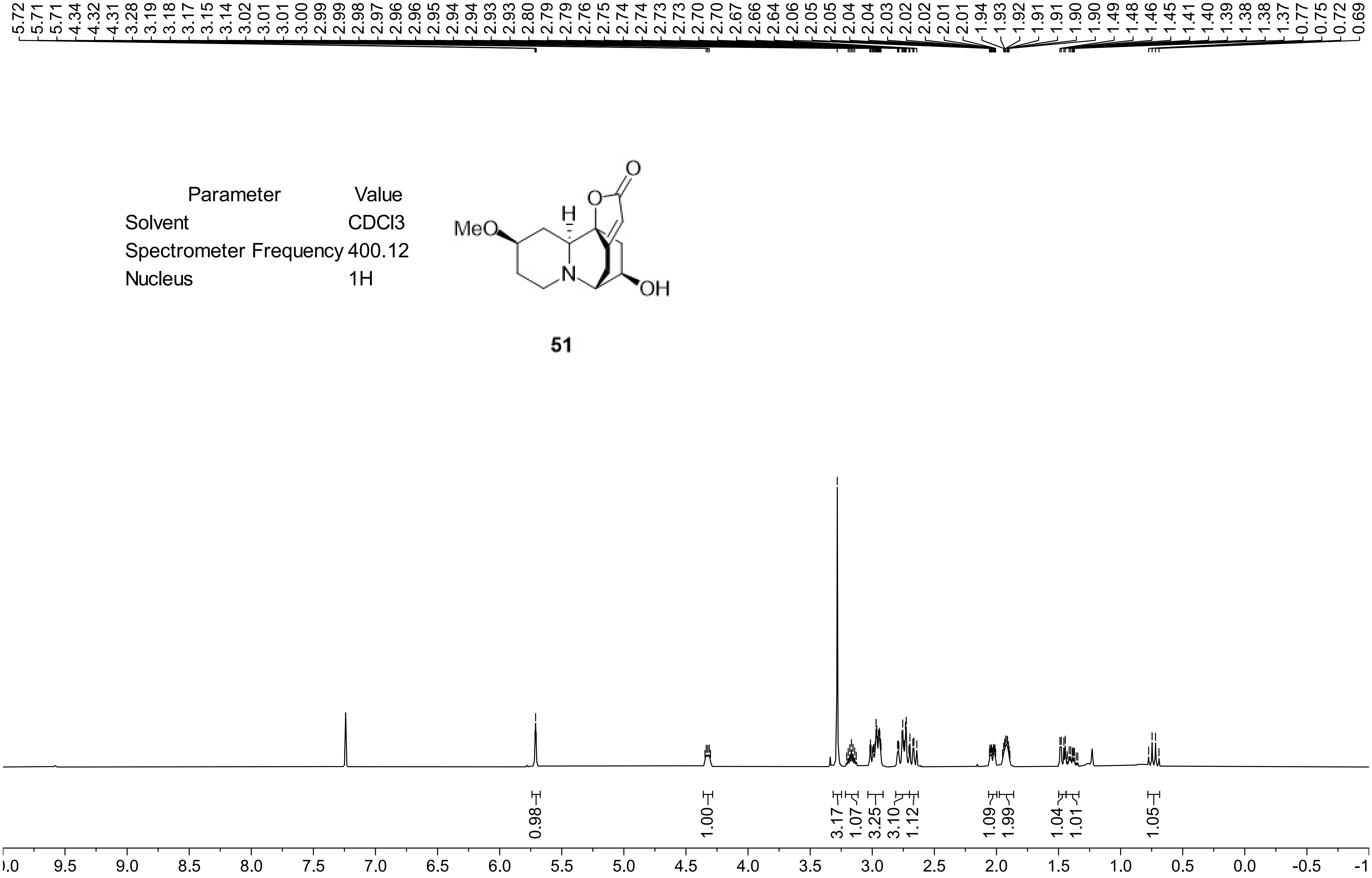

Supplementary Figure 93. ^{13}C NMR spectrum of **51** (101MHz, $CDCl_3$)

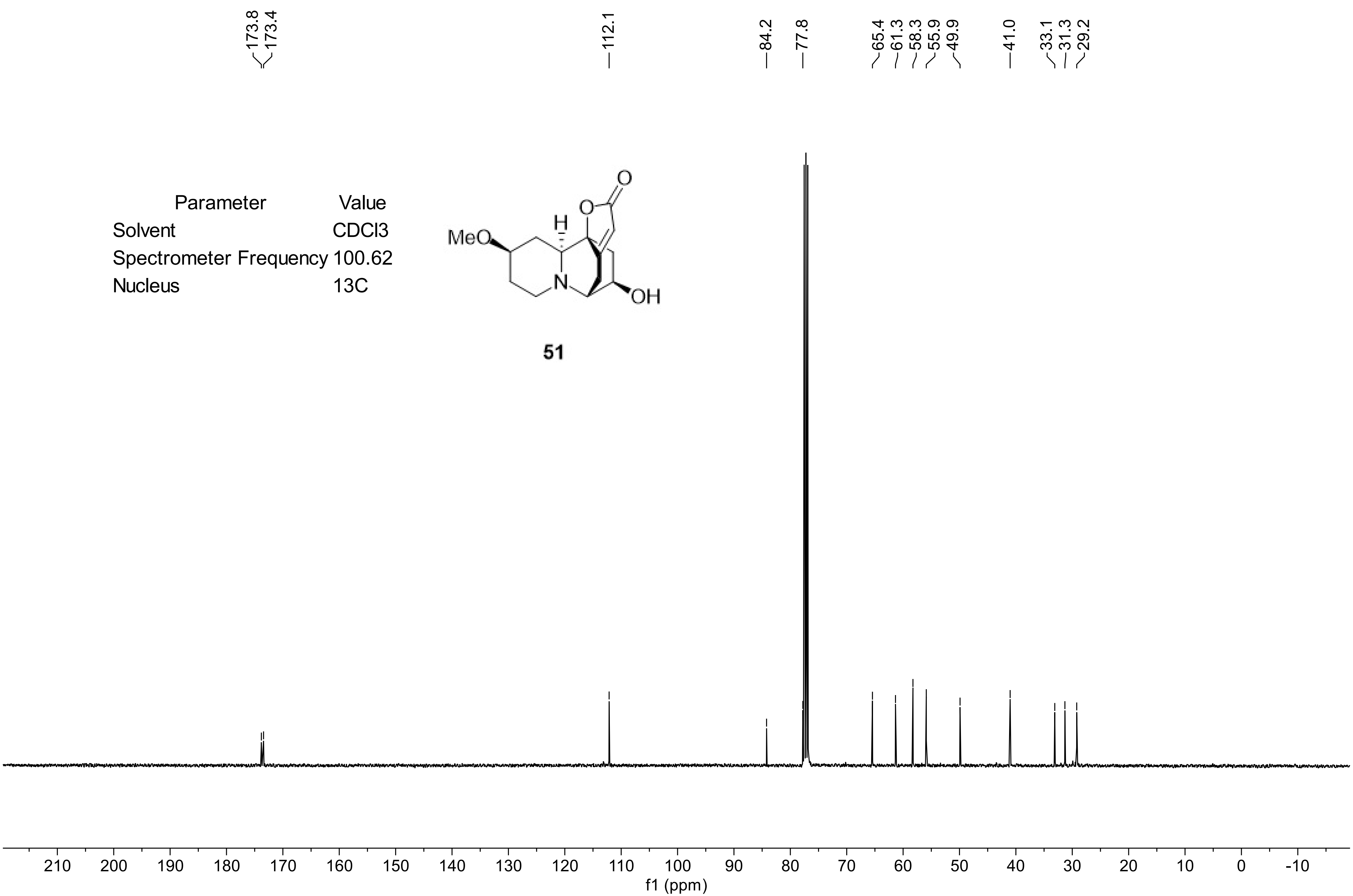

Supplementary Figure 94. ^{1}H NMR spectrum of 4-*epi*-securitinine (**52**) (400MHz, $CDCl_3$)

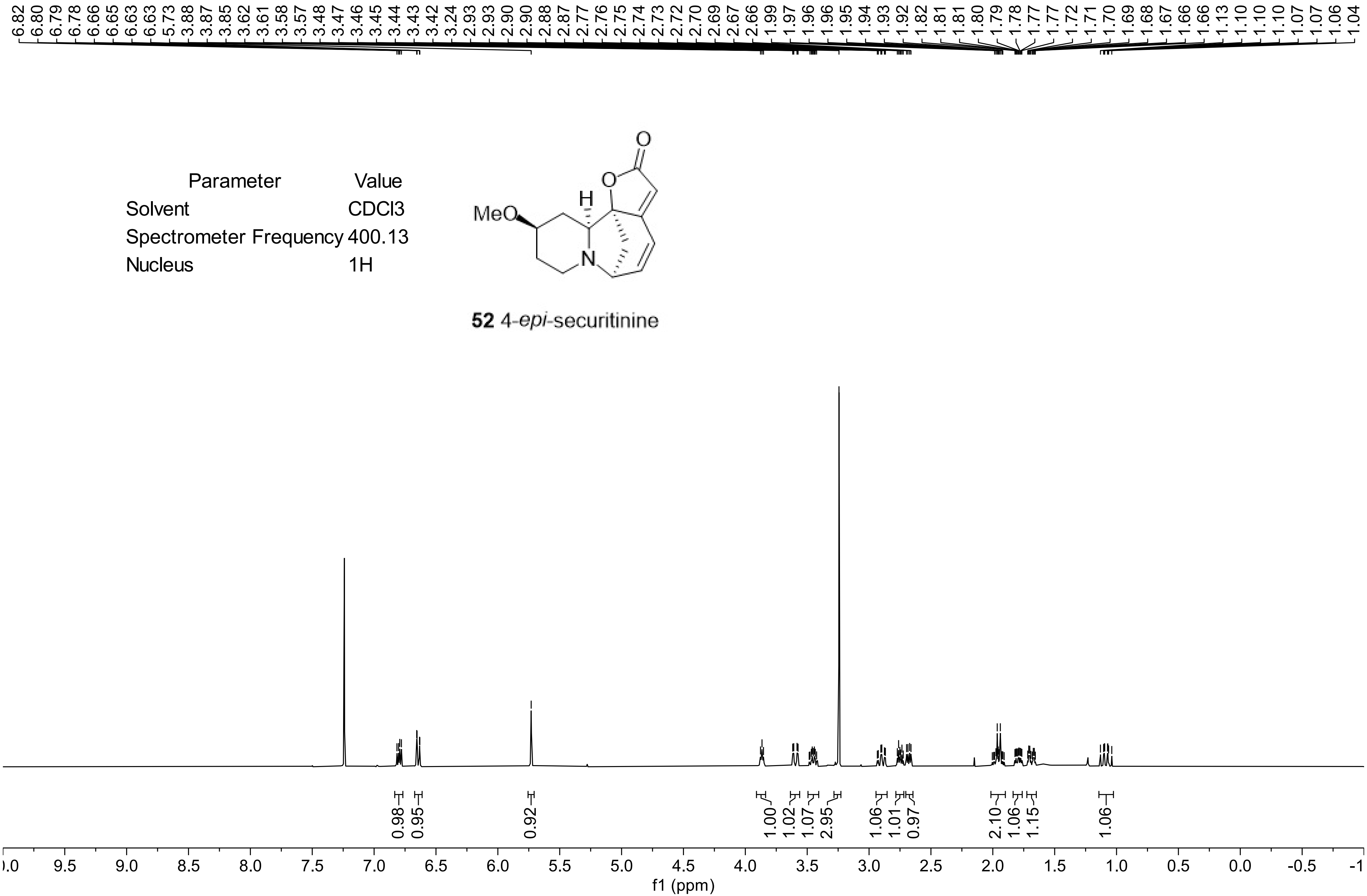

Supplementary Figure 95. ^{13}C NMR spectrum of 4-*epi*-securitinine (**52**) (101MHz, $CDCl_3$)

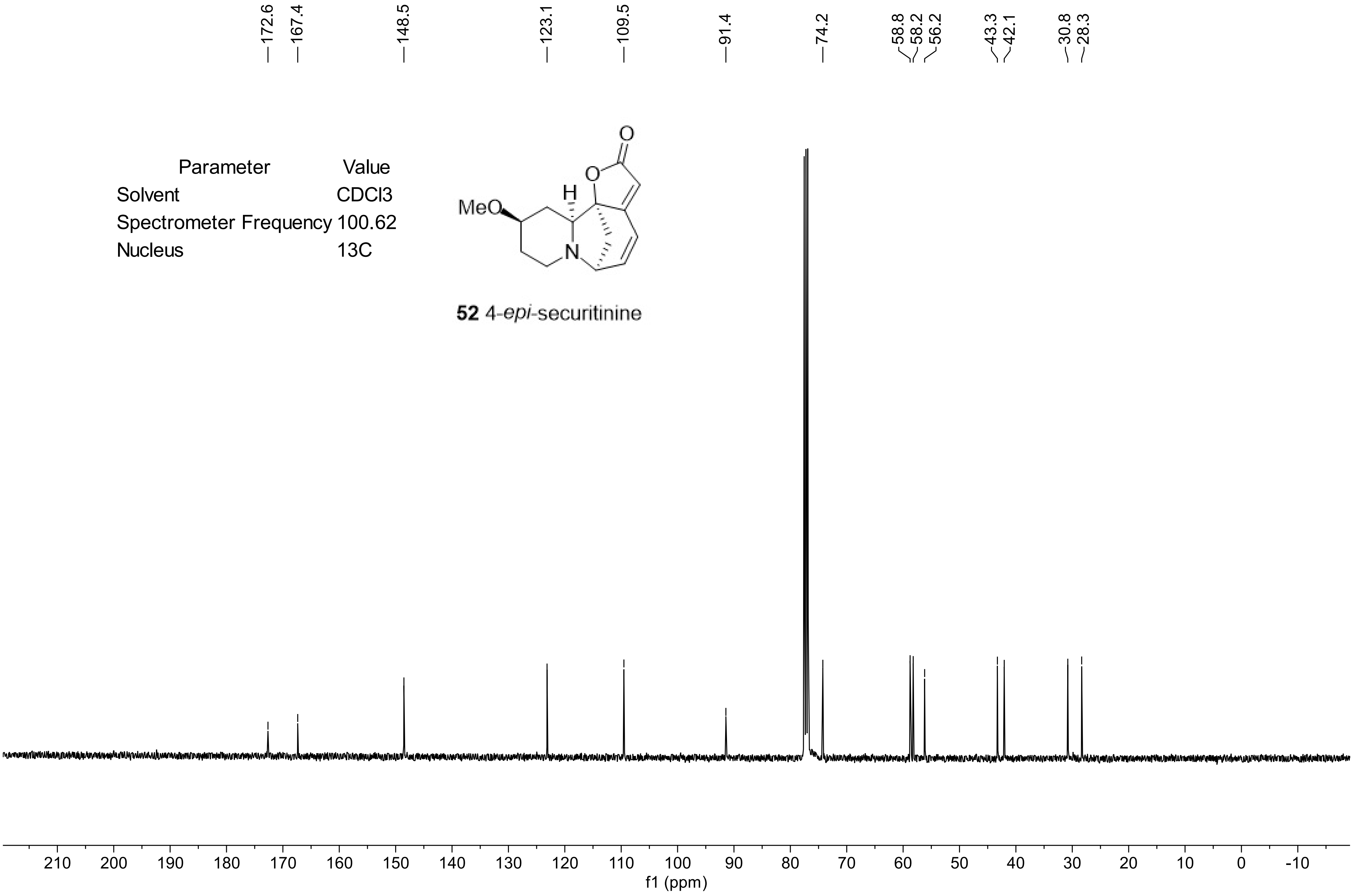

Supplementary References

(1) Still, W. C., Kahn, M. & Mitra, A. Rapid chromatographic technique for preparative separations with moderate resolution. *J. Org. Chem.* **43**, 2923–2925 (1978).

(2) Pangborn, A. B., Giardello, M. A., Grubbs, R. H., Rosen, R. K. & Timmers, F. J. Safe and convenient procedure for solvent purification. *Organometallics* **15**, 1518–1520 (1996).

(3) Park, K. J. *et al.* Securinega alkaloids from the twigs of *Securinega suffruticosa* and their biological activities. *J. Nat. Prod.* **82**, 1345–1353 (2019).

(4) Horii, Z., Ikeda, M., Hanaoka, M., Yamauchi, M. & Tamura, Y. Structure of Securitinine. *Chem. Pharm. Bull.* **15**, 1633–1640 (1967).

(5) Ohsaki, A., Kobayashi, Y., Yoneda, K., Kishida, A. & Ishiyama, H. Securinega alkaloids from the wood of *Securinega suffruticosa* var. *amamiensis*. *J. Nat. Prod.* **70**, 2003–2005 (2007).

(6) Arbain, D. *et al.* The alkaloids of *Margaritaria indica*. Part 2. The structures of 4-epiphyllanthine, margaritarine and the structural revision of securinol A. *J. Chem. Soc. Perkin Trans. 1.* 1863–1869 (1991).

(7) Jang, S. & Kim, H. Chiral ^{1}H NMR analysis of carbonyl compounds enabled by cationic cobalt complex. *Org. Lett.* **22**, 4185–4189 (2020).

(8) Han, G., LaPorte, M. G., Folmer, J. J., Werner, K. M. & Weinreb, S. M. Total syntheses of the *Securinega* alkaloids (+)-14,15-dihydronorsecurinine, (−)-norsecurinine, and phyllanthine. *J. Org. Chem.* **65**, 6293–6306 (2000).

(9) Parr, R. G. & Yang, W. *Density functional theory of atoms and molecules* (Oxford Univ. Press, New York, 1989).

(10) Bochevarov, A. D. *et al.* Jaguar: A high-performance quantum chemistry software program with strengths in life and materials sciences. *Int. J. Quantum Chem.* **113**, 2110–2142 (2013).

(11) Zhao, Y. & Truhlar, D. G. The M06 suite of density functionals for main group thermochemistry, thermochemical kinetics, noncovalent interactions, excited states, and transition elements: two new functionals and systematic testing of four M06-class functionals and 12 other functionals. *Theor. Chem. Acc.* **120**, 215–241 (2008).

(12) Dunning, T. H. Gaussian basis sets for use in correlated molecular calculations. I. The atoms boron through neon and hydrogen. *J. Chem. Phys.* **90**, 1007–1023 (1989).

(13) Marten, B. *et al.* New model for calculation of solvation free energies: Correction of self-consistent reaction field continuum dielectric theory for short-range hydrogen-bonding effects. *J. Phys. Chem.* **100**, 11775–11788 (1996).

(14) Friedrichs, M., Zhou, R., Edinger, S. R. & Friesner, R. A. Poisson−Boltzmann analytical gradients for molecular modeling calculations. *J. Phys. Chem. B* **103**, 3057–3061 (1999).

(15) Edinger, S. R., Cortis, C., Shenkin, P. S. & Friesner, R. A. Solvation free energies of peptides: Comparison of approximate continuum solvation models with accurate solution of the Poisson−Boltzmann equation. *J. Phys. Chem. B* **101**, 1190–1197 (1997).

(16) Rashin, A. A. & Honig, B. Reevaluation of the born model of ion hydration. *J. Phys. Chem.* **89**, 5588–5593 (1985).
