# Supplementary material for: Collective total synthesis of C4-oxygenated securinine-type alkaloids via stereocontrolled diversifications on the piperidine core
Source: Nat Commun. 2022 Sep 2;13:5149. doi: 10.1038/s41467-022-32902-z (PMC9440219; doi:10.1038/s41467-022-32902-z)
Supplement: Supplementary file 3 — Description of Additional Supplementary Files [file 41467_2022_32902_MOESM3_ESM.docx]

**Description of Additional Supplementary Files**

**File Name:** Supplementary Data 1

**Description:** Cartessian Coordinates and Vibrational Frequencies.
