# Supplementary material for: Collective total synthesis of C4-oxygenated securinine-type alkaloids via stereocontrolled diversifications on the piperidine core
Source: Nat Commun. 2022 Sep 2;13:5149. doi: 10.1038/s41467-022-32902-z (PMC9440219; doi:10.1038/s41467-022-32902-z)
Supplement: Supplementary file 4 — Supplementary Data 1 [file 41467_2022_32902_MOESM4_ESM.pdf]

Cartesian coordinates of the optimized geometries

The Cartesian coordinates of optimized geometries are given below in the standard XYZ format, and units are in Å.

===

Compound 30

===

C	-7.561280251	1.221169949	2.331205845
C	-8.468633652	0.041181866	2.689749718
C	-9.262335777	-0.455722272	1.475544930
N	-8.310274124	-0.750833809	0.420226097
C	-7.608533382	0.460259110	-0.014876123
C	-6.683869839	0.900196850	1.108543873
C	-8.673611641	-1.627013683	-0.690361142
C	-9.203442574	-0.817685604	-1.898062468
C	-8.077070236	0.106185354	-2.417808533
C	-6.961562634	0.114103429	-1.367630363
C	-6.372217178	-1.269430876	-1.315680623
C	-7.382843494	-2.358057499	-1.102494955
O	-9.586345673	-1.659242630	-2.969389677
O	-5.890869617	0.976473510	-1.688395739
C	-4.725549221	0.245525807	-1.771551013
C	-5.062400341	-1.183805108	-1.543868303
O	-3.669066429	0.759676933	-2.003974676
H	-8.320640564	1.289417028	-0.194244161
O	-8.430041313	2.313880444	2.078987837
C	-7.780072689	3.554571867	1.948198438
H	-6.917972565	1.466521740	3.192136526
H	-7.847432137	-0.781564176	3.057971239
H	-9.145627022	0.354813397	3.489753485
H	-10.002193451	0.308576465	1.179187536
H	-9.804668427	-1.371425390	1.733593345
H	-5.992260933	0.086161442	1.353926301
H	-6.091170311	1.768856525	0.808529794
H	-9.436841965	-2.336413622	-0.350713730
H	-10.055818558	-0.213007808	-1.554653168
H	-8.432574272	1.126105666	-2.586651087
H	-7.703216076	-0.293837756	-3.365023375
H	-7.062979221	-3.049566031	-0.319424063
H	-7.534038067	-2.920949459	-2.030877829
H	-10.369703293	-2.154039860	-2.704346895
H	-4.313162804	-1.960758567	-1.600995779
H	-8.554141998	4.324270725	1.948447824
H	-7.096518040	3.736605883	2.790250778
H	-7.209468365	3.634215832	1.014071226

===

Compound 42

===

C	-9.526927948	1.946158409	0.941412449
C	-10.563442230	0.873967052	1.310192227
C	-9.905400276	-0.471140712	1.640130401
N	-9.052383423	-0.840858757	0.528880179
C	-7.975303173	0.134207591	0.342651814
C	-8.573796272	1.445196033	-0.143465444
C	-8.614712715	-2.226151705	0.365126640
C	-8.494735718	-2.412918806	-1.165285587
C	-7.612031937	-1.272293448	-1.719953060
C	-6.933039188	-0.560222626	-0.545996308
C	-6.274012566	-1.586105943	0.339431345
C	-7.240895748	-2.521492243	1.004414082
O	-7.870195389	-3.636548281	-1.506086826
O	-5.899629116	0.307557762	-0.957470179
C	-4.696247578	-0.137908295	-0.452553928
C	-4.965247154	-1.344846368	0.374711841
O	-3.663218021	0.423716158	-0.677526176
H	-7.467552662	0.346502155	1.307159424
O	-10.134855270	3.123769760	0.458750039
C	-10.707962036	3.925271273	1.460728884
H	-8.928610802	2.185103416	1.840632558
H	-11.224780083	0.741758049	0.446951330
H	-11.171756744	1.202984333	2.159001589
H	-9.342782021	-0.393497944	2.590952635
H	-10.674646378	-1.238559127	1.774223685
H	-7.793984413	2.189686537	-0.325257123
H	-9.137421608	1.288928270	-1.069573760
H	-9.385534286	-2.887274265	0.776357234
H	-9.501411438	-2.348730326	-1.595959544
H	-8.195379257	-0.553639710	-2.300040245
H	-6.846915245	-1.705783248	-2.369004726
H	-7.268237591	-2.332747459	2.084199667

H	-6.953309059	-3.564429998	0.852504253
H	-8.456509590	-4.359376431	-1.254794836
H	-4.176668644	-1.854642272	0.910331488
H	-11.089181900	4.824599743	0.974280655
H	-11.541290283	3.426733971	1.973729849
H	-9.961462021	4.216859341	2.214213133

==

Compound 50

==

C	-9.443593025	2.285618544	1.182548404
C	-10.594126701	1.317284465	1.467658401
C	-10.093309402	-0.118571453	1.664778709
N	-9.308746338	-0.483826131	0.497618645
C	-8.123691559	0.371946543	0.372483045
C	-8.577883720	1.778380156	0.015311085
C	-9.027518272	-1.890945673	0.217013448
C	-8.950262070	-1.966699839	-1.325254321
C	-7.957563400	-0.886406720	-1.809635758
C	-7.184497833	-0.354233265	-0.599598229
C	-6.629512787	-1.516511440	0.181062192
C	-7.685193539	-2.385764122	0.798827291
O	-8.466524124	-3.218274117	-1.777239561
O	-6.066749573	0.426790267	-0.965831339
C	-4.913302898	-0.192282438	-0.533167064
C	-5.301421642	-1.427133679	0.198325291
O	-3.828283548	0.270143688	-0.739388227
H	-7.587049007	0.447173923	1.337736845
O	-8.706107140	2.372176647	2.390072584
C	-7.675957203	3.330478668	2.378446817
H	-9.847071648	3.281600237	0.936674178
H	-11.286377907	1.331717134	0.619603753
H	-11.125734329	1.661401033	2.359599590
H	-9.510664940	-0.182190582	2.600296736
H	-10.945474625	-0.801171124	1.748407483
H	-7.716934681	2.430283070	-0.157844529
H	-9.181389809	1.760333538	-0.898437560
H	-9.860106468	-2.495943785	0.592893422
H	-9.950250626	-1.760756969	-1.726290584
H	-8.469446182	-0.063224539	-2.313647509
H	-7.257515907	-1.344069839	-2.513193846
H	-7.673210621	-2.275362968	1.889530063

H	-7.519401550	-3.439719439	0.564641178
H	-9.116762161	-3.894528627	-1.555380464
H	-4.565994263	-2.064407110	0.668869555
H	-7.338122368	3.450697422	3.409348965
H	-6.820553303	3.019142389	1.764859915
H	-8.037581444	4.300179005	2.006463766

==

Compound 51

==

C	-7.491712570	0.924648166	2.582974434
C	-8.445995331	-0.254891753	2.776980639
C	-9.270983696	-0.547770321	1.520342112
N	-8.347790718	-0.712576330	0.416413426
C	-7.602260113	0.517103314	0.154566735
C	-6.645147324	0.767427742	1.311256289
C	-8.741203308	-1.420215726	-0.798584282
C	-9.254372597	-0.438897282	-1.879302740
C	-8.105916977	0.520712733	-2.271126986
C	-6.981362820	0.348198771	-1.244915009
C	-6.433617592	-1.046244860	-1.391765714
C	-7.474183559	-2.124608994	-1.317102551
O	-9.659701347	-1.113519669	-3.054377079
O	-5.889202118	1.214799285	-1.456363916
C	-4.746465206	0.468349308	-1.653845310
C	-5.124124527	-0.967798054	-1.621354699
O	-3.677559853	0.978465319	-1.827120304
H	-8.286946297	1.390405774	0.096431285
O	-6.719064713	1.005825043	3.759494066
C	-5.937421322	2.171544075	3.853930473
H	-8.087153435	1.851921439	2.474910736
H	-9.092688560	-0.048274226	3.634959698
H	-7.840659618	-1.136078954	3.013505697
H	-9.841117859	-1.472605944	1.653915882
H	-9.996800423	0.269593000	1.343301296
H	-5.966625690	-0.086001262	1.424683332
H	-6.054157257	1.667040944	1.114997149
H	-9.522213936	-2.147504807	-0.549891531
H	-10.091516495	0.132108018	-1.450916886
H	-8.433139801	1.563679695	-2.289319515
H	-7.749253750	0.251112282	-3.269486189
H	-7.170941353	-2.923532248	-0.636508286

H	-7.646640301	-2.553203821	-2.310716629
H	-10.449458122	-1.628679872	-2.854905367
H	-4.398623943	-1.750409007	-1.793145537
H	-6.546515942	3.074899197	3.700979471
H	-5.515861988	2.195104122	4.860302448
H	-5.113376141	2.182166815	3.129281998

==

Secu'amamine E (13)

==

C	-7.494416714	0.923014283	2.585191965
C	-8.444312096	-0.266031414	2.784416676
C	-9.267708778	-0.549069047	1.523202777
N	-8.344943047	-0.708647668	0.417795420
C	-7.601076603	0.522757947	0.152413905
C	-6.649172783	0.775812805	1.311668873
C	-8.740546227	-1.416700482	-0.796157897
C	-9.253718376	-0.435480654	-1.876974463
C	-8.104072571	0.520321310	-2.273441315
C	-6.980288506	0.347926974	-1.246175528
C	-6.434334278	-1.047263026	-1.393424153
C	-7.476044178	-2.124293804	-1.316211462
O	-9.665636063	-1.110354424	-3.050018311
O	-5.886038303	1.211978793	-1.459719896
C	-4.744706631	0.464438438	-1.658457279
C	-5.124882221	-0.971157372	-1.624566436
O	-3.675299883	0.972736835	-1.834328651
H	-8.288201332	1.394364595	0.094051033
H	-8.087059975	1.842813015	2.503358126
H	-9.109856606	-0.080445439	3.633872747
H	-7.858543396	-1.164085627	3.009732485
H	-9.842542648	-1.473042130	1.644952536
H	-9.988934517	0.274485052	1.351992607
H	-5.959911346	-0.072796091	1.397054315
H	-6.061465263	1.679565668	1.128340006
H	-9.523073196	-2.141775131	-0.546379507
H	-10.087223053	0.138509631	-1.445813656
H	-8.429538727	1.563622475	-2.294404507
H	-7.748292923	0.247580126	-3.271154165
H	-7.173241615	-2.923290968	-0.635344863
H	-7.650967121	-2.553103924	-2.309192657
H	-10.456656456	-1.622016549	-2.846478939
H	-4.402814865	-1.756965399	-1.796191931
H	-6.852071285	1.041084766	3.463216782

==

ent-Virosine B (44)

==

C	-9.538094521	1.942815542	0.939228594
C	-10.570762634	0.867969036	1.305903792
C	-9.904086113	-0.470132887	1.642237902
N	-9.048774719	-0.838709176	0.531380773
C	-7.972554207	0.136226565	0.341981232
C	-8.569241524	1.449367285	-0.144602194
C	-8.612509727	-2.223914385	0.366630226
C	-8.495825768	-2.411490440	-1.163406014
C	-7.609086037	-1.275101900	-1.720220327
C	-6.932077408	-0.561112285	-0.546427727
C	-6.271913052	-1.585642099	0.339147627
C	-7.237569332	-2.522362947	1.003365159
O	-7.878634930	-3.638845444	-1.505821228
O	-5.897955418	0.306287885	-0.959058642
C	-4.695909500	-0.138058394	-0.451873064
C	-4.963483810	-1.342926145	0.374774694
O	-3.663723707	0.425353527	-0.676741302
H	-7.464497089	0.352828681	1.305685759
H	-8.956037521	2.201304197	1.832685232
H	-11.240645409	0.703516960	0.454493761
H	-11.181871414	1.202722669	2.150223017
H	-9.338745117	-0.381114423	2.590842247
H	-10.663317680	-1.246893525	1.780880570
H	-7.773563862	2.178257704	-0.323757470
H	-9.103738785	1.281534672	-1.086693525
H	-9.383385658	-2.884486437	0.778207719
H	-9.503287315	-2.342199802	-1.590987444
H	-8.189163208	-0.558268905	-2.305945158
H	-6.842768669	-1.711299181	-2.365529776
H	-7.262440681	-2.337035656	2.083358288
H	-6.950370312	-3.564683676	0.846064806
H	-8.470138550	-4.357612133	-1.255301237
H	-4.175865650	-1.852967143	0.911145270
H	-10.044687271	2.858004808	0.617828906

Vibrational frequencies (in cm^{-1}) of the optimized structures.

==

Compound 30

==

46.62	63.14	68.48	92.57	134.13	156.23
184.45	215.12	247.97	263.58	277.54	302.39
316.99	344.64	354.71	382.23	385.26	406.89
429.27	454.16	492.32	503.62	527.72	569.85
585.66	600.78	666.90	672.71	716.37	758.72
776.08	805.04	824.94	859.75	863.77	880.98
898.94	922.22	947.00	960.41	975.48	978.60
999.60	1037.02	1080.87	1085.04	1091.82	1108.43
1122.70	1131.68	1154.45	1164.81	1174.55	1184.27
1187.04	1192.46	1197.48	1206.64	1226.18	1229.30
1237.89	1268.82	1280.01	1286.24	1290.32	1300.65
1316.69	1343.86	1347.47	1349.26	1368.71	1374.15
1383.91	1393.72	1399.33	1402.32	1420.61	1442.67
1443.96	1462.02	1475.54	1487.85	1495.94	1503.99
1512.51	1517.52	1530.22	1760.72	1935.91	2961.22
2995.97	3015.97	3031.75	3038.75	3090.21	3090.87
3093.29	3097.62	3098.89	3102.94	3112.81	3150.37
3152.63	3152.97	3161.33	3164.54	3293.43	3886.55

==

Compound 42

==

45.32	66.77	75.65	107.87	121.15	165.84
172.33	195.48	221.52	268.08	271.63	294.88
303.07	328.65	344.09	384.66	394.21	417.79
438.63	458.38	482.58	509.50	524.48	541.76
586.60	591.93	617.03	624.81	726.12	749.82
785.04	811.84	835.66	859.02	874.04	881.94
905.92	938.84	940.83	951.64	972.97	993.31
1012.58	1040.98	1075.36	1086.19	1096.39	1117.42
1133.57	1144.83	1160.35	1166.77	1173.69	1186.50
1190.47	1198.11	1204.02	1216.16	1225.81	1234.96
1256.65	1264.07	1275.32	1284.45	1285.95	1304.16
1307.06	1336.56	1345.38	1350.44	1355.41	1366.55
1380.60	1392.69	1402.71	1416.41	1421.39	1432.76
1453.70	1460.27	1485.39	1492.33	1495.54	1503.22
1509.93	1515.05	1527.81	1764.04	1937.56	2922.15
2957.37	2978.28	3027.82	3073.51	3081.51	3086.28
3089.02	3095.75	3101.77	3113.41	3114.63	3140.73
3153.23	3159.42	3168.71	3172.24	3291.94	3880.63

==

Compound 50

==

48.51	60.83	87.03	97.75	137.29	169.75
179.03	194.79	247.44	258.69	295.12	312.97
329.96	341.21	352.38	387.15	392.98	415.95
436.15	456.45	490.40	511.15	532.34	579.06
586.36	591.63	618.19	684.72	737.01	749.42
785.01	813.49	833.73	858.24	861.15	883.08
907.47	922.92	940.55	948.48	972.25	973.74
985.90	1027.38	1060.84	1083.62	1086.50	1116.36
1122.62	1137.17	1149.28	1165.96	1172.35	1187.20
1189.32	1191.85	1200.30	1211.92	1224.37	1234.22
1242.48	1262.59	1276.03	1282.50	1292.17	1305.39
1319.25	1343.83	1349.69	1355.77	1367.61	1372.58
1381.71	1392.07	1394.75	1401.52	1420.31	1432.32
1453.12	1459.56	1476.21	1487.15	1493.26	1495.93
1513.59	1516.54	1530.85	1764.25	1937.32	2968.77
3000.11	3015.65	3030.58	3071.80	3087.98	3090.44
3092.55	3099.71	3101.29	3112.69	3113.63	3150.34
3153.51	3154.69	3165.72	3170.62	3292.81	3880.78

==

Compound 51

==

52.22	60.69	82.16	108.67	128.73	164.63
179.64	199.83	237.92	249.35	274.07	288.55
310.18	321.10	338.71	376.01	396.95	405.17
439.51	460.94	487.15	499.10	517.64	530.81
567.07	586.59	625.61	671.01	695.10	755.98
774.28	805.69	827.81	852.01	874.24	883.89
906.10	928.64	954.57	967.26	986.81	996.08
1005.82	1054.38	1082.89	1086.09	1106.46	1106.90
1134.27	1140.59	1156.58	1169.15	1174.80	1181.22
1188.04	1197.04	1205.16	1217.55	1223.30	1233.36
1248.94	1268.60	1282.09	1284.21	1286.32	1299.95
1304.02	1337.68	1344.29	1345.60	1352.04	1369.40
1382.83	1398.14	1405.41	1416.41	1420.44	1439.85
1442.57	1461.94	1486.60	1493.48	1501.84	1503.59
1511.35	1514.79	1529.29	1762.65	1939.96	2913.37
2957.59	2966.78	3028.76	3036.58	3086.12	3092.88
3093.85	3094.32	3100.83	3104.03	3116.38	3148.73
3150.47	3154.93	3162.08	3167.30	3293.58	3885.48

==

Secu'amamine E (13)

==

67.90	89.61	114.73	144.21	194.66	227.19
266.00	278.93	308.39	312.81	324.49	358.02
382.57	398.07	411.87	460.92	474.38	506.46
516.74	565.94	586.50	611.25	665.76	695.70
756.62	772.69	797.80	822.20	847.43	864.74
870.62	884.52	909.57	927.72	954.85	968.08
975.59	987.56	1033.37	1074.59	1083.23	1086.17

1108.21	1109.80	1140.24	1160.86	1161.89	1172.31
1175.67	1195.35	1205.29	1224.27	1230.11	1262.38
1272.04	1285.96	1288.42	1292.23	1300.93	1338.45
1344.07	1347.65	1349.03	1370.59	1375.19	1390.62
1394.83	1399.76	1417.64	1439.62	1442.56	1461.62
1488.23	1494.18	1503.66	1505.26	1514.55	1762.48
1939.60	2912.93	2952.11	3037.65	3069.61	3080.25
3083.28	3094.00	3101.22	3104.96	3111.90	3125.25
3134.39	3147.99	3154.26	3163.05	3293.27	3884.80

==

ent-Virosine B (44)

==

68.43	93.67	115.78	147.94	194.32	208.75
261.39	290.47	315.65	319.09	338.73	351.83
386.67	398.56	435.79	458.02	486.64	508.73
519.13	578.95	586.60	605.75	616.34	726.44
748.07	784.40	805.45	832.85	852.82	864.39
876.34	884.36	908.14	941.04	941.45	951.07
972.52	978.25	1019.89	1063.92	1077.06	1086.95
1098.74	1118.19	1146.86	1154.78	1167.36	1172.58
1188.04	1194.60	1205.00	1225.91	1234.29	1258.52
1269.54	1277.39	1291.32	1294.71	1307.34	1335.55
1345.79	1353.63	1357.49	1364.59	1375.25	1385.76
1393.95	1397.73	1418.31	1432.60	1449.08	1459.56
1487.12	1491.44	1495.45	1505.87	1515.69	1764.47
1938.70	2920.64	2954.87	3070.51	3074.63	3080.45
3084.94	3089.43	3102.77	3111.75	3115.34	3125.86
3134.89	3147.08	3153.40	3172.58	3293.69	3881.64
